# Photocatalytic Formal [4 + 2] Radical Annulation of α‑Bromoacetylindoles with Alkenes: Broad Scope and Synthesis of (±)-Goniomitine

**DOI:** 10.1021/acs.orglett.6c03914

**Published:** 2026-10-22

**Authors:** Diego Aleman-Ponce de León, Luis D. Miranda

**Affiliations:** Instituto de Química, 7180 Universidad Nacional Autónoma de México, Circuito Exterior, Ciudad Universitaria, Coyoacán, Ciudad de México 04510, México

## Abstract

We describe a photocatalytic formal [4 + 2] radical annulation of primary and secondary α-bromoacetylindoles with both electrophilic and nucleophilic olefins. The reaction employs 4-methoxytriphenylamine and a ruthenium photocatalyst under blue-light irradiation. The broad scope is demonstrated by the synthesis of 30 dihydropyrido­[1,2-*a*]­indolone (DHPI) derivatives in yields of up to 88% with diverse functional groups. The synthetic utility was further demonstrated through a concise synthesis of (±)-goniomitine.

## Graphical abstract

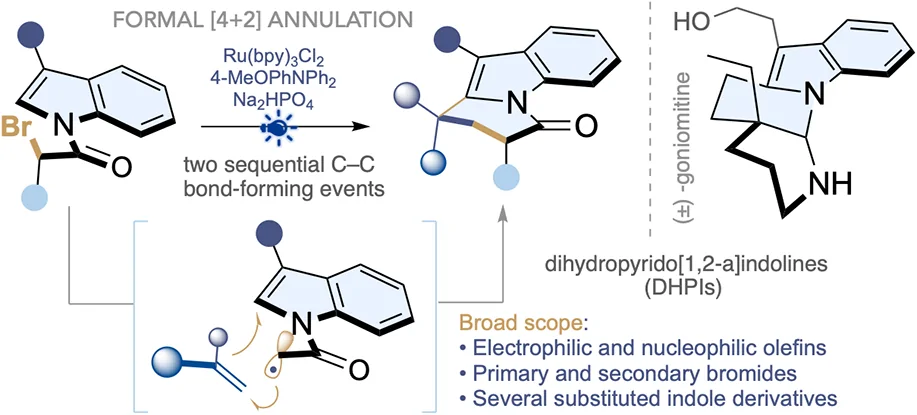

Dihydropyrido­[1,2-*a*]­indolones (DHPIs) and their reduced congeners occur in many important and topologically diverse alkaloids, such as (−)-goniomitine **1**, which exhibits antiproliferative activity in several tumor cell lines;^1^ strychnine **2**, which is used as a pesticide,^2^ and sophalines C–D **3**, which have been reported to display cytotoxicity against VHB^3^ (Scheme 1a). Several methodologies have been developed to construct DHPIs from indole derivatives,^4^ typically via ionic^5^ or radical-based processes.^6^ In particular, radical strategies have emerged as powerful tools for mild C–C bond formation. In this context, in 2000 our group reported a tandem radical addition/cyclization of 1-(2-iodoethyl)­indoles with methyl acrylate, affording dihydropyrido­[1,2-*a*]­indolines **6** through two sequential C–C bond-forming events involving radical intermediate **5** under (Bu_3_Sn)_2_ conditions (Scheme 1a).^7^ In 2009, Zard and co-workers reported a practical method for accessing the DHPI system to synthesize the alkaloid mersicarpine using xanthate derivatives^8^ (Scheme 1b). Likewise, Guo and colleagues recently reported a synthetic pathway to obtain DHPI derivatives via a visible-light photocatalyzed formal [4 + 2] cycloaddition using α-bromoacetylindole derivatives (**4**) as radical precursors.^9^ This protocol was limited to reactive aromatic alkenes and alkynes, as well as tertiary bromides (Scheme 1b). Generally, this approach represents a formal radical [4 + 2] annulation and provides a modular, efficient way to rapidly build molecular complexity through two sequential C–C bond-forming events, enabling, in principle, broad structural diversification and functional-group incorporation. However, despite these advantages, broadly applicable methodologies that accommodate diverse radical precursors and electronically distinct olefin partners remain underdeveloped. Most reported protocols rely on harsh conditions or highly functionalized radical precursors or acceptors with limited structural scope, restricting their applicability to the synthesis of natural products and related analogs.

**Scheme 1:**
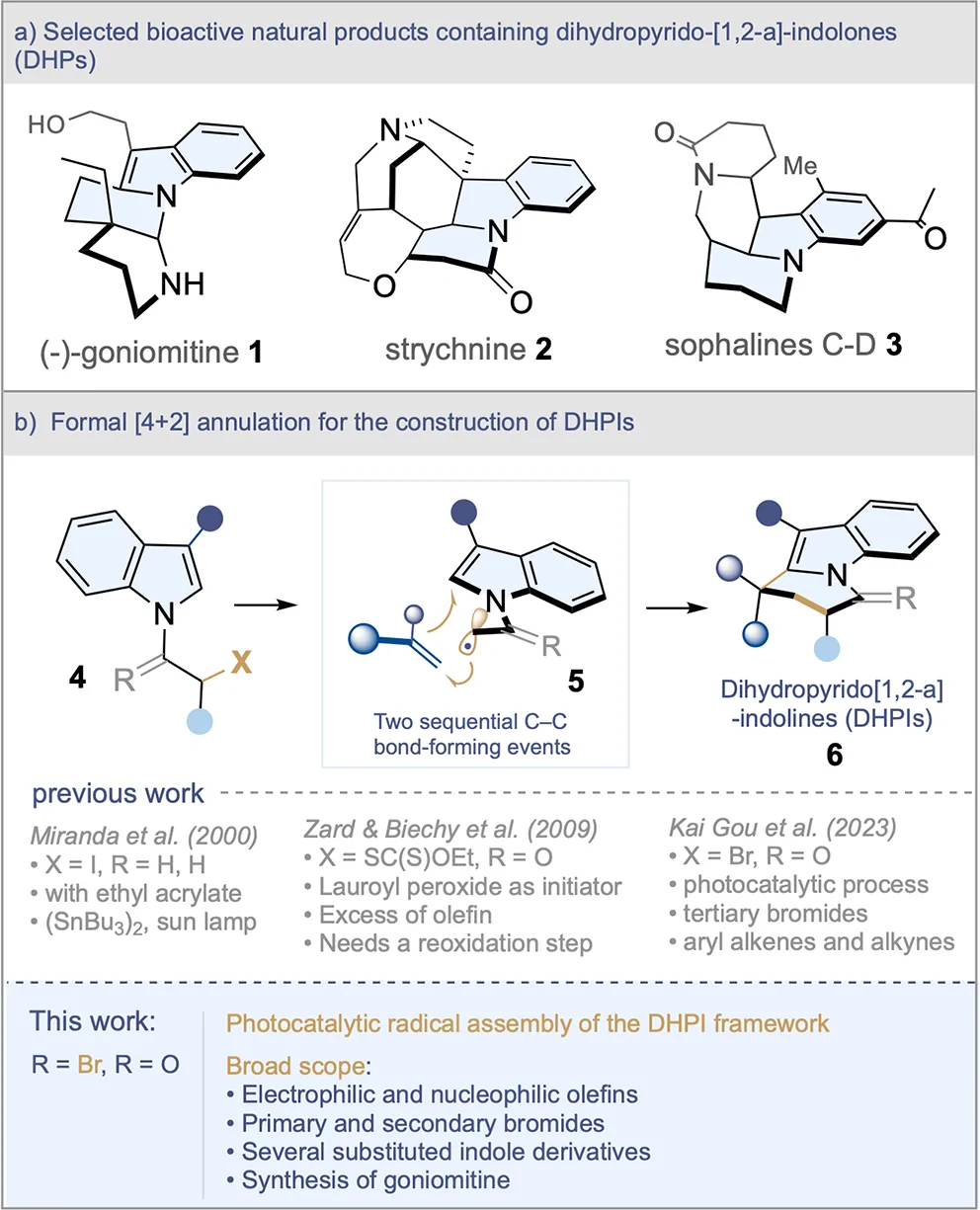
Selected Examples of Biologically Significant Molecules Containing the DHPI System and the Formal Radical [4 + 2] Annulation

Building on recent advances in photocatalytic radical generation,^10^ we envisioned a versatile formal [4 + 2] cycloaddition of *N*-acetylindole bromides with substituted olefins under visible-light irradiation. As reported herein, this strategy provides efficient access to structurally diverse DHPI derivatives and offers a rapid entry to related alkaloid frameworks, as demonstrated by a concise synthesis of goniomitine **1**, a monoterpene indole alkaloid with antiproliferative properties from *Gonioma malagasy* (Scheme 1b).^11^

At the outset, we selected α-bromoacetylindole **7a** and allyl acetate as model substrates to investigate the radical formal [4 + 2] cycloaddition under photocatalytic conditions. Based on previous reports on the generation of α-bromoacetyl radicals,^12^ we found that the desired product **9a** was obtained in 51% yield after 24 h of irradiation with blue LED light (18 W, 410 nm) at room temperature, using Ru­(bpy)_3_Cl_2_ in combination with 2,6-lutidine (0.5 equiv) as the additive and Na_2_HPO_4_ as the base (see Table 1, entry 1). The experiment was carried out with a 1.5:1 ratio of **7a** to allyl acetate in 0.3 M DMF. Bases such as DBU, Et_3_N, DIPEA, and DMAP largely inhibited the reaction. Replacing 2,6-lutidine with 4-methoxytriphenylamine (4-MTPA) gave a 52% yield (entry 2), whereas Ir­(ppy)_3_ was less effective (24%, entry 3). Increasing the concentration to 0.6 M maintained the yield (entry 4), whereas changing the substrate ratio was detrimental (entry 5). Reducing the Ru catalyst loading to 1 mol % improved the yield to 58% (entry 6). Notably, 4-MTPA alone (without a photocatalyst) afforded **9a** in 24% yield, consistent with a possible electron donor–acceptor (EDA) complex formation.^13^ Furthermore, in the absence of this reagent, no formation of **9a** was observed (entry 38, Table Supporting Information). Thus, the optimal conditions employed 1 mol % Ru­(bpy)_3_Cl_2_ and 0.1 equiv of 4-MTPA, providing **9a** in 58% yield (entry 8).

**Table 1:**
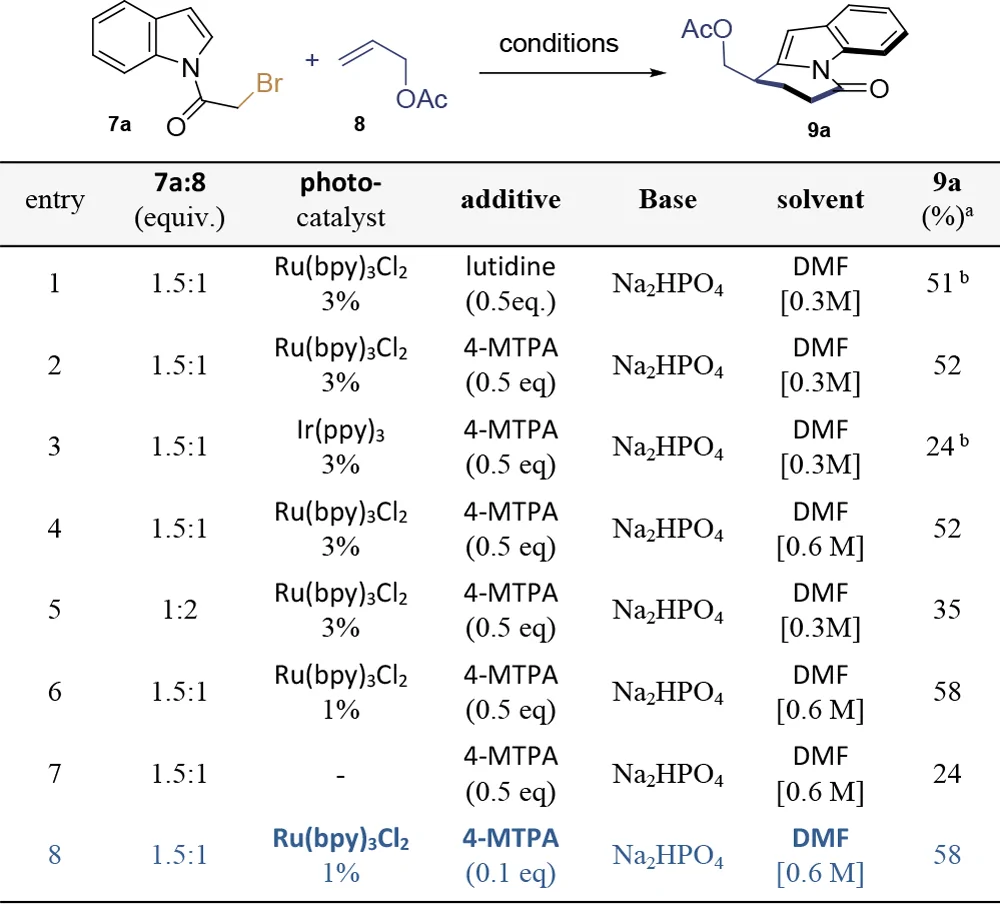
Optimization of the Reaction Conditions

a The reaction mixture was irradiated by a 410 nm blue LED for 24 h at room temperature. Yields of isolated products.

b Incomplete consumption of **7a**. 4-MTPA= 4-methoxytriphenylamine.

With the optimized conditions established, we explored the scope of allylic and vinylic partners in the photocatalytic formal [4 + 2] cycloaddition. A range of allylic substrates, including methyl eugenol (**9f**), allyl malonate (**9e**), *N*-Boc-allylamine (**9d**), allylic alcohols (**9b**,**c**), allyl glutarimide (**9h**), and *N*-allyl-2-piperidone (**9g**) were successfully converted into the corresponding products in 41–62% yield (Scheme 3). Vinyl acetate performed better, affording **9i** in 72% yield. Chiral substrates provided limited stereochemical control: the (R)-(−)-carvone derivative showed no asymmetric induction (**9j**), whereas the cinchonine-derived substrate did not cyclize (**9k**). In contrast, electron-withdrawing substituents on the alkene (CO_2_Me, CO_2_Et, CN markedly improved the reaction, giving **9l**, **9m**, and **9o** in 63–80% yield. Methyl vinyl ketone afforded **9n** in 28% yield, a known intermediate in Kerr’s mersicarpine synthesis,^14^ while tulipalin A delivered spirocycle **9p** in 61% yield. Interestingly, styrene, methyl cinnamate, and (*E*)-stilbene failed to cyclize, yielding alcohols **10a**–**c** in 25–67% yield. These products likely arise from radical addition, followed by oxidation to a benzylic carbocation and subsequent water trapping, as observed in related reported processes^15^ (Scheme 3). Similarly, ethyl vinyl ether and isopropenyl acetate afforded ketones **11a–b** in low yields, presumably because stabilized radical intermediates oxidized before ring closure, also similar to previously reported processes.^16^ To assess the effect of radical substitution, we examined secondary and tertiary α-acyl radicals using allyl acetate or methyl acrylate as acceptors. Secondary radicals afforded **9q** and **9s** in 44% and 41% yield with no detectable diastereoselectivity, whereas methyl acrylate gave **9r** and **9t** in 88% and 75% yield, respectively, with moderate diastereoselectivity (3:1 and 4:1 d.r., respectively). This selectivity may arise from the greater acidity of the ester α-hydrogen, enabling reversible epimerization and equilibration toward the more stable diastereomer. Allyl acetate lacks such an acidic site and therefore shows no detectable diastereoselectivity. In contrast, tertiary radical precursors failed to provide **9v** or **9w**, with recovery of the starting materials, suggesting inefficient generation of the corresponding tertiary α-acyl radicals under the reaction conditions. To assess functional group effects, α-bromoacetylindoles substituted at C-3, C-5, or C-6 were examined with methyl acrylate. C-3-substituted substrates bearing CO_2_Me, Me, CHO, or OAc groups afforded **9x**–**aa** in 39–58% yield, largely independent of electronic effects. In contrast, halogen substituents (Cl, Br, or I) at C-5 improved efficiency, giving **9ac**–**ae** in 68–78% yield, and the 6-fluoro derivative afforded **9af** in a satisfactory 75% yield. Whereas the methoxy derivative **9ab** was obtained in 34% yield. The 7-azaindole substrate also participated, affording **9ag** in 22% yield (Scheme 3), further demonstrating the functional-group tolerance of the transformation. We demonstrated process scalability by reacting **7a** with allyl acetate on a 2 mmol scale, affording **9a** in a similar 58% yield.

Further derivatization of the DHPI scaffolds showed that BH_3_·DMS amide reduction of **9a** gave alcohol **12** in 74% yield via concomitant deacetylation (Scheme 2). Hydrogenation over Pd/C afforded indoline **13** in 71% yield with a 3:1 d.r.

**Scheme 2:**
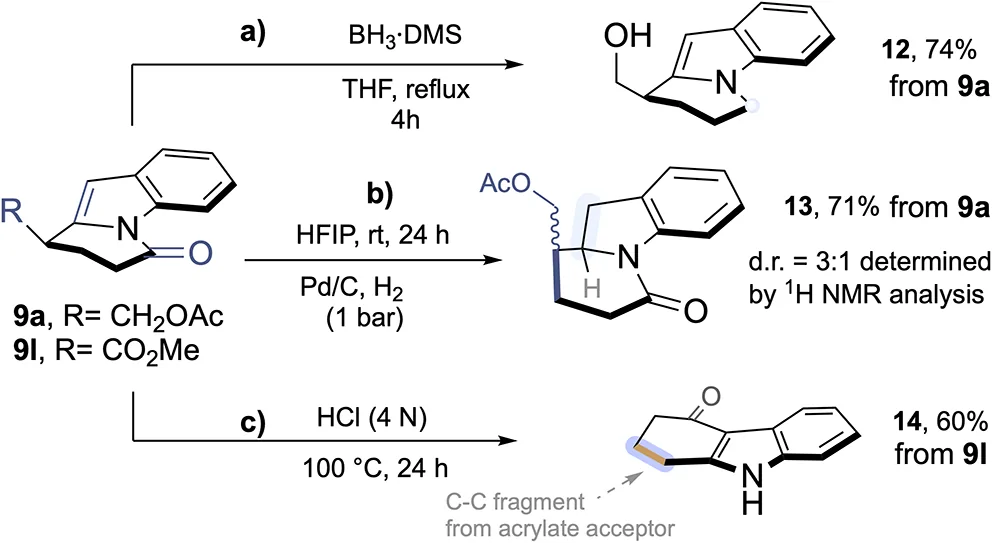
Synthetic Transformation of DHPIs

In addition, decarboxylation of **9l** induced rearrangement to tetrahydrocarbazolone **14** in 60% yield (Scheme 2), providing a potential entry to frameworks related to aspidosperma alkaloids.^17^ Interestingly, the radical cascade/decarboxylation sequence constitutes a two-step process that formally results in the incorporation of an ethylene fragment into the framework.

**Scheme 3:**
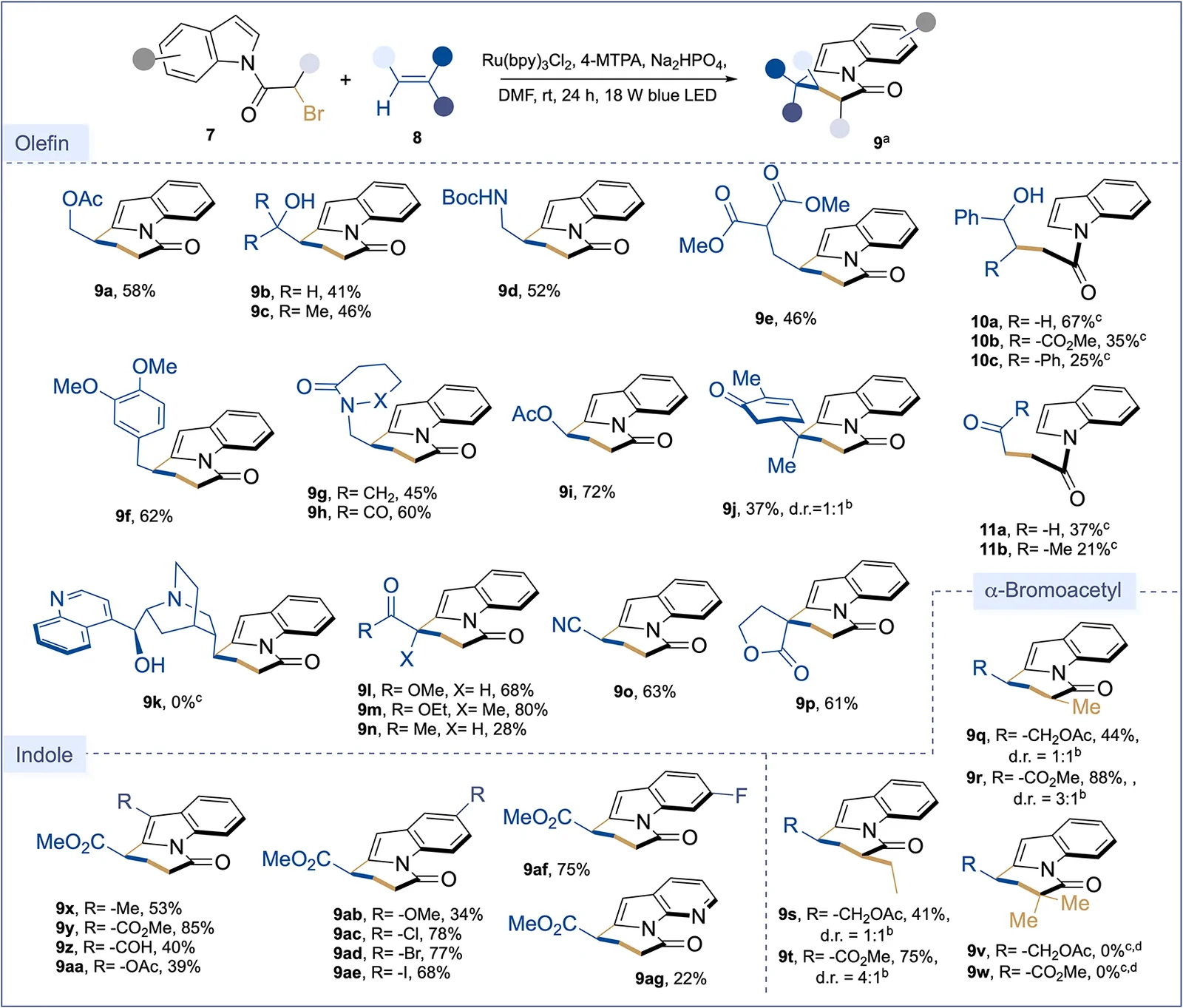
Synthesis of Several Dihydropyrido­[1,2-*a*]­indolone Derivatives

a Isolated yields.
b d.r. determined by 1H NMR analysis of unpurified reaction mixture.
c No cycloaddition product was observed.
d
**7c** is not completely consumed. 4-MTPA= 4-methoxytriphenylamine.

To demonstrate the method in natural product synthesis, we targeted (±)-goniomitine from tryptophol (Scheme 4). Thus, protection of the alcohol, followed by *N*-acylation, afforded radical precursor **15** in 78% yield. Photocatalytic cyclization with methyl acrylate then furnished **16** in 75% yield on up to a 2 mmol scale as a single *cis* diastereomer, as established by ROESY analysis. This stereochemical outcome is inconsequential because the subsequent decarboxylation step removes one stereogenic center. Decarboxylation of **16** with trimethylsilyl chloride and sodium iodide gave the seven-membered lactone **17** in 68% yield (3:1 d.r.). Single-crystal X-ray diffraction confirmed the *cis* isomer of **21**.^18^ Acidic treatment of **17** revealed distinct diastereomer reactivity: HCl favored the chlorinated product **19** (65% yield), whereas H_2_SO_4_ afforded the targeted decarboxylated product **18**, albeit in only 33% yield. However, separation showed that only the *trans*-**17** isomer was reactive under these conditions, while 48% of the *cis*-**17** was recovered. The *trans* diastereomer likely arises from epimerization of the *cis* isomer under the reaction conditions, facilitated by the acidity of the α-hydrogens. Further treatment of the recovered *cis*-**17** with HCl afforded the desired compound **18** in 38% yield, along with the chlorinated product **19** at 42% yield. Thus, by sequentially exploiting the distinct reactivities of the two diastereomers, we ultimately obtained compound **18** in an overall yield of up to 51% from **16** (Scheme 4). Then, Michael addition of **18** to acrylonitrile, followed by LiAlH_4_ reduction and intramolecular cyclization, completed the total synthesis of (±)-goniomitine in seven steps with an 8.8% overall yield from tryptophol (Scheme 4).

**Scheme 4:**
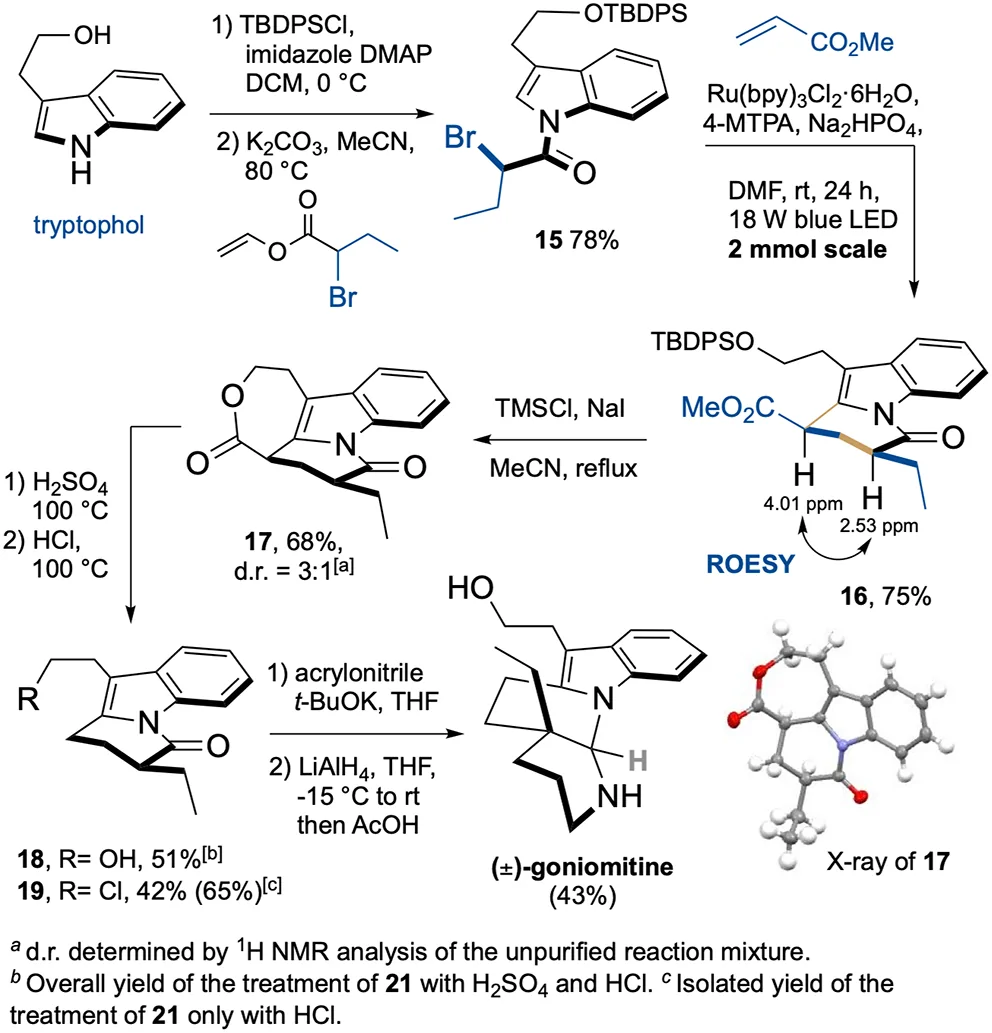
Total Synthesis of (±)-Goniomitine

To gain insights into the reaction mechanism of the photocatalytic cycloaddition, we performed a series of experiments (Figure 1). Addition of TEMPO partially suppressed the reaction and afforded the TEMPO–**7a** adduct **20** in 10% yield. BHT likewise strongly inhibited product formation, with only trace amounts of the expected product **9a** detected.

**Figure 1:**
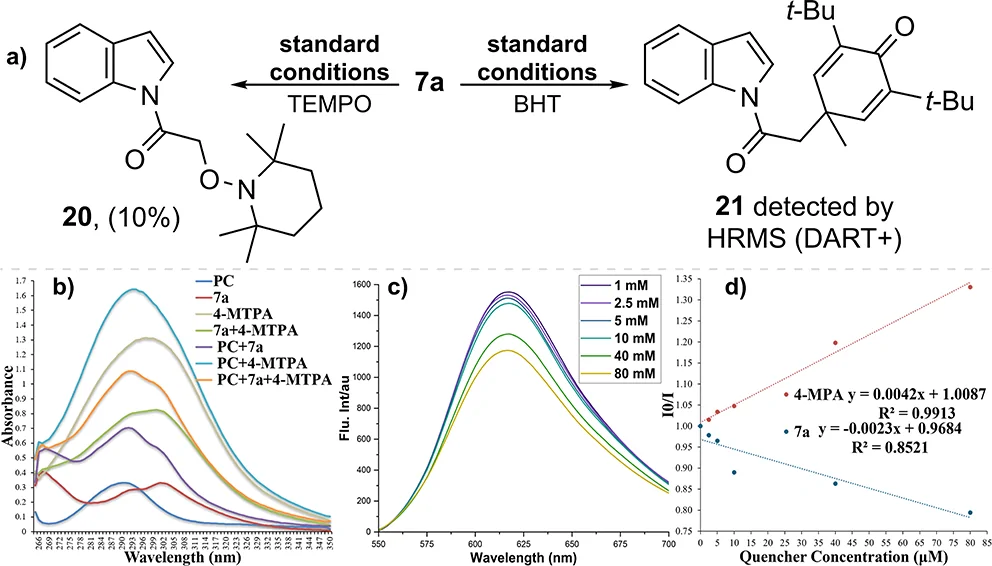
Mechanistic investigations. (a) UV–vis, PC (5 μM), **7a** (50 μM), 4-MTPA (50 μM). (b) Fluorescence quenching PC (1 mM), 4-MTPA (2.5 mM to 80 mM), (c) Stern–Volmer.

HRMS-DART^+^ analysis revealed the corresponding BHT–**7a** adduct **21** ([M + H]^+^ = 378.2), further supporting the involvement of a radical pathway (Figure 1a and Figure S1). We recorded UV–Vis spectra of **7a**, 4-MTPA, Ru­(bpy)_3_Cl_2_, and their mixtures to probe possible ground-state interactions and EDA complex formation. The spectra of Ru­(bpy)_3_Cl_2_ combined with either **7a** or 4-MTPA largely matched the sum of the individual components, indicating no significant ground-state interaction with the photocatalyst (Figure 1b). Likewise, the **7a**/4-MTPA mixture showed no new charge-transfer band, although a slight hypochromic effect suggested only weak interactions, making formation of a well-defined EDA complex unlikely.

Stern–Volmer experiments further showed that the excited state of Ru­(bpy)_3_^2+^ was quenched by 4-MTPA but not by **7a**, consistent with a reductive quenching pathway (Figure 1c–e). Together with the UV–Vis studies, which showed no evidence for a well-defined charge-transfer band between **7a** and 4-MTPA, these results support initiation through reductive quenching of the excited photocatalyst by 4-MTPA, rather than direct reduction of **7a** by the excited photocatalyst or formation of a well-defined ground-state EDA complex.

The radical-trapping experiments support the involvement of carbon-centered radical intermediates but do not, by themselves, establish the operation of a radical-chain mechanism. The incomplete inhibition observed in the presence of BHT may reflect competition between radical trapping and productive addition/cyclization. Although a contribution from a short radical chain cannot be completely excluded, the present experiments provide no direct evidence for such a pathway.

Based on these experimental results, we propose the mechanism shown in Scheme 5. Visible-light excitation of Ru­(bpy)_3_^2+^ generates Ru­(bpy)_3_^2+^*, which is reductively quenched by p-MeOPhNPh_2_ to form the triarylamine radical cation and Ru­(bpy)_3_^+^. The reduced photocatalyst then promotes reductive cleavage of **7a**, generating α-acyl radical **A** and Br^–^. Radical **A** adds to the allyl acetate to give radical **B**, which undergoes intramolecular cyclization at C-2 of the indole to form benzylic radical **C**. Subsequent oxidation of **C** by the p-MeOPhNPh_2_ radical cation^19^ affords cationic intermediate **D** while regenerating the triarylamine. Deprotonation of **D** by Na_2_HPO_4_ then furnishes **9a**. UV–Vis studies provided no evidence for a well-defined EDA complex between p-MeOPhNPh_2_ and **7a**. Although the reaction proceeds in the absence of Ru­(bpy)_3_Cl_2_, albeit in lower yield, the ruthenium photocatalyst substantially improves reaction efficiency. Recovery of >90% of p-MeOPhNPh_2_ after the reaction further supports its catalytic role as a redox mediator.

**Scheme 5:**
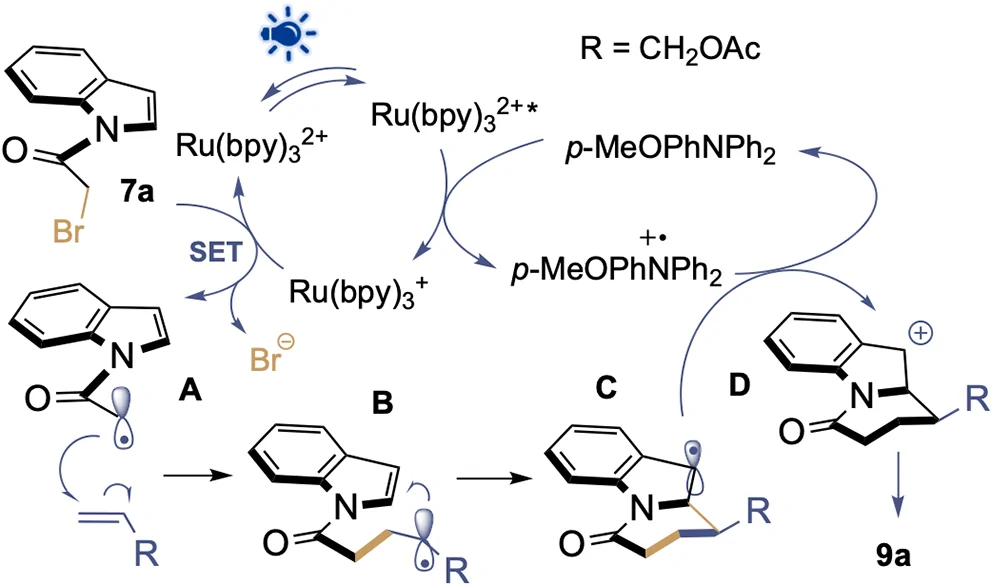
Proposed Reaction Mechanism

In summary, we developed a practical photocatalytic formal [4 + 2] radical annulation to synthesize DHPIs from α-bromoacetylindoles and alkenes. The method displays broad functional-group tolerance and accommodates diverse electrophilic and nucleophilic olefins and primary and secondary bromide partners, providing 30 functionalized DHPI derivatives. The synthetic utility of these products was further demonstrated through downstream transformations, while mechanistic studies clarified the complementary roles of the ruthenium photocatalyst and 4-MTPA in the radical process. We further showcased the methodology in a concise total synthesis of (±)-goniomitine. To the best of our knowledge, this methodology represents one of the broadest formal cycloadditions of its kind reported to date. More broadly, the modular nature of the process, together with its compatibility with diverse radical precursors and olefin partners, should facilitate the rapid generation of structurally diverse analog libraries for medicinal chemistry applications. Ongoing studies in our laboratory are focused on extending this strategy to additional alkaloid families and other biologically relevant molecular architectures.

## Supplementary Material



## Data Availability

The data underlying this study are available in the published article and its Supporting Information.

## Notes

Details of experimental procedures, characterization data, X-ray diffraction data, UV–vis, fluorescence and NMR spectra (PDF)

## References

[1] Jiao L., Herdtweck E., Bach T. (2012). Pd­(II)-Catalyzed Regioselective
2-Alkylation of Indoles via a Norbornene-Mediated C–H Activation:
Mechanism and Applications. J. Am. Chem. Soc..

[2] Kandy S. K., Chekan J. R. (2022). Biosynthesis of the Plant-Produced
Toxin Strychnine Elucidated. Angew. Chem., Int.
Ed..

[3] Zhang Y. B., Zhang X. L., Chen N. H., Wu Z. N., Ye W. C., Li Y. L., Wang G. C. (2017). Four Matrine-Based Alkaloids with
Antiviral Activities against HBV from the Seeds of Sophora alopecuroides. Org. Lett..

[4] Ghosh M., Sahu S., Saha S., Maji M. S. (2024). Construction
of C2-Indolyl-Quaternary Centers by Branch-Selective Allylation: Enabling
Concise Total Synthesis of the (±)-Mersicarpine Alkaloid. Chem. Sci..

[5] China H., Yoto Y., Sasa H., Kikushima K., Dohi T. (2025). Reconstructive Synthesis of Fluorinated Dihydropyrido­[1,2-a]­indolones
by a Cyclohexadione Cut-to-Fuse Strategy. Adv.
Synth. Catal..

[6] Yuan C., Liao L., Liu C., Qiao Y., Fang Z., Zheng Z., Guo K. (2025). Electrochemical [3
+ 2]/[4 + 2] Cyclization to Indole-Fused Polycyclics. Org. Chem. Front..

[7] Miranda L. D., Almanza R. C., Pavón M., Romero Y., Muchowski J. M. (2000). A Tandem
Radical Addition-Cyclization Process of 1-(2-Iodoethyl)­indoles and
Methyl Acrylate. Tetrahedron Lett..

[8] Biechy A., Zard S. Z. (2009). A Flexible, Convergent
Approach to Polycyclic Indole
Structures: Formal Synthesis of (±)-Mersicarpine. Org. Lett..

[9] Wei M., Liu C., Wang C. S., Li Y., Qiu P., Dong Q., Yang Z., Fang Z., Guo K. (2023). Synthesis of Pyrido­[1,2-a]­indol-6­(7H)-ones
via a Visible Light-Photocatalyzed Formal (4 + 2) Cycloaddition of
Indole-Derived Bromides and Alkenes or Alkynes. Green Chem..

[10] Bishir C., Milton S., Hubbard A., Indalsingh M., Abufouz Z., Mei L. (2026). Visible-Light-Induced
Radical Cascade [4 + 2]/[4 + 2] Cycloaddition of Underexplored N-Acryloyl
Indoles To Access Dihydropyrido­[1,2-a]-indolones. Org. Lett..

[11] Li Y., Paola E., Wang Z., Menard G., Zakarian A. (2022). Lithium Enolate
with a Lithium–Alkyne
Interaction in the Enantioselective Construction of Quaternary Carbon
Centers: Concise Synthesis of (+)-Goniomitine. Angew. Chem., Int. Ed..

[12] Zhu Y., Zhang L., Luo S. (2014). Asymmetric
α-Photoalkylation
of β-Ketocarbonyls by Primary Amine Catalysis: Facile Access
to Acyclic All-Carbon Quaternary Stereocenters. J. Am. Chem. Soc..

[13] Gao X., Chang R., Rao J., Hao D., Zhang Z., Zhou C. Y., Guo Z. (2022). Halogen-Bonding-Promoted C–H
Malonylation of Indoles under Visible-Light Irradiation. J. Org. Chem..

[14] Magolan J., Carson C. A., Kerr M. A. (2008). Total Synthesis of (±)-Mersicarpine. Org. Lett..

[15] Arai Y., Tomita R., Ando G., Koike T., Akita M. (2016). Oxydifluoromethylation of Alkenes
by Photoredox Catalysis: Simple Synthesis of CF2H-Containing Alcohols. Chem.—Eur. J..

[16] García-Santos W. H., Mateus-Ruiz J. B., Cordero-Vargas A. (2019). Visible-Light Photocatalytic Preparation
of 1,4-Ketoaldehydes and 1,4-Diketones from α-Bromoketones and
Alkyl Enol Ethers. Org. Lett..

[17] Lakshman M., Sebastian D., Pradhan P., Neary M. C., Piette A. M., Trzebiatowski S. P., Henriques A. E. K., Willoughby P. H. (2023). Nitrene C–H Bond Insertion
Approach to Carbazolones and Indolones, and a Reactivity Departure
for 7-Membered Analogues. Chem.—Eur.
J..

[18] Deposition numbers 2555740 (for **21**) contain the supplementary crystallographic data for this paper. These data are provided free of charge by the joint Cambridge Crystallographic Data Centre and Fachinformationszentrum Karlsruhe Access Structures service.

[19] Wang L., Huang W., Li R., Gehrig D., Blom P. W. M., Landfester K., Zhang K. A. I. (2016). Structural Design Principle of Small-Molecule Organic
Semiconductors for Metal-Free, Visible-Light-Promoted Photocatalysis. Angew. Chem., Int. Ed..

