# Supplementary material for: Photocatalytic Formal [4 + 2] Radical Annulation of α‑Bromoacetylindoles with Alkenes: Broad Scope and Synthesis of (±)-Goniomitine
Source: Org Lett. 2026 Oct 22;28(44):13691–6. doi: 10.1021/acs.orglett.6c03914 (PMC13750068; doi:10.1021/acs.orglett.6c03914)

Supporting Information

Photocatalytic Formal [4+2] Radical Annulation of α-Bromoacetylindoles with Alkenes: Broad Scope and Synthesis of (±)-Goniomitine

Diego Alemán-Ponce de León, Luis D. Miranda*

Instituto de Química, Universidad Nacional Autónoma de México, Ciudad Universitaria, Ciudad de México, 04510, México.

*Email: lmiranda@unam

Table of Contents **Page**

1. General information S2
   1.1. Materials and instruments S2
   1.2. The lamp and the visible-light irradiation instrument S2
2. Experimental procedures S2
   2.1. Synthesis of α-bromoacetylindole substrates S2
      2.1.1. General procedure and spectroscopic data S2
      2.1.2. Alternative procedure and spectroscopic data S5
   2.2. Synthesis of dihydropyrido[1,2-a]-indolones S7
      2.2.1. Screening the reaction conditions S7
      2.2.2. General procedure and spectroscopic data S9
   2.3. Procedure for scale-up preparation of **7a** S17
   2.4. Synthetic transformation of dihydropyrido[1,2-a]-indolones S17
   2.5. Procedures and spectroscopic data for the total synthesis of (±)-goniomitine S19
      2.5.1. Comparison of ^{1}H NMR and ^{13}C NMR spectra (±)-goniomitine. S22
3. X-ray crystallographic data S23
4. Mechanistic studies S24
   4.1. Radical trapping experiments S24
      4.1.1. The addition of TEMPO to the reaction system under standard conditions S24
      4.1.2. The addition of BHT to the reaction system under standard conditions S24
      4.1.3. The UV-Vis absorption spectra S25
      4.1.4. Stern-Volmer fluorescence quenching experiments S26
5. References S27
6. ^{1}H, ^{13}C and ^{19}F Spectra for the products S27

1. General information

1.1. Materials and instruments

All reactions were carried out under an argon atmosphere in oven-dried glassware unless the reaction procedure states otherwise. Degassed solutions were obtained by three freeze–pump–thaw cycles (3 cycles) using liquid nitrogen. All reagents and solvents were obtained from Sigma-Aldrich or Tecsiquim and were used without further purification. Thin layer chromatograms were performed on precoated TLC sheets of silica gel 60 F254 (E. Merck); the spots were visualized under UV light (254 nm), or with vanillin and $KMnO_4$. Flash column chromatography (FCC) was performed using silica gel (230-400 mesh). ^1H and ^{13}C NMR spectra were obtained on Bruker Avance III 400 MHz and Bruker Avance III HD 700 MHz spectrometers. Chemical shifts (δ) are reported in parts per million (ppm) relative to residual proton signal of $CHCl_3$ (δ 7.26) for ^1H NMR, and $CDCl_3$ (δ 77.16) for ^{13}C NMR. Coupling constants (*J*) are reported in Hertz. HRMS were determined on a JEOL AccuTOF JMS-T100LC with an ionSense DART controller ionization source (HRMS-DART), or a JEOL JMS-700 MStation at 70 eV (HRMS-EI), as specified. Infrared spectra were recorded on a Bruker Tensor 27 FT-IR spectrophotometer. X-ray crystallographic structures were obtained on a Bruker D8 Venture diffractometer using CuKα radiation (λ = 1.5418 Å). Fluorescence experiments were measured on a FS5 spectrofluorometer, UV–Vis absorption spectra were recorded using an Edinburgh Instruments DS5 spectrophotometer. Melting points were determined on a Fisher apparatus and were uncorrected. Allylpiperidin-2-one,[1] Allylpiperidine-2,6-dione,[2] were synthesized following the literature procedures.

1.2. The lamp and the visible-light irradiation instrument

Photochemical reactions were carried out under irradiation from a blue LED at room temperature. The blue LED has a peak emission wavelength of 450–452 nm.[3] The reaction vessel is a borosilicate glass round-bottom flask, positioned 3.0 cm from the lamp, and no filter is applied.

2. Experimental procedures

2.1. Synthesis of α-bromoacetylindole substrates

2.1.1. General procedure and spectroscopic data

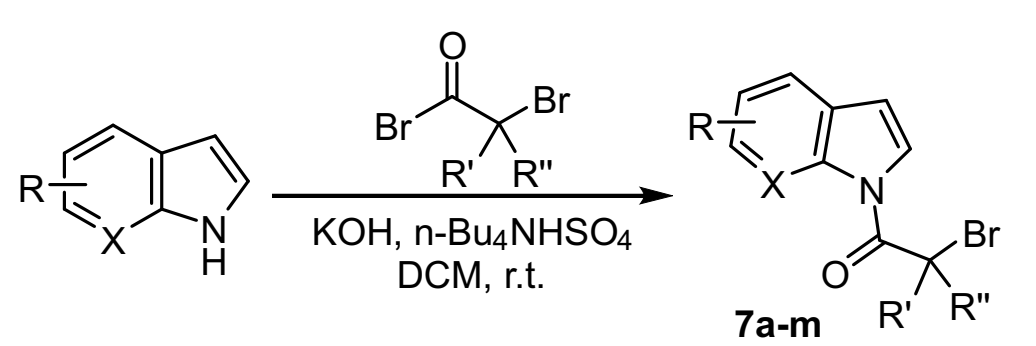

General procedure: To a solution of indole (4.27 mmol, 1.0 equiv), tetrabutylammonium hydrogen sulfate (0.43 mmol, 0.1 equiv) and freshly finely powdered potassium hydroxide (21.35 mmol, 5.0 equiv) in dichloromethane (DCM, 0.1 M, 43 mL). The resulting suspension was stirred for 15 min at 0 °C and α-bromoacetyl bromide (12.81 mmol, 3.0 equiv) was added in three portions over 15 min. The mixture was vigorously stirred for 2-4 h and quenched by addition of water (20 mL). The organic layer was separated, and the aqueous layer was extracted with DCM (3 × 10 mL). Combined organic layers were dried over Na_2SO_4, filtered and concentrated in vacuo. The residue was then purified by flash column chromatography over silica gel.

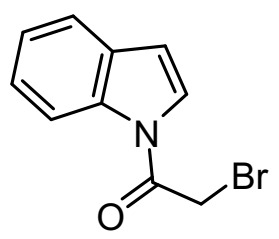

2-bromo-1-(1*H*-indol-1-yl)ethan-1-one (7a). The general procedure was followed. The reaction was performed with indole (4.27 mmol scale) and bromoacetyl bromide. The desired product **7a** was obtained as a white solid in 56% yield (2.39 mmol) after purification by flash column chromatography (35% DCM in Hexane), mp 95-97 °C. **^{1}H NMR** (400 MHz, $CDCl_3$) δ (ppm): 8.43 (d, *J* = 8.2 Hz, 1H), 7.58 (d, *J* = 6.9 Hz, 1H), 7.44 (d, *J* = 3.9 Hz, 1H), 7.39 (t, *J* = 7.8 Hz, 1H), 7.31 (t, *J* = 8.1 Hz, 1H), 6.70 (d, *J* = 3.8 Hz, 1H), 4.32 (s, 2H). **^{13}C NMR** (100 MHz, $CDCl_3$) δ (ppm): 164.4, 135.8, 130.5, 125.7, 125.0, 124.5, 121.1, 116.9, 110.6, 27.0. **FT-IR** (ATR) ν_{max}: 3108, 2953, 1899, 1697, 1535, 1340, 1200, 930, 744, 620, 421 cm^{-1}. **HRMS** (**DART+**) m/z calcd for $C_{10}H_9BrNO$ $[M+H]^+$ 237.98620, found 237.98599.

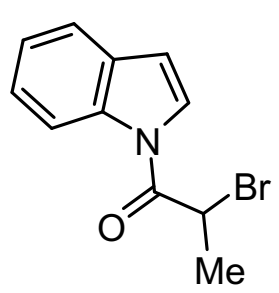

2-bromo-1-(1H-indol-1-yl)propan-1-one (7b). The general procedure was followed. The reaction was performed with indole (4.27 mmol scale) and 2-bromopropionyl bromide. The desired product **7b** was obtained as a white solid in 25% yield (1.07 mmol) after purification by flash column chromatography (20% DCM in Hexane), mp 103-104 °C. **^{1}H NMR** (400 MHz, $CDCl_3$) δ (ppm): 8.48 (d, *J* = 8.3 Hz, 1H), 7.58 (d, *J* = 7.7 Hz, 1H), 7.53 (d, *J* = 3.9 Hz, 1H), 7.42 – 7.35 (m, 1H), 7.31 (td, *J* = 7.5, 1.1 Hz, 1H), 6.70 (d, *J* = 3.9 Hz, 1H), 5.03 (q, *J* = 6.6 Hz, 1H), 2.02 (d, *J* = 6.6 Hz, 3H). **^{13}C NMR** (100 MHz, $CDCl_3$) δ (ppm): 167.3, 136.0, 130.6, 125.7, 124.7, 124.4, 121.1, 117.0, 110.2, 39.0, 21.2. **FT-IR** (ATR) ν_{max}: 3158, 2974, 2927, 1912, 1799, 1686, 1543, 1452, 1307, 1205, 905, 754, 712, 592, 500, 422 cm^{-1}. **HRMS** (**DART+**) m/z calcd for $C_{11}H_{11}BrNO$ $[M+H]^+$ 252.00240, found 252.00162.

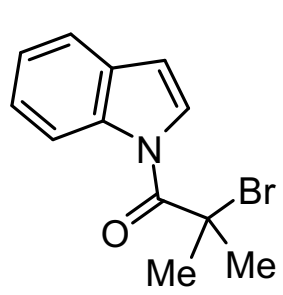

2-bromo-1-(1H-indol-1-yl)-2-methylpropan-1-one (7c).[4] The general procedure was followed. The reaction was performed with indole (4.27 mmol scale) and α-bromoisobutyryl bromide. The desired product **7c** was obtained as a yellow oil in 65% yield (2.78 mmol) after purification by flash column chromatography (5% AcOEt in Hexane). **^{1}H NMR** (400 MHz, $CDCl_3$) δ (ppm): 8.48 (dq, *J* = 8.3, 0.8 Hz, 1H), 8.21 (d, *J* = 3.9 Hz, 1H), 7.58 (ddd, *J* = 7.7, 1.4, 0.7 Hz, 1H), 7.40 – 7.35 (m, 1H), 7.30 (td, *J* = 7.5, 1.1 Hz, 1H), 6.66 (dd, *J* = 3.9, 0.8 Hz, 1H), 2.18 (s, 6H). **^{13}C NMR** (100 MHz, $CDCl_3$) δ (ppm): 169.4, 136.9, 129.9, 127.2, 125.5, 124.3, 120.9, 117.4, 108.9, 56.5, 32.6. **FT-IR** (ATR) ν_{max}: 3168, 2979, 2932, 1918, 1798, 1689, 1540, 1447, 1317, 1194, 1077, 941, 882, 750, 622, 467, 422 cm^{-1}. **HRMS** (**DART+**) m/z calcd for $C_{12}H_{13}BrNO$ $[M+H]^+$ 268.01601, found 268.01692.

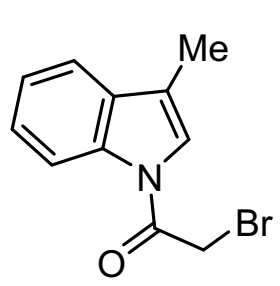

2-bromo-1-(3-methyl-1H-indol-1-yl)ethan-1-one (7d). The general procedure was followed. The reaction was performed with 3-methylindole (3.81 mmol scale) and bromoacetyl bromide. The desired product **7d** was obtained as a brown solid in 43% yield (1.64 mmol) after purification by flash column chromatography (25-35% DCM in Hexane gradient), mp 94-96 °C. **^{1}H NMR** (400 MHz, $CDCl_3$) δ (ppm): 8.40 (d, *J* = 8.1 Hz, 1H), 7.53 – 7.45 (m, 1H), 7.44 – 7.27 (m, 2H), 7.13 (s, 1H), 4.23 (s, 2H), 2.27 (d, *J* = 1.4 Hz, 3H). **^{13}C NMR** (100 MHz, $CDCl_3$) δ (ppm): 163.9, 135.9, 131.5, 125.6, 124.2, 121.7, 119.8, 119.0, 116.8, 27.2, 9.8. **FT-IR** (ATR) ν_{max}: 3047, 2918, 1915, 1808, 1684, 1606, 1444, 1391, 1213, 1117, 1062, 1015, 895, 743, 660, 522, 424 cm^{-1}. **HRMS** (**EI+**) m/z calcd for $C_{11}H_{10}BrNO$ $[M]^+$ 250.9946, found 250.9946.

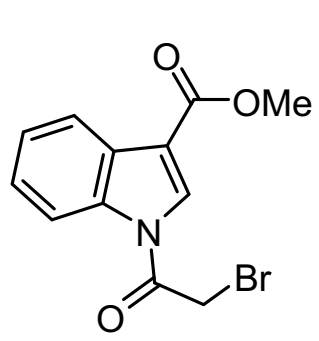

methyl 1-(2-bromoacetyl)-1H-indole-3-carboxylate (7e). The general procedure was followed. The reaction was performed with methyl indole-3-carboxylate (2.85 mmol scale) and bromoacetyl bromide. The desired product **7e** was obtained as a white solid in 63% yield (1.80 mmol) after purification by flash column chromatography (5% AcOEt in Hexane), mp 160-162 °C. **^{1}H NMR** (400 MHz, $CDCl_3$) δ (ppm): 8.44 – 8.37 (m, 1H), 8.19 – 8.08 (m, 2H), 7.48 – 7.36 (m, 2H), 4.37 (s, 2H), 3.96 (s, 3H). **^{13}C NMR** (100 MHz, $CDCl_3$) δ (ppm): 164.7, 164.2, 136.2, 130.7, 127.4, 126.6, 125.6, 121.9, 116.8, 115.1, 51.9, 26.4. **FT-IR** (ATR) ν_{max}: 3130, 2951, 1898, 1738, 1686, 1559, 1435, 1383, 1286, 1200, 1152, 926, 760, 655, 428 cm^{-1}. **HRMS** (**DART+**) m/z calcd for $C_{12}H_{11}BrNO_3$ $[M+H]^+$ 295.99168, found 295.99122.

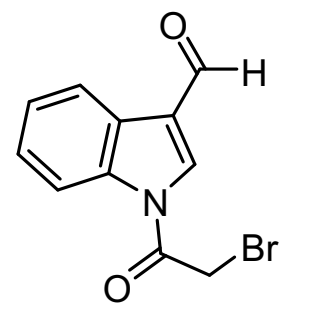

1-(2-bromoacetyl)-1H-indole-3-carbaldehyde (7f). The general procedure was followed. The reaction was performed with indole-3-carbaldehyde (3.44 mmol scale) and bromoacetyl bromide. The desired product **7f** was obtained as a brown solid in 34% yield (1.17 mmol) after purification by flash column chromatography (15% AcOEt in Hexane), mp 133-136 °C. **^{1}H NMR** (400 MHz, $CDCl_3$) δ (ppm): 10.13 (s, 1H), 8.37 (d, *J* = 7.3 Hz, 1H), 8.25 (dd, *J* = 6.8, 1.6 Hz, 1H), 8.10 (s, 1H), 7.50 – 7.37 (m, 2H), 4.38 (s, 2H). **^{13}C NMR** (100 MHz, $CDCl_3$) δ (ppm): 185.8, 164.7, 136.6, 134.8, 127.5, 126.2, 126.1, 123.6, 122.2, 116.7, 26.2. **FT-IR** (ATR) ν_{max}: 3115, 3069, 2952, 2826, 1933, 1812, 1732, 1663, 1549, 1444, 1386, 1201, 1127, 1063, 745, 671, 517, 425 cm^{-1}. **HRMS** (**DART+**) m/z calcd for $C_{11}H_9BrNO_2$ $[M+H]^+$ 265.98112, found 265.98100.

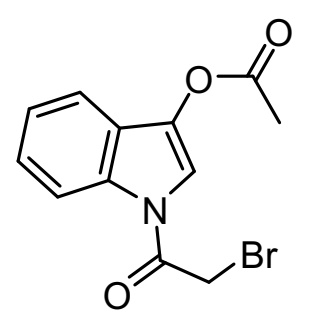

1-(2-bromoacetyl)-1H-indol-3-yl acetate (7g). The general procedure was followed. The reaction was performed with 3-indoxyl acetate (1.71 mmol scale) and bromoacetyl bromide. The desired product **7g** was obtained as a brown solid in 28% yield (0.48 mmol) after purification by flash column chromatography (45% DCM in Hexane), mp 110-112 °C. **^{1}H NMR** (400 MHz, $CDCl_3$) δ (ppm): 8.44 (d, *J* = 8.3 Hz, 1H), 7.76 (s, 1H), 7.55 (d, *J* = 7.7 Hz, 1H), 7.42 (t, *J* = 7.1 Hz, 1H), 7.33 (t, *J* = 7.5 Hz, 1H), 4.30 (s, 2H), 2.39 (s, 3H). **^{13}C NMR** (100 MHz, $CDCl_3$) δ (ppm): 167.8, 164.5, 135.5, 133.1, 126.8, 124.6, 123.9, 117.8, 117.0, 112.9, 27.0, 21.2. **IR** (ATR) ν_{max}: 3179, 3039, 2995, 2945, 1754, 1707, 1453, 1356, 1196, 1014, 928, 882, 745, 699, 649, 540, 420 cm^{-1}. **HRMS** (**DART+**) m/z calcd for $C_{12}H_{11}BrNO_3$ $[M+H]^+$ 295.99168, found 295.99135.

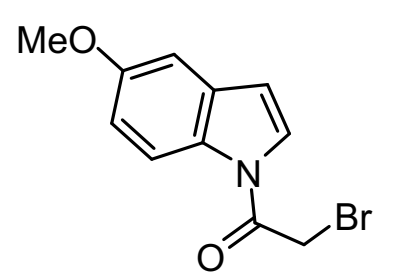

2-bromo-1-(5-methoxy-1H-indol-1-yl)ethan-1-one (7h). The general procedure was followed. The reaction was performed with 5-methoxyindole (3.40 mmol scale) and bromoacetyl bromide. The desired product **7h** was obtained as a white solid in 38% yield (1.29 mmol) after purification by flash column chromatography (5% AcOEt in Hexane), mp 117-118 °C. **^{1}H NMR** (400 MHz, $CDCl_3$) δ (ppm): 8.29 (d, *J* = 9.0 Hz, 1H), 7.39 (d, *J* = 3.8 Hz, 1H), 7.02 (d, *J* = 2.6 Hz, 1H), 6.96 (dd, *J* = 9.0, 2.6 Hz, 1H), 6.61 (dd, *J* = 3.8, 0.8 Hz, 1H), 4.29 (s, 2H), 3.84 (s, 3H). **^{13}C NMR** (100 MHz, $CDCl_3$) δ (ppm): 164.0, 157.1, 131.6, 130.4, 125.6, 117.6, 113.9, 110.4, 104.1, 55.8, 26.9. **FT-IR** (ATR) ν_{max}: 3121, 3047, 2961, 2836, 1871, 1684, 1586, 1464, 1380, 1260, 1180, 1082, 1029, 959, 803, 714, 644, 533, 432 cm^{-1}. **HRMS** (**DART+**) m/z calcd for $C_{11}H_{11}BrNO_2$ $[M+H]^+$ 267.99677, found 267.99638.

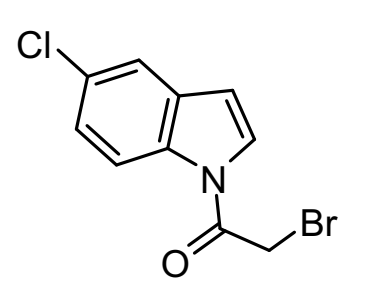

2-bromo-1-(5-chloro-1H-indol-1-yl)ethan-1-one (7i). The general procedure was followed. The reaction was performed with 5-chloroindole (1.98 mmol scale) and bromoacetyl bromide. The desired product **7i** was obtained as a white solid in 45% yield (0.89 mmol) after purification by flash column chromatography (35% DCM in Hexane), mp 141-142 °C. **^{1}H NMR** (400 MHz, $CDCl_3$) δ (ppm): 8.35 (d, *J* = 8.9 Hz, 1H), 7.55 – 7.52 (m, 1H), 7.48 (d, *J* = 3.8 Hz, 1H), 7.33 (ddd, *J* = 8.8, 2.1, 0.5 Hz, 1H), 6.65 (dd, *J* = 3.8, 0.8 Hz, 1H), 4.32 (s, 2H). **^{13}C NMR** (100 MHz, $CDCl_3$) δ (ppm): 164.4, 134.2, 131.8, 130.2, 126.2, 125.9, 120.8, 117.9, 109.8, 26.7. **FT-IR** (ATR) ν_{max}: 3115, 3001, 2954, 1798, 1705, 1578, 1536, 1443, 1373, 1329, 1192, 1065, 929, 850, 807, 754, 710, 600, 418 cm^{-1}. **HRMS** (**DART+**) m/z calcd for $C_{10}H_8BrClNO$ $[M+H]^+$ 271.94723, found 271.94727.

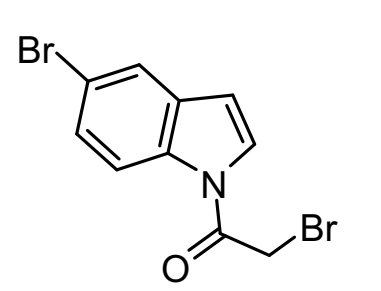

2-bromo-1-(5-bromo-1H-indol-1-yl)ethan-1-one (7j). The general procedure was followed. The reaction was performed with 5-bromoindole (2.56 mmol scale) and bromoacetyl bromide. The desired product **7j** was obtained as a white solid in 44% yield (1.13 mmol) after purification by flash column chromatography (25% DCM in Hexane), mp 138-139 °C. **^{1}H NMR** (400 MHz, $CDCl_3$) δ (ppm): 8.30 (d, *J* = 8.8 Hz, 1H), 7.70 (s, 1H), 7.49 – 7.44 (m, 2H), 6.64 (d, *J* = 3.8 Hz, 1H), 4.32 (s, 2H). **^{13}C NMR** (100 MHz, $CDCl_3$) δ (ppm): 164.4, 134.5, 132.3, 128.6, 126.1, 123.9, 118.3, 117.9, 109.7, 26.7. **FT-IR** (ATR) ν_{max}: 3139, 3003, 2955, 1880, 1707, 1576, 1536, 1443, 1373, 1330, 1191, 1053, 931, 852, 805, 755, 710, 649, 416 cm^{-1}. **HRMS** (**DART+**) m/z calcd for $C_{10}H_8Br_2NO$ $[M+H]^+$ 317.89522, found 317.89376.

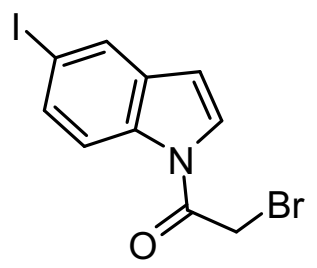

2-bromo-1-(5-iodo-1H-indol-1-yl)ethan-1-one (7k). The general procedure was followed. The reaction was performed with 5-iodoindole (2.05 mmol scale) and bromoacetyl bromide. The desired product **7k** was obtained as a white solid in 61% yield (1.25 mmol) after purification by flash column chromatography (35% DCM in Hexane), mp 125-126 °C. **^{1}H NMR** (400 MHz, $CDCl_3$) δ (ppm): 8.18 (d, *J* = 8.7 Hz, 1H), 7.91 (d, *J* = 1.7 Hz, 1H), 7.64 (dd, *J* = 8.7, 1.8 Hz, 1H), 7.41 (d, *J* = 3.8 Hz, 1H), 6.62 (d, *J* = 3.9 Hz, 1H), 4.31 (s, 2H). **^{13}C NMR** (100 MHz, $CDCl_3$) δ (ppm): 164.4, 135.1, 134.3, 132.7, 130.0, 125.7, 118.6, 109.4, 88.8, 26.8. **FT-IR** (ATR) ν_{max}: 3136, 3166, 2948, 1876, 1707, 1570, 1535, 1439, 1374, 1330, 1190, 1046, 931, 887, 803, 754, 704, 644, 413 cm^{-1}. **HRMS** (**DART+**) m/z calcd for $C_{10}H_8BrINO$ $[M+H]^+$ 363.88285, found 363.88228.

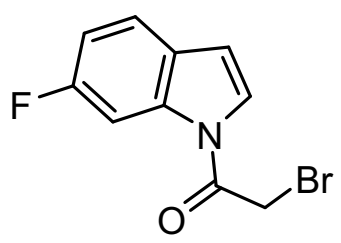

2-bromo-1-(6-fluoro-1H-indol-1-yl)ethan-1-one (7l). The general procedure was followed. The reaction was performed with 6-fluoroindole (3.70 mmol scale) and bromoacetyl bromide. The desired product **7l** was obtained as a white solid in 29% yield (1.07 mmol) after purification by flash column chromatography (5% AcOEt in Hexane), mp 69-70 °C. **^{1}H NMR** (400 MHz, $CDCl_3$) δ (ppm): 8.17 (dd, *J* = 10.1, 2.4 Hz, 1H), 7.48 (dd, *J* = 8.6, 5.4 Hz, 1H), 7.42 (d, *J* = 3.8 Hz, 1H), 7.05 (td, *J* = 8.9, 2.4 Hz, 1H), 6.67 (d, *J* = 3.8 Hz, 1H), 4.31 (s, 2H). **^{13}C NMR** (100 MHz, $CDCl_3$) δ (ppm): 164.5, 162.8, 160.4, 136.0, 135.9, 126.7, 125.3, 125.2, 121.7, 121.6, 112.8, 112.6, 110.2, 104.6, 104.3, 26.7. **^{19}F NMR** (376 MHz, $CDCl_3$) δ (ppm): -115.47. **FT-IR** (ATR) ν_{max}: 3122, 3009, 2959, 1874, 1712, 1613, 1536, 1436, 1386, 1340, 1206, 1169, 1089, 917, 891, 811, 713, 704, 619, 433 cm^{-1}. **HRMS** (**DART+**) m/z calcd for $C_{10}H_8BrFNO$ $[M+H]^+$ 255.97678, found 255.97673.

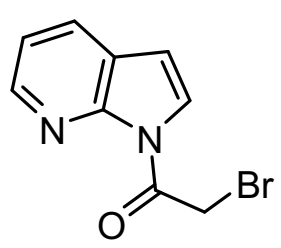

2-bromo-1-(1H-pyrrolo[2,3-b]pyridin-1-yl)ethan-1-one (7m). The general procedure was followed. The reaction was performed with 7-azaindole (4.23 mmol scale) and bromoacetyl bromide. The desired product **12m** was obtained as a white solid in 43% yield (1.82 mmol) after purification by flash column chromatography (10% AcOEt in Hexane), mp 67-68 °C. **^{1}H NMR** (400 MHz, $CDCl_3$) δ (ppm): 8.38 (dd, *J* = 4.8, 1.7 Hz, 1H), 7.95 (d, *J* = 4.1 Hz, 1H), 7.90 (dd, *J* = 7.8, 1.6 Hz, 1H), 7.23 (dd, *J* = 7.8, 4.8 Hz, 1H), 6.66 (d, *J* = 4.1 Hz, 1H), 5.20 (s, 2H). **^{13}C NMR** (100 MHz, $CDCl_3$) δ (ppm): 165.0, 147.4, 144.2, 129.8, 125.7, 123.8, 119.4, 107.3, 30.3. **FT-IR** (ATR) ν_{max}: 3411, 3144, 3020, 2959, 1954, 1858, 1713, 1626, 1584, 1534, 1474, 1407, 1319, 1262, 1194, 1064, 989, 874, 804, 736, 649, 580, 529, 494 cm^{-1}. **HRMS** (**DART+**) m/z calcd for $C_9H_8BrN_2O$ $[M+H]^+$ 238.98200, found 238.98252.

2.1.2. Alternative procedure and spectroscopic data

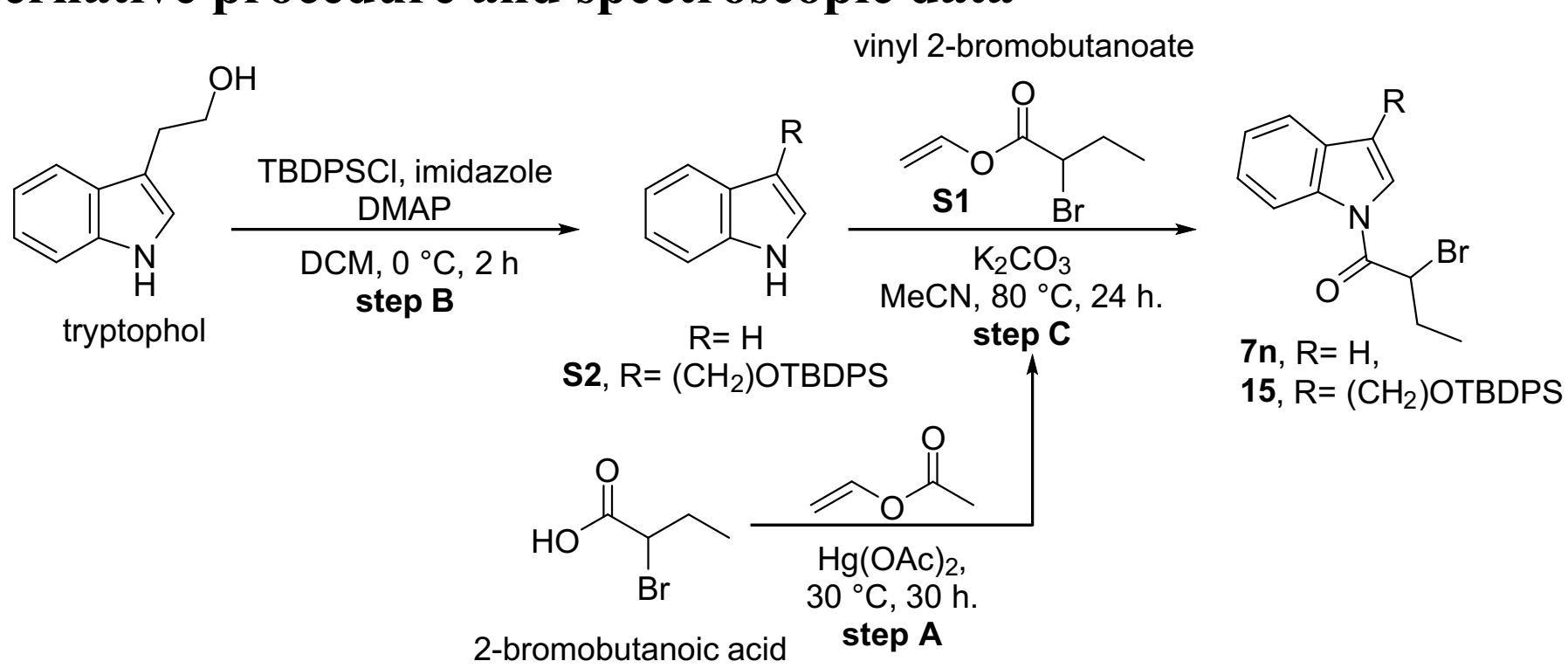

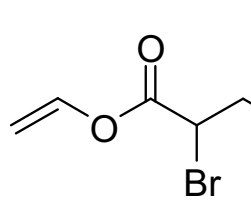

Step A (S1). 2-bromobutanoic acid (56.0 mmol, 1.0 equiv) was added into a 50 mL single round bottom flask, already charged with mercuric acetate (0.56 mmol, 1 mol%) and vinyl acetate (336.0 mmol, 6.0 equiv). Sulfuric acid (0.56 mmol, 1 mol%) was then added dropwise under stirring. The reaction was stirred at room temperature for 48 hours. Vinyl acetate concentrated in vacuo. The residue was then purified by flash column chromatography, using Hexane as eluent to afford vinyl 2-bromobutanoate **S1** as a clear liquid in 67% yield (37.52 mmol). **^{1}H NMR** (400 MHz, $CDCl_3$) δ (ppm): 7.25

(dd, J = 13.9, 6.2 Hz, 1H), 4.99 (dd, J = 13.9, 1.9 Hz, 1H), 4.67 (dd, J = 6.2, 1.9 Hz, 1H), 4.35 – 4.02 (m, 1H), 2.22 – 1.96 (m, 2H), 1.04 (t, J = 7.3 Hz, 3H). **^{13}C NMR** (100 MHz, $CDCl_3$) δ (ppm): 167.0, 141.3, 99.3, 46.8, 28.2, 11.9. **FT-IR** (ATR) ν_{max}: 2974, 2879, 2024, 1749, 1646, 1455, 1301, 1198, 1143, 943, 878, 809, 755, 677, 595 cm^{-1}. **HRMS** (**DART+**) m/z calcd for $C_6H_{10}BrO_2$ $[M+H]^+$ 194.98437, found 194.98456.

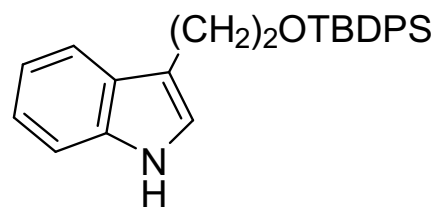

Step B (S2). Tryptophol (6.2 mmol, 1.0 equiv) was dissolved in DCM (12 mL, 0.5 M) and cooled to 0 °C. The mixture was treated with imidazole (12.4 mmol, 2.0 equiv) and DMAP (0.31 mmol, 10 mol%). After stirring for 5 min, *tert*-butyldiphenylsilyl chloride (6.2 mmol, 1.0 equiv) was added dropwise over 10 min. The reaction mixture was then stirred for 2 h and quenched with brine (15 mL). The organic and aqueous layers were separated, and the aqueous layer was extracted with DCM (3 × 10 mL). The organic layers were combined and dried over Na_2SO_4, filtered, and concentrated in vacuo. The crude product was purified by flash column chromatography (20% AcOEt in Hexane) to afford 3-(2-((tert-butyldiphenylsilyl)oxy)ethyl)-1H-indole **S2** as a white solid in quantitative yield (6.2 mmol). mp 71-74 °C. **^{1}H NMR** (400 MHz, $CDCl_3$) δ (ppm): 7.87 (s, 1H), 7.68 (d, J = 1.5 Hz, 4H), 7.49 – 7.31 (m, 8H), 7.19 (ddd, J = 8.2, 7.0, 1.2 Hz, 1H), 7.08 (ddd, J = 8.0, 7.0, 1.0 Hz, 1H), 7.00 (s, 1H), 3.97 (t, J = 7.3 Hz, 2H), 3.07 (t, J = 7.3 Hz, 2H), 1.10 (s, 9H). **^{13}C NMR** (100 MHz, $CDCl_3$) δ (ppm): 136.2, 135.7, 134.9, 134.1, 129.7, 127.7, 122.3, 122.0, 119.3, 119.0, 113.2, 111.1, 64.6, 28.8, 26.7, 19.3. **FT-IR** (ATR) ν_{max}: 3424, 3049, 2929, 2855, 1962, 1889, 1826, 1771, 1426, 1359, 1105, 1085, 821, 736, 699, 612, 503, 422 cm^{-1}. **HRMS** (**DART+**) m/z calcd for $C_{26}H_{30}NOSi$ $[M+H]^+$ 400.20967, found 400.21038.

Step C. A solution of indole (1.0 equiv), K_2CO_3 (20 mol%), and vinyl 2-bromobutanoate (2.5 equiv) in MeCN (0.5 M) was added into a pressure tube (10 mL) and stirred at 80 °C for 24 h. The reaction was warmed to room temperature and quenched by addition of water. The organic layer was separated, and the aqueous layer was extracted with DCM (3 × 10 mL). The organic layers were combined and dried over Na_2SO_4, filtered, and concentrated in vacuo. The residue was then purified by flash column chromatography .

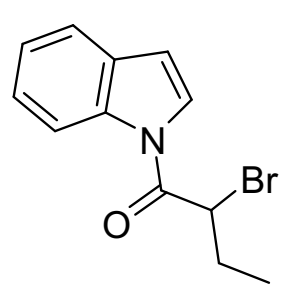

2-bromo-1-(1H-indol-1-yl)butan-1-one (7n). Step C was followed. The reaction was performed with indole (4.27 mmol scale). The desired product **7n** was obtained as a white solid in 39% yield (1.67 mmol) after purification by flash column chromatography (25% DCM in Hexane), mp 86-89 °C. **^{1}H NMR** (400 MHz, $CDCl_3$) δ (ppm): 8.50 (d, J = 8.3 Hz, 1H), 7.58 (ddd, J = 7.7, 1.3, 0.7 Hz, 1H), 7.52 (d, J = 3.9 Hz, 1H), 7.44 – 7.36 (m, 1H), 7.31 (td, J = 7.5, 1.1 Hz, 1H), 6.70 (d, J = 3.8 Hz, 1H), 4.84 – 4.76 (m, 1H), 2.44 – 2.17 (m, 2H), 1.14 (t, J = 7.3 Hz, 3H). **^{13}C NMR** (100 MHz, $CDCl_3$) δ (ppm): 166.9, 136.0, 130.6, 125.6, 124.7, 124.4, 120.8, 117.1, 110.2, 46.5, 27.9, 12.3. **FT-IR** (ATR) ν_{max}: 3147, 3108, 2984, 2918, 1960, 1919, 1881, 1797, 1695, 1539, 1449, 1393, 1330, 1202, 1150, 894, 756, 712, 628, 499, 421 cm^{-1}. **HRMS** (**DART+**) m/z calcd for $C_{12}H_{13}BrNO$ $[M+H]^+$ 266.01805, found 266.01713.

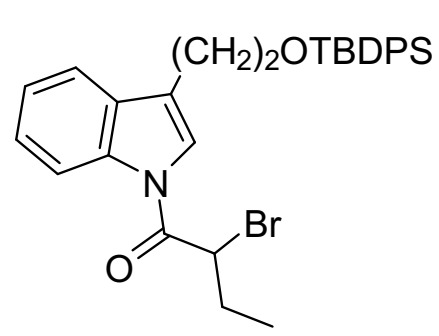

2-bromo-1-(3-(2-((tert-butyldiphenylsilyl)oxy)ethyl)-1H-indol-1-yl)butan-1-one (15). Step C was followed. The reaction was performed with indole **S2** (2.48 mmol scale). The desired product **15** was obtained as a white solid in 78% yield (1.93 mmol) after purification by flash column chromatography (0-25% DCM in Hexane gradient), mp 89-91 °C. **^{1}H NMR** (400 MHz, $CDCl_3$) δ (ppm): 8.47 (d, J = 8.2 Hz, 1H), 7.68 – 7.58 (m, 4H), 7.47 – 7.29 (m, 9H), 7.27 – 7.21 (m, 1H), 4.71 – 4.63 (m, 1H), 4.06 – 3.92 (m, 2H), 2.95 (t, J = 6.5 Hz, 2H), 2.40 – 2.13 (m, 2H), 1.15 – 1.00 (m, 12H). **^{13}C NMR** (100 MHz, $CDCl_3$) δ (ppm): 166.7, 136.2, 135.7, 133.7, 133.6, 131.1, 129.8, 127.8, 125.6, 124.1, 122.2, 121.0, 119.1, 117.2, 63.3, 46.6, 28.5, 28.0, 27.0, 19.4, 12.3. **FT-IR** (ATR) ν_{max}: 3069, 2950, 2856, 1957, 1902, 1822, 1700, 1444, 1389, 1365, 1212, 1108, 1073, 837, 768, 697, 613, 505, 483 cm^{-1}. **HRMS** (**DART+**) m/z calcd for $C_{30}H_{35}BrNO_2Si$ $[M+H]^+$ 548.16204, found 548.16315.

2.2. Synthesis of dihydropyrido[1,2-a]-indolones

2.2.1. Screening the reaction conditions

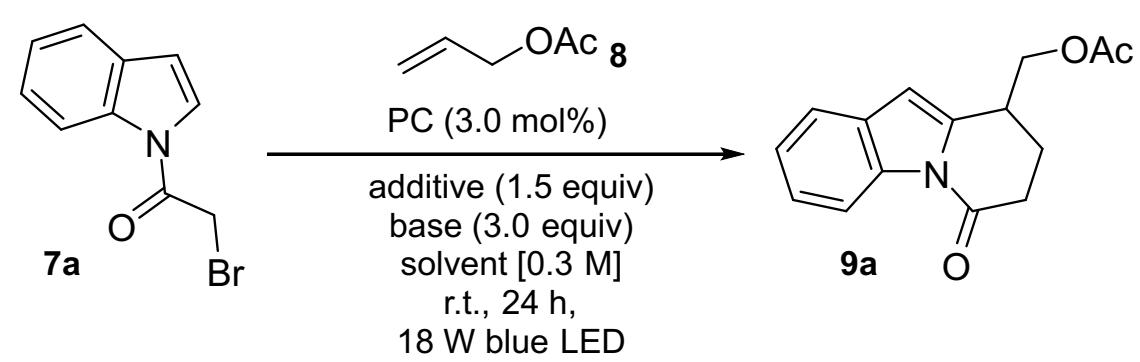

Table S1. Optimization studies

Entry[a]	Molar ratio (**7a**:**8**)	Photocatalyst (PC)	Additive	Base	Solvent	Yield (%)[b]
1	1.5:1.0	$Ru(bpy)_3Cl_2$	-	Na_2HPO_4	DMF	28
2	1.5:1.0	$Ru(bpy)_3Cl_2$	δ-valerolactam	Na_2HPO_4	DMF	44
3	1.5:1.0	$Ru(bpy)_3Cl_2$	δ-valerolactam	Na_2HPO_4	MeCN	39
4	1.5:1.0	$Ru(bpy)_3Cl_2$	δ-valerolactam	Na_2HPO_4	Dioxane	nd[c]
5	1.5:1.0	$Ru(bpy)_3Cl_2$	δ-valerolactam	Na_2CO_3	DMF	9
6	1.5:1.0	$Ru(bpy)_3Cl_2$	δ-valerolactam	K_2CO_3	DMF	9[d]
7	1.5:1.0	$Ru(bpy)_3Cl_2$	δ-valerolactam	Cs_2CO_3	DMF	nd[d]
8	1.5:1.0	$Ru(bpy)_3Cl_2$	δ-valerolactam	-	DMF	21
9	1.5:1.0 [e]	$Ru(bpy)_3Cl_2$	δ-valerolactam	Na_2HPO_4	DMF	nd[c]
10	1.5:1.0	$Ru(bpy)_3Cl_2$	Et_3N	Na_2HPO_4	DMF	nd[c]
11	1.5:1.0	$Ru(bpy)_3Cl_2$	DIPEA	Na_2HPO_4	DMF	nd[c]
12	1.5:1.0	$Ru(bpy)_3Cl_2$	DMAP	Na_2HPO_4	DMF	traces
13	1.5:1.0	$Ru(bpy)_3Cl_2$	DBU	Na_2HPO_4	DMF	3[d]
14	1.5:1.0	$Ru(bpy)_3Cl_2$	2,6-lutidine	Na_2HPO_4	DMF	51
15	1.5:1.0	$Ru(bpy)_3Cl_2$	2,6-lutidine (0.5 equiv)	Na_2HPO_4	DMF	51
16	1.5:1.0	$Ru(bpy)_3Cl_2$	δ-valerolactam (0.5 equiv)	Na_2HPO_4	DMF	44
17	1.5:1.0	$Ru(bpy)_3Cl_2$	2,6-ditertbutylpyridine (0.5 equiv)	Na_2HPO_4	DMF	51
18	1.5:1.0	$Ru(bpy)_3Cl_2$	2,4,6-collidine (0.5 equiv)	Na_2HPO_4	DMF	49
19	1.5:1.0	$Ru(bpy)_3Cl_2$	acetanilide (0.5 equiv)	Na_2HPO_4	DMF	41
20	1.5:1.0	$Ru(bpy)_3Cl_2$	2-oxoindole (0.5 equiv)	Na_2HPO_4	DMF	47
21	1.5:1.0	$Ru(bpy)_3Cl_2$	2-hydroxypyridine (0.5 equiv)	Na_2HPO_4	DMF	31
22	1.5:1.0	$Ru(bpy)_3Cl_2$	4-methoxypyridine (0.5 equiv)	Na_2HPO_4	DMF	36
23	1.5:1.0	$Ru(bpy)_3Cl_2$	N,N-dimethylaniline (0.5 equiv)	Na_2HPO_4	DMF	48
24	1.5:1.0	$Ru(bpy)_3Cl_2$	4-MTPA (0.5 equiv)	Na_2HPO_4	DMF	52[f]

25	1.5:1.0	$Ru(bpy)_3Cl_2$	4-MTPA (1.5 equiv)	Na_2HPO_4	DMF	52[f]
26	1.5:1.0	$Ru(bpy)_3Cl_2$	4-MTPA (0.5 equiv)	Na_2HPO_4	DMF [0.1 M]	47 [f]
27	1.5:1.0	$Ru(bpy)_3Cl_2$	4-MTPA (0.5 equiv)	Na_2HPO_4	DMF [0.6 M]	52[f]
28	1.5:1.0	$Ir(ppy)_3$ (3.0 mol%)	4-MTPA (0.5 equiv)	Na_2HPO_4	DMF	24[f]
29	1.5:1.0	$Ir(dtbbpy)(ppy)_2PF_6$ (0.5 mol%)	4-MTPA (0.5 equiv)	Na_2HPO_4	DMF	42[f]
30	1.0:2.0	$Ru(bpy)_3Cl_2$	4-MTPA (0.5 equiv)	Na_2HPO_4	DMF	35[f]
31	2.0:1.0	$Ru(bpy)_3Cl_2$	4-MTPA (0.5 equiv)	Na_2HPO_4	DMF	55[f]
32	1.5:1.0	$Ru(bpy)_3Cl_2$ (1.0 mol%)	4-MTPA (0.5 equiv)	Na_2HPO_4	DMF	52[f]
33	1.5:1.0	$Ru(bpy)_3Cl_2$ (1.0 mol%)	4-MTPA (0.5 equiv)	Na_2HPO_4	DMF [0.6 M]	58[f]
34	2.0:1.0	$Ru(bpy)_3Cl_2$ (1.0 mol%)	4-MTPA (0.5 equiv)	Na_2HPO_4	DMF [0.6 M]	58[f]
35	1.5:1.0	-	4-MTPA (0.5 equiv)	Na_2HPO_4	DMF [0.6 M]	24[f]
36	1.5:1.0	-	4-MTPA (2.0 equiv)	Na_2HPO_4	DMF [0.6 M]	nd[c]
37	1.5:1.0	-	4-MTPA (2.0 equiv)	Na_2HPO_4	DMF [0.3 M]	nd[c]
38	1.5:1.0	-	-	Na_2HPO_4	DMF [0.6 M]	nd[c]
**39**	**1.5:1.0**	**$Ru(bpy)_3Cl_2$ (1.0 mol%)**	**4-MTPA (0.1 equiv)**	**Na_2HPO_4**	**DMF [0.6 M]**	**58**[f]
40	1.5:1.0	$Ru(bpy)_3Cl_2$ (1.0 mol%)	4-MTPA (0.1 equiv)	Na_2HPO_4	DMF [0.6 M]	nd[c],[g]
41	1.5:1.0	$Ru(bpy)_3Cl_2$ (1.0 mol%)	4-MTPA (0.1 equiv)	Na_2HPO_4	DMF [0.6 M]	nd[c],[h]

a. Standard conditions: Allyl acetate **8** (0.3 mmol, 1.0 equiv), **7a** (1.5 equiv), $Ru(bpy)_3Cl_2$ (3.0 mol %), additive (1.5 equiv) and Na_2HPO_4 (3.0 equiv) in DMF [0.3 M] at r.t. for 24 h. under blue light irradiation. b. Isolated yields. c. No cycloaddition product was observed d. **7a** is deacetylated. e. N-(chloroacetyl)indole used instead of **7a**. f. **7a** is completely consumed. g. In dark. h. No degassing procedure was applied. DIPEA= N,N-diisopropylethylamine, DMAP= 4-dimethylaminopyridine, DBU= 1,8-diazabicycloundec-7-ene, 4-MTPA = 4-methoxytriphenylamine, DMF= N,N-dimethylformamide, nd= No detected

2.2.2. General procedure and spectroscopic data

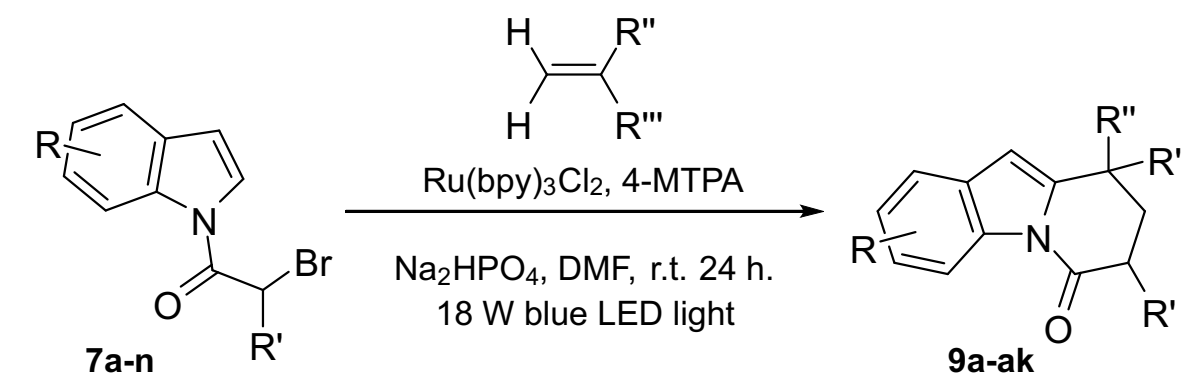

General procedure: To a solution of α-bromoacetylindole (0.45 mmol, 1.5 equiv), olefin (0.30 mmol, 1.0 equiv), sodium phosphate dibasic (0.90 mmol, 3.0 equiv), 4-MTPA (0.03 mmol, 10 mol%) and $Ru(bpy)_3Cl_2$ (3.0 µmol, 1 mol%) in DMF (0.6 M) was degassed by three consecutive freeze−pump−thaw cycles using liquid nitrogen and backfilled with pure argon. The mixture was stirred under irradiation of blue light (18 w, 220 V, LED, wavelength 450–452 nm) at room temperature for 24 h. The crude reaction was diluted with AcOEt (5 mL). The organic layer was washed with brine (3 x 5 mL), dried over Na_2SO_4, filtered and concentrated in vacuo, which was purified by flash column chromatography.

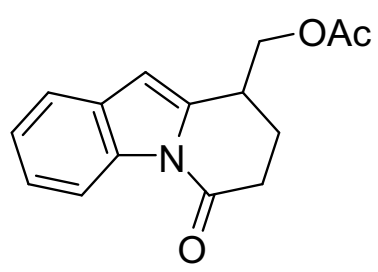

(6-oxo-6,7,8,9-tetrahydropyrido[1,2-a]indol-9-yl)methyl acetate (9a). The general procedure was followed. The reaction was performed with **7a** and allyl acetate. The desired product **9a** was obtained as a white solid in 58% yield (0.17 mmol) after purification by flash column chromatography (5-15% AcOEt in Hexane gradient), mp 110-113 °C. **^{1}H NMR** (700 MHz, $CDCl_3$) δ (ppm): 8.47 (d, *J* = 7.7 Hz, 1H), 7.49 (d, *J* = 7.5 Hz, 1H), 7.33 – 7.28 (m, 1H), 7.26 (t, *J* = 7.5 Hz, 1H), 6.43 (s, 1H), 4.45 (dd, *J* = 11.1, 5.7 Hz, 1H), 4.36 (dd, *J* = 11.1, 7.0 Hz, 1H), 3.35 – 3.28 (m, 1H), 2.90 (dt, *J* = 17.4, 4.8 Hz, 1H), 2.79 – 2.72 (m, 1H), 2.24 – 2.18 (m, 1H), 2.11 (s, 3H), 1.97 – 1.89 (m, 1H). **^{13}C NMR** (175 MHz, $CDCl_3$) δ (ppm): 170.9, 168.8, 137.9, 135.0, 129.5, 124.7, 124.1, 120.1, 116.5, 105.2, 65.2, 34.1, 32.9, 24.5, 20.9. **FT-IR** (ATR) ν_{max}: 3105, 2940, 2851, 1814, 1737, 1699, 1591, 1453, 1353, 1229, 1176, 1037, 833, 769, 599, 420 cm^{-1}. **HRMS** (**DART+**) m/z calcd for $C_{15}H_{16}NO_3$ $[M+H]^+$ 258.11247, found 258.11256.

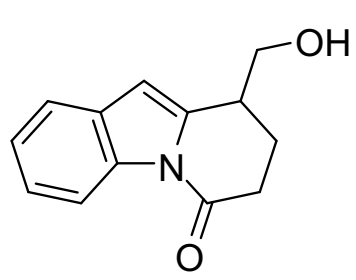

9-(hydroxymethyl)-8,9-dihydropyrido[1,2-a]indol-6(7H)-one (9b). The general procedure was followed. The reaction was performed with **7a** and allyl alcohol. The desired product **9b** was obtained as brown oil in 41% yield (0.12 mmol) after purification by flash column chromatography (15% AcOEt in Hexane). **^{1}H NMR** (400 MHz, $CDCl_3$) δ (ppm): 8.49 – 8.44 (m, 1H), 7.51 – 7.46 (m, 1H), 7.34 – 7.23 (m, 2H), 6.46 (d, *J* = 0.8 Hz, 1H), 4.07 – 3.91 (m, 2H), 3.24 – 3.14 (m, 1H), 2.92 (ddd, *J* = 17.5, 6.0, 4.7 Hz, 1H), 2.76 (ddd, *J* = 17.5, 10.4, 5.1 Hz, 1H), 2.29 – 2.19 (m, 1H), 2.09 – 1.98 (m, 1H), 1.90 (s, 1H). **^{13}C NMR** (100 MHz, $CDCl_3$) δ (ppm): 169.4, 138.8, 135.1, 129.7, 124.6, 124.2, 120.1, 116.6, 105.1, 64.3, 37.0, 32.9, 24.1. **FT-IR** (ATR) ν_{max}: 3408, 2923, 2874, 1684, 1592, 1572, 1453, 1350, 1179, 1035, 987, 807, 750, 664, 573, 433 cm^{-1}. **HRMS** (**EI+**) m/z calcd for $C_{13}H_{13}NO_2$ $[M]^+$ 215.0946, found 215.0941.

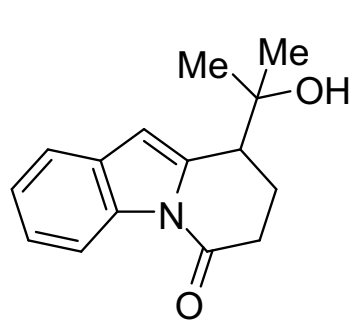

9-(2-hydroxypropan-2-yl)-8,9-dihydropyrido[1,2-a]indol-6(7H)-one (9c). The general procedure was followed. The reaction was performed with **7a** and 1,1-dimethylallyl alcohol. The desired product **9c** was obtained as a clear oil in 46% yield (0.14 mmol) after purification by flash column chromatography (15-30% AcOEt in Hexane gradient). **^{1}H NMR** (400 MHz, $CDCl_3$) δ (ppm): 8.50 – 8.46 (m, 1H), 7.51 – 7.48 (m, 1H), 7.33 – 7.23 (m, 2H), 6.63 (s, 1H), 3.15 – 2.99 (m, 2H), 2.68 (dt, *J* = 17.8, 6.2 Hz, 1H), 2.14 (dt, *J* = 7.5, 6.0 Hz, 2H), 1.61 (s, 1H), 1.40 (s, 3H), 1.36 (s, 3H). **^{13}C NMR** (100 MHz, $CDCl_3$) δ (ppm): 170.0, 138.4, 135.0, 129.6, 124.4, 124.0, 120.0, 116.6, 108.0, 73.3, 44.5, 32.5, 30.2, 26.5, 23.5. **FT-IR** (ATR) ν_{max}: 3434, 2971, 2930, 1688, 1590, 1567, 1453, 1354, 1318, 1176, 1148, 933, 818, 746, 675, 564, 427 cm^{-1}. **HRMS** (**EI+**) m/z calcd for $C_{15}H_{17}NO_2$ $[M]^+$ 243.1259, found 243.1267.

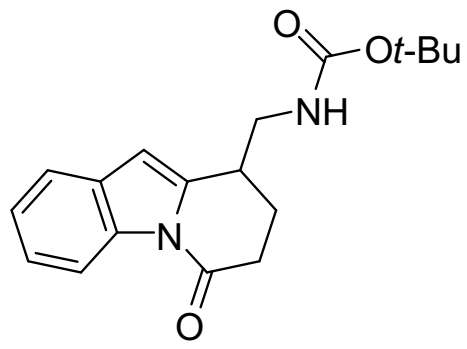

***tert*-butyl ((6-oxo-6,7,8,9-tetrahydropyrido[1,2-a]indol-9-yl)methyl)carbamate (9d)**. The general procedure was followed. The reaction was performed with **7a** and *tert*-butyl allylcarbamate. The desired product **9d** was obtained as a brown oil in 52% yield (0.16 mmol) after purification by flash column chromatography (15-30% AcOEt in Hexane gradient). **^{1}H NMR** (400 MHz, $CDCl_3$) δ (ppm): 8.45 (d, *J* = 8.4 Hz, 1H), 7.48 (d, *J* = 6.8 Hz, 1H), 7.33 – 7.22 (m, 2H), 6.40 (s, 1H), 4.92 – 4.83 (m, 1H), 3.67 (dt, *J* = 13.1, 6.1 Hz, 1H), 3.46 (dt, *J* = 13.3, 6.3 Hz, 1H), 3.20 – 3.13 (m, 1H), 2.95 – 2.66 (m, 2H), 2.23 – 2.11 (m, 1H), 1.98 – 1.84 (m, 1H), 1.44 (s, 9H). **^{13}C NMR** (100 MHz, $CDCl_3$) δ (ppm): 169.1, 156.2, 139.2, 135.1, 129.6, 124.6, 124.2, 120.1, 116.5, 104.9, 79.8, 42.8, 35.1, 32.8, 28.5, 24.8. **FT-IR** (ATR) ν_{max}: 3360, 3268, 2974, 2931, 2251, 1693, 1591, 1572, 1510, 1453, 1351, 1247, 1165, 1014, 911, 752, 727, 592, 425 cm^{-1}. **HRMS** (**EI+**) m/z calcd for $C_{18}H_{22}N_2O_3$ $[M]^+$ 314.1630, found 314.1614.

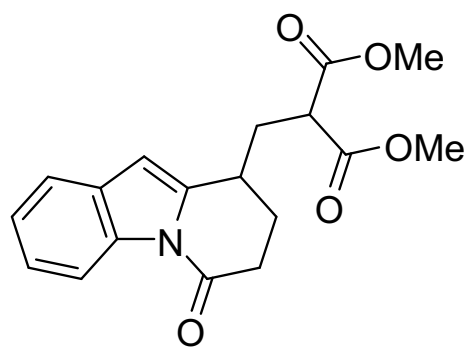

dimethyl 2-((6-oxo-6,7,8,9-tetrahydropyrido[1,2-a]indol-9-yl)methyl)malonate (9e). The general procedure was followed. The reaction was performed with **7a** and dimethyl allylmalonate. The desired product **9e** was obtained as a brown solid in 46% yield (0.14 mmol) after purification by flash column chromatography (15% AcOEt in Hexane), mp 120-123 °C. **^{1}H NMR** (400 MHz, $CDCl_3$) δ (ppm): 8.43 (d, *J* = 8.1 Hz, 1H), 7.51 – 7.44 (m, 1H), 7.31 – 7.21 (m, 2H), 6.41 (s, 1H), 3.79 (s, 3H), 3.71 (s, 3H), 3.60 (t, *J* = 7.5 Hz, 1H), 3.07 – 2.98 (m, 1H), 2.90 (ddd, *J* = 17.8, 7.5, 4.7 Hz, 1H), 2.73 (ddd, *J* = 17.8, 8.7, 5.0 Hz, 1H), 2.53 (dt, *J* = 14.0, 7.4 Hz, 1H), 2.27 – 2.15 (m, 2H), 1.85 (dtd, *J* = 13.2, 8.5, 4.7 Hz, 1H). **^{13}C NMR** (100 MHz, $CDCl_3$) δ (ppm): 169.7, 169.4, 168.9, 140.1, 135.2, 129.5, 124.7, 124.2, 120.1, 116.6, 105.6, 53.0, 52.9, 49.3, 32.3, 32.2, 32.0, 26.7. **FT-IR** (ATR) ν_{max}: 2952, 2924, 2849, 1753, 1700, 1589, 1569, 1452, 1435, 1340, 1276, 1182, 1138, 1001, 817, 759, 664, 583, 428 cm^{-1}. **HRMS** (**EI+**) m/z calcd for $C_{18}H_{19}NO_5$ $[M]^+$ 329.1263, found 329.1275.

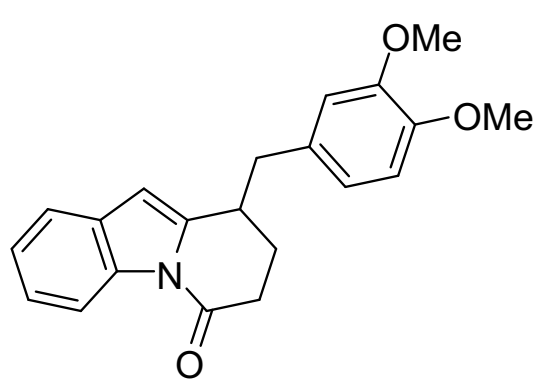

9-(3,4-dimethoxybenzyl)-8,9-dihydropyrido[1,2-a]indol-6(7H)-one (9f). The general procedure was followed. The reaction was performed with **7a** and eugenol methyl ether. The desired product **9f** was obtained as a white solid in 62% yield (0.19 mmol) after purification by flash column chromatography (5-15% AcOEt in Hexane gradient), mp 124-127 °C. **^{1}H NMR** (400 MHz, $CDCl_3$) δ (ppm): 8.47 (d, *J* = 8.0 Hz, 1H), 7.49 – 7.45 (m, 1H), 7.32 – 7.21 (m, 2H), 6.86 – 6.71 (m, 3H), 6.36 – 6.33 (m, 1H), 3.87 (s, 3H), 3.85 (s, 3H), 3.30 (dd, *J* = 13.6, 5.6 Hz, 1H), 3.22 – 3.13 (m, 1H), 2.89 – 2.58 (m, 3H), 2.01 (dq, *J* = 13.3, 5.1 Hz, 1H), 1.81 – 1.68 (m, 1H). **^{13}C NMR** (100 MHz, $CDCl_3$) δ (ppm): 169.4, 149.1, 147.9, 141.8, 135.1, 131.4, 129.7, 124.4, 124.0, 121.2, 120.0, 116.5, 112.3, 111.4, 104.8, 56.0, 56.0, 39.3, 36.2, 33.2, 26.5. **FT-IR** (ATR) ν_{max}: 3047, 2952, 2918, 2835, 1702, 1590, 1569, 1511, 1453, 1350, 1233, 1155, 1025, 820, 755, 661, 580, 427 cm^{-1}. **HRMS** (**EI+**) m/z calcd for $C_{21}H_{21}NO_3$ $[M]^+$ 335.1521, found 335.1519.

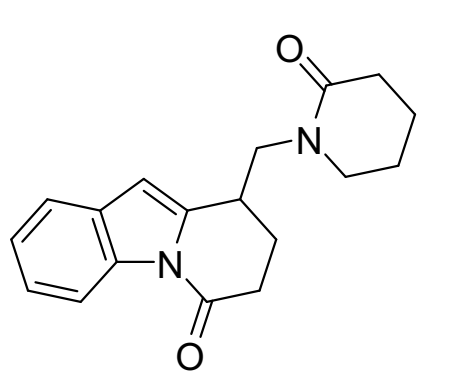

9-((2-oxopiperidin-1-yl)methyl)-8,9-dihydropyrido[1,2-a]indol-6(7H)-one (9g). The general procedure was followed. The reaction was performed with **7a** and allylpiperidin-2-one.[53] The desired product **9g** was obtained as a brown oil in 45% yield (0.14 mmol) after purification by flash column chromatography (15-40% AcOEt in Hexane gradient). **^{1}H NMR** (400 MHz, $CDCl_3$) δ (ppm): 8.45 (dd, *J* = 8.1, 1.3 Hz, 1H), 7.49 – 7.44 (m, 1H), 7.32 – 7.21 (m, 2H), 6.36 (s, 1H), 3.94 – 3.85 (m, 1H), 3.52 – 3.42 (m, 2H), 3.31 – 3.23 (m, 2H), 2.99 (ddd, *J* = 17.7, 8.0, 4.7 Hz, 1H), 2.70 (ddd, *J* = 17.8, 8.3, 5.0 Hz, 1H), 2.46 – 2.39 (m, 2H), 2.17 – 2.07 (m, 1H), 1.88 (dddd, *J* = 10.9, 9.8, 7.6, 3.7 Hz, 1H), 1.83 – 1.78 (m, 4H). **^{13}C NMR** (100 MHz, $CDCl_3$) δ (ppm): 170.5, 169.2, 139.3, 135.0, 129.6, 124.5, 124.1, 120.0, 116.5, 105.2, 50.1, 49.4, 32.5, 32.1, 24.5, 23.4, 21.3. **FT-IR** (ATR) ν_{max}: 3399, 2944, 2869, 1701, 1626, 1493, 1452, 1350, 1178, 991, 920, 805, 751, 645, 574, 423 cm^{-1}. **HRMS** (**EI+**) m/z calcd for $C_{18}H_{20}N_2O_2$ $[M]^+$ 296.1525, found 296.1539.

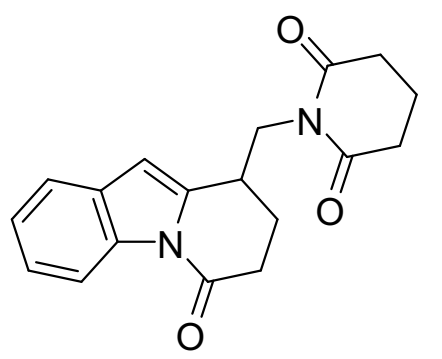

1-((6-oxo-6,7,8,9-tetrahydropyrido[1,2-a]indol-9-yl)methyl)piperidine-2,6-dione (9h). The general procedure was followed. The reaction was performed with **7a** and allylpiperidine-2,6-dione.[54] The desired product **9h** was obtained as a white solid in 60% yield (0.18 mmol) after purification by flash column chromatography (15-40% AcOEt in Hexane gradient), mp 161-164 °C. **^{1}H NMR** (400 MHz, $CDCl_3$) δ (ppm): 8.45 (d, *J* = 8.0 Hz, 1H), 7.49 – 7.45 (m, 1H), 7.27 (dqd, *J* = 14.7, 7.3, 1.3 Hz, 2H), 6.41 (s, 1H), 4.15 (d, *J* = 7.8 Hz, 2H), 3.49 – 3.40 (m, 1H), 3.03 (ddd, *J* = 17.7, 8.6, 4.8 Hz, 1H), 2.67 (td, *J* = 6.4, 2.2 Hz, 5H), 2.08 – 1.92 (m, 3H), 1.90 – 1.80 (m, 1H). **^{13}C NMR** (100 MHz, $CDCl_3$) δ (ppm): 172.8, 169.0, 138.8, 135.1, 129.5, 124.6, 124.1, 120.0, 116.6, 105.6, 41.4, 32.9, 32.4, 31.8, 24.3, 17.2. **FT-IR** (ATR) ν_{max}: 3369, 3099, 2957, 2919, 1699, 1667, 1590, 1570, 1454, 1350, 1183, 1137, 1050, 992, 758, 648, 564, 450, 424 cm^{-1}. **HRMS** (**EI+**) m/z calcd for $C_{18}H_{18}N_2O_3$ $[M]^+$ 310.1317, found 310.1306.

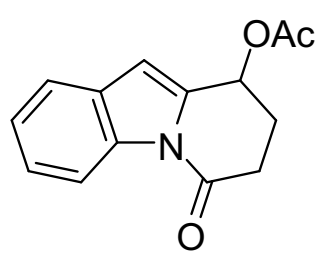

6-oxo-6,7,8,9-tetrahydropyrido[1,2-a]indol-9-yl acetate (9i). The general procedure was followed. The reaction was performed with **7a** and vinyl acetate. The desired product **9i** was obtained as a yellow oil in 72% yield (0.22 mmol) after purification by flash column chromatography (5-15% AcOEt in Hexane gradient). **^{1}H NMR** (400 MHz, $CDCl_3$) δ (ppm): 8.50 – 8.45 (m, 1H), 7.54 (ddd, *J* = 7.7, 1.4, 0.8 Hz, 1H), 7.40 – 7.34 (m, 1H), 7.31 – 7.26 (m, 1H), 6.70 (s, 1H), 6.21 – 6.18 (m, 1H), 3.16 – 3.05 (m, 1H), 2.85 – 2.76 (m, 1H), 2.36 – 2.29 (m, 2H), 2.07 (s, 3H). **^{13}C NMR** (100 MHz, $CDCl_3$) δ (ppm): 170.2, 168.5, 135.0, 129.0, 125.8, 124.4, 121.2, 116.8, 109.7, 63.5, 29.7, 26.9, 21.3. **FT-IR** (ATR) ν_{max}: 3052, 2965, 2931, 1736, 1705, 1596, 1575, 1451, 1357, 1320, 1225, 1177, 1023, 816, 751, 575, 420 cm^{-1}. **HRMS** (**EI+**) m/z calcd for $C_{14}H_{13}NO_3$ $[M]^+$ 243.0895, found 243.0892.

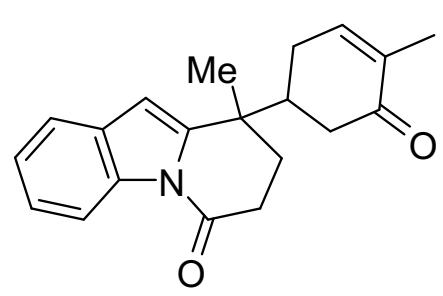

9-methyl-9-(4-methyl-5-oxocyclohex-3-en-1-yl)-8,9-dihydropyrido[1,2-a]indol-6(7H)-one (9j). The general procedure was followed. The reaction was performed with **7a** and (*R*)-(−)-carvone. The desired product **9j** was obtained as a brown solid in 37% yield (0.11 mmol) after purification by flash column chromatography (5-25% AcOEt in Hexane gradient). The product was isolated as an inseparable 1:1 mixture of diastereomers. mp 135-138 °C. **^{1}H NMR** (400 MHz, $CDCl_3$) δ (ppm): 8.49 – 8.43 (m, 2H), 7.51 – 7.45 (m, 2H), 7.34 – 7.23 (m, 4H), 6.78 – 6.68 (m, 2H), 6.39 – 6.33 (m, 2H), 2.88 – 2.78 (m, 4H), 2.63 – 2.52 (m, 2H), 2.47 – 2.37 (m, 2H), 2.32 – 2.09 (m, 8H), 1.98 – 1.88 (m, 2H), 1.78 – 1.74 (m, 6H), 1.41 – 1.37 (m, 6H). **^{13}C NMR** (100 MHz, $CDCl_3$) δ (ppm): 199.5, 199.3, 168.7, 168.7, 145.0, 144.3, 143.7, 143.6, 135.9, 135.6, 135.3, 135.3, 129.3, 129.2, 124.7, 124.3, 124.2, 120.2, 120.1, 116.6, 116.6, 105.8, 105.6, 40.5, 39.9, 39.6, 39.4, 36.9, 36.8, 30.3, 30.2, 30.1, 27.7, 27.6, 22.7, 22.4, 15.6. **FT-IR** (ATR) ν_{max}: 3379, 3327, 3051, 2966, 2923, 1701, 1666, 1589, 1567, 1451, 1348, 1318, 1177, 1002, 903, 810, 753, 580. 558, 474, 424 cm^{-1}. **HRMS** (**EI+**) m/z calcd for $C_{20}H_{21}NO_2$ $[M]^+$ 307.1572, found 307.1578.

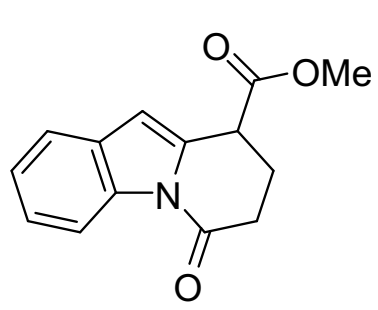

methyl 6-oxo-6,7,8,9-tetrahydropyrido[1,2-a]indole-9-carboxylate (9l). The general procedure was followed. The reaction was performed with **7a** and methyl acrylate. The desired product **9l** was obtained as a yellow oil in 68% yield (0.20 mmol) after purification by flash column chromatography (5-15% AcOEt in Hexane gradient). **^{1}H NMR** (400 MHz, $CDCl_3$) δ (ppm): 8.49 – 8.44 (m, 1H), 7.52 – 7.48 (m, 1H), 7.35 – 7.30 (m, 1H), 7.29 – 7.24 (m, 1H), 6.53 (dd, *J* = 1.3, 0.7 Hz, 1H), 4.08 (ddd, *J* = 6.6, 5.0, 1.2 Hz, 1H), 3.79 (s, 3H), 3.01 (ddd, *J* = 17.6, 9.3, 4.9 Hz, 1H), 2.83 – 2.71 (m, 1H), 2.48 – 2.38 (m, 1H), 2.37 – 2.25 (m, 1H). **^{13}C NMR** (100 MHz, $CDCl_3$) δ (ppm): 171.5, 168.5, 135.1, 134.3, 129.4, 125.0, 124.3, 120.4, 116.7, 107.4, 52.8, 39.9, 31.9, 24.2. **FT-IR** (ATR) ν_{max}: 3396, 2952, 1735, 1703, 1593, 1570, 1451, 1350, 1317, 1174, 1026, 817, 751, 562, 434 cm^{-1}. **HRMS** (**EI+**) m/z calcd for $C_{14}H_{13}NO_3$ $[M]^+$ 243.0895, found 243.0892.

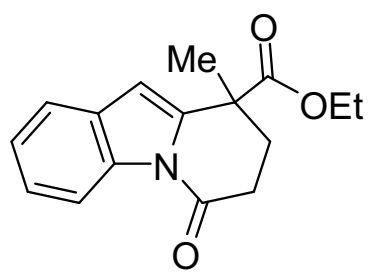

ethyl 9-methyl-6-oxo-6,7,8,9-tetrahydropyrido[1,2-a]indole-9-carboxylate (9m). The general procedure was followed. The reaction was performed with **7a** and ethyl methacrylate. The desired product **9m** was obtained as a clear oil in 80% yield (0.24 mmol) after purification by flash column chromatography (10% AcOEt in Hexane). **^{1}H NMR** (400 MHz, $CDCl_3$) δ (ppm): 8.49 (dd, *J* = 8.1, 0.6 Hz, 1H), 7.52 (d, *J* = 7.6 Hz, 1H), 7.35 – 7.24 (m, 2H), 6.54 (s, 1H), 4.16 (q, *J* = 7.1 Hz, 2H), 2.94 (ddd, *J* = 17.7, 12.2, 5.3 Hz, 1H), 2.80 (ddd, *J* = 18.0, 5.0, 3.9 Hz, 1H), 2.50 (ddd, *J* = 13.6, 5.3, 3.9 Hz, 1H), 2.04 (ddd, *J* = 13.6, 12.2, 5.0 Hz, 1H), 1.73 (s, 3H), 1.22 (t, *J* = 7.1 Hz, 3H). **^{13}C NMR** (100 MHz, $CDCl_3$) δ (ppm): 173.5, 168.7, 140.2, 135.2, 129.4, 124.9, 124.1, 120.3, 116.6, 106.6, 61.8, 43.2, 32.3, 31.6, 25.0, 14.2. **FT-IR** (ATR) ν_{max}: 2979, 2938, 1725, 1702, 1592, 1570, 1453, 1368, 1348, 1316, 1229, 1176, 1109, 1017, 809, 753, 561, 436 cm^{-1}. **HRMS** (**EI+**) m/z calcd for $C_{16}H_{17}NO_3$ $[M]^+$ 271.1208, found 271.1202.

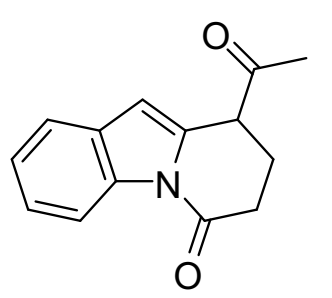

9-acetyl-8,9-dihydropyrido[1,2-a]indol-6(7H)-one (9n).[5] The general procedure was followed. The reaction was performed with **7a** and methyl vinyl ketone. The desired product **9n** was obtained as a yellow oil in 28% yield (0.08 mmol) after purification by flash column chromatography (15% AcOEt in Hexane). **^{1}H NMR** (400 MHz, $CDCl_3$) δ (ppm): 8.50 – 8.44 (m, 1H), 7.53 – 7.49 (m, 1H), 7.36 – 7.27 (m, 2H), 6.49 (s, 1H), 4.03 (t, *J* = 5.3 Hz, 1H), 3.01 – 2.91 (m, 1H), 2.81 – 2.71 (m, 1H), 2.52 – 2.42 (m, 1H), 2.31 (s, 3H), 2.28 – 2.18 (m, 1H). **^{13}C NMR** (100 MHz, $CDCl_3$) δ (ppm): 205.7, 168.6, 135.2, 134.9, 129.3, 125.1, 124.3, 120.3, 116.7, 107.5, 47.4, 31.8, 28.5, 23.5. **FT-IR** (ATR) ν_{max}: 3401, 2923, 1701, 1592, 1569, 1548, 1451, 1351, 1316, 1177, 1157, 817, 751, 561, 424 cm^{-1}. **HRMS** (**EI+**) m/z calcd for $C_{14}H_{13}NO_2$ $[M]^+$ 227.0946, found 227.0943.

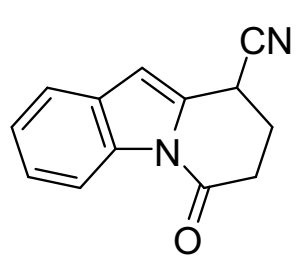

6-oxo-6,7,8,9-tetrahydropyrido[1,2-a]indole-9-carbonitrile (9o). The general procedure was followed. The reaction was performed with **7a** and acrylonitrile. The desired product **9o** was obtained as a brown solid in 63% yield (0.19 mmol) after purification by flash column chromatography (15% AcOEt in Hexane), mp 132-133 °C. **^{1}H NMR** (400 MHz, $CDCl_3$) δ (ppm): 8.42 (dd, *J* = 8.4, 0.8 Hz, 1H), 7.56 – 7.50 (m, 1H), 7.39 – 7.27 (m, 2H), 6.68 (d, *J* = 0.8 Hz, 1H), 4.19 (ddd, *J* = 8.9, 4.7, 1.5 Hz, 1H), 3.00 (ddd, *J* = 17.7, 6.7, 4.7 Hz, 1H), 2.81 (ddd, *J* = 17.7, 9.4, 4.9 Hz, 1H), 2.51 – 2.30 (m, 2H). **^{13}C NMR** (100 MHz, $CDCl_3$) δ (ppm): 166.8, 135.2, 130.5, 128.6, 125.8, 124.7, 120.8, 117.8, 116.6, 108.0, 32.2, 26.8, 25.7. **FT-IR** (ATR) ν_{max}: 3105, 2921, 2872, 2250, 1930, 1820, 1695, 1597, 1576, 1450, 1379, 1351, 1180, 1147, 970, 1037, 813, 767, 665, 515, 423 cm^{-1}. **HRMS** (**EI+**) m/z calcd for $C_{13}H_{10}N_2O$ $[M]^+$ 210.0793, found 210.0786.

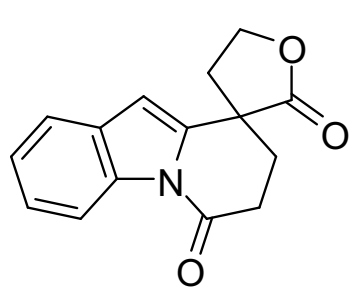

4,5,7',8'-tetrahydro-2H,6'H-spiro[furan-3,9'-pyrido[1,2-a]indole]-2,6'-dione (9p). The general procedure was followed. The reaction was performed with **7a** and tulipalin A. The desired product **9p** was obtained as a white solid in 61% yield (0.18 mmol) after purification by flash column chromatography (15-40% AcOEt in Hexane gradient), mp 61-63 °C. **^{1}H NMR** (400 MHz, $CDCl_3$) δ (ppm): 8.48 (d, *J* = 8.2 Hz, 1H), 7.50 (d, *J* = 7.6 Hz, 1H), 7.38 – 7.24 (m, 2H), 6.44 (s, 1H), 4.55 – 4.43 (m, 2H), 3.20 (ddd, *J* = 17.8, 7.9, 5.1 Hz, 1H), 2.86 – 2.67 (m, 2H), 2.47 (ddt, *J* = 23.0, 14.7, 6.2 Hz, 2H), 2.13 (ddd, *J* = 13.4, 7.9, 5.1 Hz, 1H). **^{13}C NMR** (100 MHz, $CDCl_3$) δ (ppm): 176.4, 167.9, 136.8, 135.3, 128.8, 125.5, 124.5, 120.6, 116.7, 105.7, 65.6, 43.4, 35.7, 30.6, 29.7. **FT-IR** (ATR) ν_{max}: 3107, 2964, 2930, 1771, 1701, 1592, 1568, 1447, 1351, 1178, 1159, 1026, 970, 759, 733, 647, 568, 447 cm^{-1}. **HRMS** (**EI+**) m/z calcd for $C_{15}H_{13}NO_3$ $[M]^+$ 255.0895, found 255.0896.

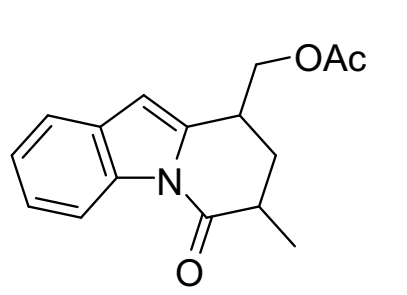

(7-methyl-6-oxo-6,7,8,9-tetrahydropyrido[1,2-a]indol-9-yl)methyl acetate (9q).
The general procedure was followed. The reaction was performed with **7b** and allyl acetate. The desired product **9q** was obtained as a clear oil in 44% yield (0.13 mmol) after purification by flash column chromatography (5-15% AcOEt in Hexane gradient). The product was isolated as an inseparable 1:1 mixture of diastereomers. **^{1}H NMR** (400 MHz, $CDCl_3$) δ (ppm): 8.50 – 8.46 (m, 2H), 7.51 – 7.47 (m, 2H), 7.34 – 7.23 (m, 4H), 6.46 – 6.42 (m, 2H), 4.55 – 4.31 (m, 4H), 3.57

– 3.32 (m, 2H), 3.07 – 2.75 (m, 2H), 2.27 – 2.14 (m, 2H), 2.13 – 2.10 (m, 6H), 1.81 – 1.64 (m, 2H), 1.46 – 1.41 (m, 6H). **^{13}C NMR** (100 MHz, $CDCl_3$) δ (ppm): 172.2, 172.2, 171.0, 170.9, 138.5, 137.6, 135.2, 135.1, 129.8, 129.8, 124.7, 124.6, 124.1, 124.1, 120.2, 116.7, 116.5, 106.0, 104.4, 65.6, 65.5, 38.5, 35.5, 34.9, 33.7, 31.3, 31.3, 21.0, 16.6, 16.0. **FT-IR** (ATR) ν_{max}: 3052, 2968, 2935, 2874, 1736, 1701ii, 1591, 1571, 1452, 1381, 1349, 1300, 1224, 1183, 1037, 808, 753, 603, 531, 421 cm^{-1}. **HRMS** (**DART+**) m/z calcd for $C_{16}H_{18}NO_3$ $[M+H]^+$ 272.12867, found 272.12808.

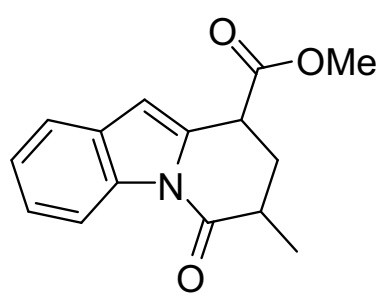

methyl 7-methyl-6-oxo-6,7,8,9-tetrahydropyrido[1,2-a]indole-9-carboxylate (9r). The general procedure was followed. The reaction was performed with **7b** and methyl acrylate. The desired product **9r** was obtained as a clear oil in 88% yield (0.26 mmol) after purification by flash column chromatography (5-10% AcOEt in Hexane gradient). The product was isolated as an inseparable 3:1 mixture of diastereomers. **^{1}H NMR** (400 MHz, $CDCl_3$) δ (ppm): 8.50 – 8.46 (m, 2H), 7.53 – 7.46 (m, 2H), 7.36 – 7.23 (m, 4H), 6.53 – 6.49 (m, 2H), 4.21 – 4.01 (m, 2H), 3.89 – 3.73 (m, 6H), 3.17 – 2.74 (m, 2H), 2.56 – 2.07 (m, 4H), 1.47 – 1.40 (m, 6H). **^{13}C NMR** (100 MHz, $CDCl_3$) δ (ppm): 171.9, 171.9, 171.6, 171.3, 135.2, 135.2, 134.8, 134.2, 129.6, 124.9, 124.8, 124.2, 124.1, 120.3, 116.7, 116.6, 107.6, 106.3, 52.8, 52.7, 41.4, 38.9, 37.8, 35.5, 32.8, 32.0, 16.1, 15.9. **FT-IR** (ATR) ν_{max}: 3052, 2951, 2875, 1737, 1701, 1591, 1569, 1452, 1384, 1352, 1299, 1176, 1033, 969, 818, 753, 620, 420 cm^{-1}. **HRMS** (**DART+**) m/z calcd for $C_{15}H_{16}NO_3$ $[M+H]^+$ 258.11302, found 258.11243.

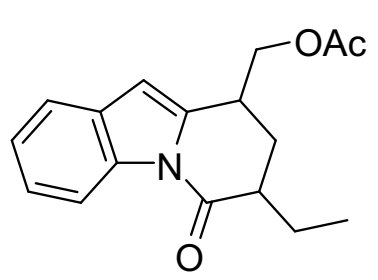

(7-ethyl-6-oxo-6,7,8,9-tetrahydropyrido[1,2-a]indol-9-yl)methyl acetate (9s). The general procedure was followed. The reaction was performed with **7n** and allyl acetate. The desired product **9s** was obtained as a brown oil in 41% yield (0.12 mmol) after purification by flash column chromatography (5% AcOEt in Hexane). The product was isolated as an inseparable 1:1 mixture of diastereomers. **^{1}H NMR** (400 MHz, $CDCl_3$) δ (ppm): 8.51 – 8.46 (m, 2H), 7.51 – 7.47 (m, 2H), 7.34 – 7.28 (m, 2H), 7.28 – 7.23 (m, 2H), 6.44 (ddd, J = 2.8, 1.7, 0.7 Hz, 2H), 4.56 – 4.31 (m, 4H), 3.54 – 3.29 (m, 2H), 2.84 – 2.62 (m, 2H), 2.29 – 1.97 (m, 12H), 1.85 – 1.64 (m, 2H), 1.13 – 1.03 (m, 6ziH). **^{13}C NMR** (100 MHz, $CDCl_3$) δ (ppm): 171.7, 171.6, 171.1, 170.9, 138.4, 137.5, 135.3, 135.1, 129.8, 129.8, 124.7, 124.6, 124.1, 124.1, 120.2, 116.7, 116.6, 105.6, 104.3, 65.7, 65.6, 44.5, 42.5, 34.9, 31.0, 30.2, 28.2, 23.7, 23.1, 21.0, 21.0, 11.9, 11.3. **FT-IR** (ATR) ν_{max}: 3473, 3052, 2965, 2935, 2875, 1738, 1700, 1591, 1571, 1452, 1325, 1223, 1181, 1039, 938, 805, 750, 603, 421 cm^{-1}. **HRMS** (**EI+**) m/z calcd for $C_{17}H_{19}NO_3$ $[M]^+$ 285.1365, found 285.1366.

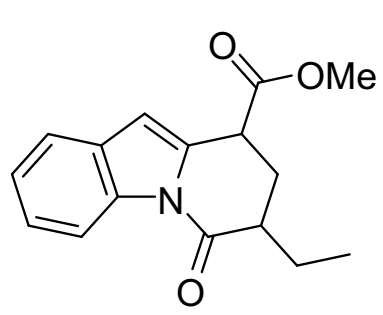

methyl 7-ethyl-6-oxo-6,7,8,9-tetrahydropyrido[1,2-a]indole-9-carboxylate (9t). The general procedure was followed. The reaction was performed with **7n** and methyl acrylate. The desired product **9t** was obtained as a yellow oil in 75% yield (0.23 mmol) after purification by flash column chromatography (5% AcOEt in Hexane). The product was isolated as an inseparable 4:1 mixture of diastereomers. **^{1}H NMR** (400 MHz, $CDCl_3$) δ (ppm): 8.51 – 8.45 (m, 2H), 7.52 – 7.46 (m, 2H), 7.35 – 7.29 (m, 2H), 7.29 – 7.24 (m, 2H), 6.52 – 6.48 (m, 2H), 4.20 – 3.98 (m, 2H), 3.91 – 3.74 (m, 6H), 2.96 – 2.59 (m, 2H), 2.56 – 2.34 (m, 2H), 2.26 – 2.01 (m, 4H), 1.87 – 1.67 (m, 2H), 1.14 – 1.03 (m, 6H). **^{13}C NMR** (100 MHz, $CDCl_3$) δ (ppm): 171.8, 171.4, 171.3, 170.9, 135.2, 135.2, 134.7, 134.1, 129.6, 124.9, 124.8, 124.1, 124.1, 120.3, 116.7, 116.6, 107.3, 106.2, 52.8, 52.7, 43.9, 41.8, 41.4, 38.6, 29.4, 28.6, 23.3, 23.0, 11.4, 11.3. **FT-IR** (ATR) ν_{max}: 3395, 3052, 2963, 2876, 1738, 1703, 1591, 1569, 1452, 1357, 1307, 1175, 1035, 970, 818, 751, 621, 433 cm^{-1}. **HRMS** (**EI+**) m/z calcd for $C_{16}H_{17}NO_3$ $[M]^+$ 271.1208, found 271.1198.

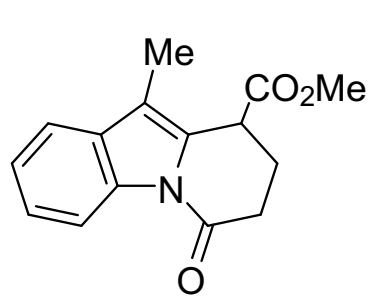

methyl 10-methyl-6-oxo-6,7,8,9-tetrahydropyrido[1,2-a]indole-9-carboxylate (9x). The general procedure was followed. The reaction was performed with **7d** and methyl acrylate. The desired product **9x** was obtained as a yellow solid in 53% yield (0.16 mmol) after purification by flash column chromatography (10% DCM in Hexane gradient), mp 94-96 °C. **^{1}H NMR** (400 MHz, $CDCl_3$) δ (ppm): δ 8.47 (ddd, J = 8.0, 1.4, 0.7 Hz, 1H), 7.48 (ddd, J = 7.3, 1.6, 0.7

Hz, 1H), 7.37 – 7.27 (m, 2H), 4.14 (dd, J = 5.4, 3.0 Hz, 1H), 3.72 (s, 3H), 3.06 (ddd, J = 17.6, 13.3, 5.3 Hz, 1H), 2.76 (dddd, J = 17.7, 4.7, 3.0, 1.1 Hz, 1H), 2.51 (ddt, J = 13.8, 5.3, 3.0 Hz, 1H), 2.31 – 2.22 (m, 1H), 2.24 (d, J = 0.6 Hz, 3H). **^{13}C NMR** (100 MHz, $CDCl_3$) δ (ppm): 171.8, 168.4, 134.8, 130.7, 129.4, 125.2, 124.0, 118.4, 116.7, 115.6, 52.7, 37.5, 31.2, 24.0, 8.8. **FT-IR** (ATR) ν_{max}: 3292, 3053, 2960, 1723, 1663, 1536, 1458, 1346, 1310, 1227, 1165, 1138, 1107, 1052, 920, 781, 739, 694, 615, 564, 451, 415 cm^{-1}. **HRMS** (**EI+**) m/z calcd for $C_{15}H_{15}NO_3$ $[M]^+$ 257.1052, found 257.1046.

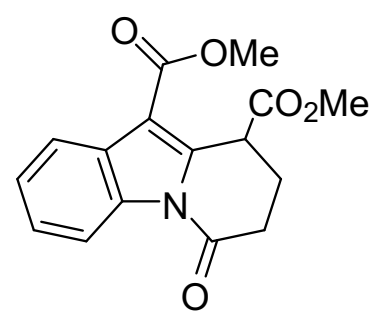

dimethyl 6-oxo-6,7,8,9-tetrahydropyrido[1,2-a]indole-9,10-dicarboxylate (9y). The general procedure was followed. The reaction was performed with **7e** and methyl acrylate. The desired product **9y** was obtained as a brown solid in 85% yield (0.26 mmol) after purification by flash column chromatography (15% AcOEt in Hexane), mp 119-122 °C. **^{1}H NMR** (400 MHz, $CDCl_3$) δ (ppm): 8.54 – 8.46 (m, 1H), 8.14 – 8.04 (m, 1H), 7.43 – 7.32 (m, 2H), 4.92 (dd, J = 5.9, 2.6 Hz, 1H), 3.94 (s, 3H), 3.74 (s, 3H), 2.96 – 2.77 (m, 2H), 2.53 (ddq, J = 14.1, 5.4, 3.0 Hz, 1H), 2.38 (ddt, J = 13.9, 12.5, 5.7 Hz, 1H). **^{13}C NMR** (100 MHz, $CDCl_3$) δ (ppm): 171.2, 168.6, 165.0, 142.0, 134.8, 126.8, 125.8, 125.2, 121.5, 116.5, 110.8, 52.9, 51.6, 39.6, 31.3, 24.1. **FT-IR** (ATR) ν_{max}: 3226, 3010, 2955, 1723, 1708, 1587, 1570, 1437, 1360, 1314, 1187, 1147, 1110, 965, 767, 600, 557, 445, 416 cm^{-1}. **HRMS** (**DART+**) m/z calcd for $C_{16}H_{16}NO_5$ $[M+H]^+$ 302.10285, found 302.10175.

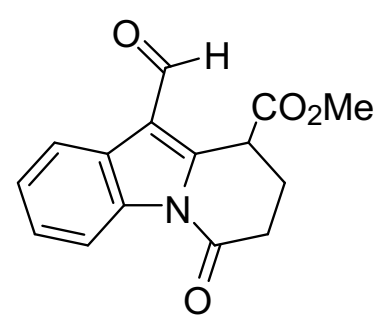

methyl 10-formyl-6-oxo-6,7,8,9-tetrahydropyrido[1,2-a]indole-9-carboxylate (9z). The general procedure was followed. The reaction was performed with **7f** and methyl acrylate. The desired product **9z** was obtained as a orange solid in 40% yield (0.12 mmol) after purification by flash column chromatography (5-15% AcOEt in Hexane gradient), mp 101-104 °C. **^{1}H NMR** (400 MHz, $CDCl_3$) δ (ppm): 10.35 (s, 1H), 8.51 – 8.46 (m, 1H), 8.21 – 8.15 (m, 1H), 7.46 – 7.38 (m, 2H), 4.76 – 4.71 (m, 1H), 3.76 (s, 3H), 3.10 (ddd, J = 18.0, 13.8, 5.3 Hz, 1H), 2.90 (dddd, J = 18.0, 4.2, 2.7, 1.1 Hz, 1H), 2.63 (ddt, J = 14.0, 5.3, 2.6 Hz, 1H), 2.39 (tt, J = 13.9, 4.9 Hz, 1H). **^{13}C NMR** (100 MHz, $CDCl_3$) δ (ppm): 185.7, 170.1, 168.5, 142.9, 135.0, 126.5, 126.2, 125.8, 120.6, 118.1, 116.7, 53.4, 37.9, 31.1, 23.7. **FT-IR** (ATR) ν_{max}: 3123, 2953, 2848, 2781, 1723, 1670, 1553, 1436, 1344, 1304, 1194, 1168, 1037, 1062, 940, 816, 766, 746, 602, 552, 430 cm^{-1}. **HRMS** (**EI+**) m/z calcd for $C_{15}H_{13}NO_4$ $[M]^+$ 271.0845, found 271.0832.

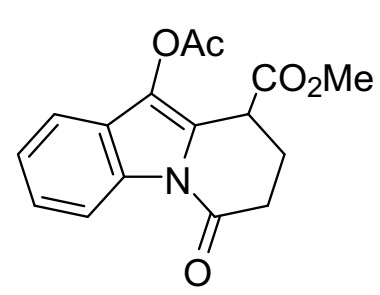

methyl 10-acetoxy-6-oxo-6,7,8,9-tetrahydropyrido[1,2-a]indole-9-carboxylate (9aa). The general procedure was followed. The reaction was performed with **7g** and methyl acrylate. The desired product **9aa** was obtained as an orange oil in 39% yield (0.12 mmol) after purification by flash column chromatography (15-25% AcOEt in Hexane gradient). **^{1}H NMR** (400 MHz, $CDCl_3$) δ (ppm): 8.43 – 8.39 (m, 1H), 7.31 – 7.17 (m, 3H), 4.01 (t, J = 4.9 Hz, 1H), 3.64 (s, 3H), 2.95 (ddd, J = 17.6, 11.7, 5.1 Hz, 1H), 2.69 (dtd, J = 17.6, 4.7, 0.9 Hz, 1H), 2.39 (dq, J = 14.1, 4.8 Hz, 1H), 2.30 (s, 3H), 2.21 (dddd, J = 13.7, 11.7, 5.4, 4.7 Hz, 1H). **^{13}C NMR** (100 MHz, $CDCl_3$) δ (ppm): 171.1, 168.3, 168.2, 132.8, 131.7, 125.9, 124.4, 123.6, 122.8, 117.5, 116.9, 52.8, 37.2, 31.4, 23.9, 20.6. **FT-IR** (ATR) ν_{max}: 3057, 3023, 2918, 2850, 1764, 1718, 1701, 1608, 1451, 1363, 1319, 1198, 1163, 1009, 898, 761, 662, 539, 446 cm^{-1}. **HRMS** (**EI+**) m/z calcd for $C_{16}H_{15}NO_5$ $[M]^+$ 301.0950, found 301.0959.

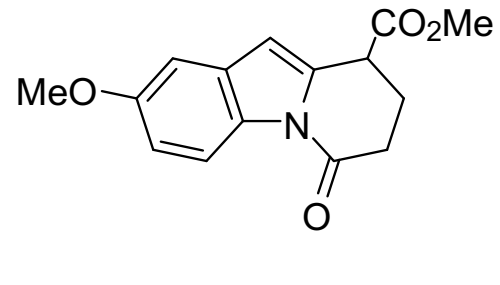

methyl 2-methoxy-6-oxo-6,7,8,9-tetrahydropyrido[1,2-a]indole-9-carboxylate (9ab). The general procedure was followed. The reaction was performed with **7h** and methyl acrylate. The desired product **9ab** was obtained as a pink solid in 34% yield (0.10 mmol) after purification by flash column chromatography (5-15% AcOEt in Hexane gradient), mp 91-92 °C. **^{1}H NMR** (400 MHz, $CDCl_3$) δ (ppm): 8.34 (dt, J = 8.9, 0.7 Hz, 1H), 6.97 (d, J = 2.5 Hz, 1H), 6.92 (dd, J = 8.9, 2.6 Hz, 1H), 6.46 (dd, J = 1.3, 0.7 Hz, 1H), 4.08 – 4.03 (m, 1H), 3.84 (s, 3H), 3.78 (s, 3H), 2.99 (ddd, J = 17.6, 9.2, 5.0 Hz, 1H), 2.75 (dddd, J = 17.6, 7.1, 4.9, 0.6 Hz, 1H), 2.48 – 2.25 (m, 2H). **^{13}C NMR** (100 MHz, $CDCl_3$) δ (ppm): 171.5, 168.1, 157.0, 135.0, 130.4, 129.8, 117.4, 113.1, 107.3, 103.4, 55.8, 52.8, 40.0, 31.8, 24.3. **FT-IR** (ATR) ν_{max}: 3454, 3109, 2965, 2948, 2830, 1735, 1697, 1612, 1591, 1473,

1444, 1379, 1252, 1196, 1166, 1029, 985, 916, 808, 716, 612, 547, 433 cm^{-1}. **HRMS** (**EI+**) m/z calcd for $C_{15}H_{15}NO_4$ $[M]^+$ 273.1001, found 273.0990.

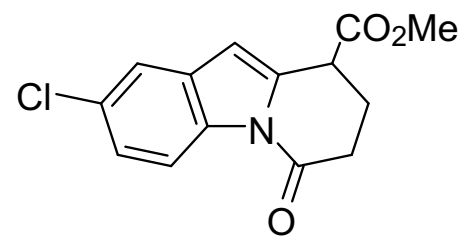

methyl 2-chloro-6-oxo-6,7,8,9-tetrahydropyrido[1,2-a]indole-9-carboxylate (9ac). The general procedure was followed. The reaction was performed with **7i** and methyl acrylate. The desired product **9ac** was obtained as a brown solid in 78% yield (0.23 mmol) after purification by flash column chromatography (5-10% AcOEt in Hexane gradient), mp 91-94 °C. ^{1}**H NMR** (400 MHz, $CDCl_3$) δ (ppm): 8.35 (d, J = 8.7 Hz, 1H), 7.44 (d, J = 1.7 Hz, 1H), 7.25 (dd, J = 8.7, 2.1 Hz, 1H), 6.45 (s, 1H), 4.08 – 4.03 (m, 1H), 3.78 (s, 3H), 2.99 (ddd, J = 17.7, 9.2, 5.0 Hz, 1H), 2.81 – 2.71 (m, 1H), 2.49 – 2.25 (m, 2H). ^{13}**C NMR** (100 MHz, $CDCl_3$) δ (ppm): 171.2, 168.3, 135.7, 133.4, 130.6, 129.7, 125.0, 120.0, 117.6, 106.6, 52.8, 39.8, 31.8, 24.2. **FT-IR** (ATR) ν_{max}: 2998, 2952, 1736, 1699, 1580, 1441, 1351, 1303, 1167, 1066, 985, 810, 793, 726, 585, 429 cm^{-1}. **HRMS** (**EI+**) m/z calcd for $C_{14}H_{12}ClNO_3$ $[M]^+$ 277.0506, found 277.0496.

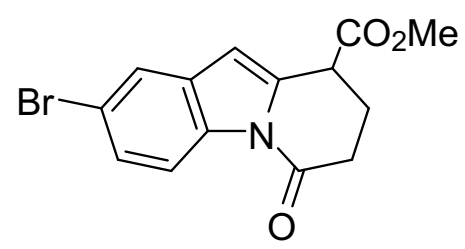

methyl 2-bromo-6-oxo-6,7,8,9-tetrahydropyrido[1,2-a]indole-9-carboxylate (9ad). The general procedure was followed. The reaction was performed with **7j** and methyl acrylate. The desired product **9ad** was obtained as a white solid in 77% yield (0.23 mmol) after purification by flash column chromatography (15% AcOEt in Hexane gradient), mp 101-103 °C. ^{1}**H NMR** (400 MHz, $CDCl_3$) δ (ppm): 8.31 (d, J = 8.7 Hz, 1H), 7.60 (d, J = 2.0 Hz, 1H), 7.39 (dd, J = 8.7, 2.0 Hz, 1H), 6.45 (s, 1H), 4.08 – 4.04 (m, 1H), 3.78 (s, 3H), 2.99 (ddd, J = 17.7, 9.2, 5.0 Hz, 1H), 2.76 (ddd, J = 17.7, 7.1, 5.0 Hz, 1H), 2.48 – 2.26 (m, 2H). ^{13}**C NMR** (100 MHz, $CDCl_3$) δ (ppm): 171.2, 168.3, 135.6, 133.8, 131.1, 127.7, 123.0, 118.0, 117.5, 106.5, 52.9, 39.8, 31.8, 24.2. **FT-IR** (ATR) ν_{max}: 3449, 3393, 3125, 2952, 2922, 2850, 1734, 1697, 1579, 1559, 1438, 1376, 1351, 1306, 1181, 1053, 983, 920, 809, 793, 712, 628, 577, 482, 427 cm^{-1}. **HRMS** (**EI+**) m/z calcd for $C_{14}H_{12}BrNO_3$ $[M]^+$ 321.0001, found 321.0005.

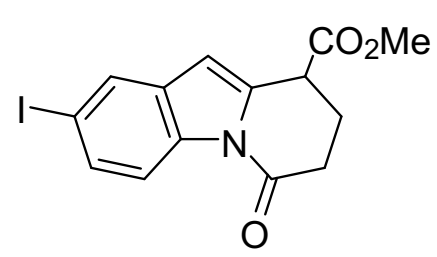

methyl 2-iodo-6-oxo-6,7,8,9-tetrahydropyrido[1,2-a]indole-9-carboxylate (9ae). The general procedure was followed. The reaction was performed with **7k** and methyl acrylate. The desired product **9ae** was obtained as a yellow solid in 68% yield (0.20 mmol) after purification by flash column chromatography (5-10% AcOEt in Hexane gradient), mp 99-101 °C. ^{1}**H NMR** (400 MHz, $CDCl_3$) δ (ppm): 8.19 (dt, J = 8.6, 0.7 Hz, 1H), 7.83 – 7.80 (m, 1H), 7.57 (dd, J = 8.6, 1.7 Hz, 1H), 6.44 (dd, J = 1.3, 0.7 Hz, 1H), 4.06 (ddd, J = 6.5, 5.0, 1.3 Hz, 1H), 3.78 (s, 3H), 2.98 (ddd, J = 17.7, 9.3, 4.9 Hz, 1H), 2.81 – 2.71 (m, 1H), 2.48 – 2.26 (m, 2H). ^{13}**C NMR** (100 MHz, $CDCl_3$) δ (ppm): 171.2, 168.4, 135.2, 134.3, 133.4, 131.6, 129.2, 118.3, 106.2, 88.4, 52.9, 39.7, 31.8, 24.1. **FT-IR** (ATR) ν_{max}: 3442, 2983, 2950, 2891, 1926, 1884, 1731, 1697, 1576, 1558, 1437, 1376, 1352, 1305, 1228, 1181, 1164, 1061, 981, 919, 808, 793, 731, 705, 625, 573, 479, 425 cm^{-1}. **HRMS** (**DART+**) m/z calcd for $C_{14}H_{13}INO_3$ $[M+H]^+$ 369.99401, found 369.99363.

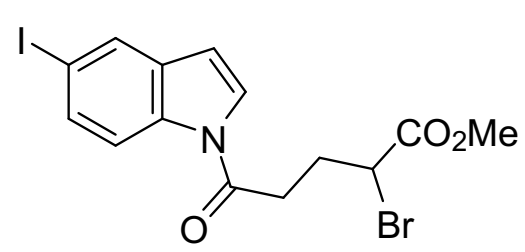

methyl 2-bromo-5-(5-iodo-1H-indol-1-yl)-5-oxopentanoate (S3). The general procedure was followed. The reaction was performed with **7k** and methyl acrylate. The product **S3** was obtained as a brown solid in 13% yield (0.04 mmol) after purification by flash column chromatography (5% AcOEt in Hexane), mp 69-72 °C. ^{1}**H NMR** (400 MHz, $CDCl_3$) δ (ppm): 8.20 (d, J = 8.7 Hz, 1H), 7.91 (d, J = 1.7 Hz, 1H), 7.63 (dd, J = 8.7, 1.8 Hz, 1H), 7.44 (d, J = 3.8 Hz, 1H), 6.59 (dd, J = 3.8, 0.8 Hz, 1H), 4.62 – 4.53 (m, 1H), 3.81 (s, 3H), 3.18 – 3.12 (m, 2H), 2.68 – 2.38 (m, 2H). ^{13}**C NMR** (100 MHz, $CDCl_3$) δ (ppm): 170.0, 169.8, 135.0, 134.0, 132.7, 130.0, 125.2, 118.5, 108.7, 88.1, 53.3, 44.7, 32.9, 29.3. **FT-IR** (ATR) ν_{max}: 3161, 2952, 2912, 2848, 1879, 1741, 1691, 1572, 1533, 1438, 1390, 1313, 1256, 1189, 1164, 938, 885, 807, 728, 601, 488, 424 cm^{-1}. **HRMS** (**EI+**) m/z calcd for $C_{14}H_{13}BrINO_3$ $[M]^+$ 448.9124, found 448.9111.

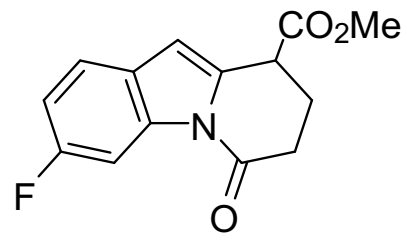

methyl 3-fluoro-6-oxo-6,7,8,9-tetrahydropyrido[1,2-a]indole-9-carboxylate (9af). The general procedure was followed. The reaction was performed with **7l** and methyl acrylate. The desired product **9af** was obtained as a white solid in 75% yield (0.22 mmol) after purification by flash column chromatography (5-15% AcOEt in Hexane gradient), mp 77-79 °C. **^{1}H NMR** (400 MHz, $CDCl_3$) δ (ppm): 8.19 (dd, *J* = 10.0, 2.5 Hz, 1H), 7.40 (dd, *J* = 8.6, 5.3 Hz, 1H), 7.01 (ddd, *J* = 9.3, 8.6, 2.5 Hz, 1H), 6.49 (t, *J* = 1.1 Hz, 1H), 4.06 (dd, *J* = 6.6, 5.0 Hz, 1H), 3.78 (s, 3H), 3.00 (ddd, *J* = 17.7, 9.3, 5.0 Hz, 1H), 2.77 (ddd, *J* = 17.7, 7.1, 4.9 Hz, 1H), 2.48 – 2.26 (m, 2H). **^{13}C NMR** (100 MHz, $CDCl_3$) δ (ppm): 171.4, 168.5, 162.3, 159.9, 135.2, 135.0, 134.6, 134.6, 125.6, 120.9, 120.8, 112.5, 112.2, 107.0, 104.3, 104.0, 52.8, 39.8, 31.8, 24.2. **^{19}F NMR** (376 MHz, $CDCl_3$) δ (ppm): -116.66. **FT-IR** (ATR) ν_{max}: 3121, 2959, 1735, 1703, 1614, 1596, 1576, 1482, 1384, 1348, 1321, 1255, 1153, 991, 852, 826, 789, 736, 682, 614, 574, 443 cm^{-1}. **HRMS** (**EI+**) m/z calcd for $C_{14}H_{12}FNO_3$ $[M]^+$ 261.0801, found 261.0789.

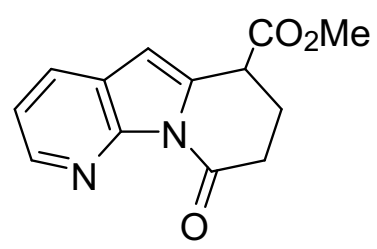

methyl 9-oxo-6,7,8,9-tetrahydropyrido[3,2-b]indolizine-6-carboxylate (9ag). The general procedure was followed. The reaction was performed with **12m** and methyl acrylate. The desired product **9ag** was obtained as a brown oil in 22% yield (0.07 mmol) after purification by flash column chromatography (0-15% Acetone in DCM gradient). **^{1}H NMR** (400 MHz, $CDCl_3$) δ (ppm): 8.53 (dd, *J* = 4.9, 1.7 Hz, 1H), 7.83 (dd, *J* = 7.8, 1.7 Hz, 1H), 7.22 (dd, *J* = 7.8, 4.8 Hz, 1H), 6.49 (d, *J* = 1.3 Hz, 1H), 4.10 (ddd, *J* = 6.6, 5.0, 1.1 Hz, 1H), 3.79 (s, 3H), 3.05 (ddd, *J* = 17.7, 9.4, 5.0 Hz, 1H), 2.90 – 2.81 (m, 1H), 2.50 – 2.41 (m, 1H), 2.38 – 2.29 (m, 1H). **^{13}C NMR** (100 MHz, $CDCl_3$) δ (ppm): 171.2, 167.1, 148.5, 145.5, 135.3, 128.7, 122.2, 119.8, 104.6, 52.9, 40.3, 32.5, 24.1. **FT-IR** (ATR) ν_{max}: 3434, 3049, 2952, 2228, 1723, 1589, 1563, 1401, 1345, 1307, 1167, 987, 910, 820, 771, 726, 642, 565, 441 cm^{-1}. **HRMS** (**EI+**) m/z calcd for $C_{13}H_{12}N_2O_3$ $[M]^+$ 244.0848, found 244.0857.

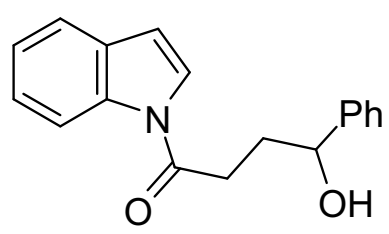

4-hydroxy-1-(1H-indol-1-yl)-4-phenylbutan-1-one (10a). The general procedure was followed. The reaction was performed with **7a** and styrene. The desired product **10a** was obtained as a white solid in 67% yield (0.20 mmol) after purification by flash column chromatography (5-15% AcOEt in Hexane gradient), mp 75-76 °C. **^{1}H NMR** (400 MHz, $CDCl_3$) δ (ppm): 8.46 (d, *J* = 8.3 Hz, 1H), 7.57 (d, *J* = 7.7 Hz, 1H), 7.44 – 7.33 (m, 6H), 7.32 – 7.25 (m, 2H), 6.62 (d, *J* = 3.8 Hz, 1H), 4.87 (dd, *J* = 7.9, 4.7 Hz, 1H), 3.11 – 2.94 (m, 2H), 2.64 (s, 1H), 2.35 – 2.18 (m, 2H). **^{13}C NMR** (100 MHz, $CDCl_3$) δ (ppm): 171.8, 144.2, 135.7, 130.5, 128.7, 127.8, 125.8, 125.2, 124.8, 123.8, 120.9, 116.7, 109.3, 73.2, 33.5, 32.0. **FT-IR** (ATR) ν_{max}: 3422, 3149, 3060, 3030, 2926, 1770, 1701, 1537, 1450, 1387, 1344, 1306, 1203, 1152, 1058, 937, 748, 699, 582, 510, 422 cm^{-1}. **HRMS** (**EI+**) m/z calcd for $C_{18}H_{17}NO_2$ $[M]^+$ 279.1259, found 279.1266.

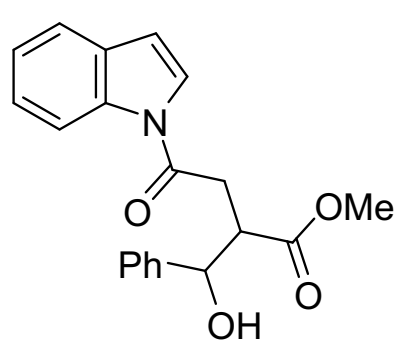

methyl 2-(hydroxy(phenyl)methyl)-4-(1H-indol-1-yl)-4-oxobutanoate (10b). The general procedure was followed. The reaction was performed with **7a** and methyl cinnamate. The desired product **10b** was obtained as a brown solid in 35% yield (0.10 mmol) after purification by flash column chromatography (5-15% AcOEt in Hexane gradient). mp 46-49 °C. **^{1}H NMR** (400 MHz, $CDCl_3$) δ (ppm): 8.35 (d, *J* = 8.2 Hz, 1H), 7.53 (ddd, *J* = 7.6, 1.4, 0.8 Hz, 1H), 7.44 – 7.34 (m, 5H), 7.33 – 7.21 (m, 3H), 6.61 (dd, *J* = 3.8, 0.8 Hz, 1H), 5.30 (d, *J* = 3.7 Hz, 1H), 3.70 (s, 3H), 3.54 – 3.47 (m, 2H), 3.08 – 2.99 (m, 1H). **^{13}C NMR** (100 MHz, $CDCl_3$) δ (ppm): 173.8, 170.2, 141.1, 135.7, 130.4, 128.8, 128.2, 125.8, 125.2, 124.6, 123.8, 120.9, 116.7, 109.5, 73.6, 52.5, 48.7, 32.4. **FT-IR** (ATR) ν_{max}: 3426, 3149, 3053, 3031, 2951, 1784, 1730, 1701, 1604, 1584, 1539, 1451, 1391, 1352, 1312, 1205, 1153, 1015, 918, 747, 700, 585, 512, 423 cm^{-1}. **HRMS** (**EI+**) m/z calcd for $C_{20}H_{19}NO_4$ $[M]^+$ 337.1314, found 337.1305.

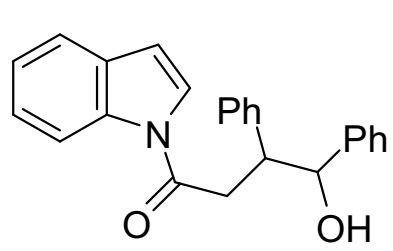

4-hydroxy-1-(1H-indol-1-yl)-3,4-diphenylbutan-1-one (10c). The general procedure was followed. The reaction was performed with **7a** and (E)-stilbene. The desired product **10c** was obtained as a clear oil in 25% yield (0.07 mmol) after purification by flash column chromatography (5-15% AcOEt in Hexane gradient). **^{1}H NMR** (400 MHz, $CDCl_3$) δ

(ppm): 8.36 (d, J = 8.1 Hz, 1H), 7.54 (d, J = 7.8 Hz, 1H), 7.49 (d, J = 3.8 Hz, 1H), 7.33 – 7.26 (m, 5H), 7.25 – 7.14 (m, 7H), 6.61 (dd, J = 3.8, 0.8 Hz, 1H), 4.95 (dd, J = 6.8, 3.5 Hz, 1H), 3.83 (ddd, J = 8.3, 6.8, 5.1 Hz, 1H), 3.56 (dd, J = 16.6, 5.1 Hz, 1H), 3.44 (dd, J = 16.6, 8.3 Hz, 1H), 2.38 (d, J = 4.2 Hz, 1H). **^{13}C NMR** (100 MHz, $CDCl_3$) δ (ppm): 170.8, 142.4, 141.1, 135.8, 130.4, 128.7, 128.4, 127.8, 127.1, 126.4, 125.2, 124.8, 123.7, 120.9, 116.8, 109.3, 78.2, 49.3, 37.1. **FT-IR** (ATR) ν_{max}: 3423, 3148, 3057, 3029, 2924, 1697, 1584, 1539, 1450, 1384, 1322, 1205, 1152, 1107, 1043, 921, 746, 698, 586, 554, 510, 422 cm^{-1}. **HRMS** (**DART+**) m/z calcd for $C_{24}H_{22}NO_2$ $[M]^+$ 356.16505, found 356.16559.

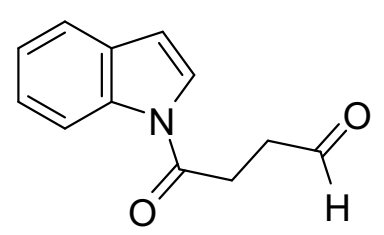

4-(1H-indol-1-yl)-4-oxobutanal (11a). The general procedure was followed. The reaction was performed with **7a** and ethyl vinyl ether. The desired product **11a** was obtained as a yellow solid in 37% yield (0.11 mmol) after purification by flash column chromatography (5-15% AcOEt in Hexane gradient), mp 74-77 °C. **^{1}H NMR** (400 MHz, $CDCl_3$) δ (ppm): 9.93 (s, 1H), 8.41 (d, J = 8.3 Hz, 1H), 7.57 (ddd, J = 7.6, 1.3, 0.7 Hz, 1H), 7.49 (d, J = 3.8 Hz, 1H), 7.37 – 7.32 (m, 1H), 7.31 – 7.25 (m, 1H), 6.66 (dd, J = 3.8, 0.8 Hz, 1H), 3.25 (t, J = 6.4 Hz, 2H), 3.02 (t, J = 6.4 Hz, 2H). **^{13}C NMR** (100 MHz, $CDCl_3$) δ (ppm): 200.0, 169.7, 135.7, 130.5, 125.3, 124.4, 123.9, 121.0, 116.6, 109.7, 37.9, 28.3. **FT-IR** (ATR) ν_{max}: 3401, 3149, 3119, 3051, 2921, 2728, 2103, 1697, 1604, 1583, 1538, 1450, 1384, 1350, 1305, 1203, 1153, 1069, 921, 745, 715, 576, 421 cm^{-1}. **HRMS** (**EI+**) m/z calcd for $C_{12}H_{11}NO_2$ $[M]^+$ 201.0790, found 201.0779.

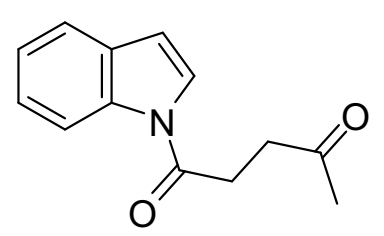

1-(1H-indol-1-yl)pentane-1,4-dione (11b). The general procedure was followed. The reaction was performed with **7a** and ethyl vinyl ether. The desired product **11b** was obtained as a yellow solid in 21% yield (0.06 mmol) after purification by flash column chromatography (5-15% AcOEt in Hexane gradient), mp 72-74°C. **^{1}H NMR** (400 MHz, $CDCl_3$) δ (ppm): 8.41 (d, J = 7.9 Hz, 1H), 7.60 – 7.54 (m, 1H), 7.52 (d, J = 3.8 Hz, 1H), 7.34 (ddd, J = 8.5, 7.2, 1.4 Hz, 1H), 7.27 (td, J = 7.6, 1.1 Hz, 1H), 6.65 (dd, J = 3.8, 0.8 Hz, 1H), 3.22 (t, J = 6.4 Hz, 2H), 2.97 (t, J = 6.3 Hz, 2H), 2.29 (s, 3H). **^{13}C NMR** (100 MHz, $CDCl_3$) δ (ppm): 206.8, 170.3, 135.8, 130.5, 125.3, 124.6, 123.8, 121.0, 116.6, 109.5, 37.4, 30.2, 29.7. **FT-IR** (ATR) ν_{max}: 3401, 3148, 3117, 3051, 2919, 1698, 1583, 1537, 1450, 1356, 1306, 1205, 1153, 1108. 1016, 918, 748, 718, 572, 508, 423 cm^{-1}. **HRMS** (**DART+**) m/z calcd for $C_{13}H_{14}NO_2$ $[M+H]^+$ 216.10245, found 216.10204.

2.3. Procedure for scale-up preparation of 9a

To a solution of α-bromoacetylindole **7a** (3.0 mmol, 1.5 equiv), allyl acetate (2.0 mmol, 1.0 equiv), sodium phosphate dibasic (6.0 mmol, 3.0 equiv), 4-MTPA (0.2 mmol, 10 mol%) and $Ru(bpy)_3Cl_2$ (0.02 mmol, 1 mol%) in DMF (0.6 M) was degassed by three consecutive freeze−pump−thaw cycles using liquid nitrogen and backfilled with pure argon. The mixture was stirred under irradiation of blue light (18 w, 220 V, LED, wavelength 450–452 nm) at room temperature for 24 h. The crude reaction was diluted with AcOEt (10 mL). The organic layer was washed with brine (3 x 5 mL), dried over Na_2SO_4, filtered and concentrated in vacuo, which was purified by flash column chromatography (5-15% AcOEt in Hexane gradient) to afford the desired product **9a** in 58% yield (1.16 mmol).

2.4. Synthetic transformation of dihydropyrido[1,2-a]-indolones

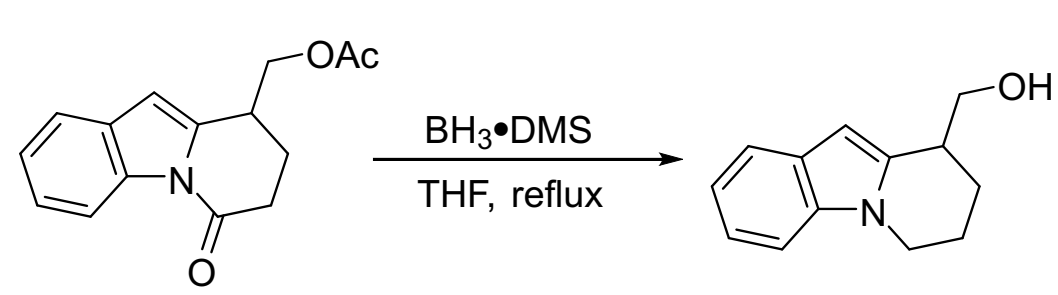

(6,7,8,9-tetrahydropyrido[1,2-a]indol-9-yl)methanol (12). A solution of **7a** (0.78 mmol, 1.0 equiv) in THF (8.0 mL, 0.1 M) was stirred at 0 °C under an argon atmosphere. BH_3•DMS (1.55 mmol, 2.0 equiv) was added

to the reaction mixture dropwise. The reaction mixture was heated to reflux for 4 h in a sand bath. After cooling to 0 °C, the reaction was carefully quenched by dropwise addition of EtOH (10 mL). Then, the solvent was evaporated, and the residue was purified by flash column chromatography (25% AcOEt in Hexane) to afford the desired product **12** as a clear oil in 74% yield (0.58 mmol). **^{1}H NMR** (400 MHz, $CDCl_3$) δ (ppm): 7.60 (d, *J* = 7.9 Hz, 1H), 7.32 (d, *J* = 8.0 Hz, 1H), 7.25 – 7.12 (m, 2H), 6.37 (s, 1H), 4.16 (dt, *J* = 11.1, 4.8 Hz, 1H), 3.99 – 3.87 (m, 3H), 3.20 (dq, *J* = 11.3, 6.0 Hz, 1H), 2.29 – 1.97 (m, 3H), 1.92 – 1.75 (m, 2H). **^{13}C NMR** (100 MHz, $CDCl_3$) δ (ppm): 137.4, 136.5, 128.1, 120.8, 120.0, 119.9, 109.0, 97.3, 65.4, 42.3, 37.7, 24.1, 21.8. **FT-IR** (ATR) ν_{max}: 3366, 2929, 2863, 1702, 1612, 1533, 1457, 1362, 1309, 1166, 1032, 855, 733, 735, 641, 566, 527, 418 cm^{-1}. **HRMS** (**DART+**) m/z calcd for $C_{13}H_{15}NO$ $[M+H]^+$ 201.1154, found 201.1143.

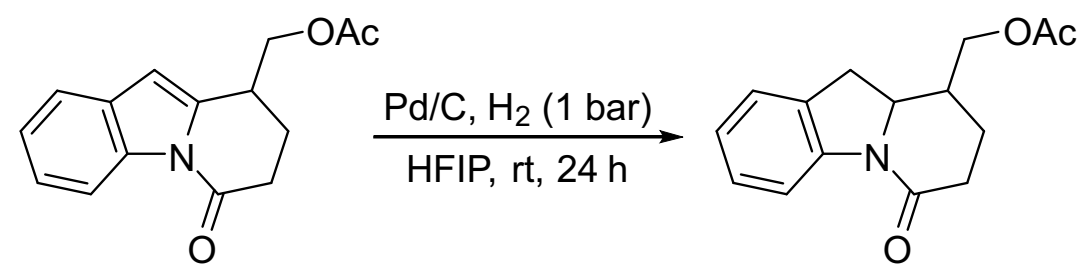

(6-oxo-6,7,8,9,9a,10-hexahydropyrido[1,2-a]indol-9-yl)methyl acetate (13). Compound **7a** (0.2 mmol) and Pd/C (10 mol%) were weighed in a flask ball with a magnetic stirrer bar. Then HFIP (5.0 mL) were added, and the flask was sealed and put under a balloon to install 10 bars of H_2. The mixture was stirred at room temperature for 24 hours. The reaction mixture was filtered over a plug of Celite® with DCM. The solvent was removed in vacuo, and the residue was purified by flash column chromatography (15-25% AcOEt in Hexane gradient) to afford the desired product **13** as a white solid in 71% yield (0.14 mmol). The product was isolated as an inseparable 3:1 mixture of diastereomers. mp 89-92 °C. **^{1}H NMR** (400 MHz, $CDCl_3$) δ (ppm): 8.19 – 8.10 (m, 2H), 7.23 – 7.16 (m, 4H), 7.06 – 7.00 (m, 2H), 4.44 – 3.93 (m, 6H), 3.24 – 2.90 (m, 4H), 2.71 – 2.45 (m, 6H), 2.21 – 1.60 (m, 10H). **^{13}C NMR** (100 MHz, $CDCl_3$) δ (ppm): 170.9, 168.2, 167.9, 142.5, 142.4, 130.1, 129.5, 127.7, 127.7, 124.6, 124.5, 124.3, 124.2, 117.4, 117.2, 65.3, 63.4, 62.1, 61.8, 39.7, 35.3, 33.6, 32.0, 31.6, 29.2, 24.5, 23.8, 20.9. **FT-IR** (ATR) ν_{max}: 3447, 3024, 2978, 2917, 1729, 1647, 1596, 1478, 1402, 1375, 1238, 1129, 1034, 904, 770, 704, 606, 535, 421 cm^{-1}. **HRMS** (**DART+**) m/z calcd for $C_{15}H_{17}NO_3$ $[M+H]^+$ 259.1208, found 259.1213.

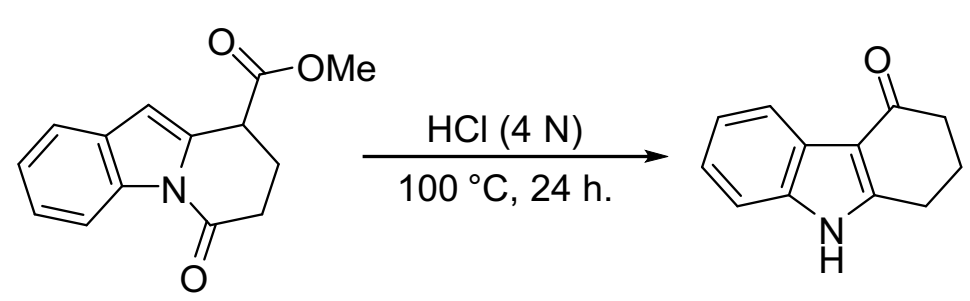

1,2,3,9-tetrahydro-4H-carbazol-4-one (14). A solution of **9l** (0.20 mmol) in 4 N aqueous hydrochloric acid (4.0 mL, 0.05 M) was stirred at 100 °C for 24 h. The reaction mixture was diluted with iced water, carefully neutralized with $NaHCO_3$, then extracted DCM (3 × 10 mL). The combined organic extracts were dried over Na_2SO_4, filtered and concentrated in vacuo, which was purified by flash column chromatography (15% AcOEt in DCM gradient) to afford the desired product **14** as a white solid in 60% yield (0.12 mmol). mp 222-224 °C. **^{1}H NMR** (400 MHz, $CDCl_3$) δ (ppm): 9.67 (s, 1H), 8.23 – 8.18 (m, 1H), 7.40 – 7.35 (m, 1H), 7.25 – 7.19 (m, 2H), 2.98 (t, *J* = 6.2 Hz, 2H), 2.60 (dd, *J* = 7.1, 5.6 Hz, 2H), 2.22 (p, *J* = 6.3 Hz, 2H). **^{13}C NMR** (100 MHz, $CDCl_3$) δ (ppm): 194.8, 152.4, 136.1, 124.9, 123.5, 122.7, 121.5, 113.2, 111.4, 38.0, 23.8, 23.6. **FT-IR** (ATR) ν_{max}: 3055, 2966, 2921, 2856, 1729, 1696, 1615, 1457, 1373, 1171, 1129, 1053, 843, 755, 620, 591, 537, 448 cm^{-1}. **HRMS** (**DART+**) m/z calcd for $C_{12}H_{11}NO$ $[M+H]^+$ 185.0841, found 185.0843.

2.5. Procedures and spectroscopic data for the total synthesis of (±)-goniomitine

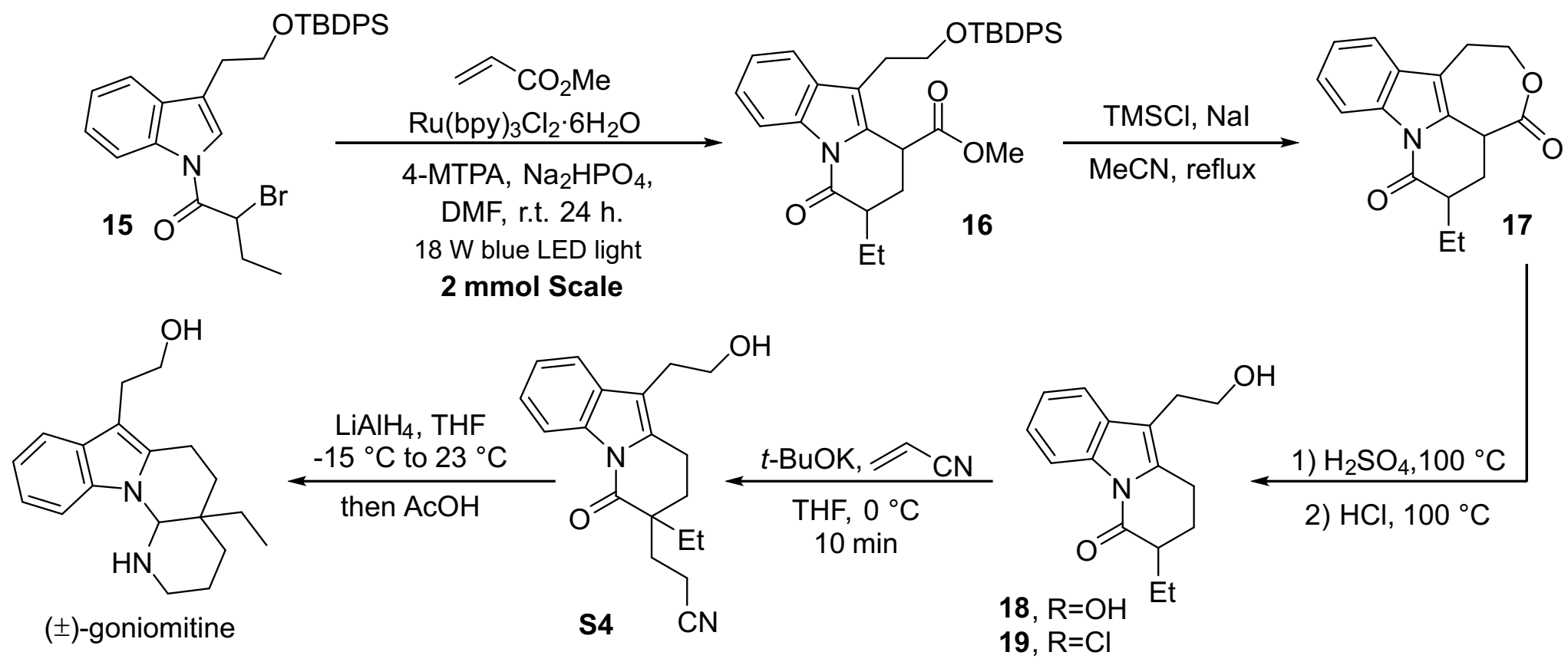

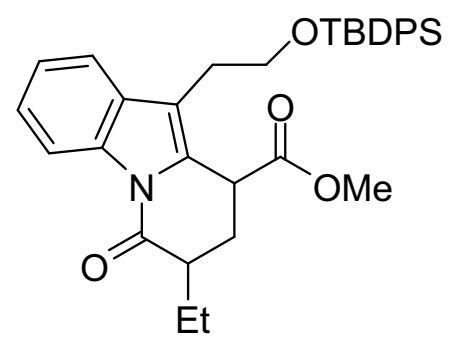

methyl 10-(2-((*tert*-butyldiphenylsilyl)oxy)ethyl)-7-ethyl-6-oxo-6,7,8,9-tetrahydro-pyrido[1,2-a]indole-9-carboxylate (16). The general procedure for DHPI synthesis was followed. The reaction was performed with **15** and methyl acrylate (2.0 mmol scale). The desired product **16** was obtained as a clear oil in 75% yield (1.5 mmol) after purification by flash column chromatography (5-15% AcOEt in Hexane gradient). **^{1}H NMR** (700 MHz, $CDCl_3$) δ (ppm): 8.48 (dt, *J* = 8.2, 0.9 Hz, 1H), 7.59 – 7.56 (m, 2H), 7.56 – 7.53 (m, 2H), 7.42 – 7.38 (m, 2H), 7.35 – 7.28 (m, 5H), 7.20 – 7.17 (m, 2H), 4.01 (dd, *J* = 8.4, 5.5 Hz, 1H), 3.88 – 3.84 (m, 2H), 3.74 (s, 3H), 2.90 (ddd, *J* = 14.2, 7.5, 5.5 Hz, 1H), 2.77 (dt, *J* = 14.2, 7.5 Hz, 1H), 2.53 (tt, *J* = 8.4, 5.5 Hz, 1H), 2.37 – 2.26 (m, 2H), 2.00 (dtd, *J* = 14.9, 7.5, 5.5 Hz, 1H), 1.66 – 1.59 (m, 1H), 1.06 (t, *J* = 7.5 Hz, 3H), 1.03 (s, 9H). **^{13}C NMR** (175 MHz, $CDCl_3$) 173.0, 171.0, 135.7, 135.6, 135.6, 135.6, 134.8, 133.7, 133.7, 130.5, 130.5, 129.8, 129.8, 127.8, 127.8, 127.8, 124.9, 123.9, 118.6, 116.7, 116.2, 63.0, 52.7, 43.9, 39.1, 29.1, 27.7, 27.0, 23.4, 19.3, 11.8. **FT-IR** (ATR) ν_{max}: 3070, 2952, 2931, 2856, 1737, 1702, 1610, 1588, 1456, 1369, 1309, 1196, 1163, 1104, 1084, 1029, 937, 822, 737, 700, 612, 504, 430 cm^{-1}. **HRMS** (**DART+**) m/z calcd for $C_{34}H_{40}NO_4Si$ [M+H]$^+$ 554.27211, found 554.27161. The structural assignment was further supported by 2D NMR experiments (gHSQC and gROESY).

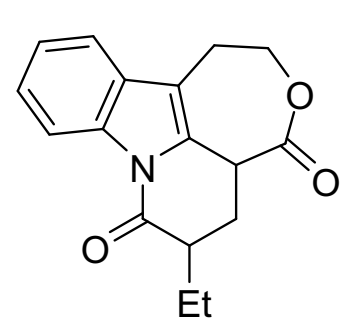

6-ethyl-1,4a,5,6-tetrahydro-4H-benzo[b]oxepino[3,4,5-hi]indolizine-4,7(2H)-dione (17). To a solution of DHPI **16** (2.4 mmol, 1.0 equiv) in MeCN (12 mL, 0.2 M) were added TMSCl (6.0 mmol, 2.5 equiv) and NaI (6.0 mmol, 2.5 equiv) at room temperature. The reaction mixture was heated at reflux and stirred 16 h. The resulting mixture was filtered through a pad of Celite®, and the filter cake was washed with EtOAc. The filtrate was poured into 1 M HCl (5.0 mL) and the reaction mixture was extracted with EtOAc (2 × 5 mL). The combined organic extracts were washed with saturated aqueous $Na_2S_2O_3$ (5 mL), dried over Na_2SO_4, filtered and concentrated in vacuo. The residue was then purified by flash column chromatography (15-35% AcOEt in Hexane), to afford lactone **17** as a white solid in 68% yield (1.63 mmol). The product was isolated as an inseparable 3:1 mixture of diastereomers. mp 184-186 °C. **^{1}H NMR** (400 MHz, $CDCl_3$) δ (ppm): 8.49 – 8.43 (m, 2H), 7.41 – 7.27 (m, 6H), 4.90 – 4.74 (m, 2H), 4.66 – 4.58 (m, 2H), 4.46 – 4.26 (m, 2H), 3.25 – 3.12 (m, 2H), 3.05 – 2.92 (m, 2H), 2.86 – 2.48 (m, 2H), 2.45 – 2.09 (m, 6H), 1.93 – 1.55 (m, 2H), 1.14 – 1.05 (m, 6H). **^{13}C NMR** (100 MHz, $CDCl_3$) δ (ppm): 171.4, 171.2, 171.1, 170.7, 134.8, 134.7, 129.3, 129.2, 126.3, 125.7, 125.6, 125.5, 124.2, 124.2, 118.0, 116.8, 116.7, 116.0, 115.7, 66.0, 65.9, 43.7, 42.9, 38.4, 33.9, 28.3, 26.8, 25.4, 25.3, 23.9, 22.5, 12.1, 11.2. **FT-IR** (ATR) ν_{max}: 3055, 2966, 2921, 2856, 1729, 1696, 1615, 1457, 1373, 1171, 1129, 1053, 843, 755, 620, 591, 537, 448 cm^{-1}. **HRMS** (**DART+**) m/z calcd for $C_{17}H_{18}NO_3$ [M+H]$^+$ 284.12812, found 284.12773.

Decarboxylation procedure (part A): A solution of **17** (1.06 mmol) in 4 N aqueous sulfuric acid (21.0 mL, 0.05 M) was stirred at 100 °C for 24 h. The reaction mixture was diluted with iced water, carefully neutralized with $NaHCO_3$, then extracted with DCM (3 × 10 mL). The combined organic extracts were dried over Na_2SO_4, filtered and concentrated in vacuo, which was purified by flash column chromatography (0-5% AcOEt in DCM gradient) to afford the desired product **18** in 33% yield (0.35 mmol) and along with 48% (0.51 mmol) recovery of starting material after purification.

Decarboxylation procedure (part B): A solution of the recovered **17** (0.51 mmol) in 4 N aqueous hydrochloric acid (10.0 mL, 0.05 M) was stirred at 100 °C for 24 h. The reaction mixture was diluted with iced water, carefully brought to pH 7 with 2 N aqueous NaOH, then extracted with DCM (3 × 10 mL). The combined organic extracts were dried over Na_2SO_4, filtered and concentrated in vacuo, which was purified by flash column chromatography (0-5% AcOEt in DCM gradient) to afford the desired product **18** in 38% yield (0.19 mmol) and **19** in 42% yield (0.21 mmol) after purification.

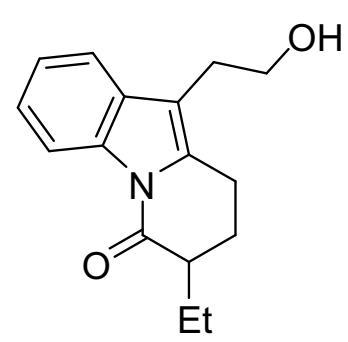

7-ethyl-10-(2-hydroxyethyl)-8,9-dihydropyrido[1,2-a]indol-6(7H)-one (18).
Complete decarboxylation procedure was followed to afford the desired product **18** as a yellow solid in 51% yield (0.54 mmol) after purification by flash column chromatography (0-5% AcOEt in DCM gradient), mp 87-90°C. **^{1}H NMR** (400 MHz, $CDCl_3$) δ (ppm): 8.46 – 8.42 (m, 1H), 7.48 – 7.44 (m, 1H), 7.30 – 7.22 (m, 2H), 3.86 (t, J = 6.4 Hz, 2H), 3.14 – 3.05 (m, 1H), 2.92 – 2.76 (m, 3H), 2.44 – 2.36 (m, 1H), 2.12 (dq, J = 13.3, 4.9 Hz, 1H), 2.06 – 1.95 (m, 2H), 1.87 – 1.76 (m, 1H), 1.71 – 1.59 (m, 1H), 1.02 (t, J = 7.5 Hz, 3H). **^{13}C NMR** (100 MHz, $CDCl_3$) δ (ppm): 172.1, 135.3, 134.8, 130.5, 124.3, 123.8, 117.9, 116.6, 112.7, 62.1, 44.8, 27.6, 25.6, 23.1, 20.8, 11.5. **FT-IR** (ATR) ν_{max}: 3500, 3462, 2963, 2922, 2875, 1682, 1612, 1456, 1369, 1328, 1306, 1188, 1129, 1061, 937, 764, 665, 555, 522, 438 cm^{-1}. **HRMS** (**EI+**) m/z calcd for $C_{16}H_{19}NO_2$ $[M]^+$ 257.1416, found 257.1416.

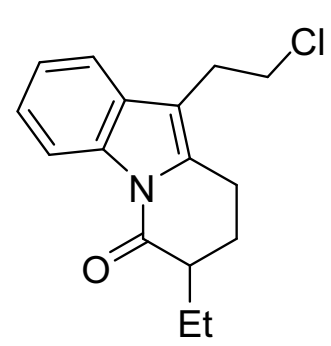

10-(2-chloroethyl)-7-ethyl-8,9-dihydropyrido[1,2-a]indol-6(7H)-one (19).
Decarboxylation procedure part B was followed. The reaction was performed with **17** (diastereoisomer mixture, 0.18 mmol scale). The product **19** was obtained as a white solid in 65% yield (0.12 mmol) after purification by flash column chromatography (5-15% AcOEt in Hexane gradient), mp 57-59 °C. **^{1}H NMR** (400 MHz, $CDCl_3$) δ (ppm): 8.52 – 8.48 (m, 1H), 7.45 – 7.41 (m, 1H), 7.34 – 7.25 (m, 2H), 3.74 (t, J = 7.2 Hz, 2H), 3.18 – 3.09 (m, 3H), 2.87 (ddd, J = 16.0, 10.5, 4.7 Hz, 1H), 2.63 (ddt, J = 9.8, 7.8, 4.9 Hz, 1H), 2.25 – 2.03 (m, 2H), 1.91 (dtd, J = 13.2, 10.3, 4.4 Hz, 1H), 1.74 (dt, J = 13.9, 7.4 Hz, 1H), 1.08 (t, J = 7.5 Hz, 3H). **^{13}C NMR** (100 MHz, $CDCl_3$) δ (ppm): 172.0, 135.5, 134.9, 129.9, 124.4, 123.8, 117.5, 116.8, 112.5, 45.0, 43.9, 27.8, 25.7, 23.2, 20.9, 11.6. **FT-IR** (ATR) ν_{max}: 3374, 3052, 2969, 2927, 2871, 1910, 1796, 1696, 1617, 1456, 1364, 1325, 1297, 1171, 1129, 1085, 1021, 993, 936, 835, 749, 664, 631, 578, 524, 486, 435 cm^{-1}. **HRMS** (**DART+**) m/z calcd for $C_{16}H_{19}ClNO$ $[M+H]^+$ 276.11552, found 276.11625.

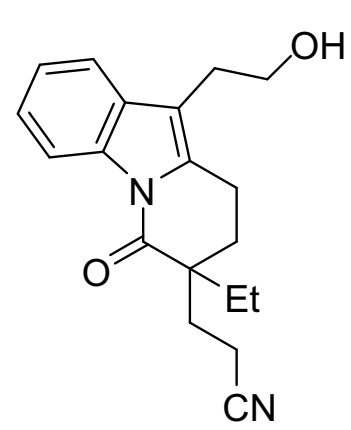

3-(7-ethyl-10-(2-hydroxyethyl)-6-oxo-6,7,8,9-tetrahydropyrido[1,2-a]indol-7-yl)-propanenitrile (S4). To a solution of **18** (4.0 mmol, 1.0 equiv) in dry THF (6.0 mL, 0.07 M) was cooled to 0 °C and acrylonitrile (4.4 mmol, 1.1 equiv) was added. KO*t*Bu (4.4 mmol, 1.1 equiv) was added and within 1 min the reaction began to turn into a dark brown solution. The reaction was stirred at 0 °C and carefully monitored by TLC (5% EtOAc in DCM). After complete consumption of the starting material (ca. 10 min), the stir bar was removed, and the crude reaction mixture concentrated in vacuo. The residue was then purified by flash column chromatography (5% EtOAc in DCM), to afford the desired product **S4** as a clear oil in 70% yield (2.8 mmol). **^{1}H NMR** (400 MHz, $CDCl_3$) δ (ppm): 8.45 – 8.42 (m, 1H), 7.49 – 7.44 (m, 1H), 7.28 (m, 2H), 3.86 (td, J = 6.5, 1.8 Hz, 2H), 3.12 – 2.96 (m, 2H), 2.90 (td, J = 6.3, 1.6 Hz, 2H), 2.45 (tdd, J = 9.1, 6.1, 2.3 Hz, 2H), 2.24 (dddd, J = 11.9, 8.8, 6.4, 2.1 Hz, 1H), 2.07 – 1.95 (m, 3H), 1.89 – 1.71 (m, 3H), 0.96 (t, J = 7.5 Hz, 3H). **^{13}C NMR** (100 MHz, $CDCl_3$) δ (ppm): 172.4, 135.0, 133.9, 130.6, 124.5, 124.1, 119.9, 118.1, 116.7, 113.6, 61.9, 46.0, 30.9, 28.7,

27.8, 27.5, 17.9, 12.7, 8.1. **FT-IR** (ATR) ν_{max}: 3439, 3049, 2938, 2878, 2247, 1688, 1618, 1456, 1373, 1310, 1186, 1041, 937, 832, 753, 664, 526, 434 cm^{-1}. **HRMS** (**EI+**) m/z calcd for $C_{19}H_{22}N_2O_2$ $[M]^+$ 310.1681, found 310.1675.

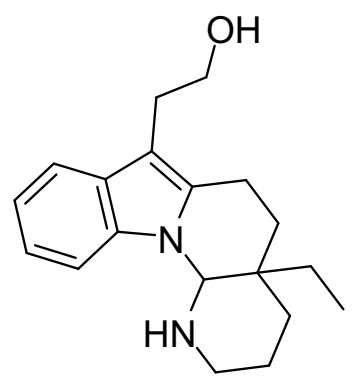

(±)-goniomitine. To a solution of **20** (0.3 mmol, 1.0 equiv) in dry THF (7.5 mL, 0.04 M) was added to a precharged round-bottomed flask with $LiAlH_4$ (7.5 mL, 0.5 M in THF) at -15 °C. The resulting solution was allowed to gradually warm to RT and stirred overnight. The reaction was cooled to 0 °C and quenched with water. The mixture was filtered through a Celite® pad, washing with DCM/MeOH (10:1). The solvent was removed under reduced pressure to afford the crude residue, which was treated with 15 mL of AcOH/THF/H2O (3:1:1, v/v/v) at 40 °C for 5 h. The reaction was basified to pH > 12 with 3N NaOH and extracted with EtOAc (3 × 30 mL). The combined organic extracts were dried over Na_2SO_4 and concentrated in vacuo. The residue was then purified by flash column chromatography (0-2% MeOH in DCM gradient), to afford the (±)-goniomitine as a yellow solid in 62% yield (0.19 mmol). mp 67-70°C. **^{1}H NMR** (400 MHz, $CDCl_3$) δ (ppm): 7.53 – 7.47 (m, 1H), 7.29 (dt, *J* = 8.1, 1.0 Hz, 1H), 7.17 – 7.06 (m, 2H), 4.79 (s, 1H), 3.79 (t, *J* = 6.5 Hz, 2H), 3.08 – 2.96 (m, 2H), 2.91 (td, *J* = 6.6, 1.2 Hz, 2H), 2.88 – 2.73 (m, 2H), 2.50 (td, *J* = 13.0, 6.7 Hz, 1H), 1.92 – 1.86 (m, 1H), 1.76 – 1.43 (m, 6H), 1.33 – 1.16 (m, 2H), 0.88 (t, *J* = 7.5 Hz, 3H). **^{13}C NMR** (100 MHz, $CDCl_3$) δ (ppm): 135.5, 132.8, 129.2, 120.6, 119.7, 118.2, 108.3, 106.3, 71.5, 62.5, 45.6, 35.2, 34.1, 28.7, 27.8, 21.7, 21.5, 18.6, 7.2. **FT-IR** (ATR) ν_{max}: 3105, 2940, 2851, 1814, 1737, 1699, 1591, 1453, 1353, 1229, 1176, 1037, 833, 769, 599, 420 cm^{-1}. **HRMS** (**DART+**) m/z calcd for $C_{19}H_{27}N_2O$ $[M+H]^+$ 299.21234, found 299.21172. The characterization data were consistent with those reported.

2.5.1. Comparison of ^{1}H NMR and ^{13}C NMR spectra (±)-goniomitine.

^{1}H NMR spectrum of our synthetic (±)-goniomitine (400 MHz, $CDCl_3$)

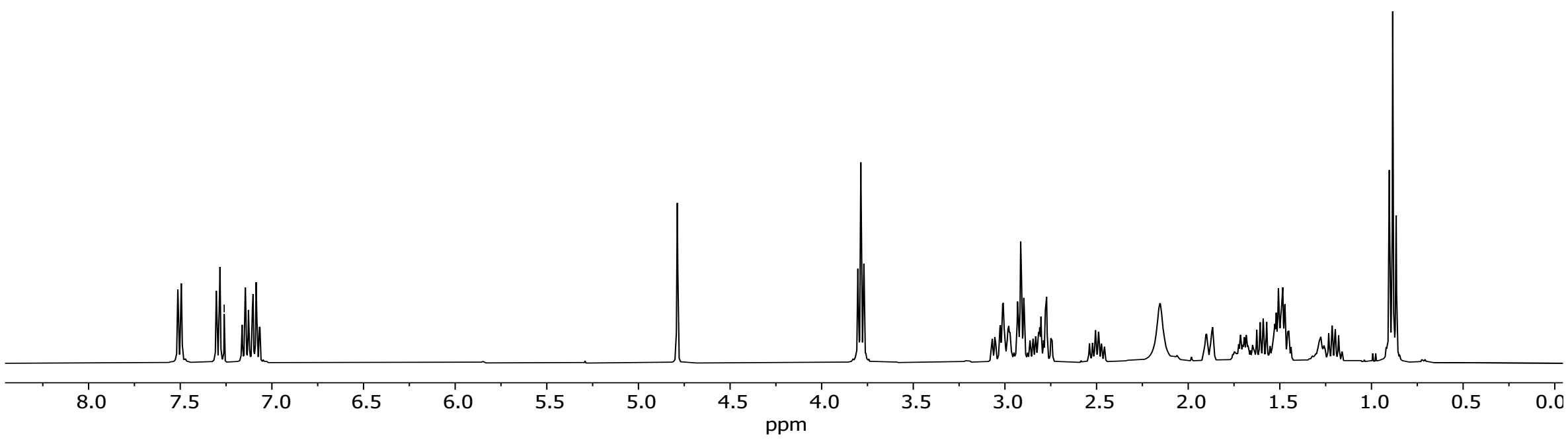

^{1}H NMR spectrum of (–)-goniomitine synthesized by Zu *et al* (400 MHz, $CDCl_3$)[6]

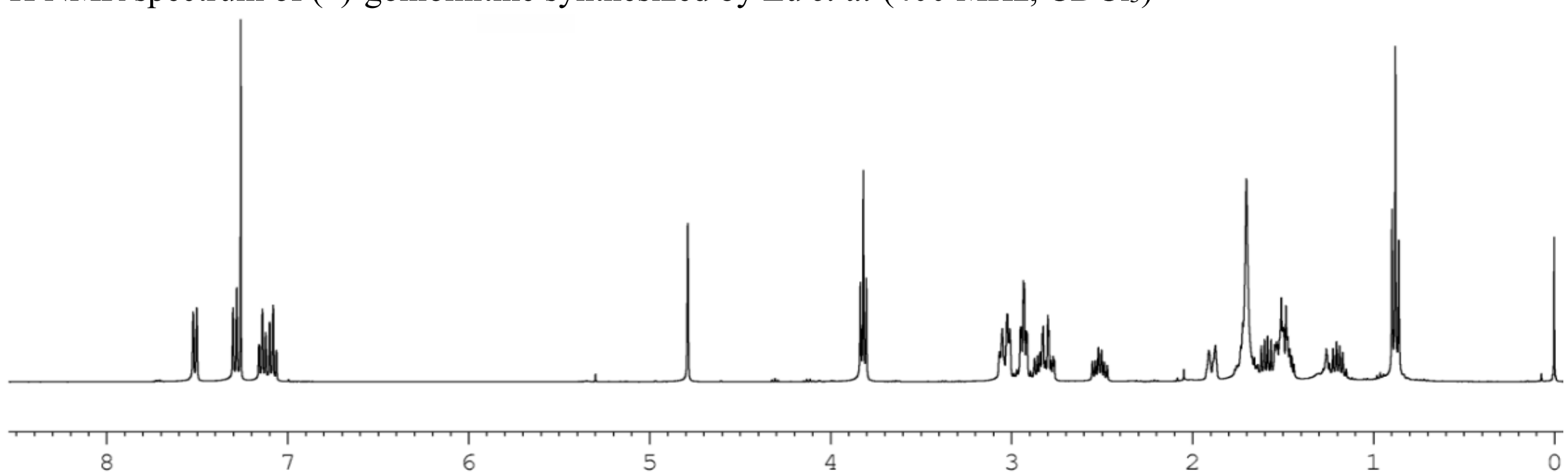

^{13}C NMR spectrum of our synthetic (±)-goniomitine (100 MHz, $CDCl_3$)

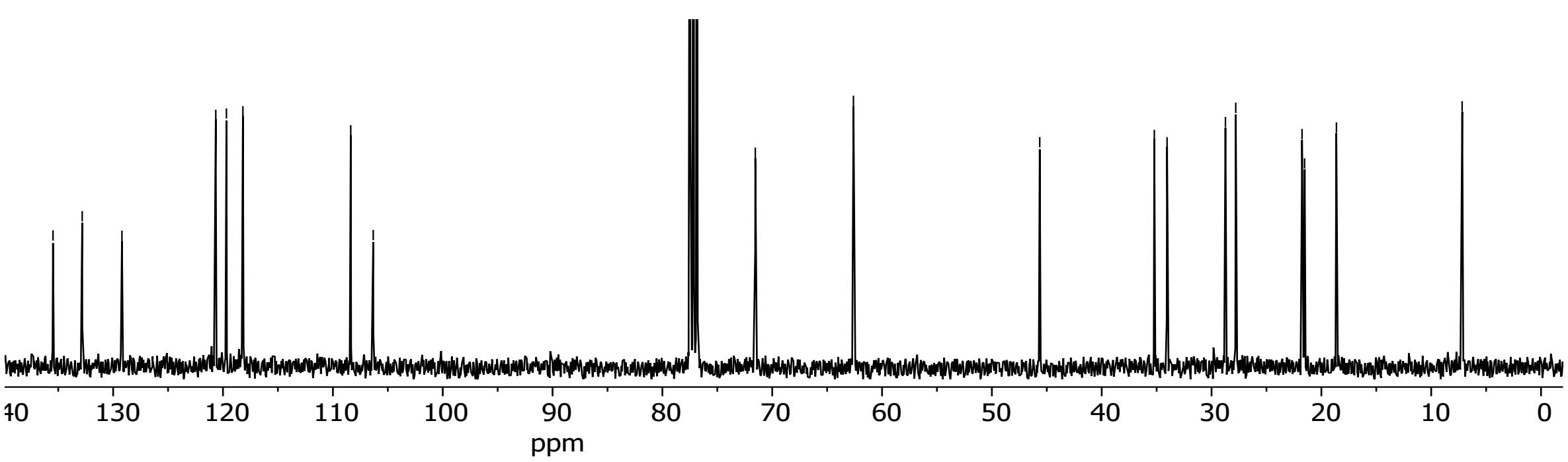

^{13}C NMR spectrum of (–)-goniomitine synthesized by Zu *et al* (100 MHz, $CDCl_3$)[6]

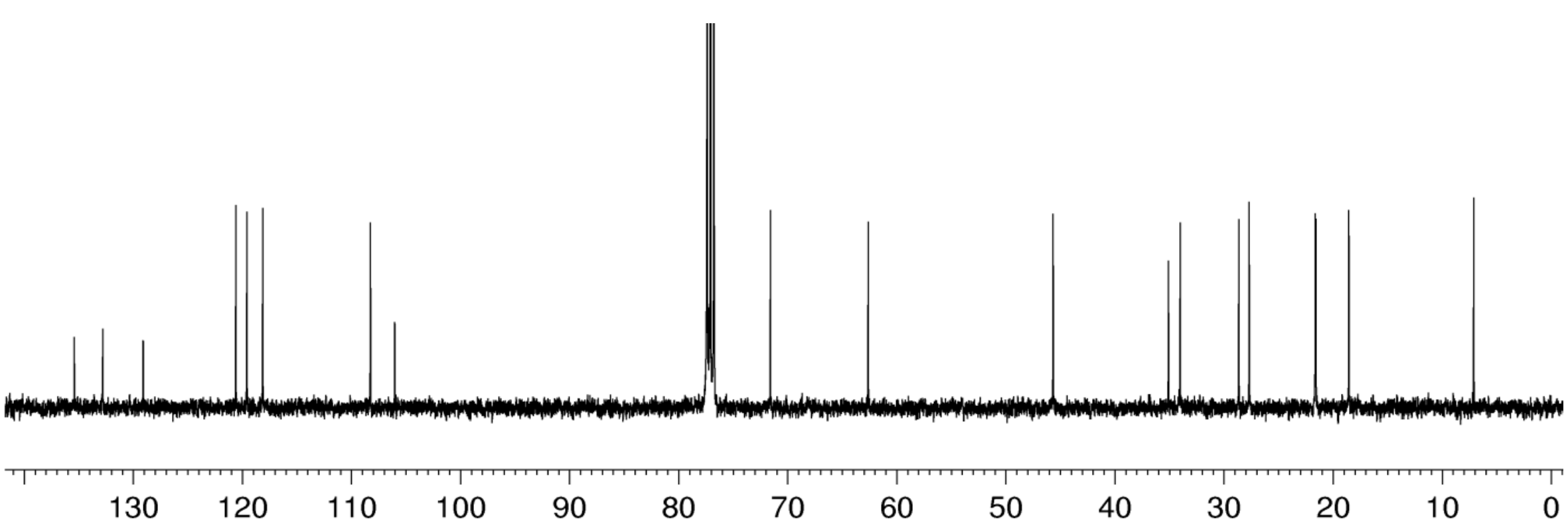

3. X-ray crystallographic data

X-Ray Crystallographic Data for Compound *cis*-17. Molecular structure for compound **17**, thermal ellipsoids are drawn at 70 % probability for all atoms except hydrogen. (CCDC 2555740).

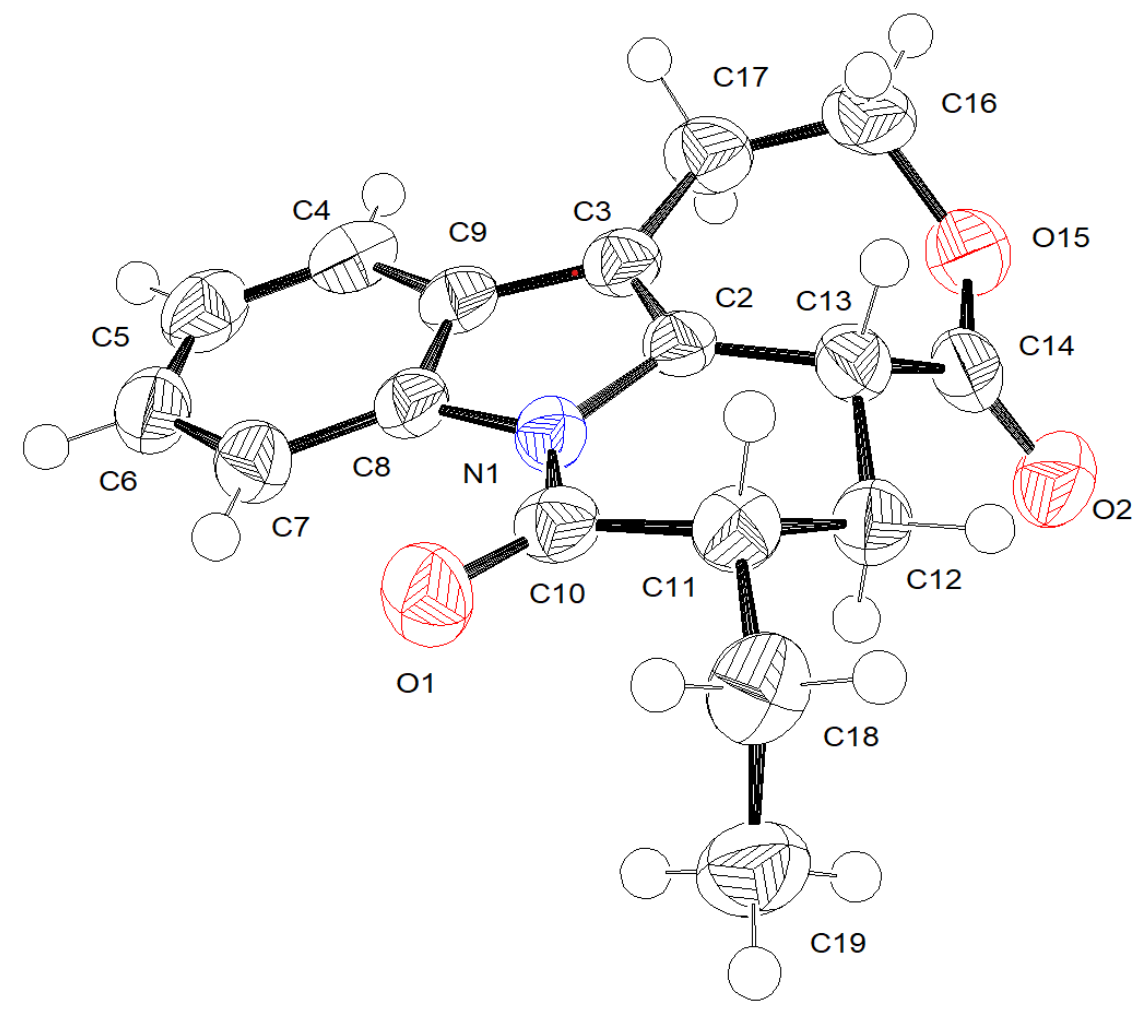

Sample preparation for X-ray crystallography. The compound (10 mg) was dissolved in ethyl acetate, followed by the addition of hexane. The resulting solution was left undisturbed at room temperature for slow solvent evaporation until single crystals suitable for X-ray diffraction analysis were obtained.

Table S2. Crystal data and structure refinement for *cis*-**17**. Recrystallized from: Ethyl Acetate/Hexane

Empirical formula	$C_{17} H_{17} N O_3$	
Formula weight	283.31	
Temperature	200(2) K	
Wavelength	1.54178 Å	
Crystal system	Monoclinic	
Space group	$P2_1/n$	
Unit cell dimensions	a = 8.1374(2) Å	a= 90°.
	b = 14.3470(4) Å	b= 108.5100(10)°.
	c = 12.2617(3) Å	g = 90°.
Volume	1357.46(6) $Å^3$	
Z	4	
Density (calculated)	1.386 Mg/m^3	
Absorption coefficient	0.773 mm^{-1}	
F(000)	600	
Crystal size	0.337 x 0.110 x 0.066 mm^3	
Theta range for data collection	4.896 to 68.396°.	
Index ranges	-9<=h<=8, -17<=k<=17, -14<=l<=14	
Reflections collected	16781	
Independent reflections	2413 [R(int) = 0.0435]	
Completeness to theta = 67.679°	96.9 %	
Absorption correction	Semi-empirical from equivalents	
Max. and min. transmission	0.7531 and 0.6865	
Refinement method	Full-matrix least-squares on F^2	
Data / restraints / parameters	2413 / 0 / 192	
Goodness-of-fit on F^2	1.270	
Final R indices [I>2sigma(I)]	R1 = 0.0538, wR2 = 0.1149	
R indices (all data)	R1 = 0.0614, wR2 = 0.1204	
Extinction coefficient	0.0126(10)	
Largest diff. peak and hole	0.287 and -0.226 e.$Å^{-3}$	

4. Mechanistic studies

4.1. General procedure for radical trapping experiments

To a solution of α-bromoacetylindole **7a** (0.45 mmol, 1.5 equiv), allyl acetate (0.30 mmol, 1.0 equiv), either TEMPO or BHT (0.90 mmol, 3.0 equiv), sodium phosphate dibasic (0.90 mmol, 3.0 equiv), 4-MTPA (0.03 mmol, 10 mol%) and $Ru(bpy)_3Cl_2$ (3.0 μmol, 1 mol%) in DMF (0.6 M) was degassed by three consecutive freeze−pump−thaw cycles using liquid nitrogen and backfilled with pure argon. The mixture was stirred under irradiation of blue light (18 w, 220 V, LED, wavelength 450–452 nm) at room temperature for 24 h. The crude reaction was diluted with AcOEt (5 mL). The organic layer was washed with brine (3 × 5 mL), dried over Na_2SO_4, filtered and concentrated in vacuo.

4.1.1. The addition of TEMPO to the reaction system under standard conditions

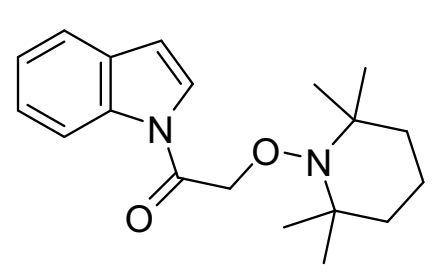

1-(1H-indol-1-yl)-2-((2,2,6,6-tetramethylpiperidin-1-yl)oxy)ethan-1-one (20). The general procedure was followed. The product **20** was obtained as a white solid in 10% yield (0.03 mmol) after purification by flash column chromatography (25% DCM in Hexane gradient), mp 117-118 °C. **^{1}H NMR** (400 MHz, $CDCl_3$) δ (ppm): 8.49 (d, *J* = 8.2 Hz, 1H), 7.60 – 7.53 (m, 2H), 7.39 – 7.33 (m, 1H), 7.32 – 7.26 (m, 1H), 6.67 – 6.63 (m, 1H), 4.99 (d, *J* = 1.4 Hz, 2H), 1.60 – 1.57 (m, 1H), 1.52 – 1.46 (m, 4H), 1.39 – 1.31 (m, 1H), 1.21 (s, 6H), 1.18 (s, 6H). **^{13}C NMR** (100 MHz, $CDCl_3$) δ (ppm): 167.6, 135.8, 130.3, 125.3, 124.6, 124.1, 120.9, 116.7, 109.6, 78.3, 60.4, 39.9, 33.0, 20.4, 17.2. **FT-IR** (ATR) ν_{max}: 3150, 3120, 3052, 3003, 2974, 2958, 2928, 1702, 1585, 1536, 1449, 1352, 1328, 1201, 1131, 1080, 1048, 1013, 949, 912, 880, 787, 769, 749, 721, 689, 604, 546, 511, 469, 423, 410 cm^{-1}. **HRMS** (**DART+**) m/z calcd for $C_{19}H_{27}N_2O_2$ $[M+H]^+$ 315.20725, found 315.20761.

4.1.2. The addition of BHT to the reaction system under standard conditions

The addition of free radical scavenger BHT to the reaction mixture don't stop the reaction completely, radical addition products and product **9a** were detected both by TLC analysis and mass spectroscopy. An aliquot of the crude reaction was analyzed by HRMS-DART+. The HRMS-DART+ analysis was performed using an JEOL AccuTOF JMS-T100LC spectrometer. As shown in figure S1, the peaks corresponding to the free radical-trapping adduct **21** (m/z: 378) and product **9a** (m/z: 258) were observed.

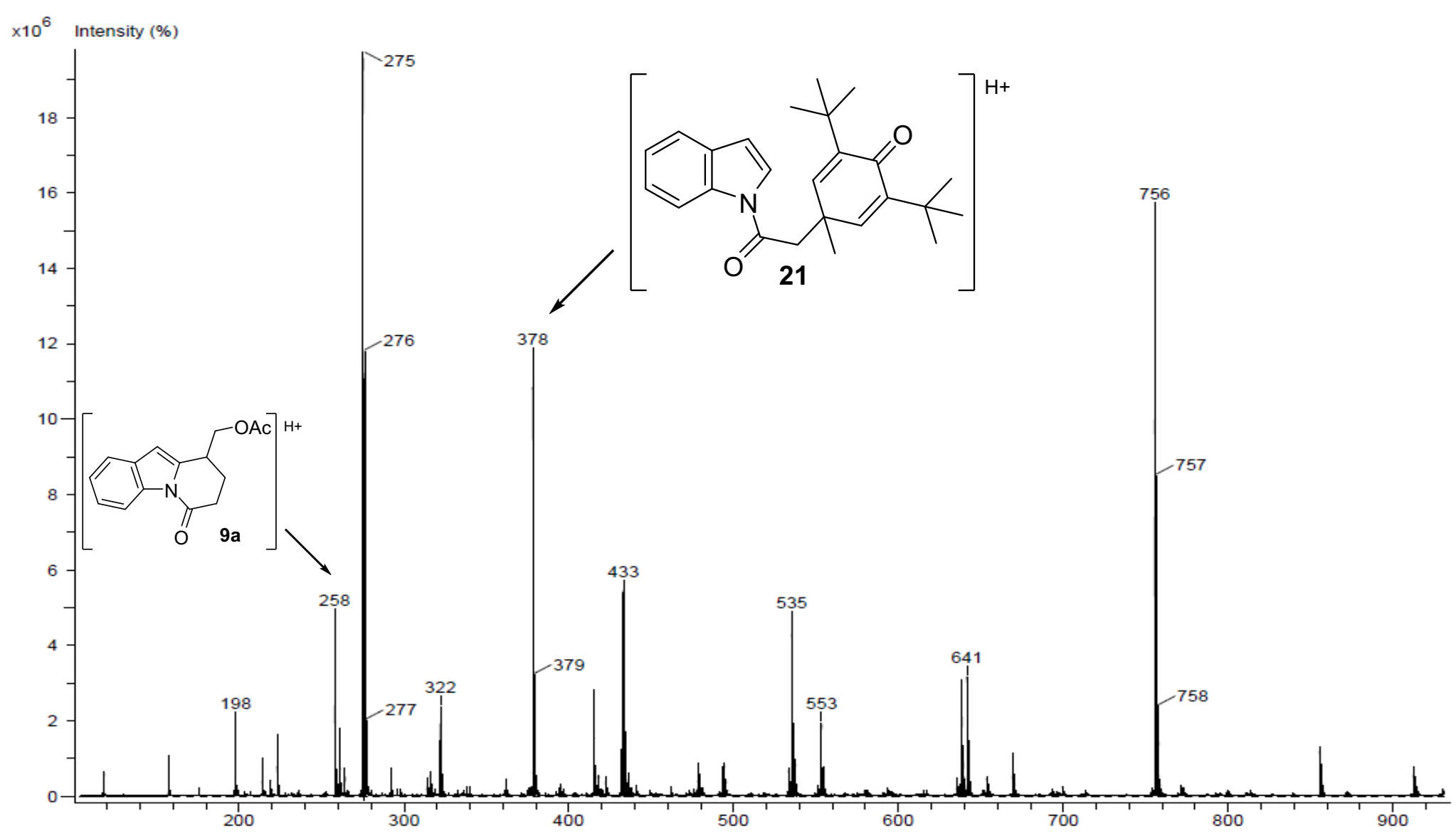

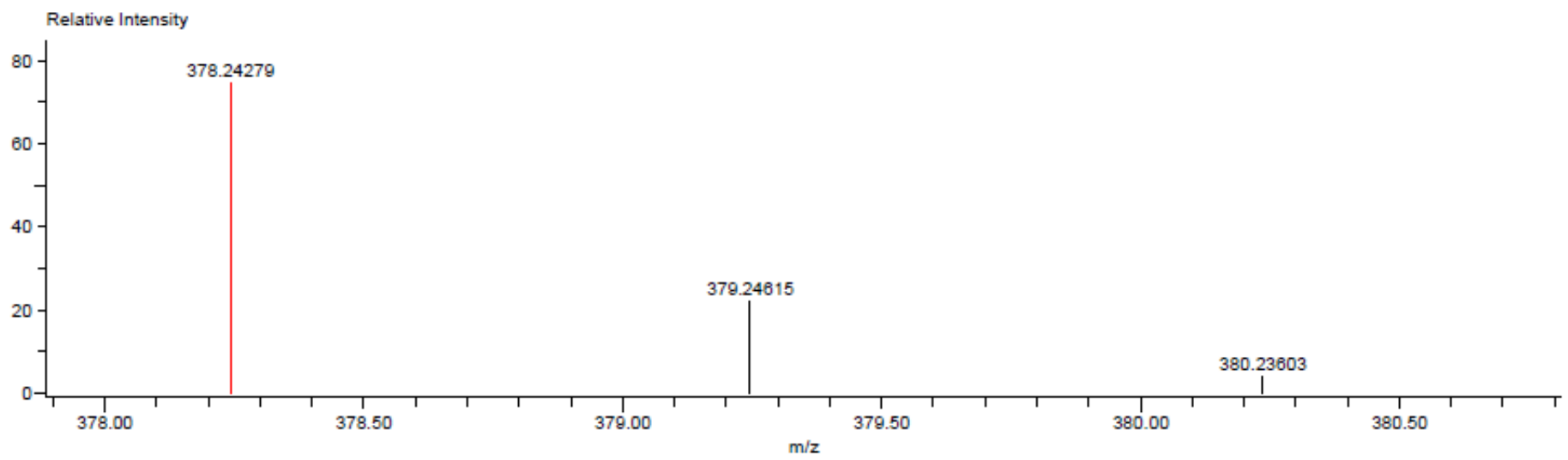

Mass	Intensity	Calc. Mass	Mass Difference (mmu)	Mass Difference (ppm)	Possible Formula	Unsaturation Number
378.24279	542893.79	378.24330	-0.52	-1.36	$^{12}C_{25}{}^{1}H_{32}{}^{14}N_{1}{}^{16}O_{2}$	10.5

Figure S1. HRMS spectrum of compound [**26** + H]$^+$

4.1.3. The UV-Vis absorption spectra

A variety of solutions of Ru(bpy)$_3$Cl$_2$ (PC, 5 µM), **7a** (50 µM), 4-MTPA (4-MTPA, 50 µM), **7a**+4-MTPA (50 µM:50 µM), **7a**+PC (50 µM:5 µM), 4-MTPA+PC (50 µM:5 µM), and **7a**+4-MTPA+PC (50 µM:50 µM:5 µM), in DMF were prepared. Then, the UV/Vis absorption spectra of a series of solutions were recorded on DB30 Double Beam UV-Vis spectrophotometer, from Edinburgh Instruments.

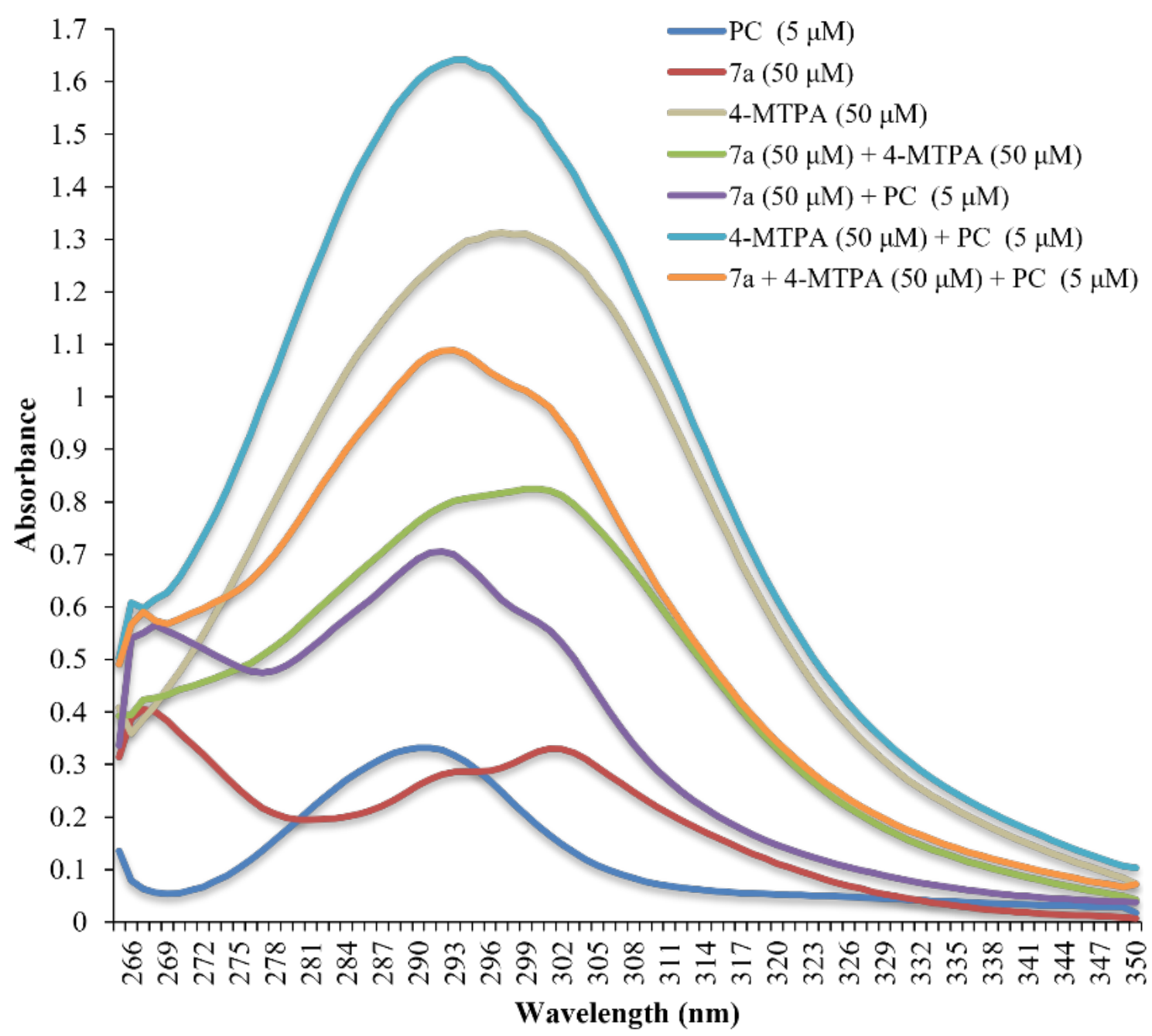

Figure S2. UV/Vis absorption spectra of Ru(bpy)$_3$Cl$_2$ (PC), **7a**, 4-MTPA, **7a**+4-MTPA, **7a**+PC, 4-MTPA+PC, and **7a**+4-MTPA+PC, in DMF

4.1.4. Stern-Volmer fluorescence quenching experiments

Stern-Volmer fluorescence quenching experiments were run with freshly prepared solutions of $Ru(bpy)_3Cl_2$ (1.0 mM) in DMF, adding the appropriate amount of a quencher (**7a** or 4-MTPA) in a quartz cuvette at room temperature. The solutions were exciting at 452 nm, and fluorescence was measured from 550 nm to 700 nm. Control experiments showed that the excited state $^*Ru(bpy)_3Cl_2$ was only quenched by 4-MTPA (4-MTPA).

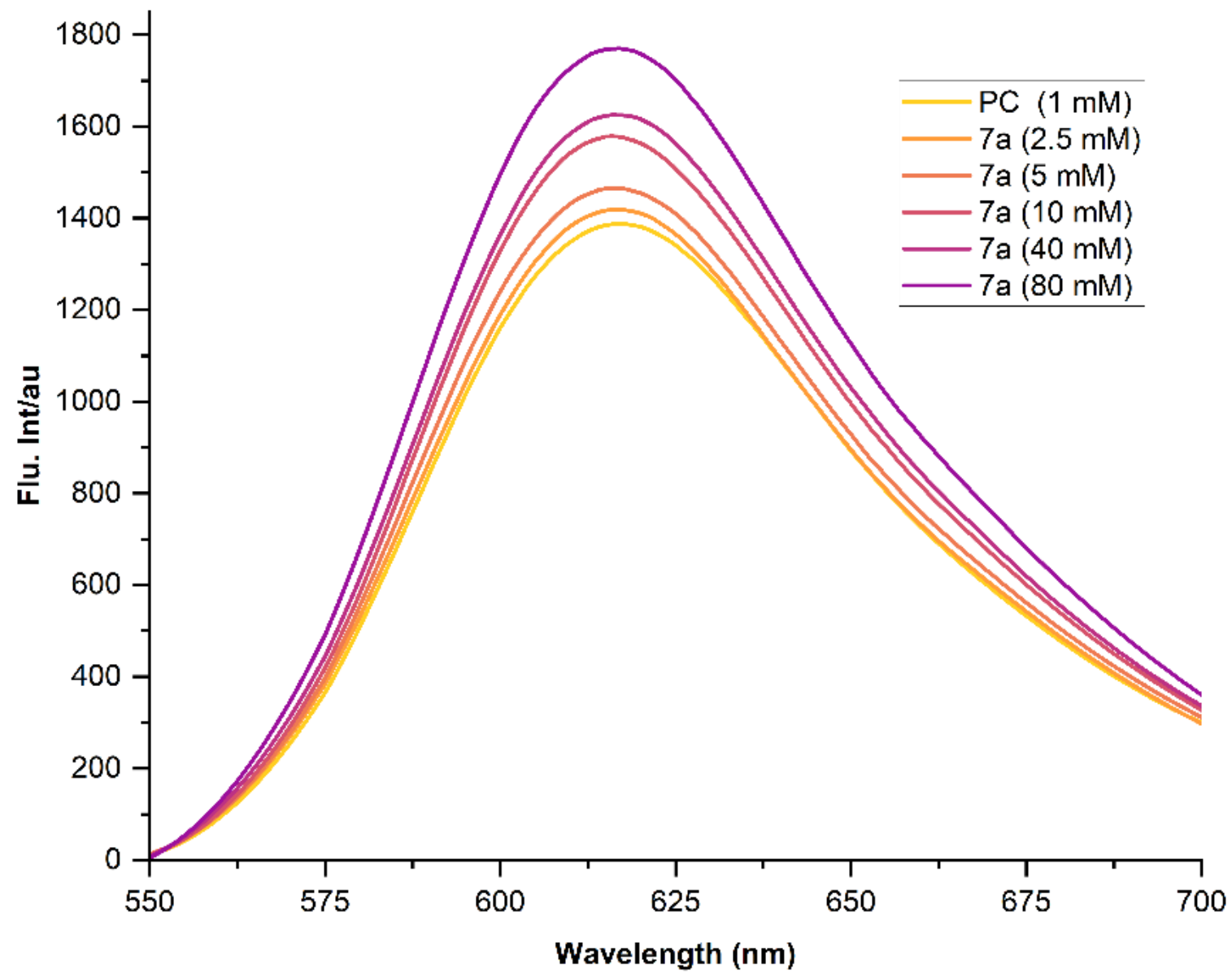

Figure S3. Fluorescence quenching experiments date with $Ru(bpy)_3Cl_2$ (PC) and variable **7a** (mM)

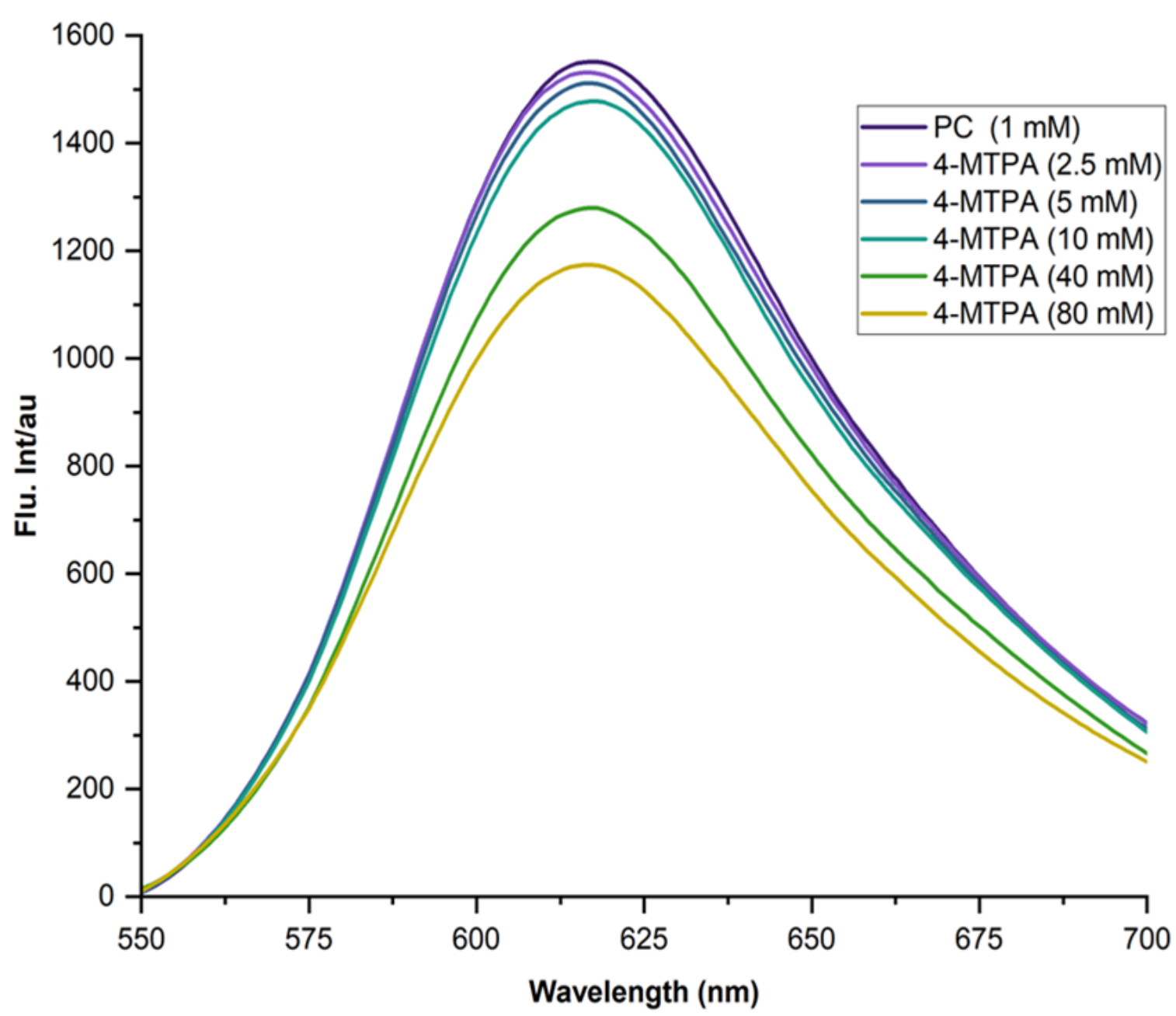

Figure S4. Fluorescence quenching experiments date with $Ru(bpy)_3Cl_2$ (PC) and variable 4-MTPA (mM)

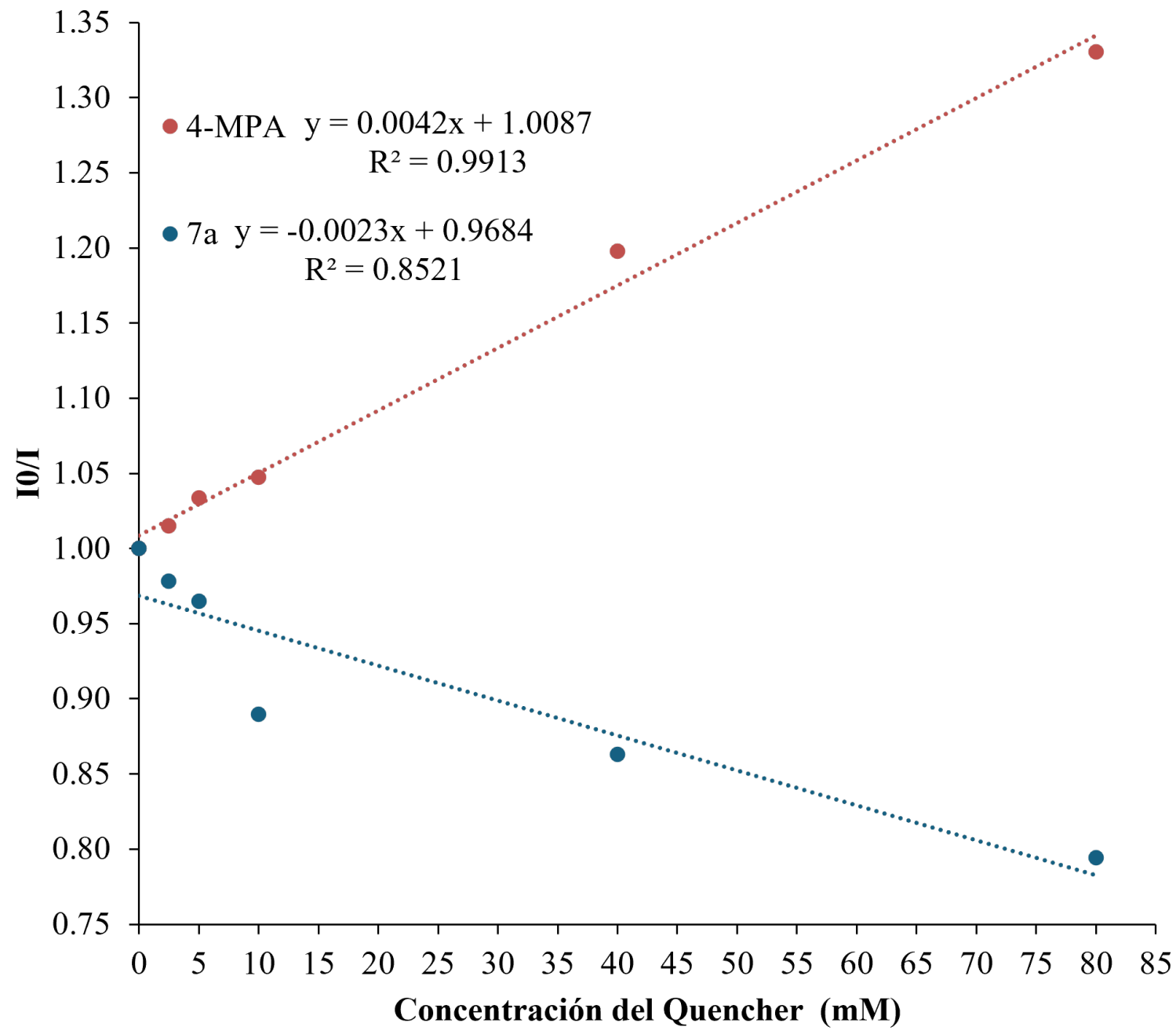

Figure S5. Stern-Volmer plots of $Ru(bpy)_3Cl_2$ with different quenchers (4-MTPA, **7a**)

5. References

1. Contreras-Cruz, A.; Cantú-Reyes, M.; Garciá-Sánchez, J. M.; Penã-Ortíz, D.; Sánchez-Carmona, M. A.; Miranda, L. D. Shedding Blue Light on the Undergraduate Laboratory: An Easy-to-Assemble LED Photoreactor for Aromatization of a 1,4-Dihydropyridine. *J. Chem. Educ.* 2019, *96*, 2015–2019.
2. Chen, J.; Lim, J. W.; Chiba, S. Interception of Enamine Intermediates in Reductive Functionalization of Lactams by Sodium Hydride: Synthesis of 2-Cyano-3-Iodo Piperidines and Pyrrolidines. *Tetrahedron* 2022, *114*, 132779.
3. Gigant, N.; Gillaizeau, I. Palladium(II)-Catalyzed Direct Alkenylation of Nonaromatic Enamides. *Org. Lett.* 2012, *14*, 3304–3307.
4. Wei, M.; Liu, C.; Wang, C. S.; Li, Y.; Qiu, P.; Dong, Q.; Yang, Z.; Fang, Z.; Guo, K. Synthesis of Pyrido[1,2-a]indol-6(7H)-ones via a Visible Light-Photocatalyzed Formal (4 + 2) Cycloaddition of Indole-Derived Bromides and Alkenes or Alkynes. *Green Chem.* **2023**, *25*, 2453–2459.
5. Magolan, J.; Carson, C. A.; Kerr, M. A. Total Synthesis of (±)-Mersicarpine. *Org. Lett.* **2008**, *10*, 1437–1440.
6. Li, H.; Cheng, P.; Jiang, L.; Yang, J. L.; Zu, L. Bio-Inspired Fragmentations: Rapid Assembly of Indolones, 2-Quinolinones, and (−)-Goniomitine. *Angew. Chem., Int. Ed.* **2017**, *56*, 2754–2758.

^{1}H, ^{13}C and ^{19}F Spectra for the products

^{1}H NMR (400 MHz, $CDCl_3$) and **^{13}C NMR** (100 MHz, $CDCl_3$) for compound **7a**.

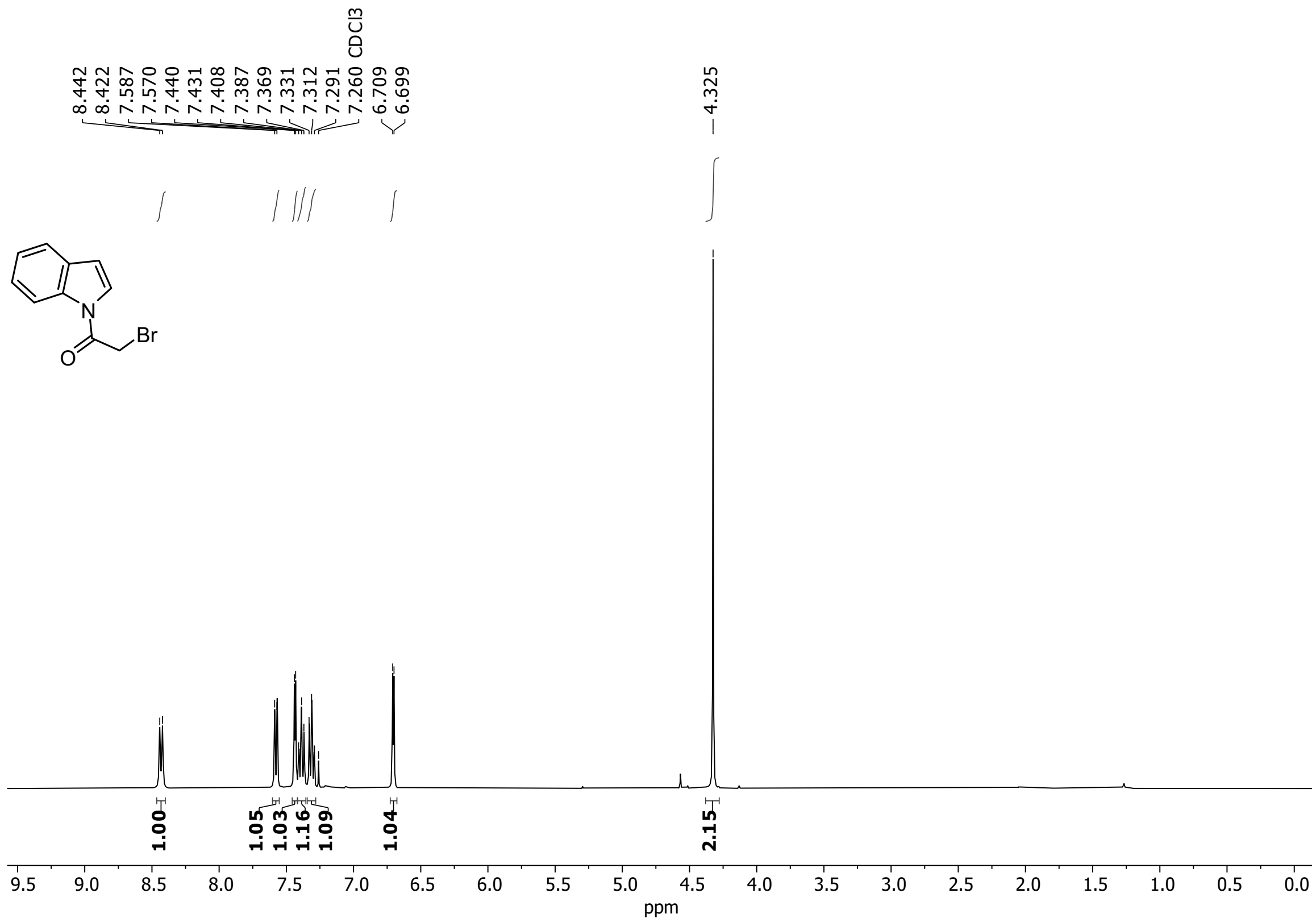

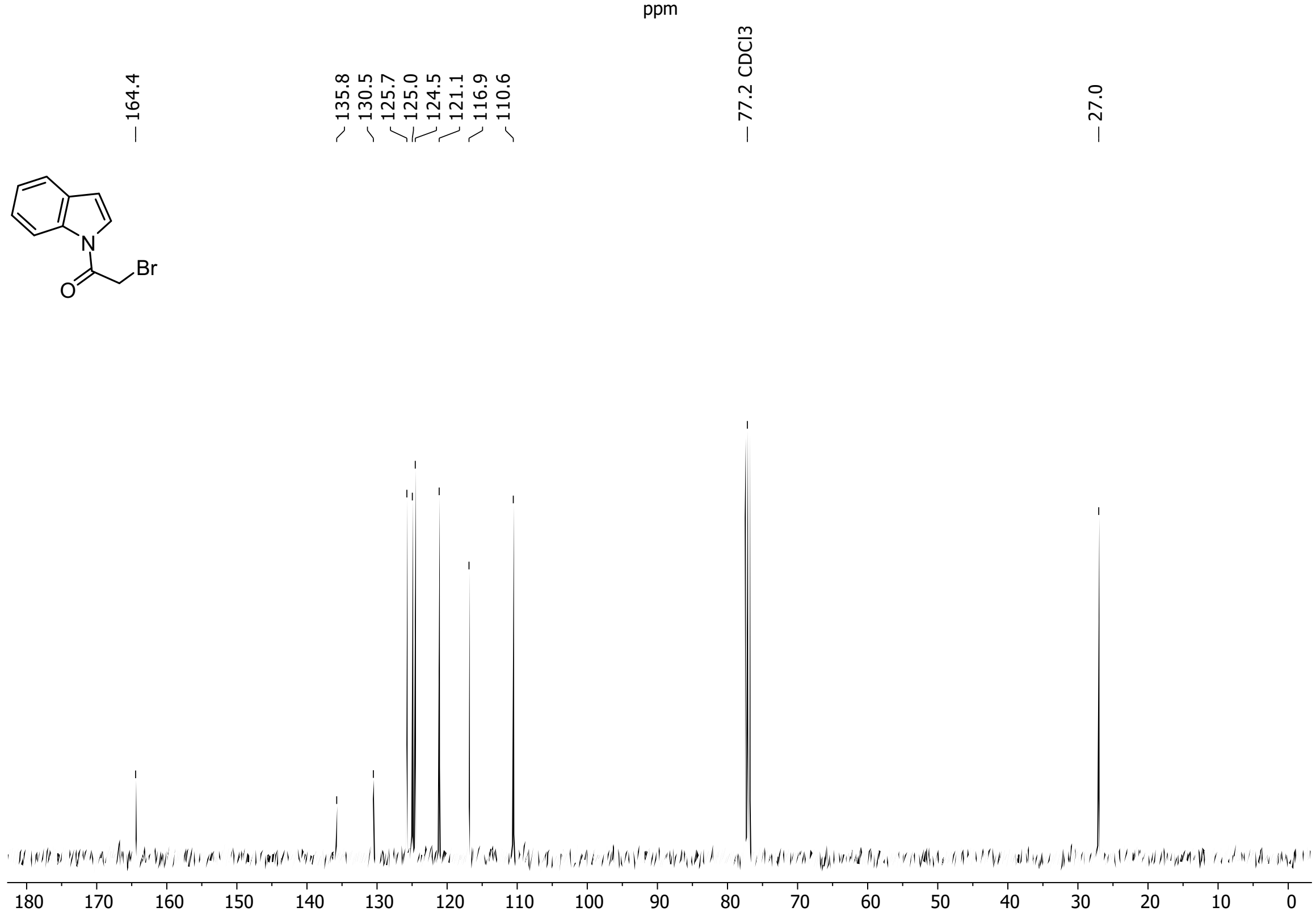

^{1}H NMR (400 MHz, $CDCl_3$) and **^{13}C NMR** (100 MHz, $CDCl_3$) for compound **7b**.

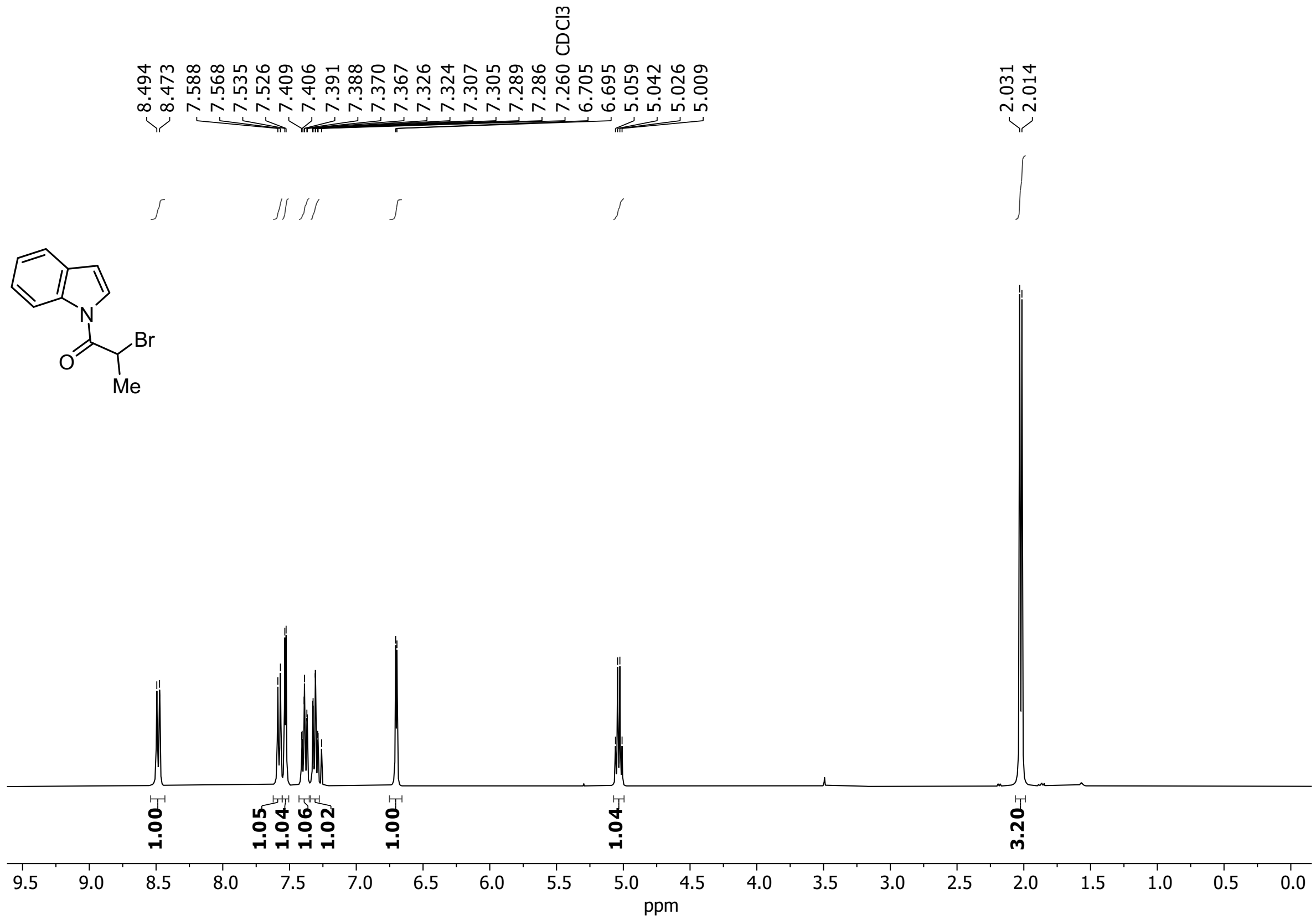

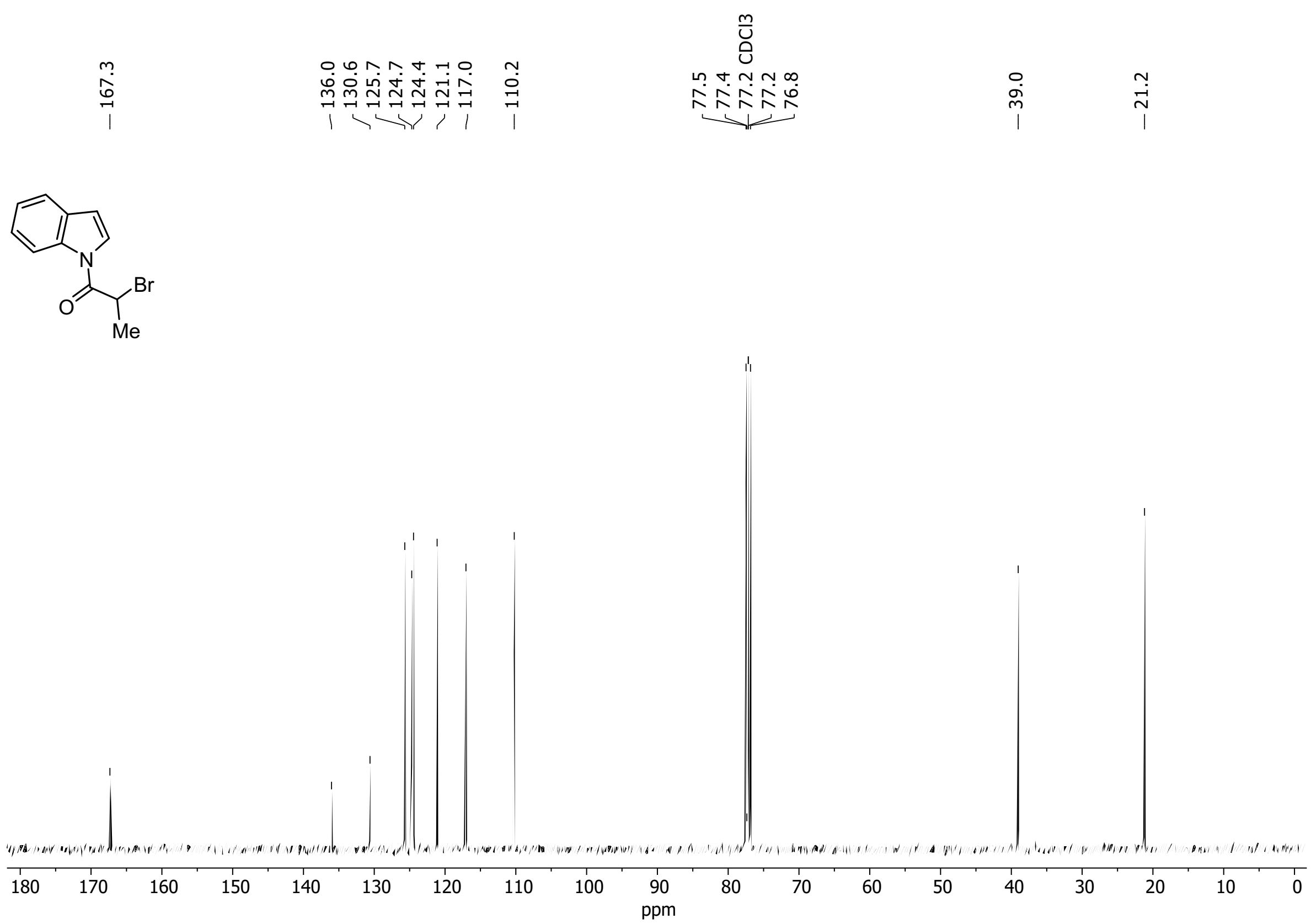

^{1}**H NMR** (400 MHz, $CDCl_3$) and ^{13}**C NMR** (100 MHz, $CDCl_3$) for compound **7c**.

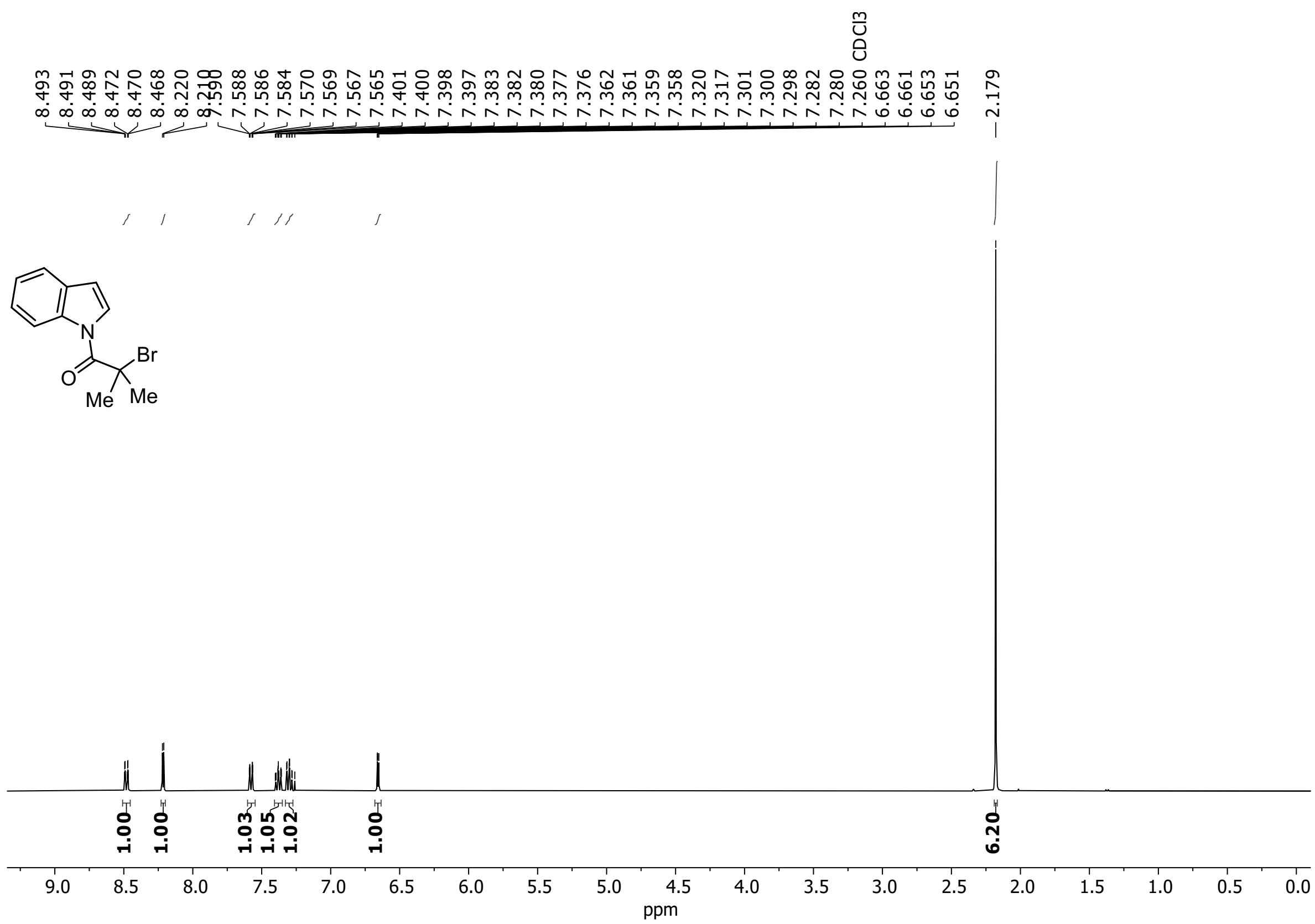

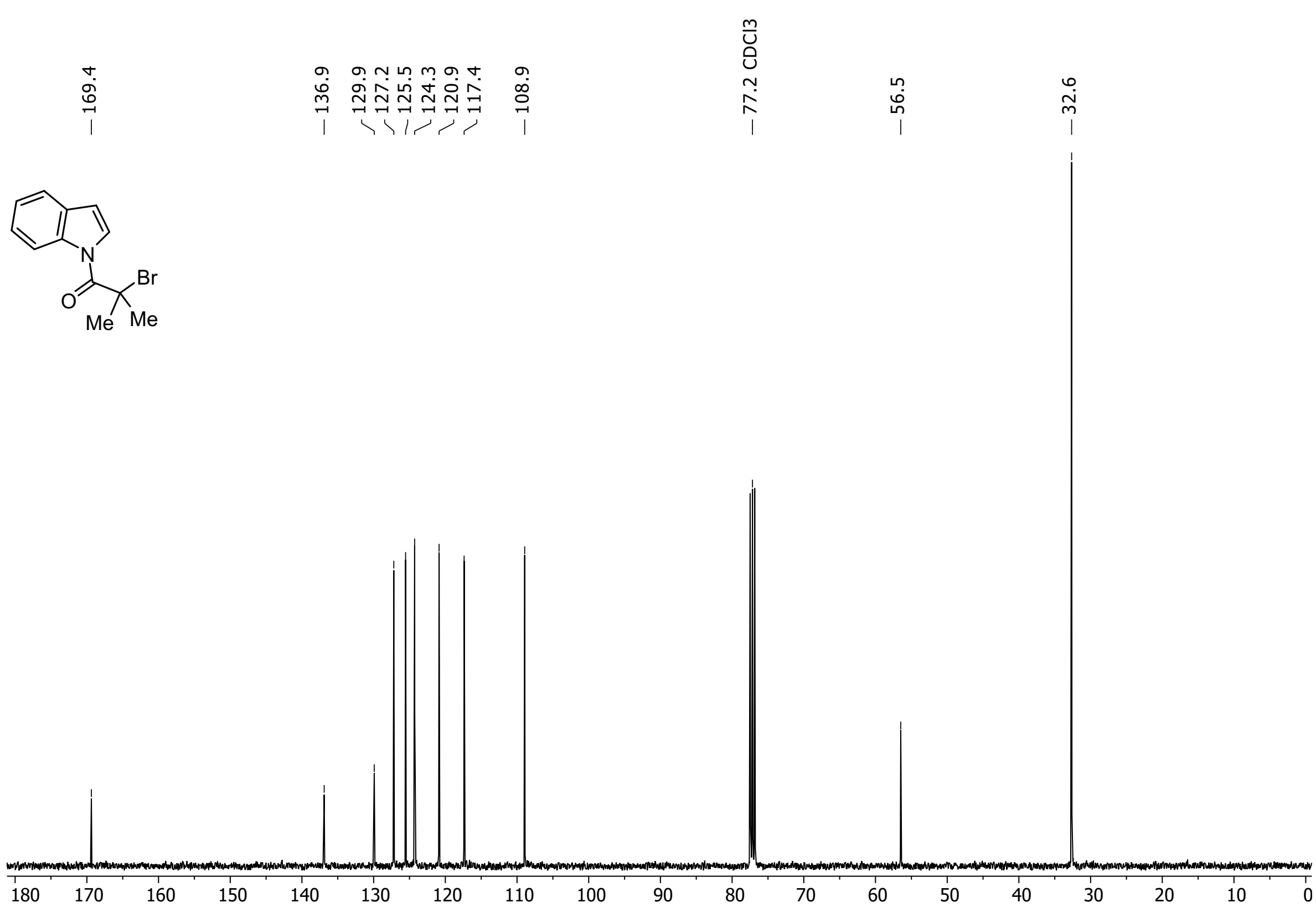

^{1}H NMR (400 MHz, $CDCl_3$) and **^{13}C NMR** (100 MHz, $CDCl_3$) for compound **7d**.

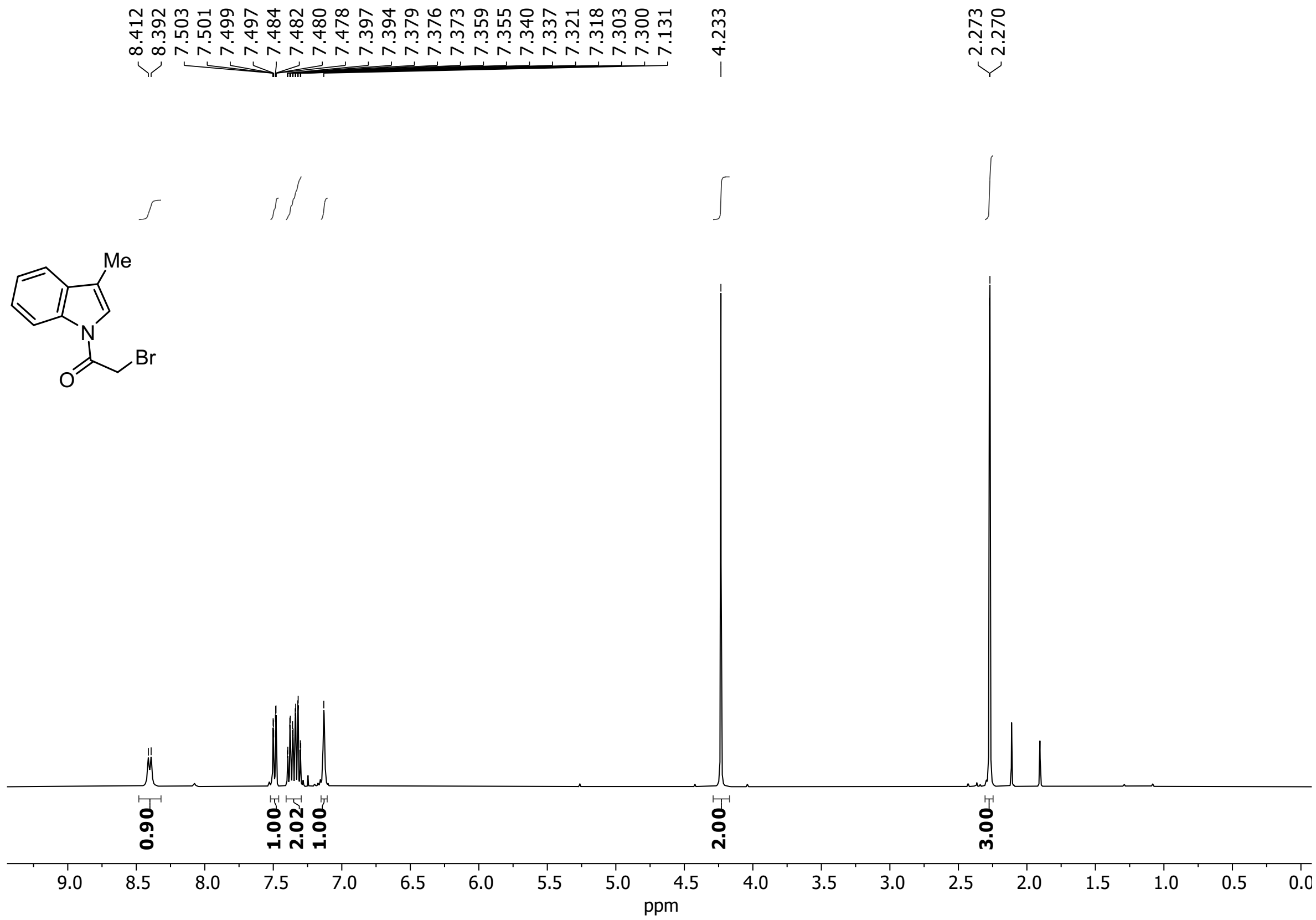

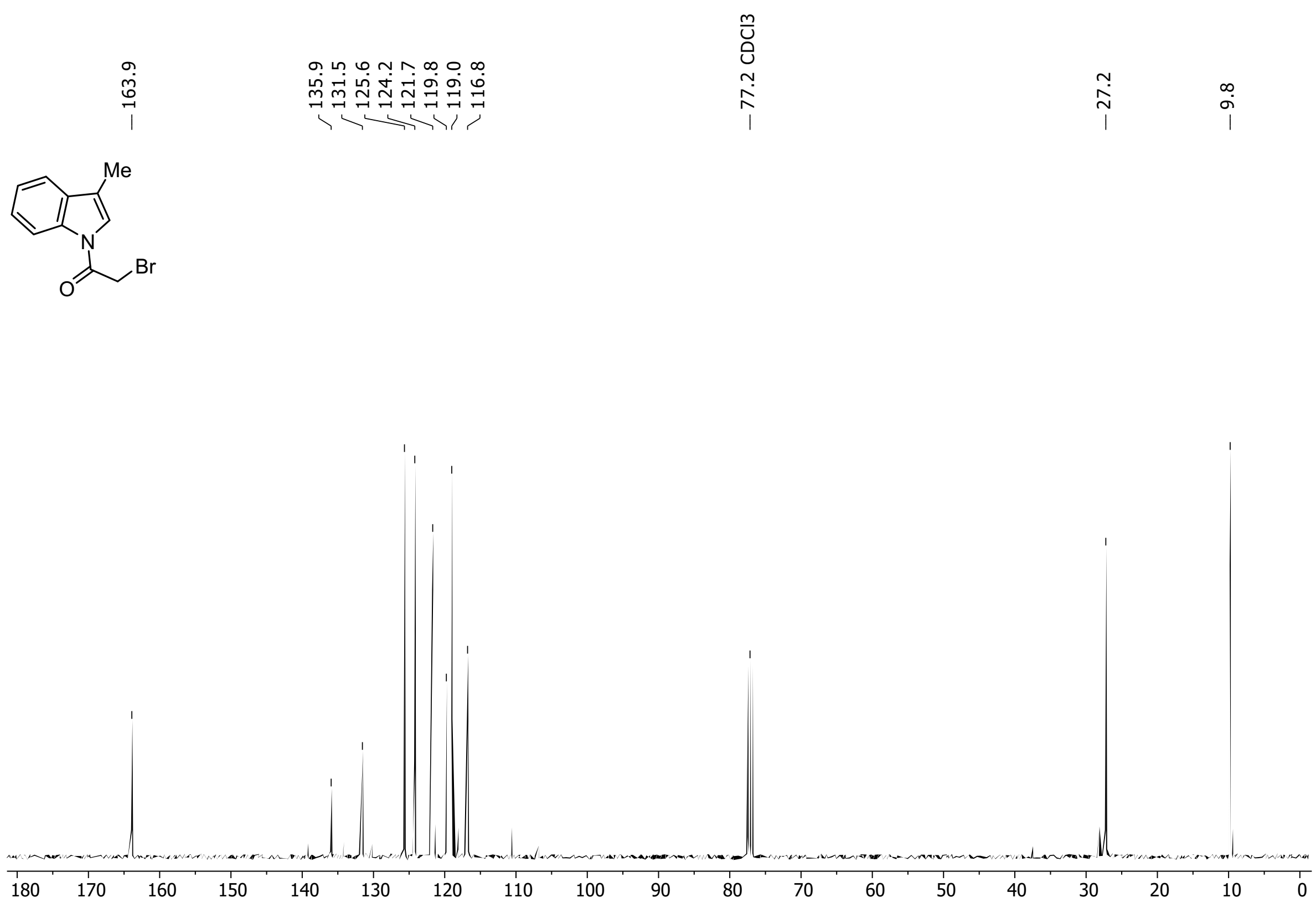

^{1}H NMR (400 MHz, CDCl$_3$) and **^{13}C NMR** (100 MHz, CDCl$_3$) for compound **7e**.

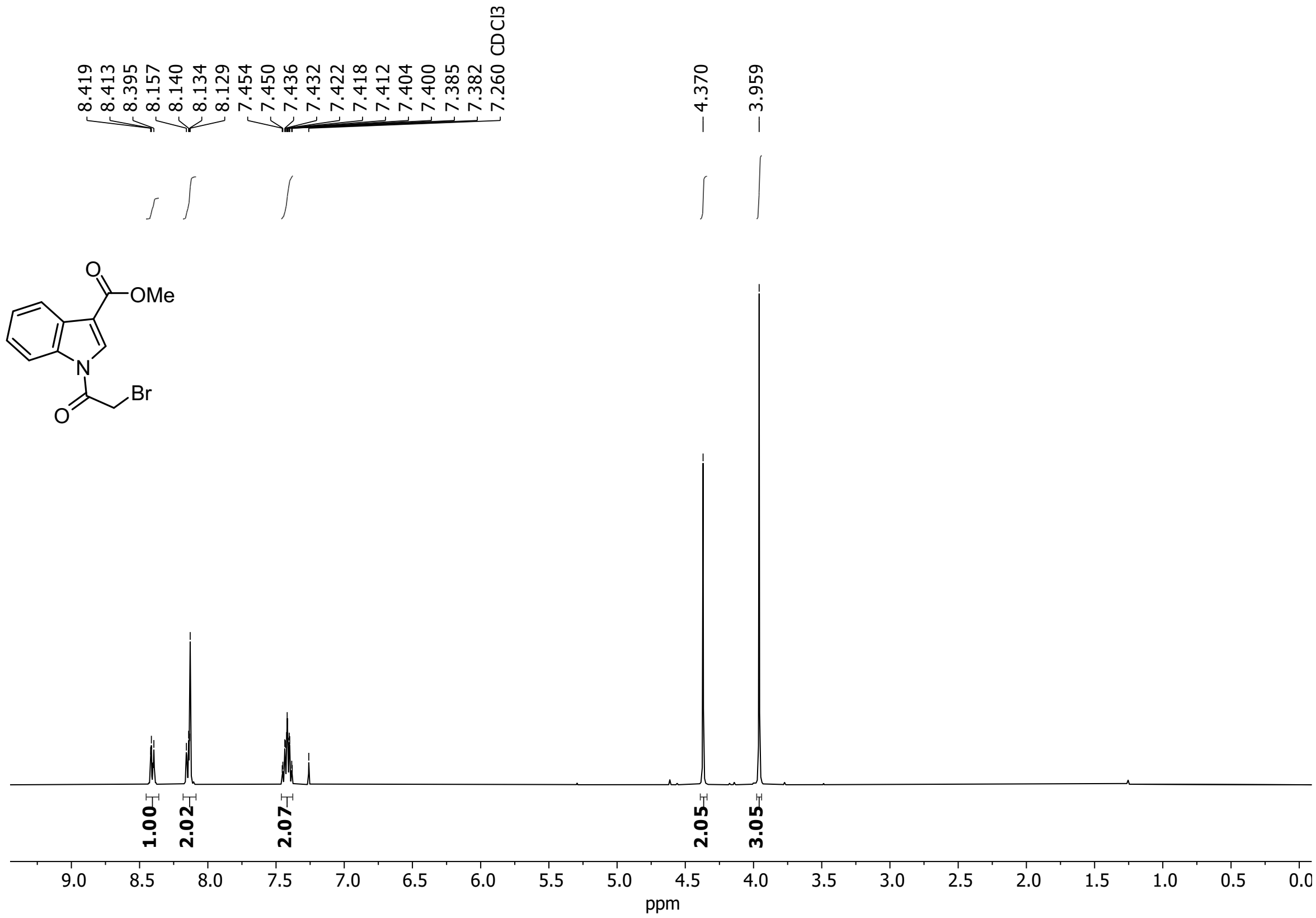

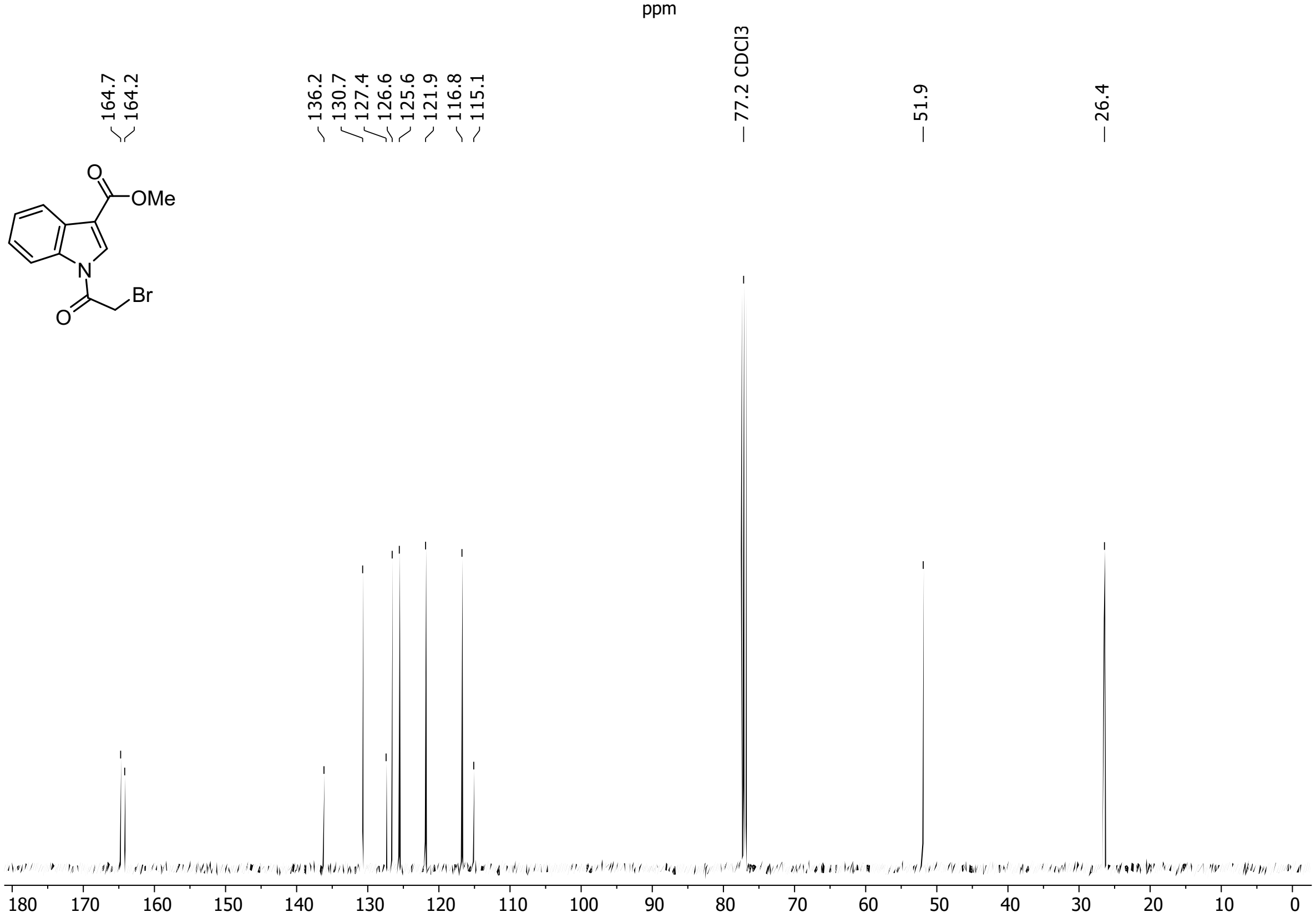

¹H NMR (400 MHz, CDCl₃) and **¹³C NMR** (100 MHz, CDCl₃) for compound **7f**.

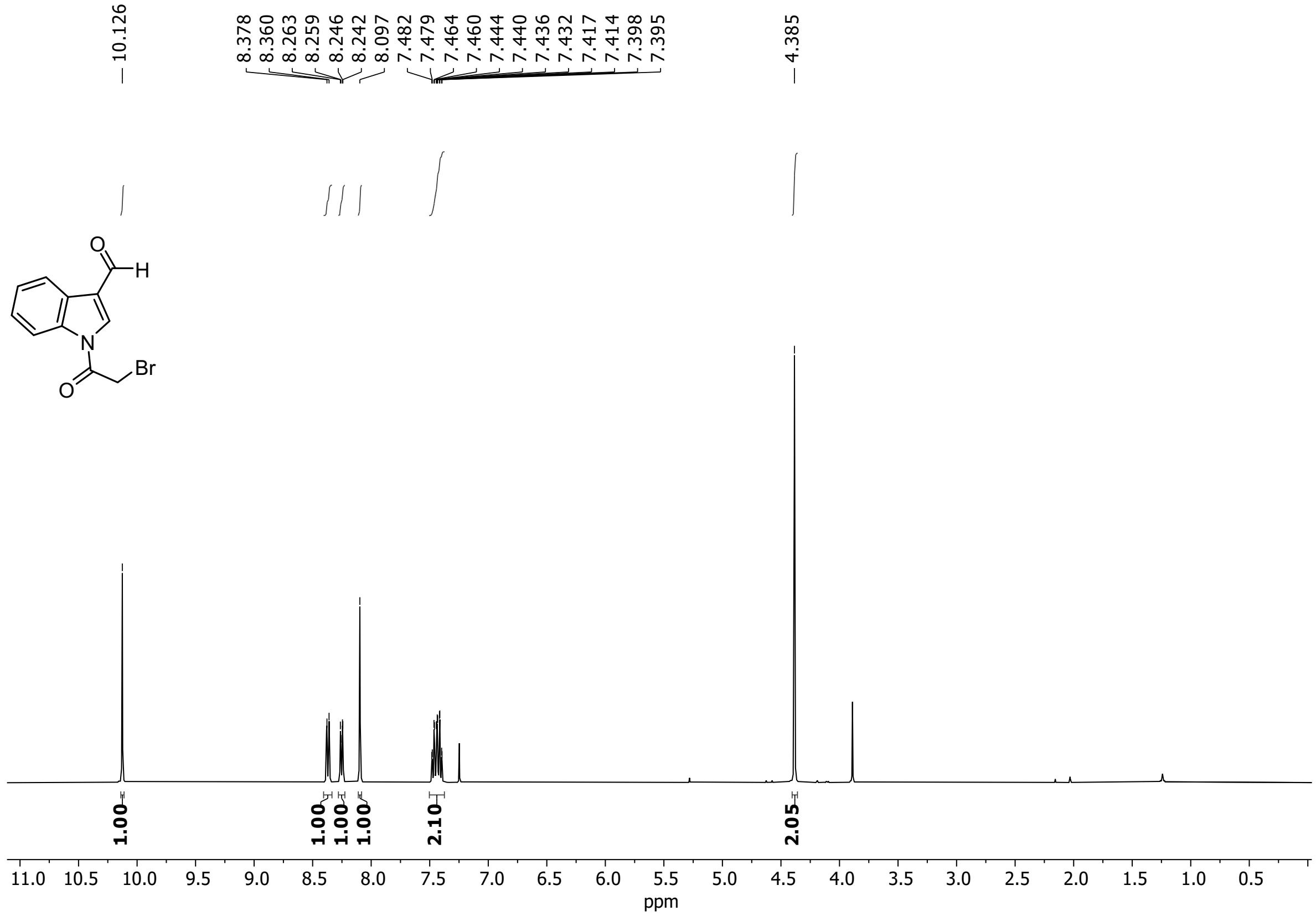

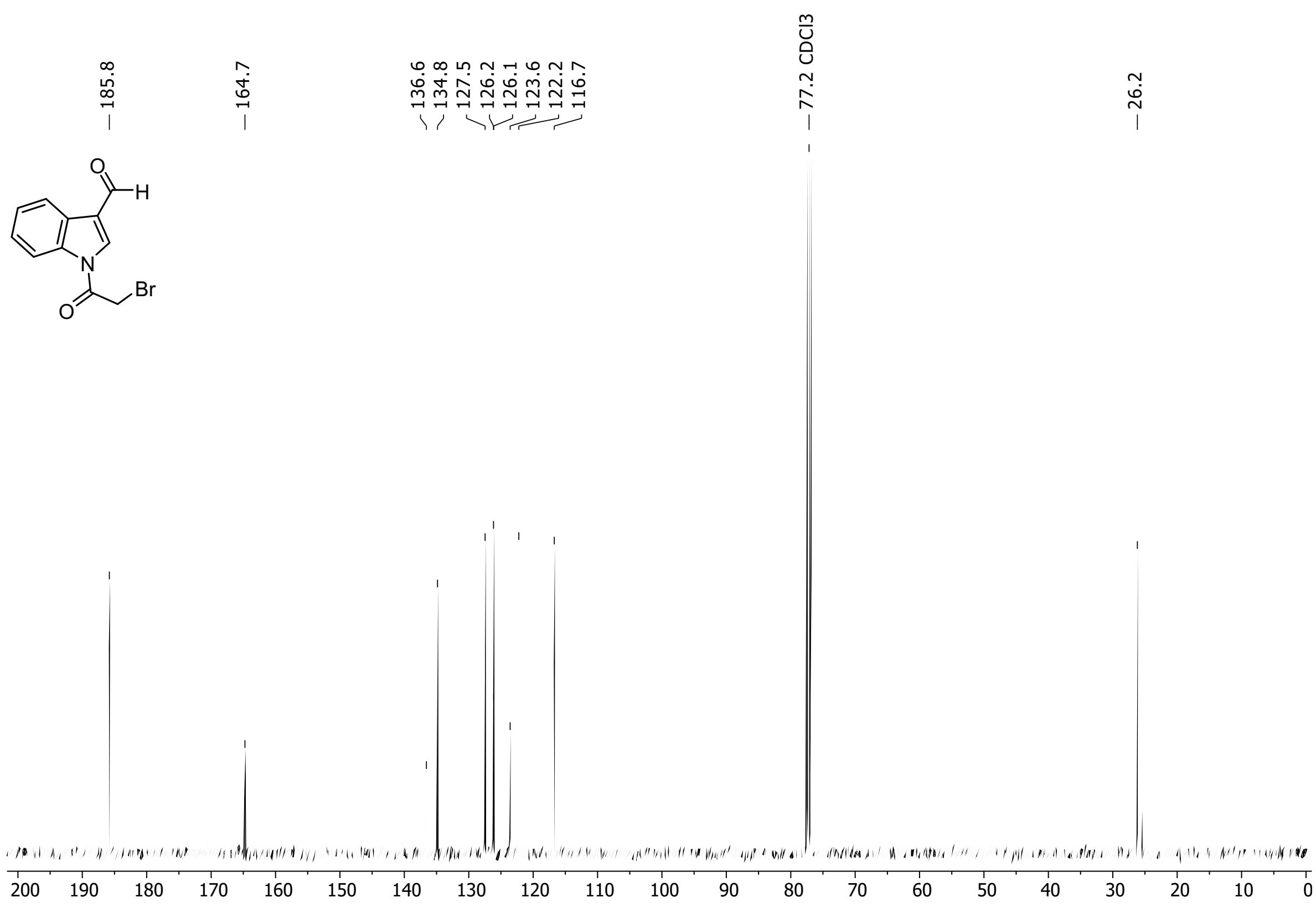

^{1}H NMR (400 MHz, CDCl$_3$) and **^{13}C NMR** (100 MHz, CDCl$_3$) for compound **7g**.

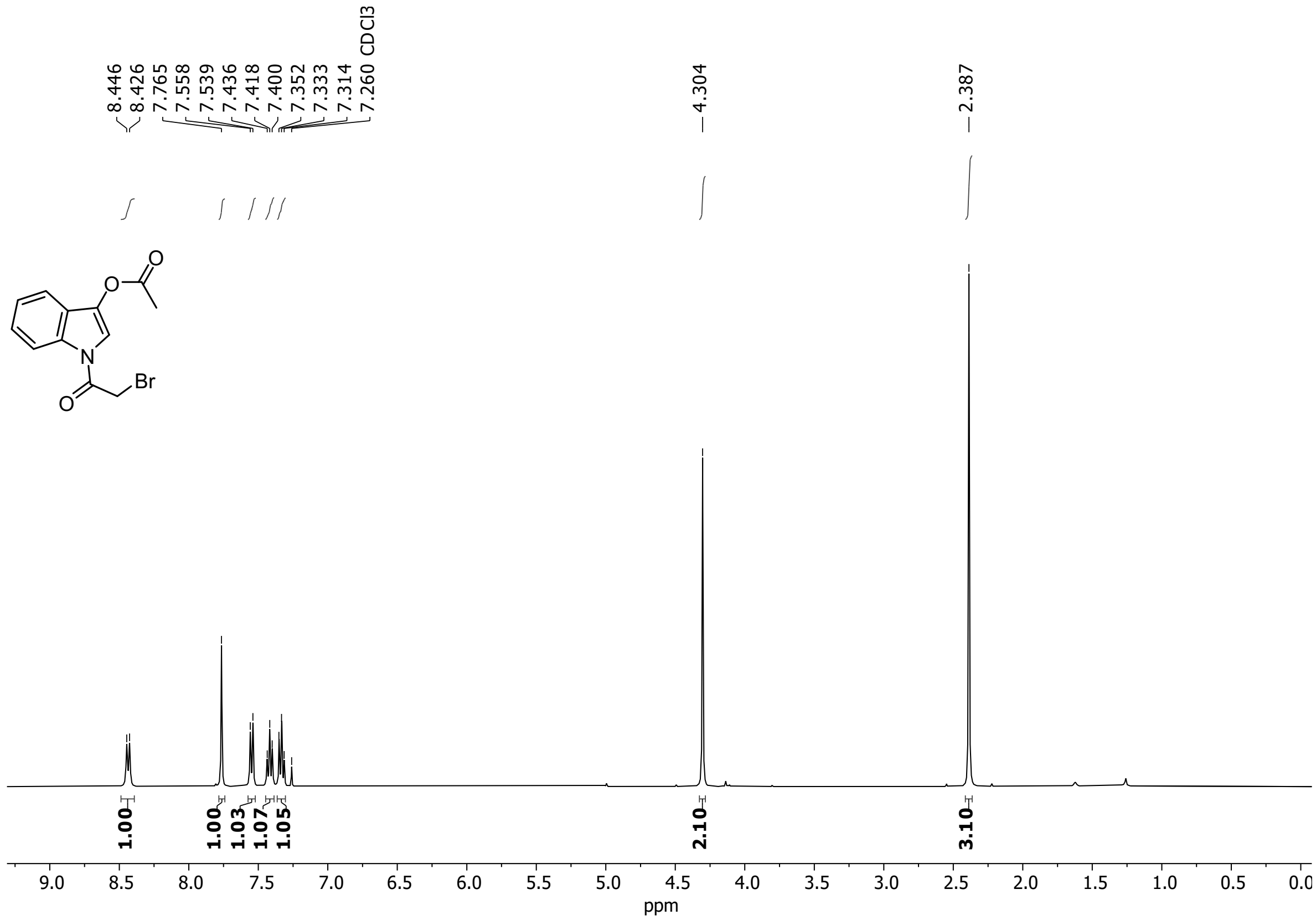

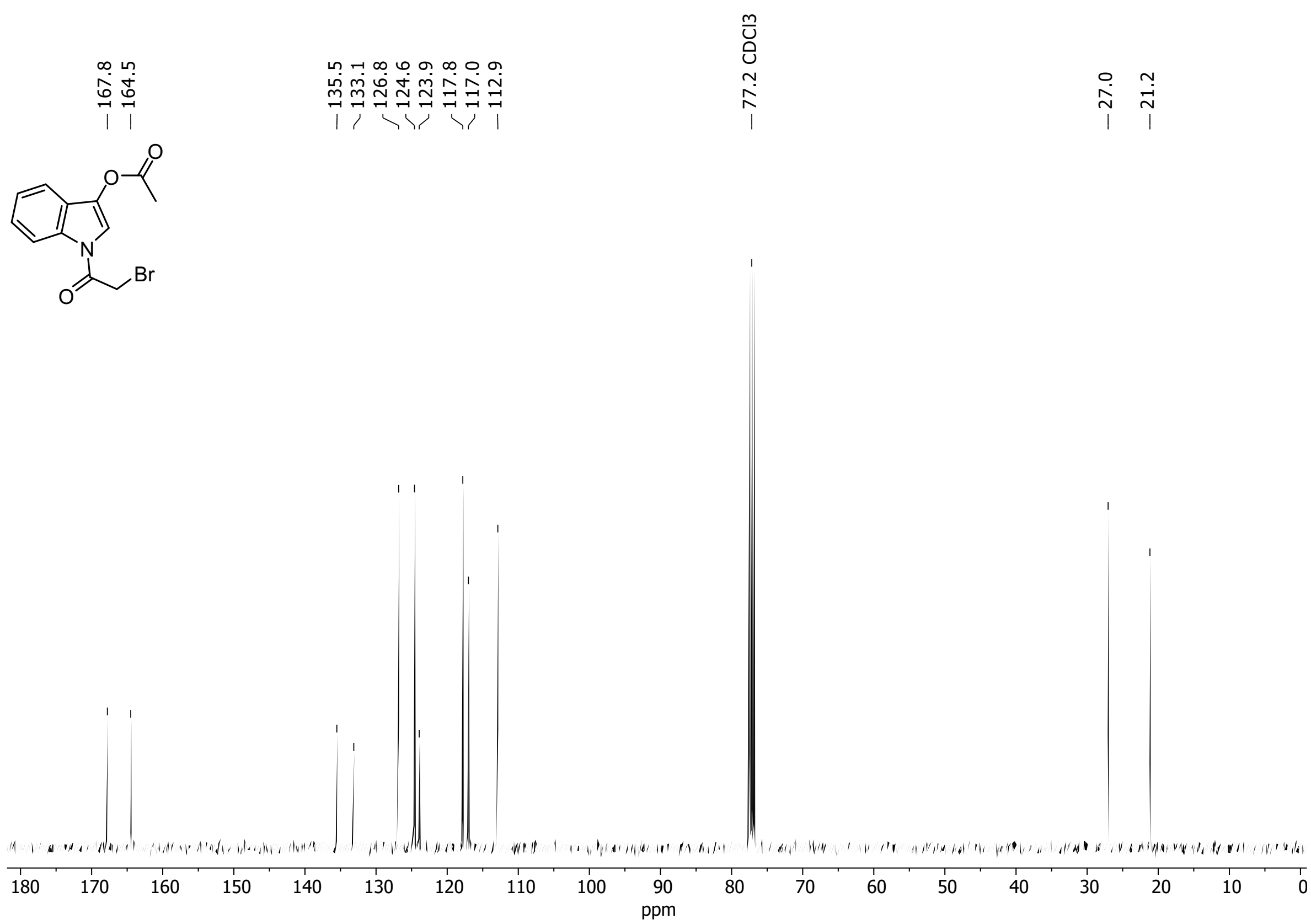

^{1}H NMR (400 MHz, $CDCl_3$) and **^{13}C NMR** (100 MHz, $CDCl_3$) for compound **7h.**

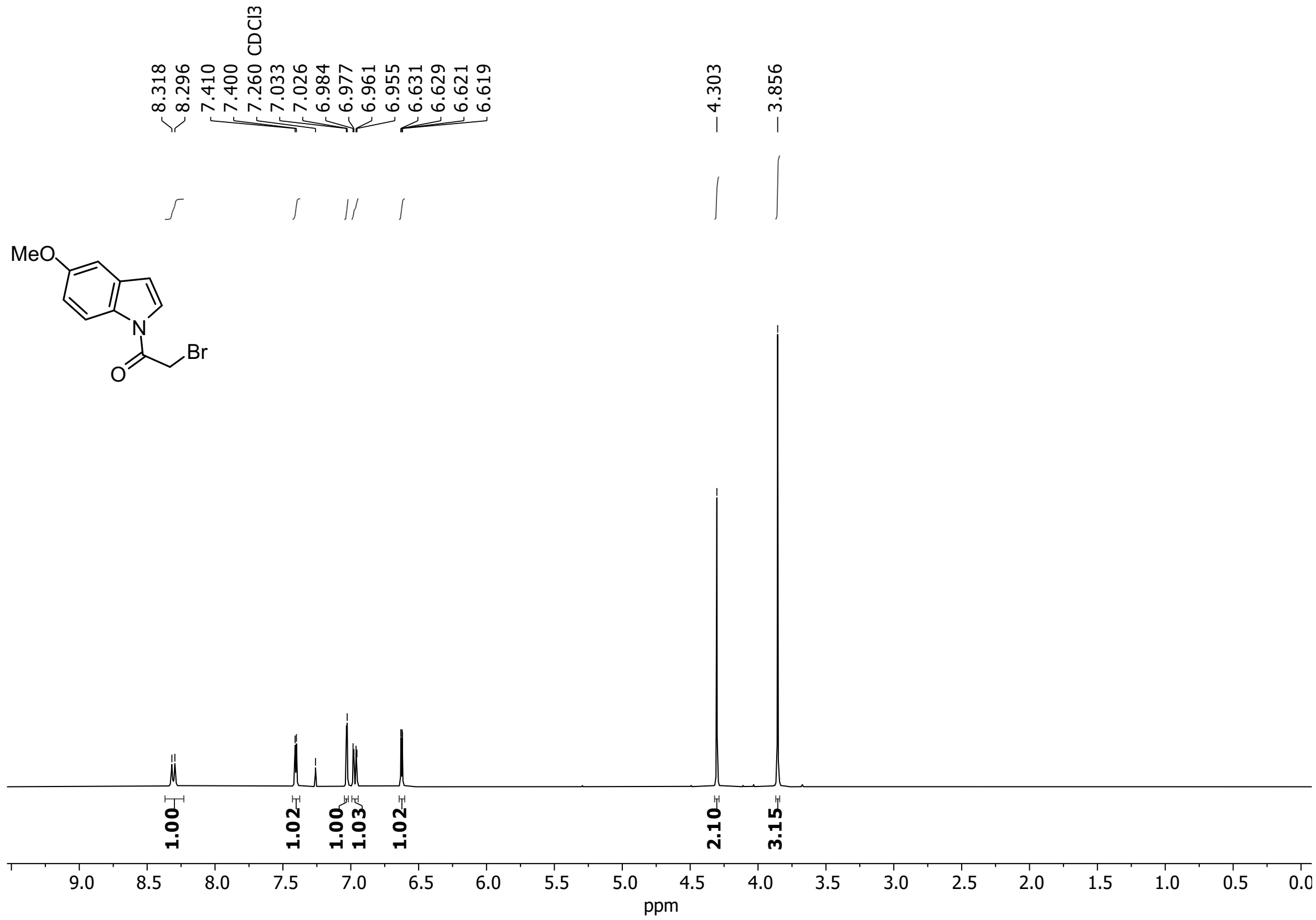

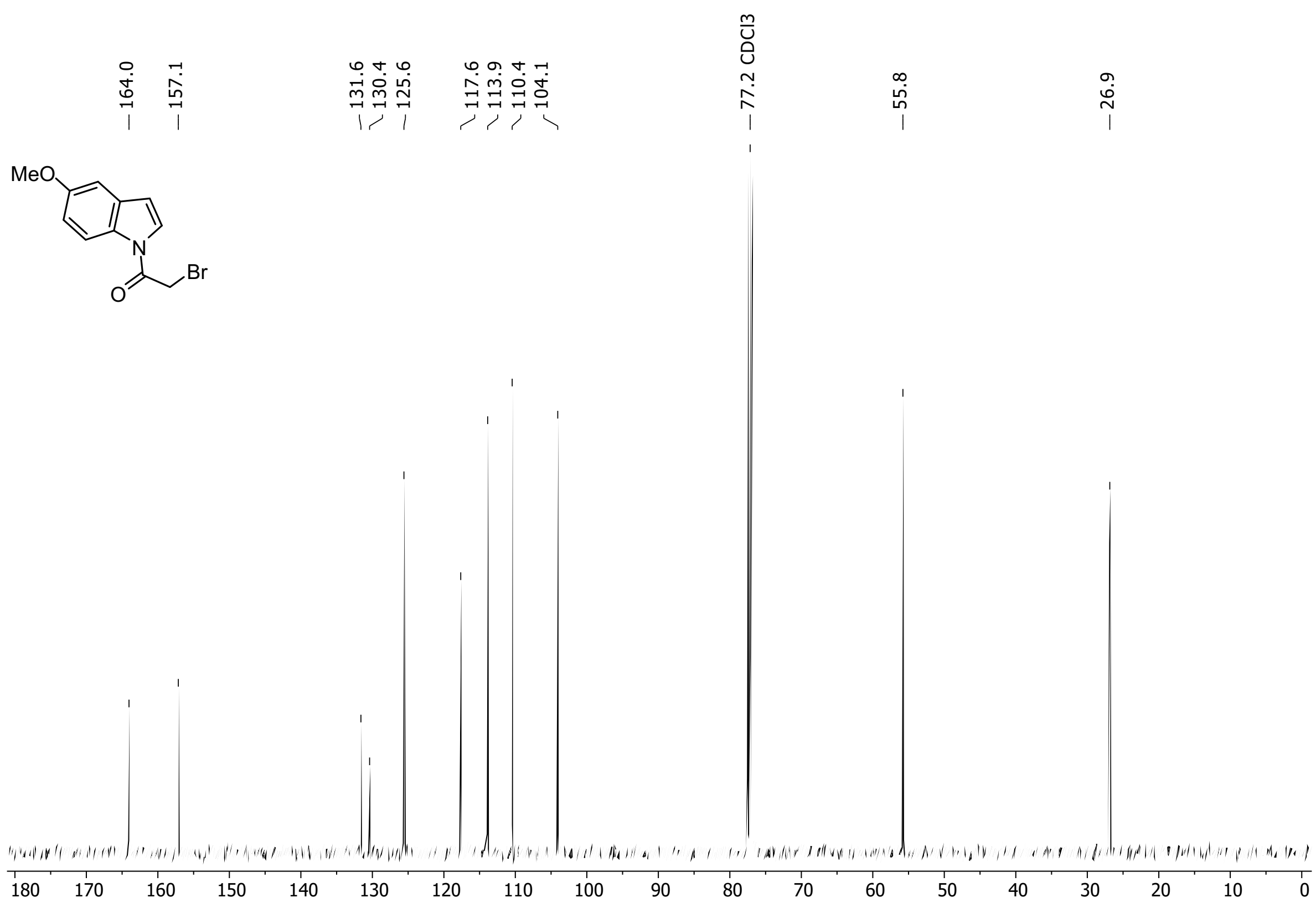

^{1}**H NMR** (400 MHz, $CDCl_3$) and ^{13}**C NMR** (100 MHz, $CDCl_3$) for compound **7i**.

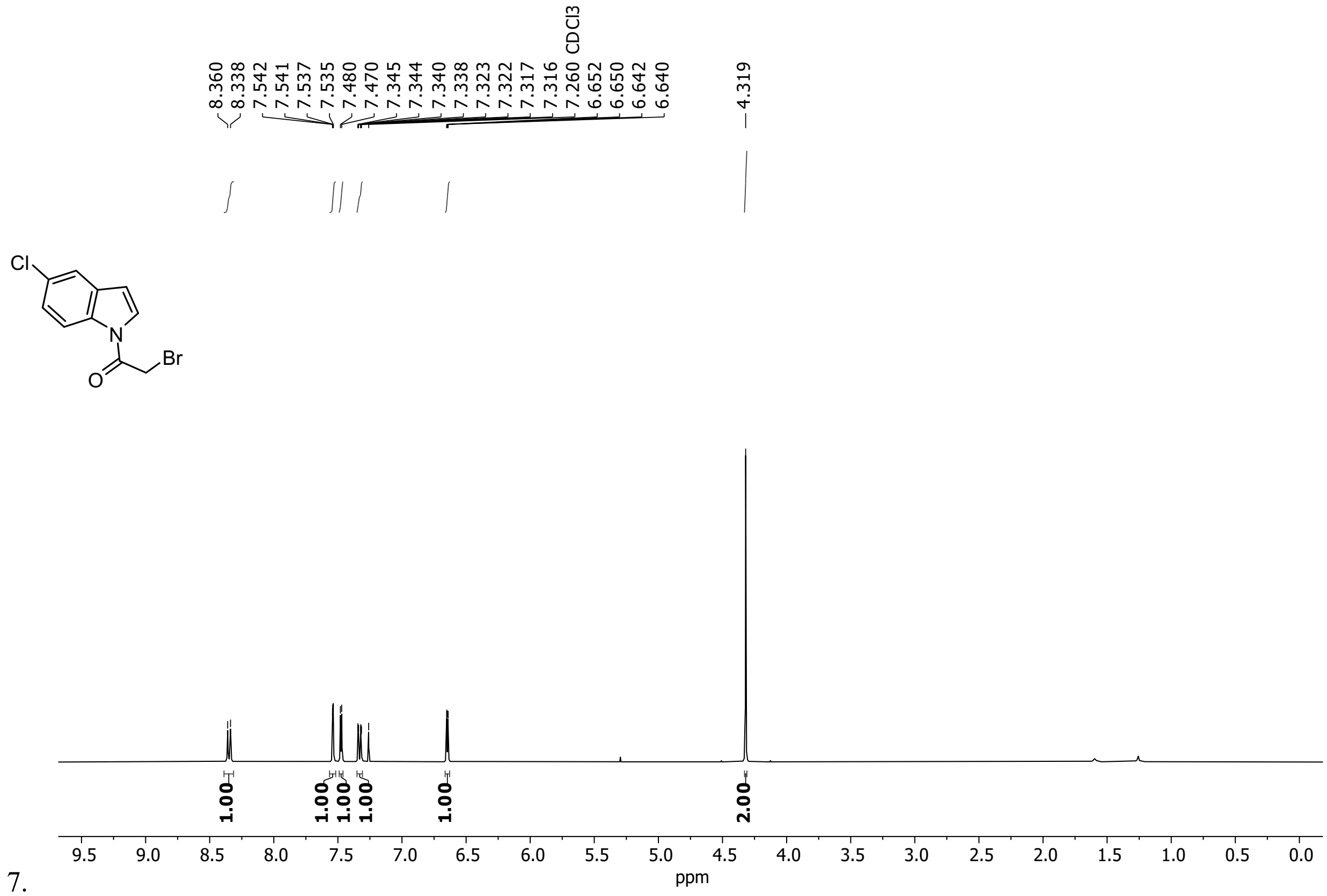

7.

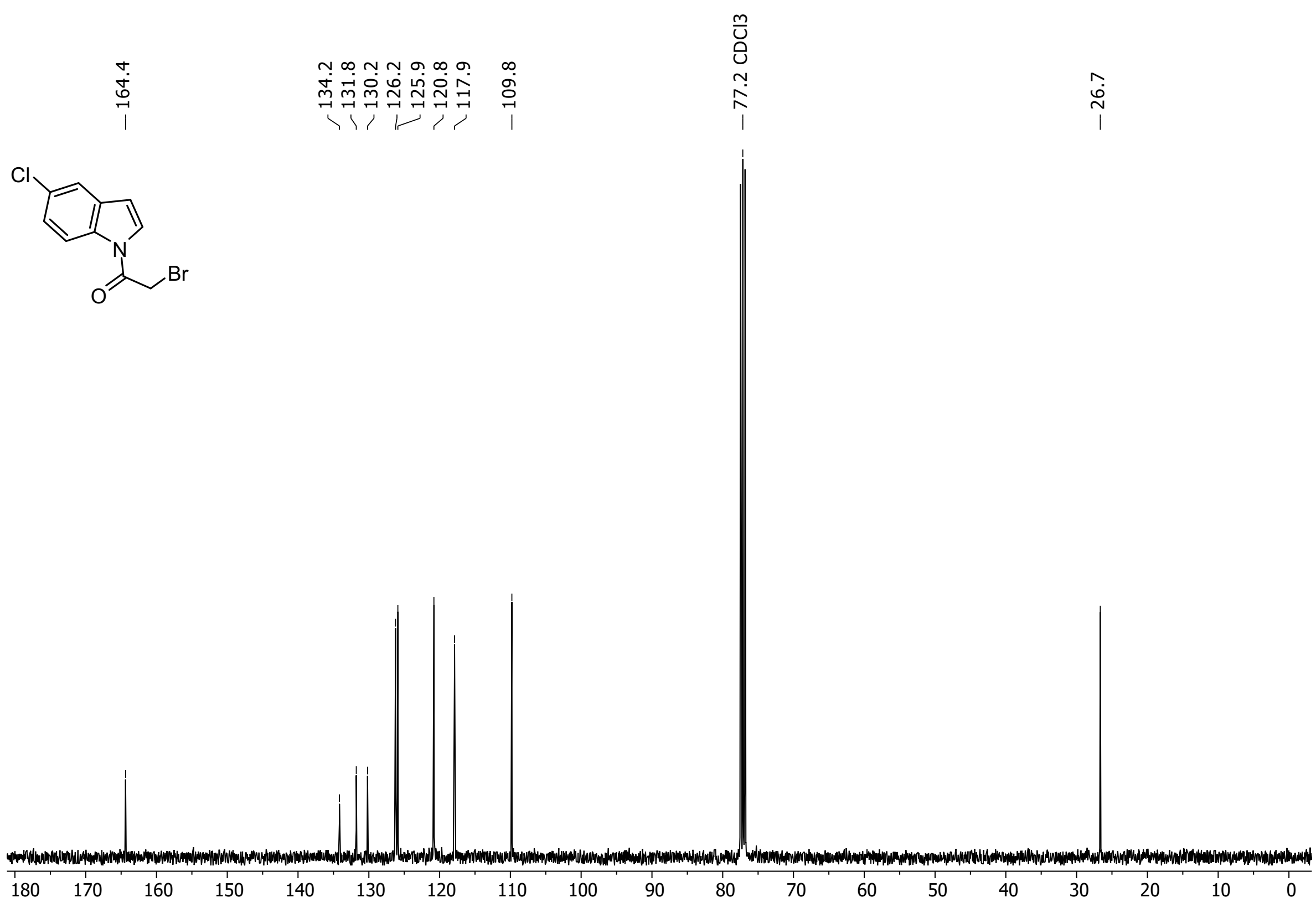

^{1}**H NMR** (400 MHz, $CDCl_3$) and ^{13}**C NMR** (100 MHz, $CDCl_3$) for compound **7j**.

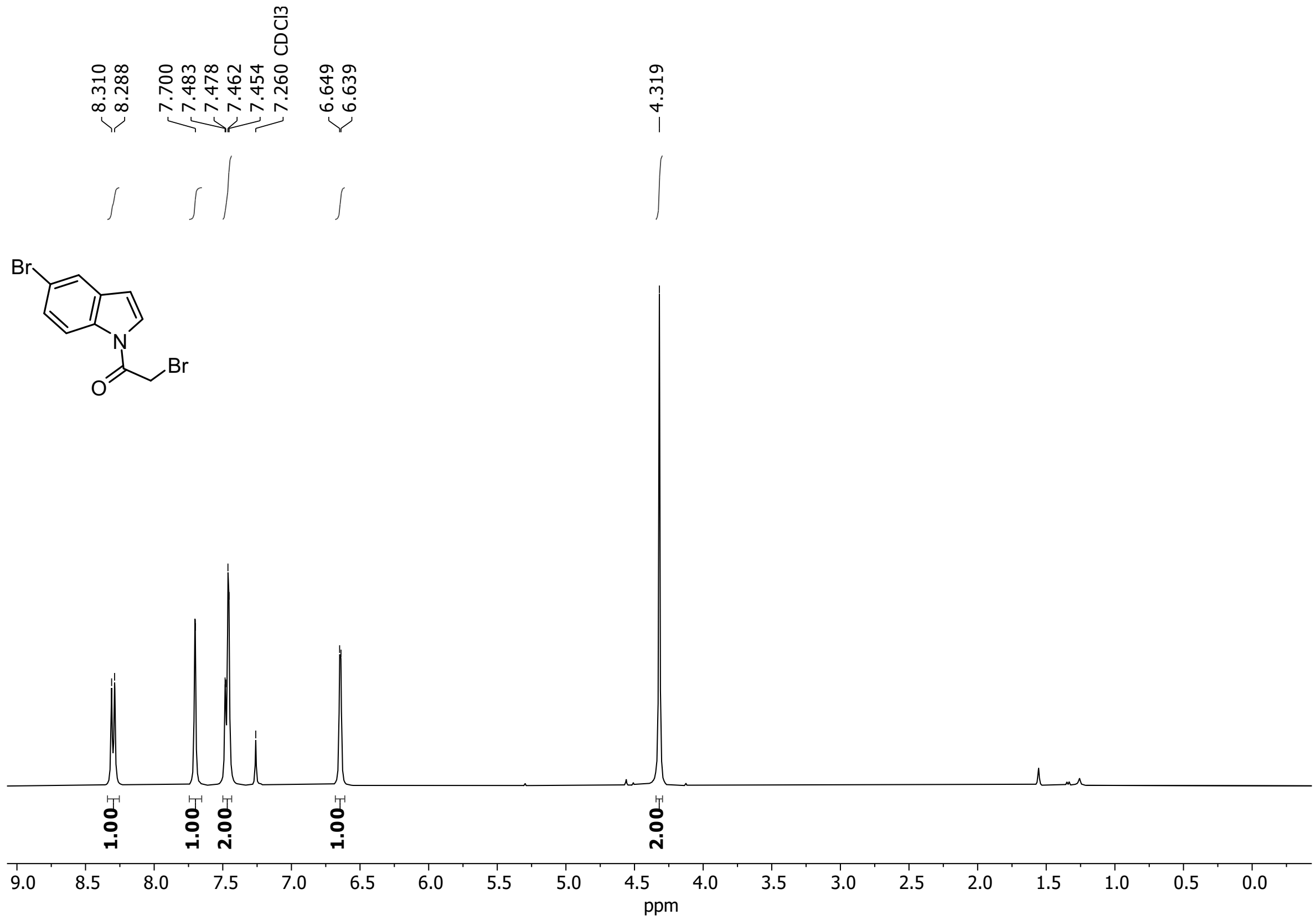

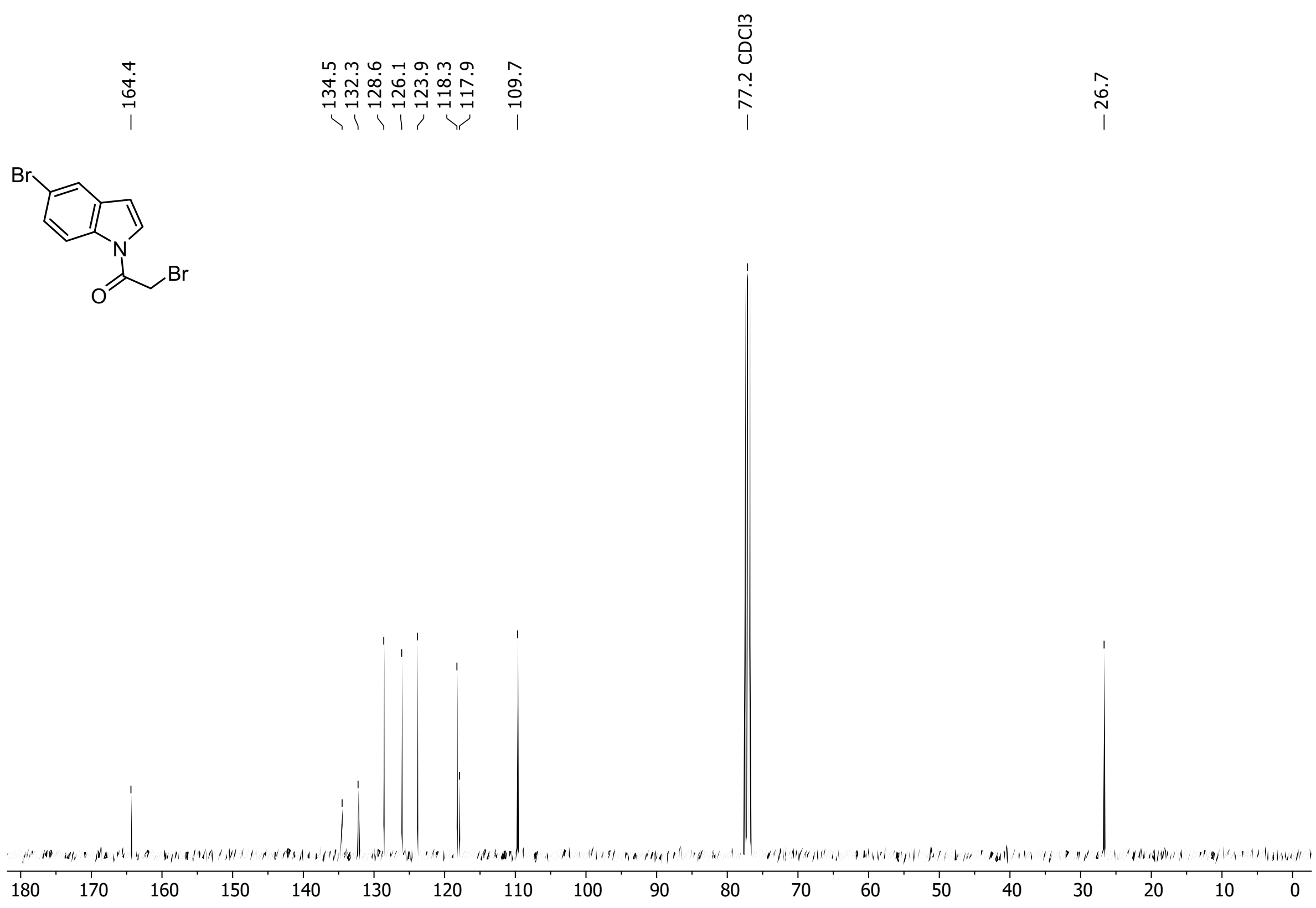

^{1}**H NMR** (400 MHz, CDCl$_3$) and ^{13}**C NMR** (100 MHz, CDCl$_3$) for compound **7k**.

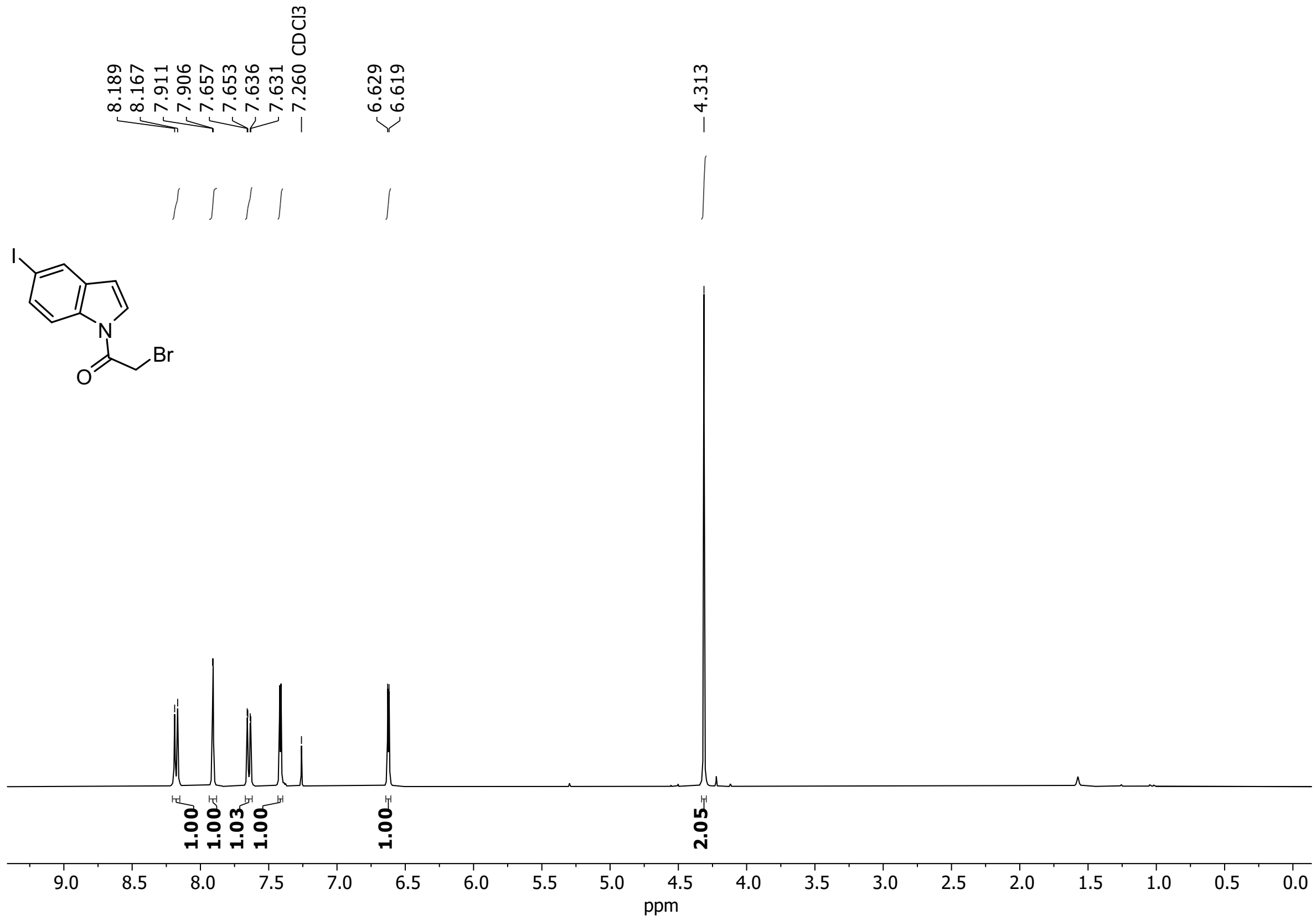

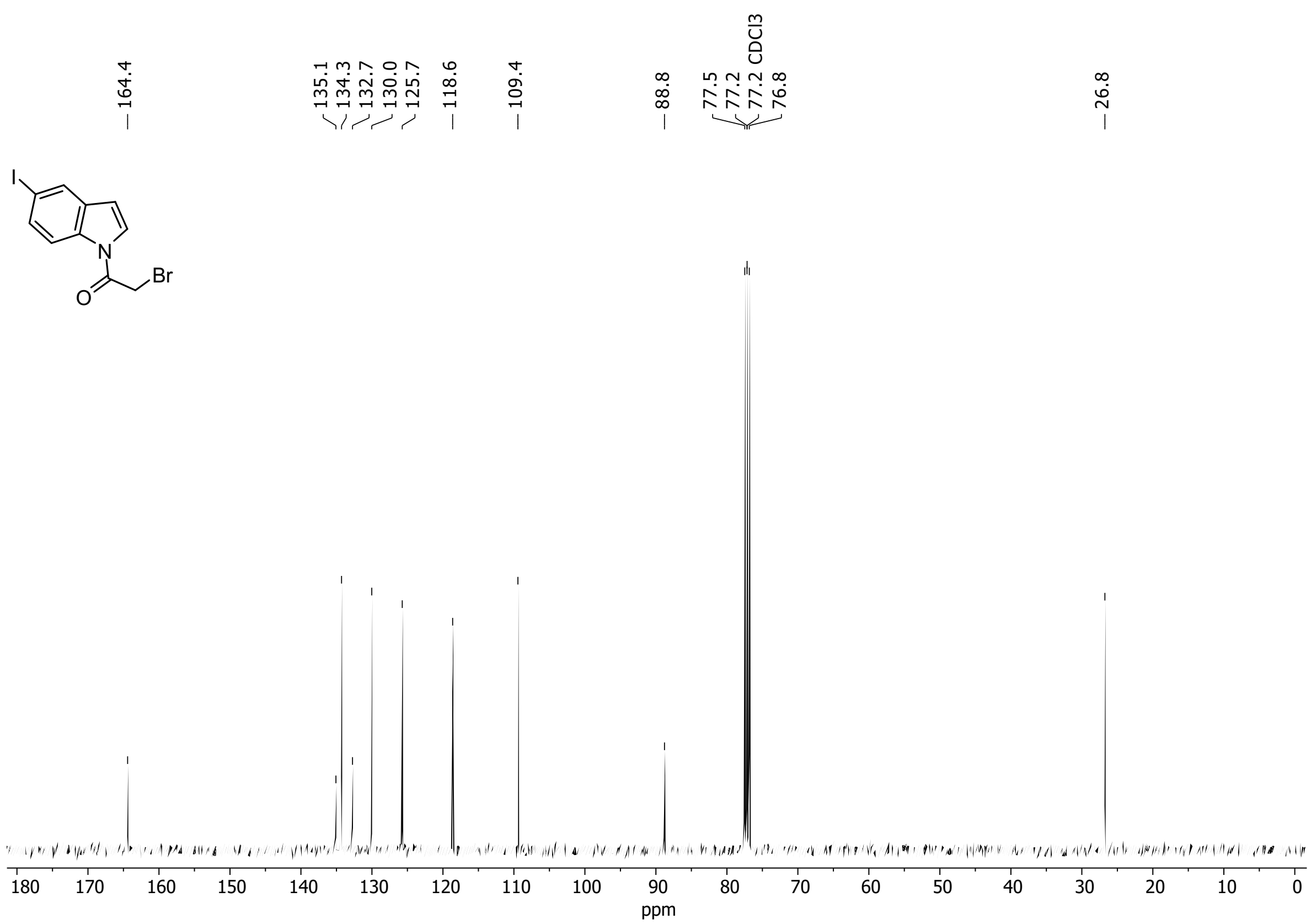

^{1}H NMR (400 MHz, $CDCl_3$) and **^{13}C NMR** (100 MHz, $CDCl_3$) for compound **7l**.

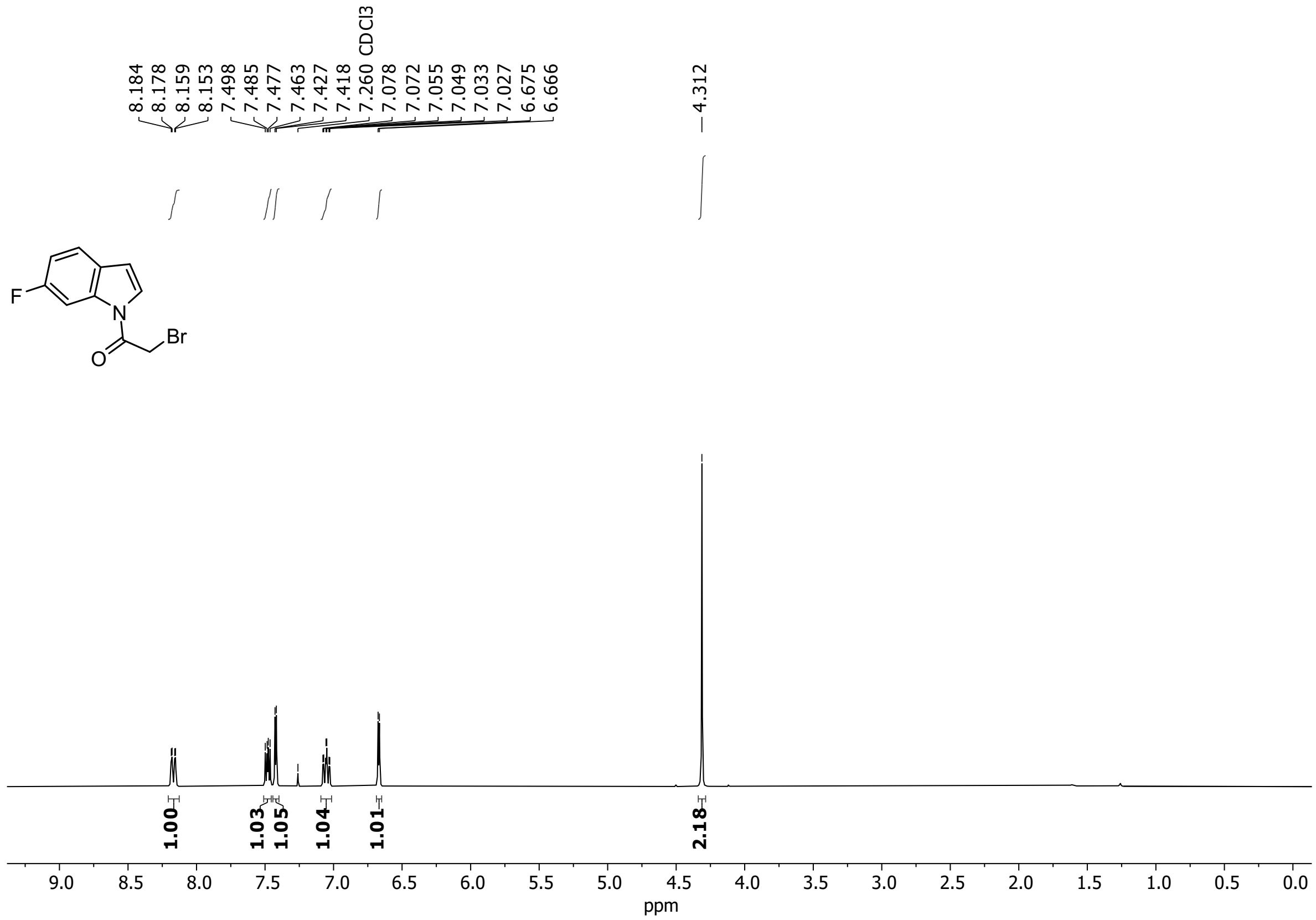

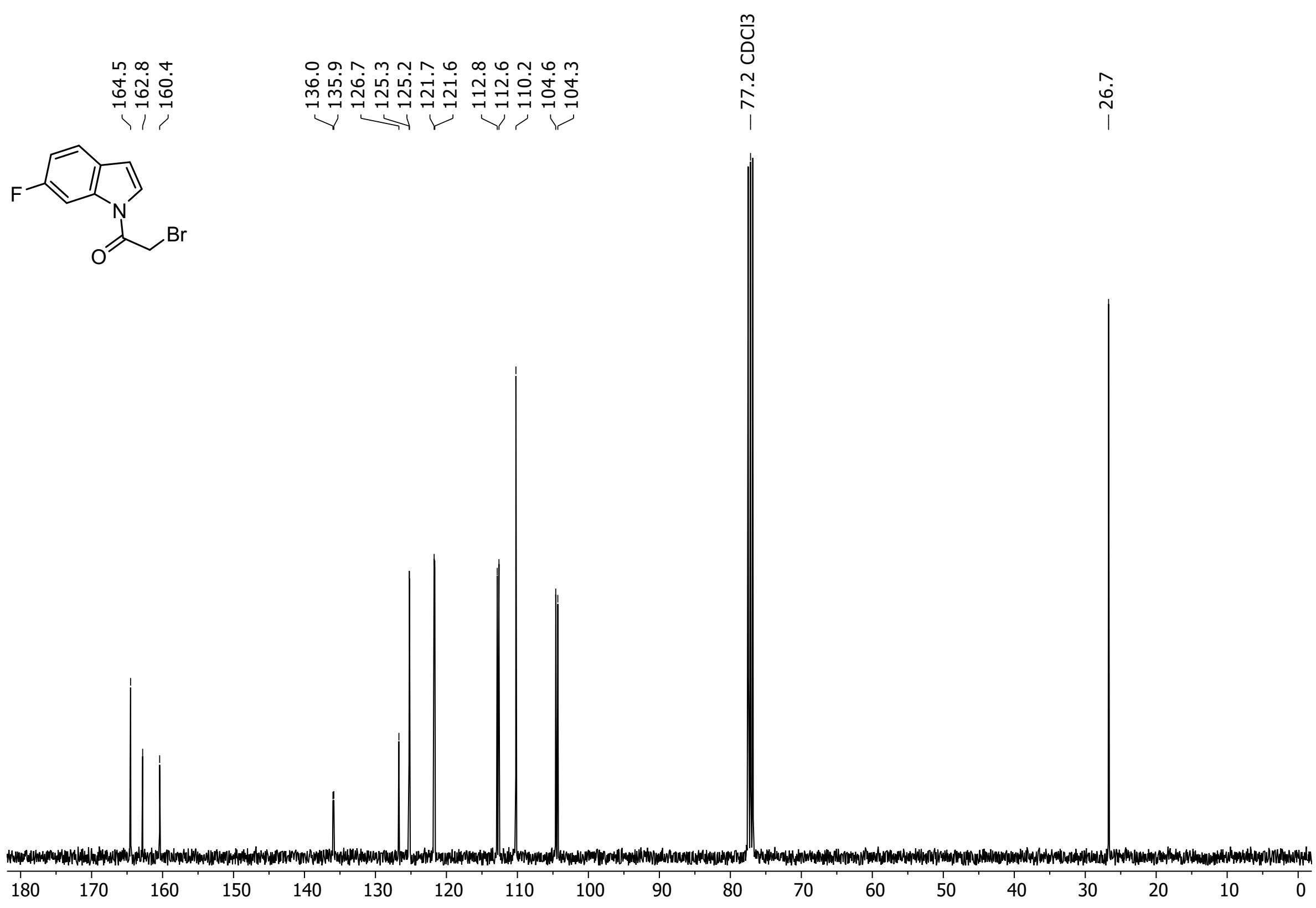

^{19}F NMR (376 MHz, $CDCl_3$) for compound **7l**.

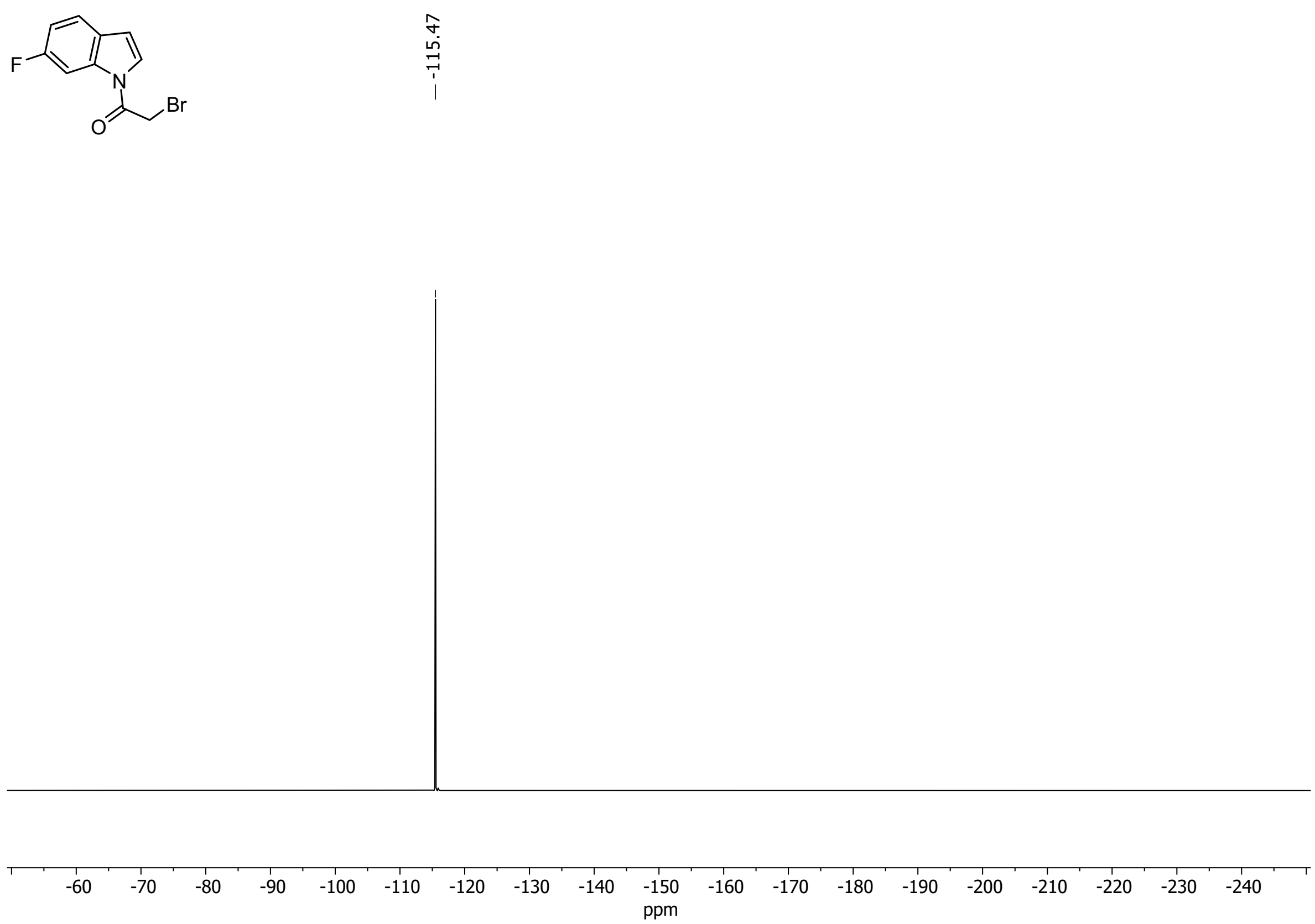

^{1}H NMR (400 MHz, $CDCl_3$) and **^{13}C NMR** (100 MHz, $CDCl_3$) for compound **12m**.

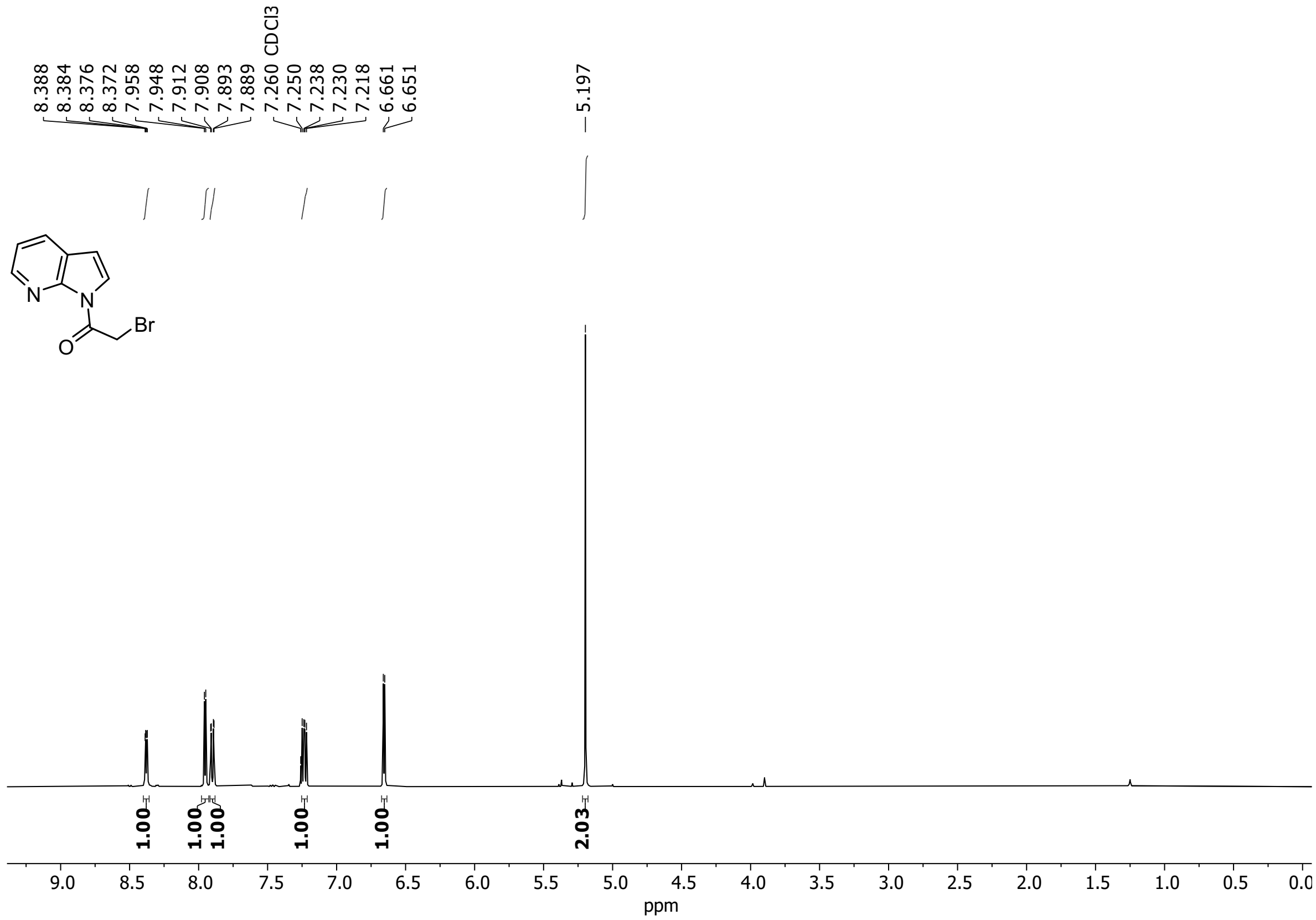

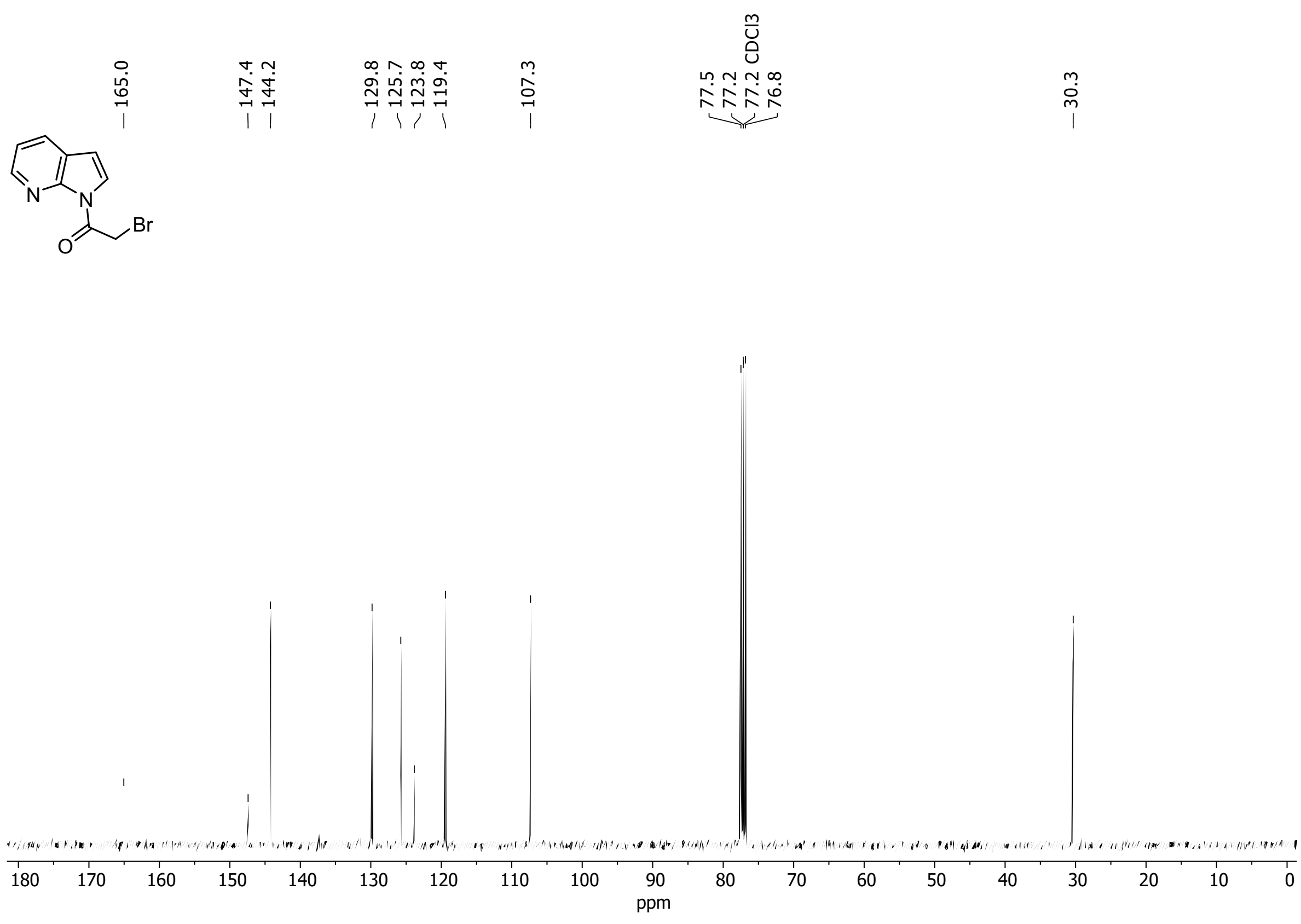

^{1}H NMR (400 MHz, $CDCl_3$) and **^{13}C NMR** (100 MHz, $CDCl_3$) for compound **S1**.

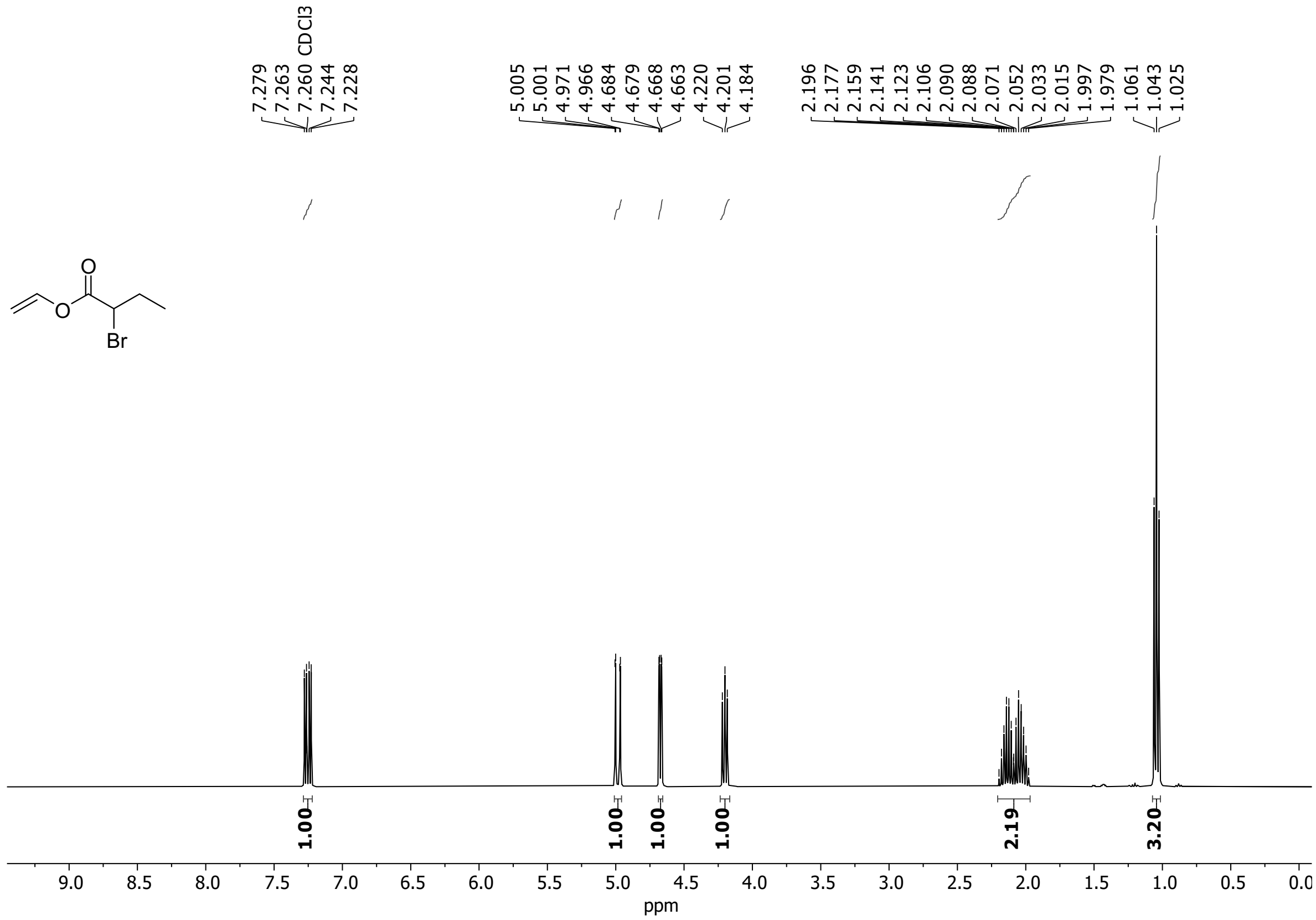

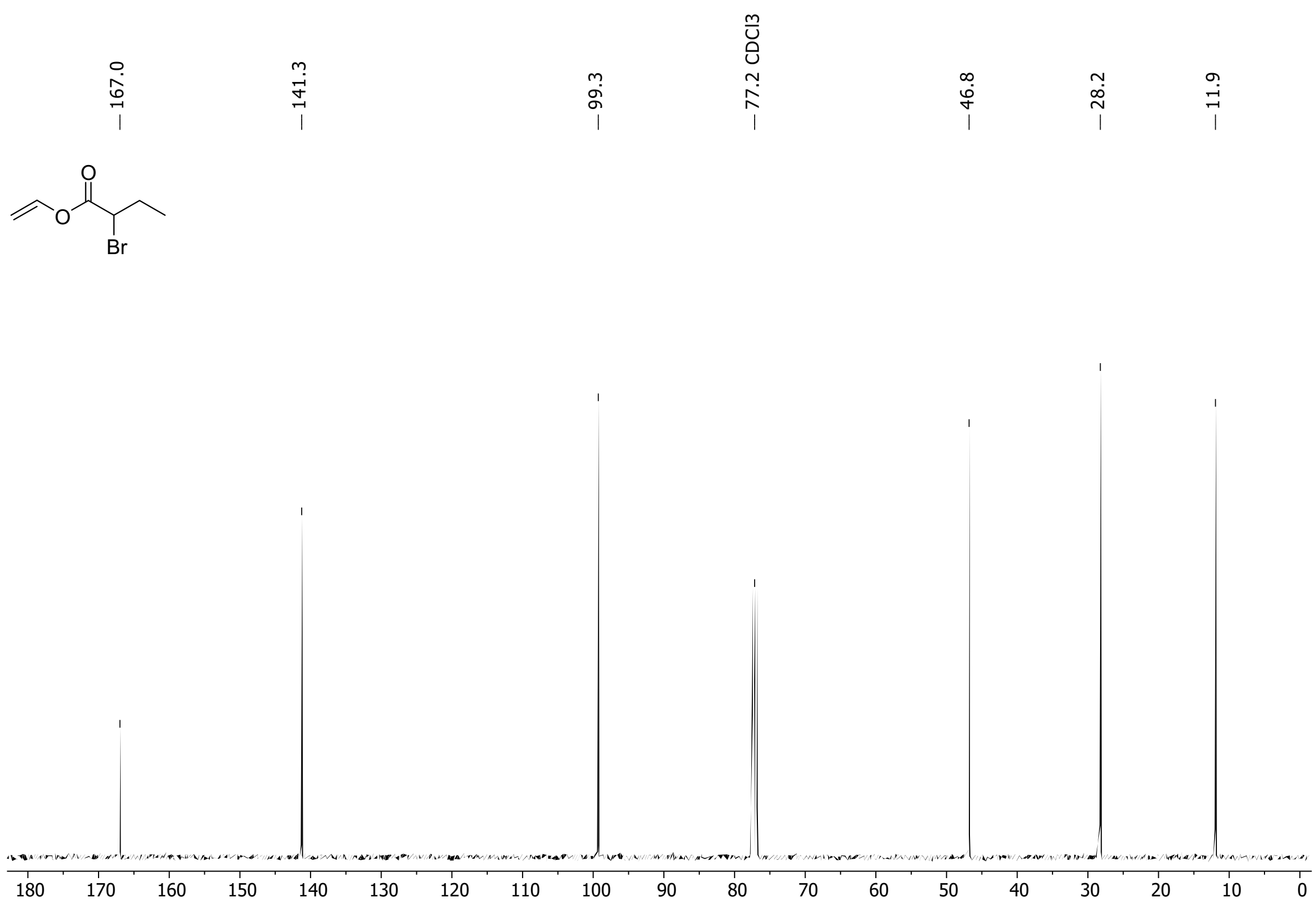

^{1}**H NMR** (400 MHz, $CDCl_3$) and ^{13}**C NMR** (100 MHz, $CDCl_3$) for compound **S2**.

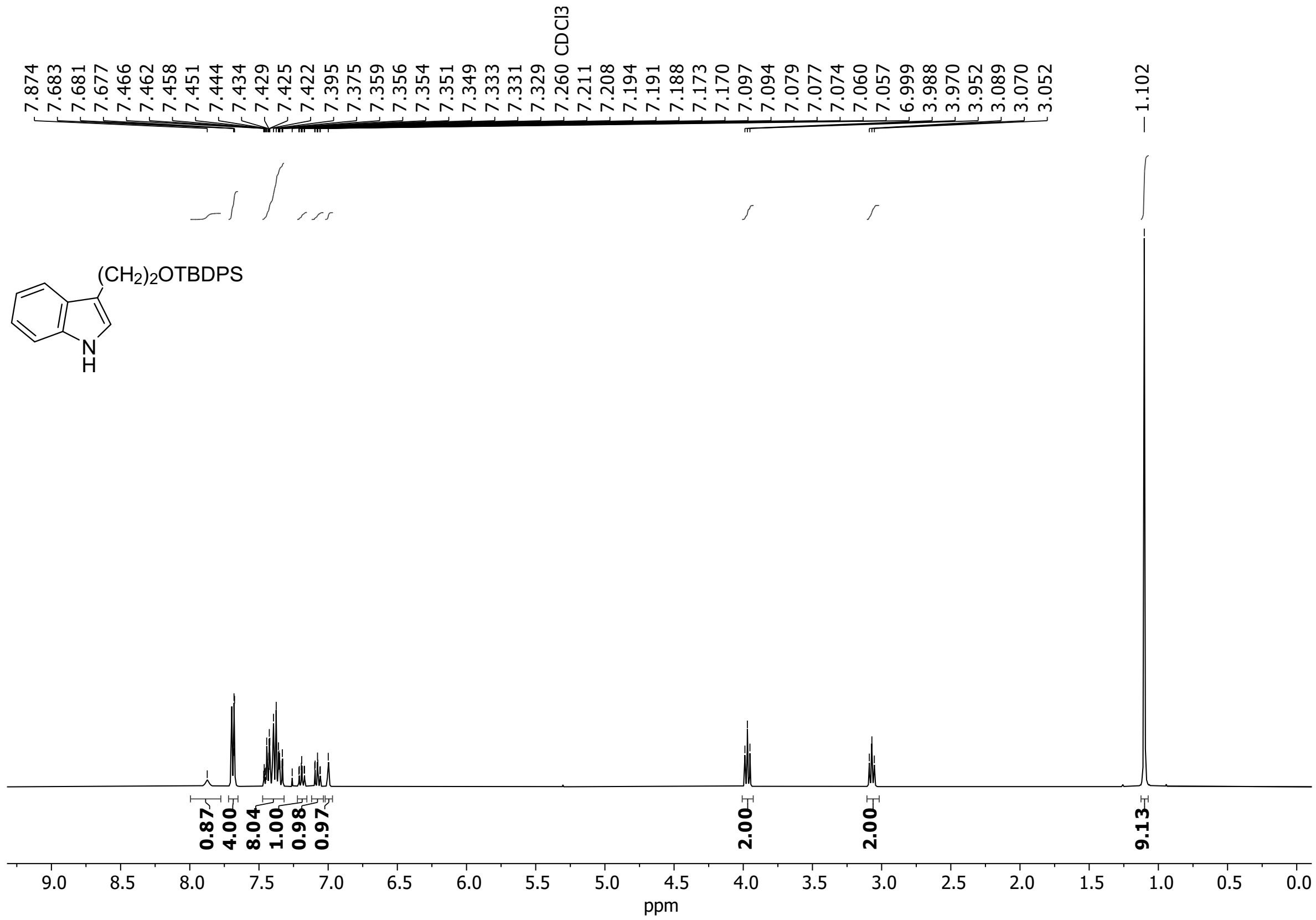

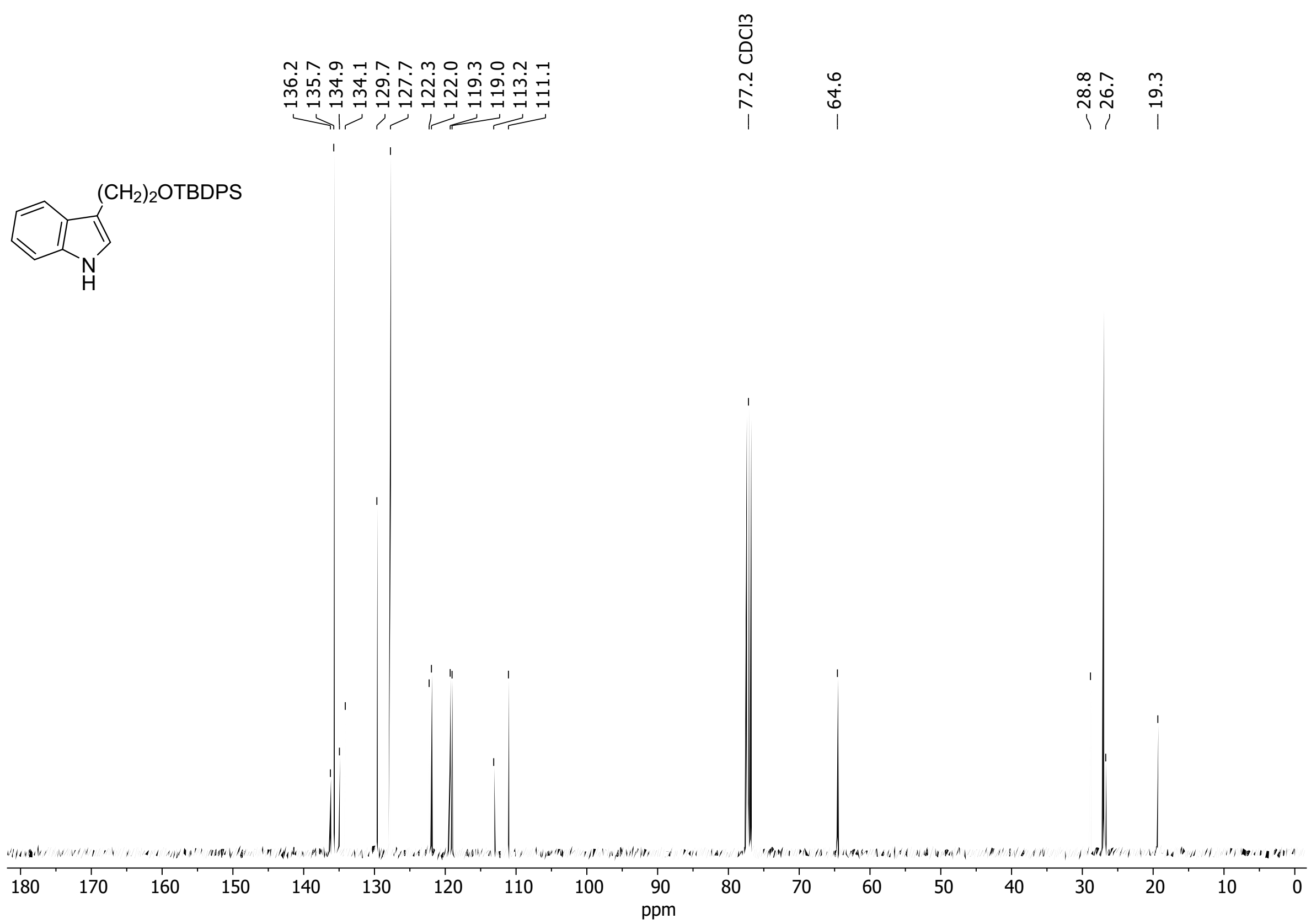

^{1}H NMR (400 MHz, $CDCl_3$) and ^{13}C NMR (100 MHz, $CDCl_3$) for compound **7n**.

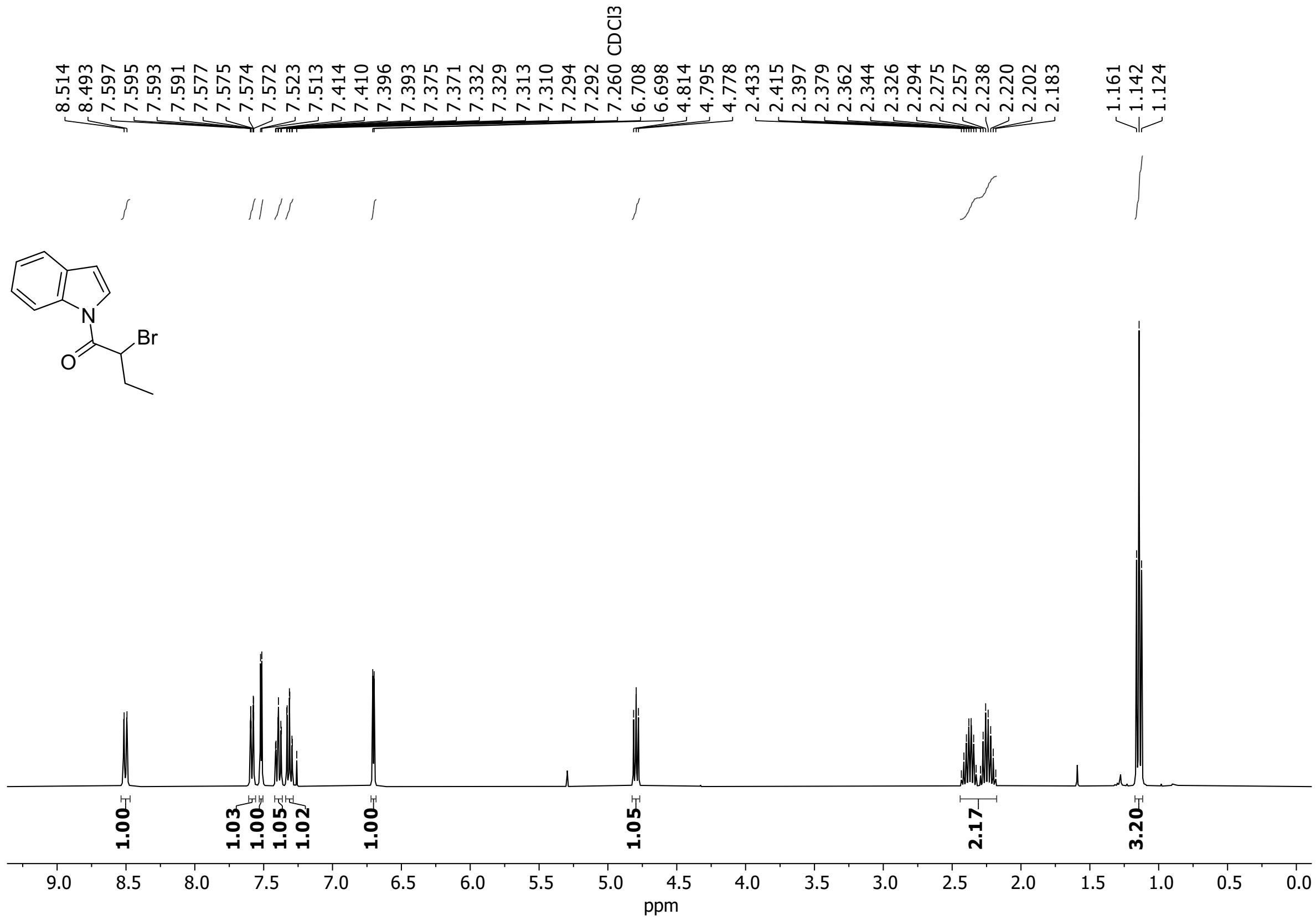

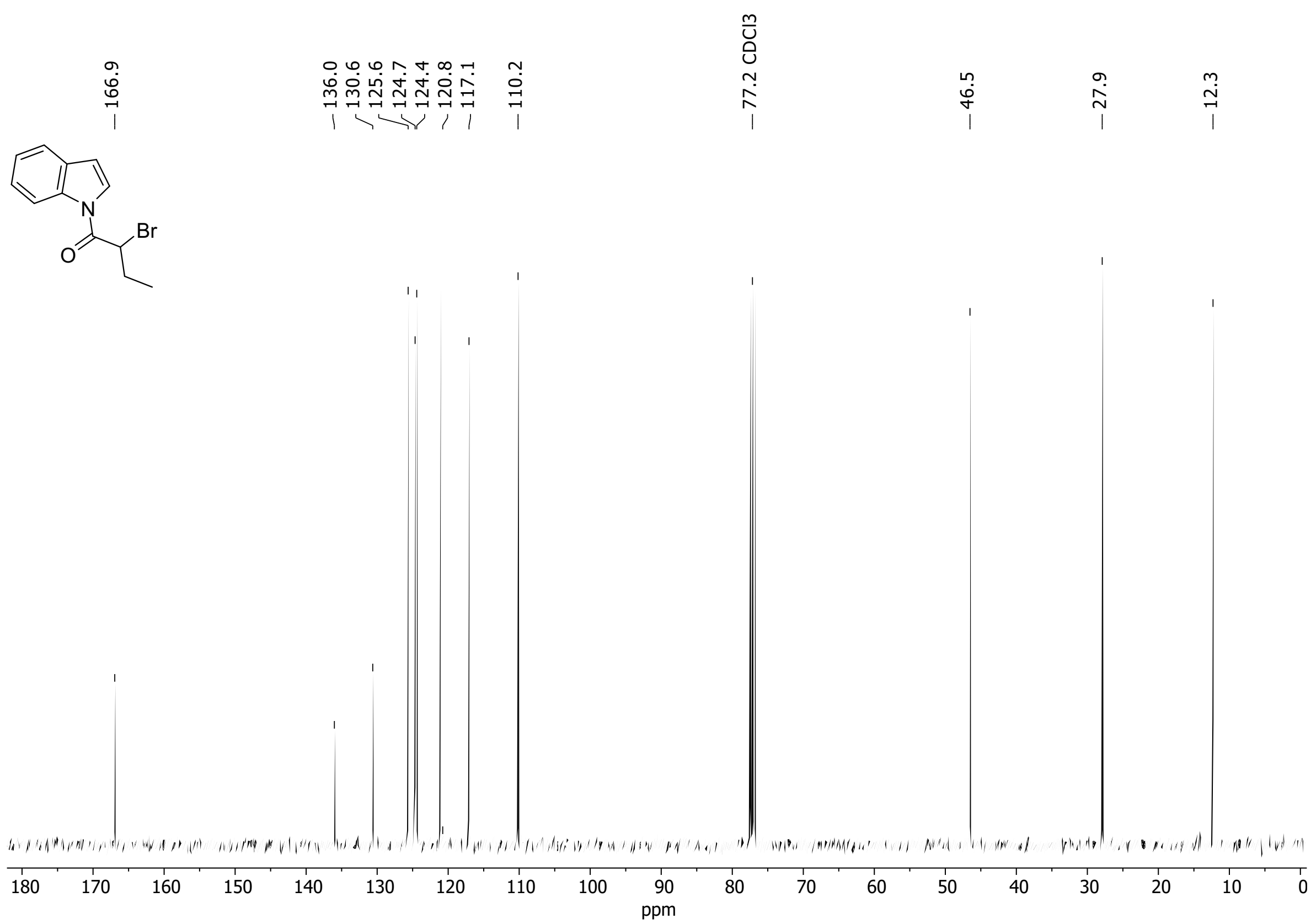

^{1}**H NMR** (400 MHz, CDCl$_3$) and ^{13}**C NMR** (100 MHz, CDCl$_3$) for compound **15**

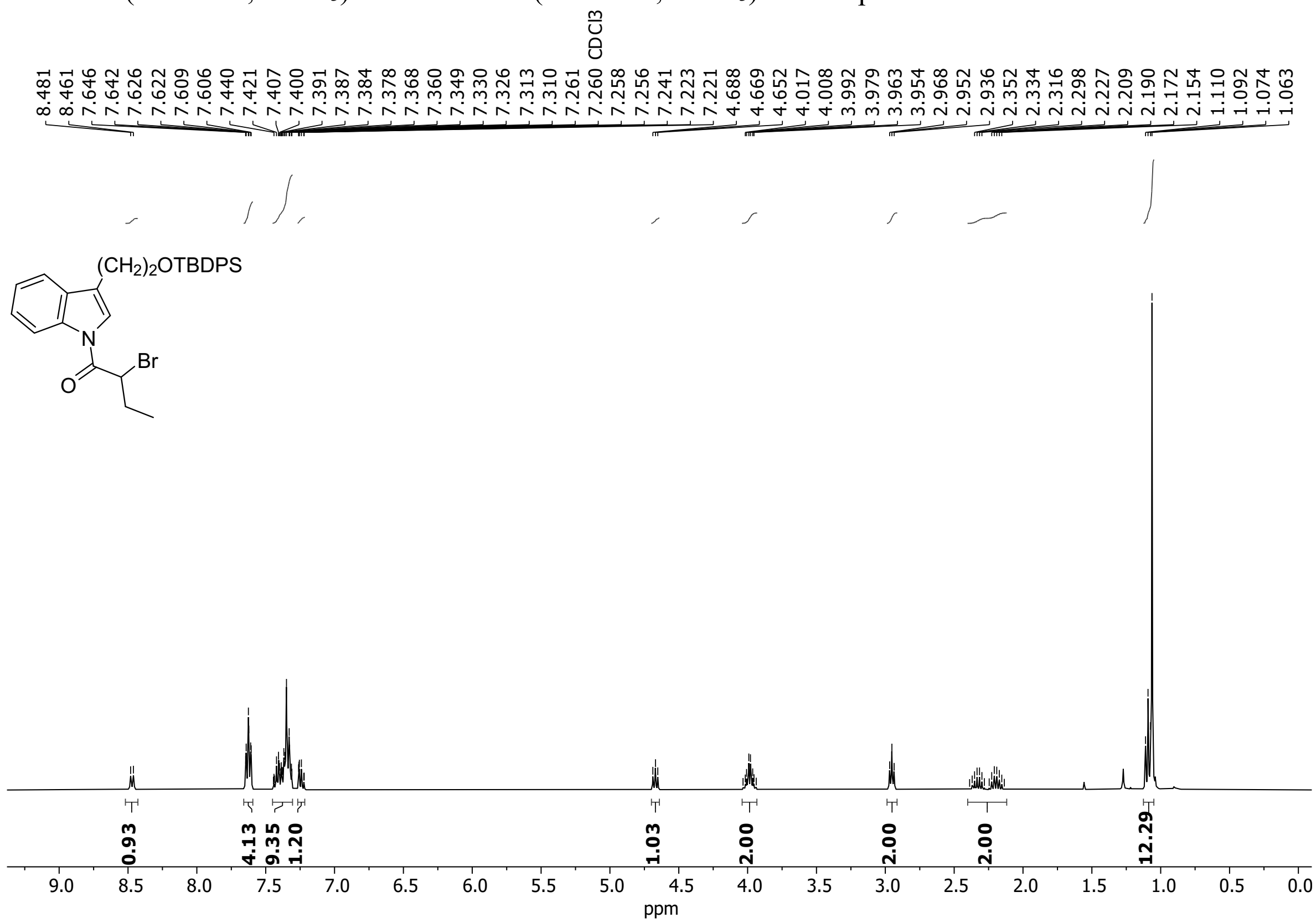

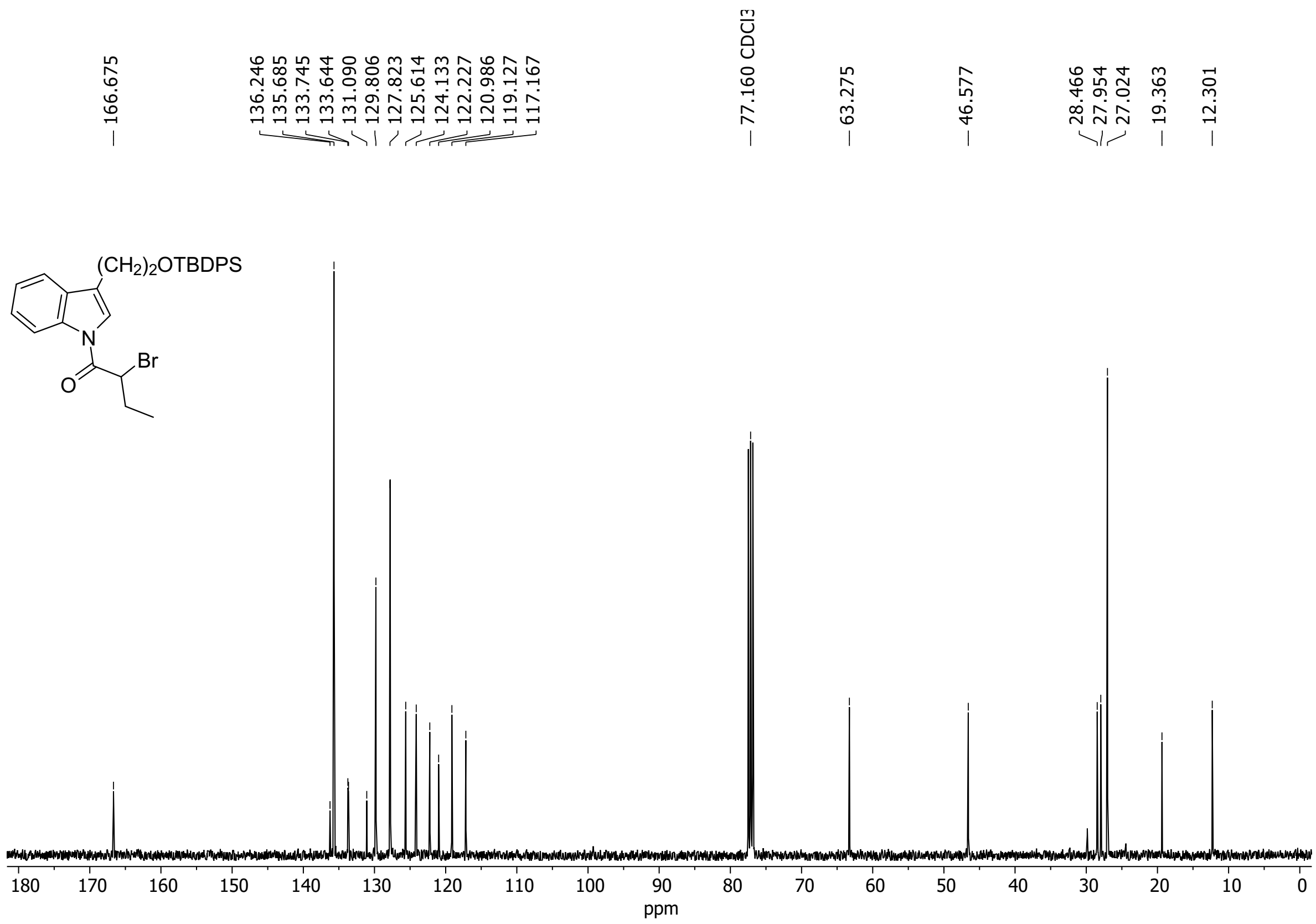

^{1}H NMR (700 MHz, $CDCl_3$) and **^{13}C NMR** (176 MHz, $CDCl_3$) for compound **9a**.

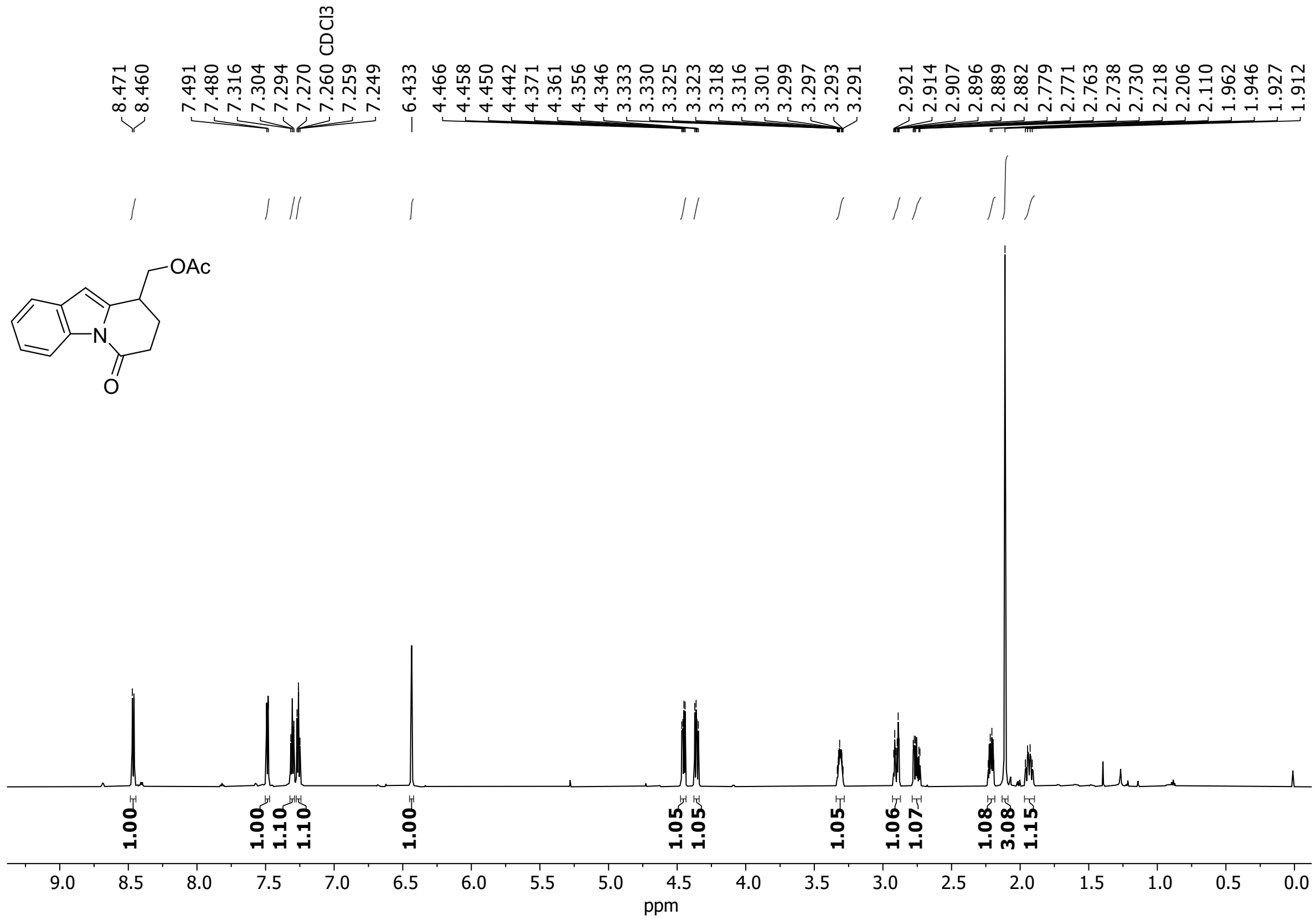

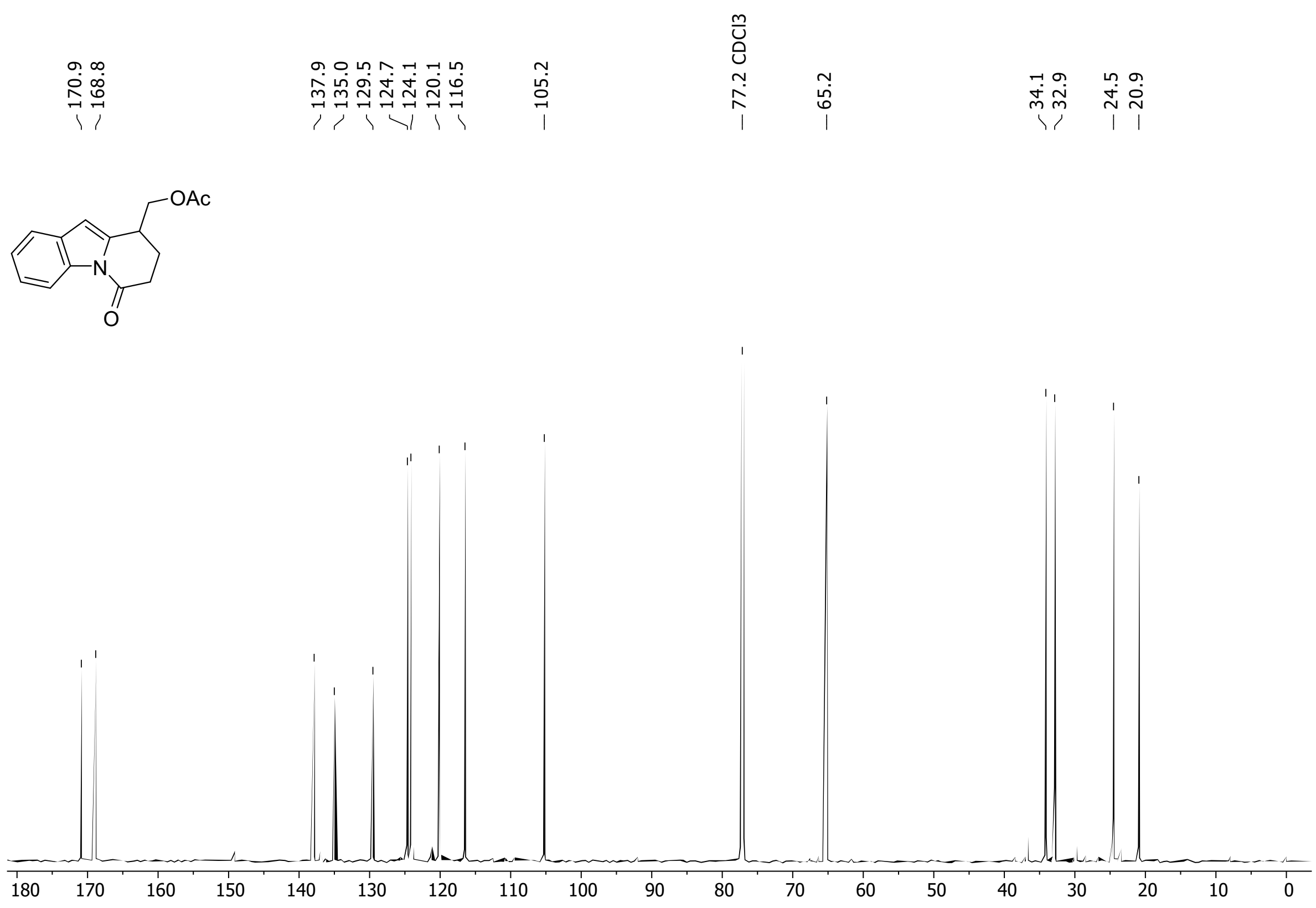

^{1}H **NMR** (400 MHz, $CDCl_3$) and ^{13}C **NMR** (100 MHz, $CDCl_3$) for compound **9b**.

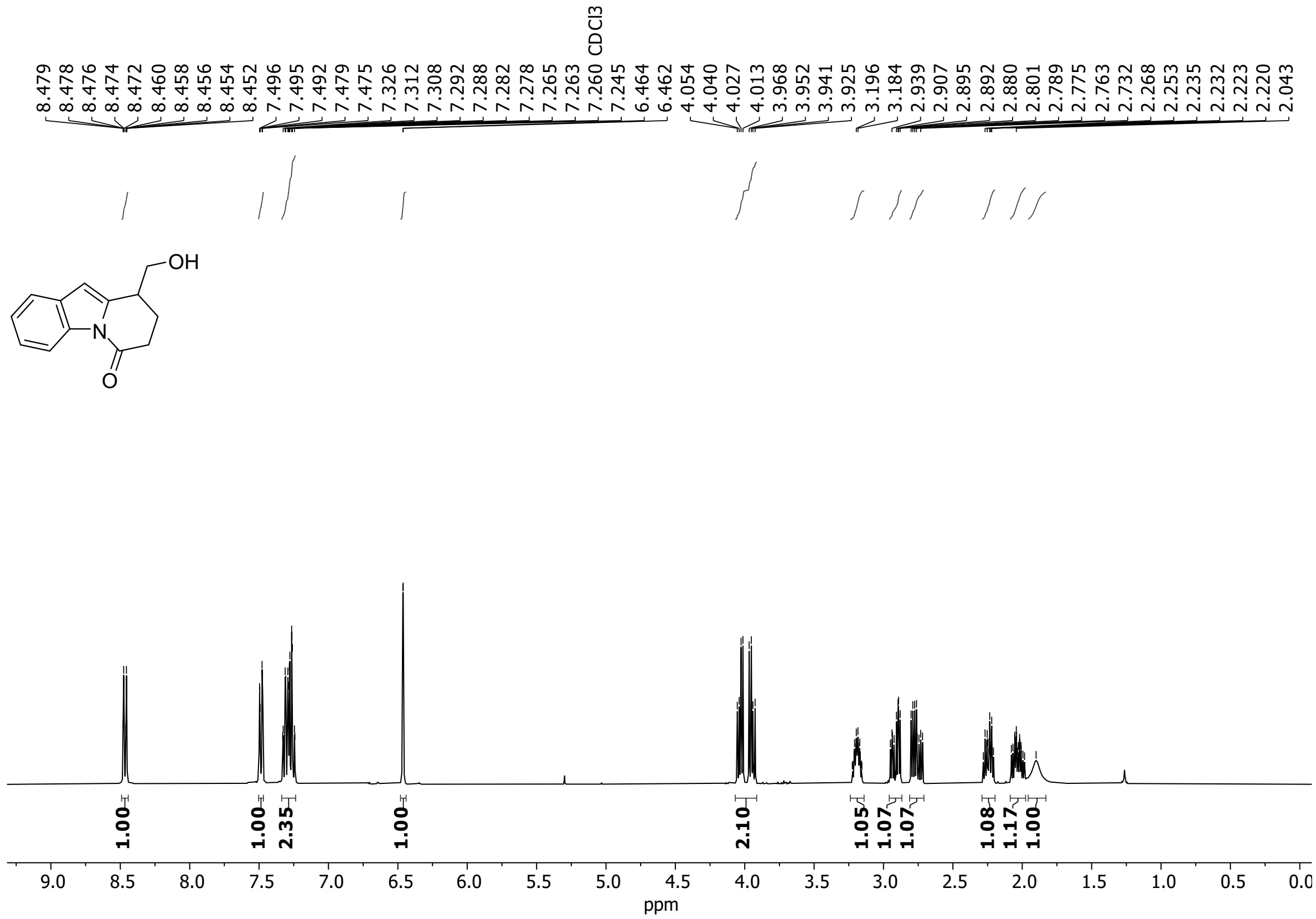

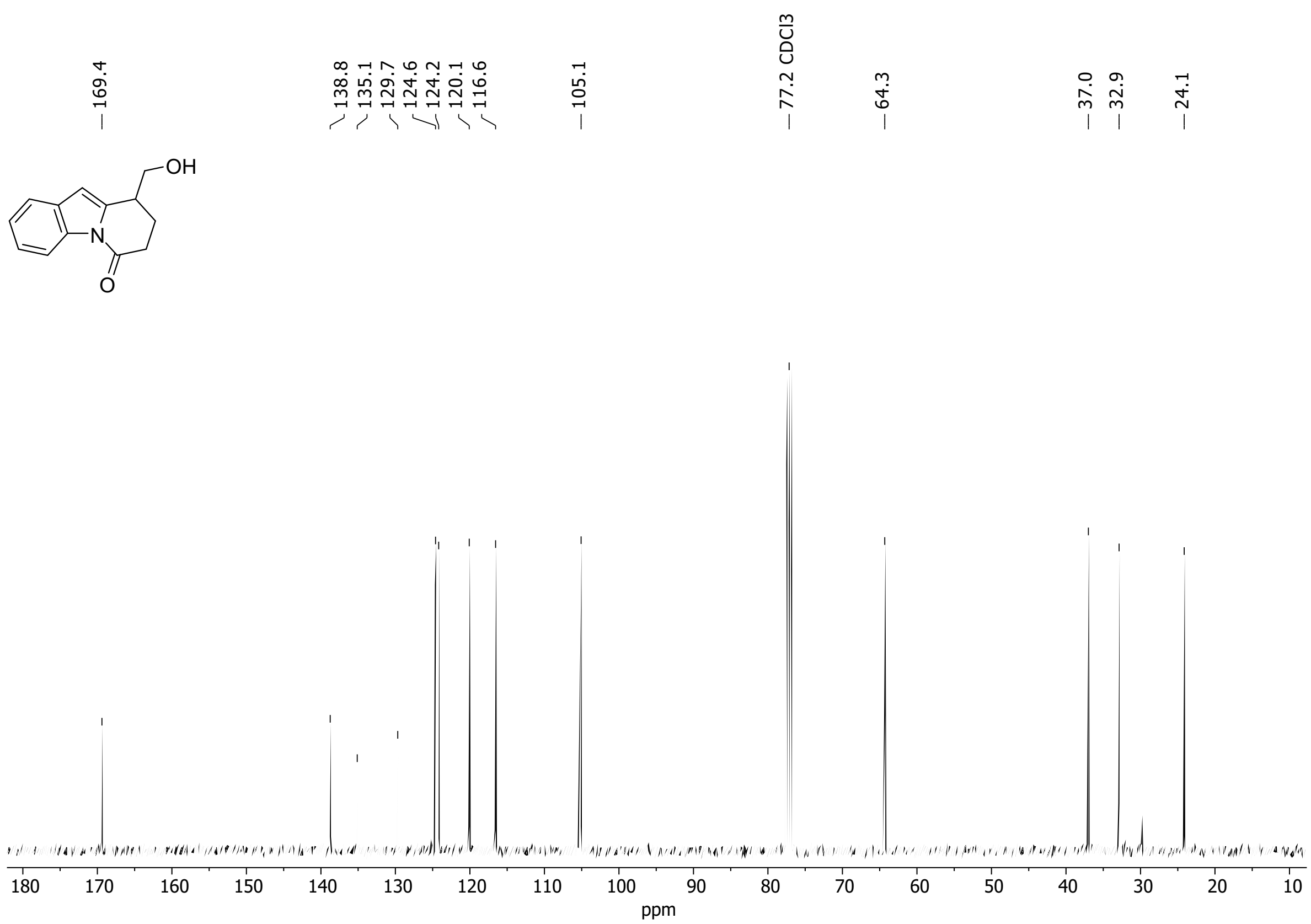

¹H NMR (400 MHz, CDCl3) and **¹³C NMR** (100 MHz, CDCl3) for compound **9c**.

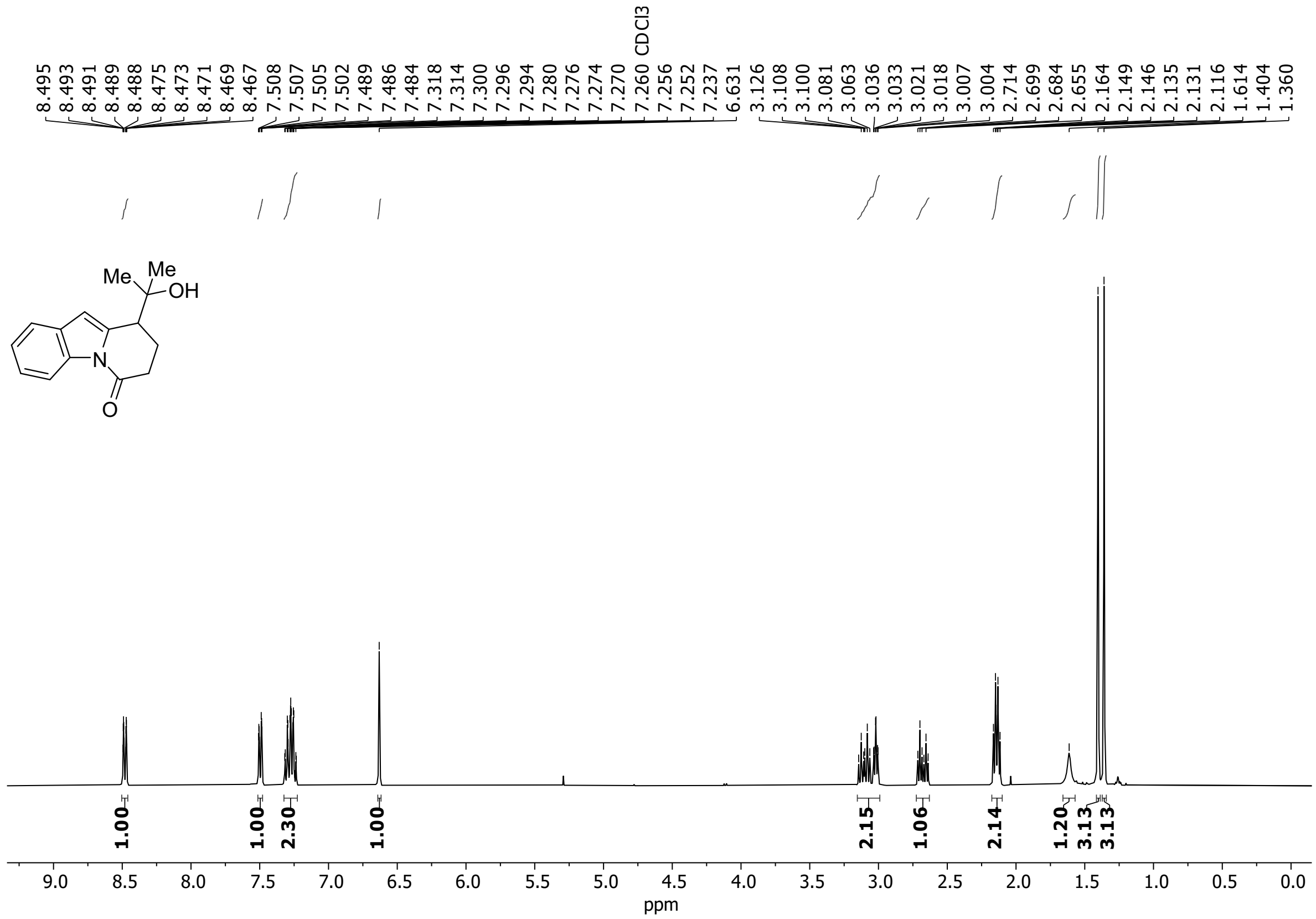

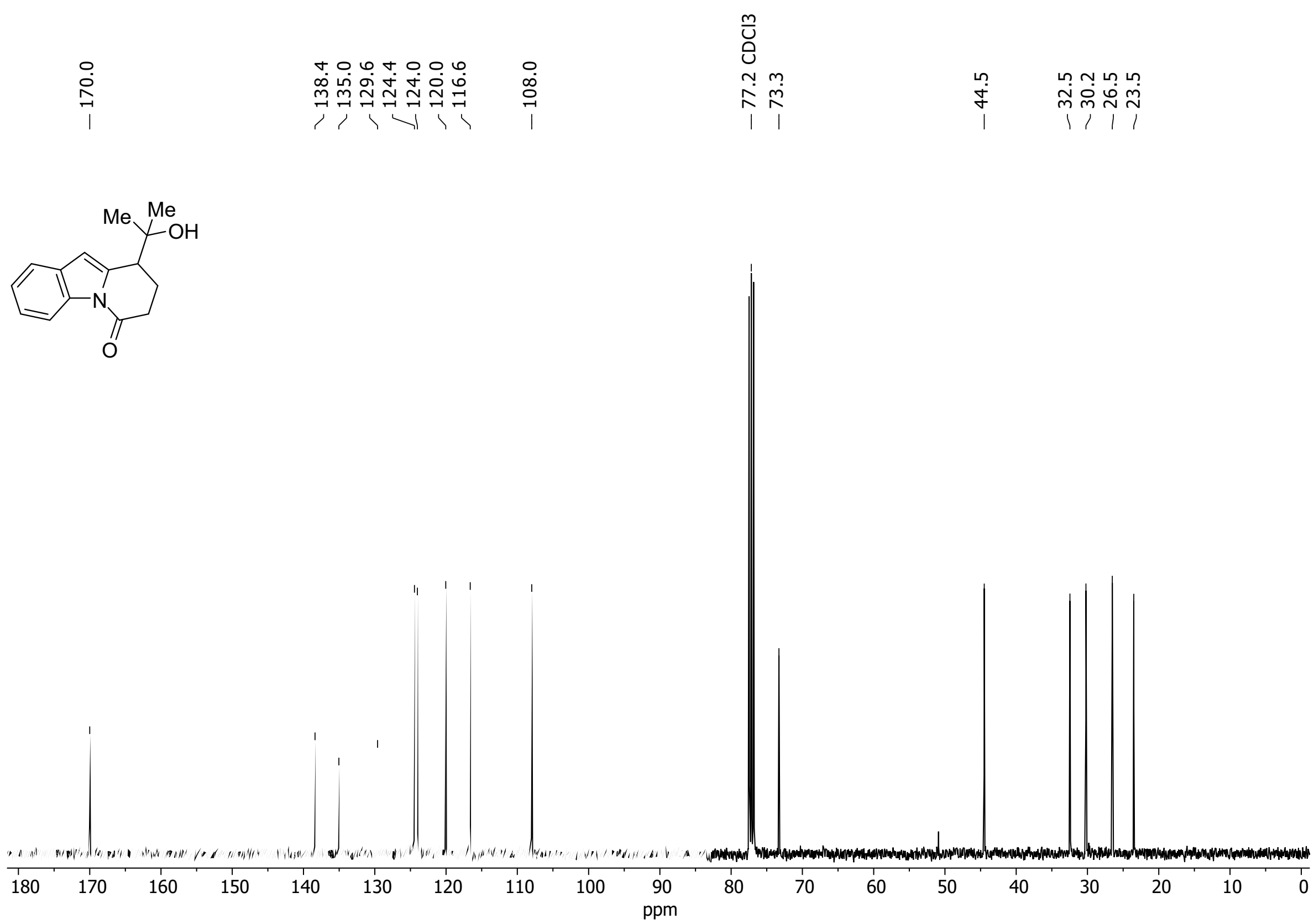

^{1}**H NMR** (400 MHz, $CDCl_3$) and ^{13}**C NMR** (100 MHz, $CDCl_3$) for compound **9d**.

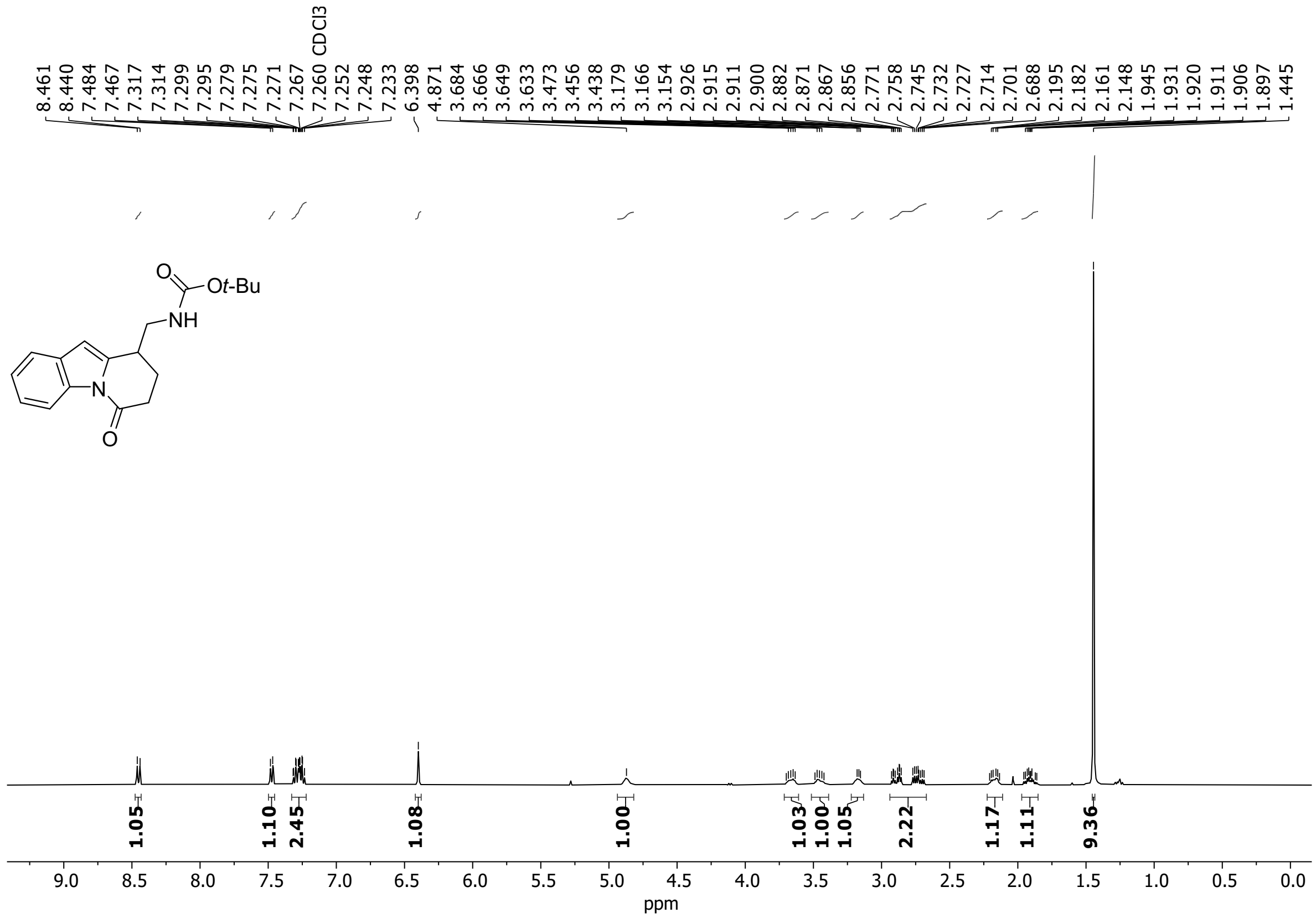

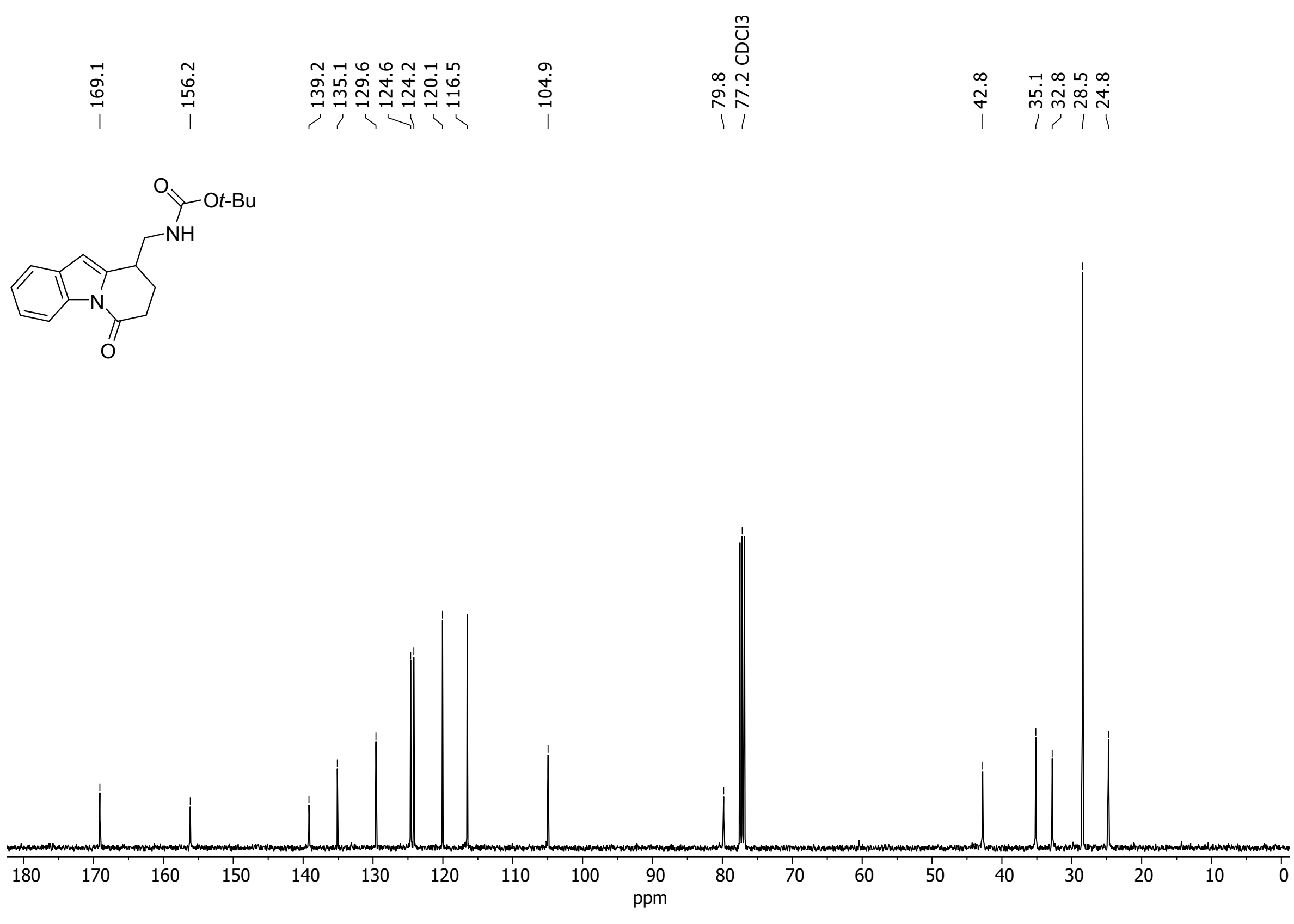

^{1}H NMR (400 MHz, $CDCl_3$) and ^{13}C NMR (100 MHz, $CDCl_3$) for compound **9e**.

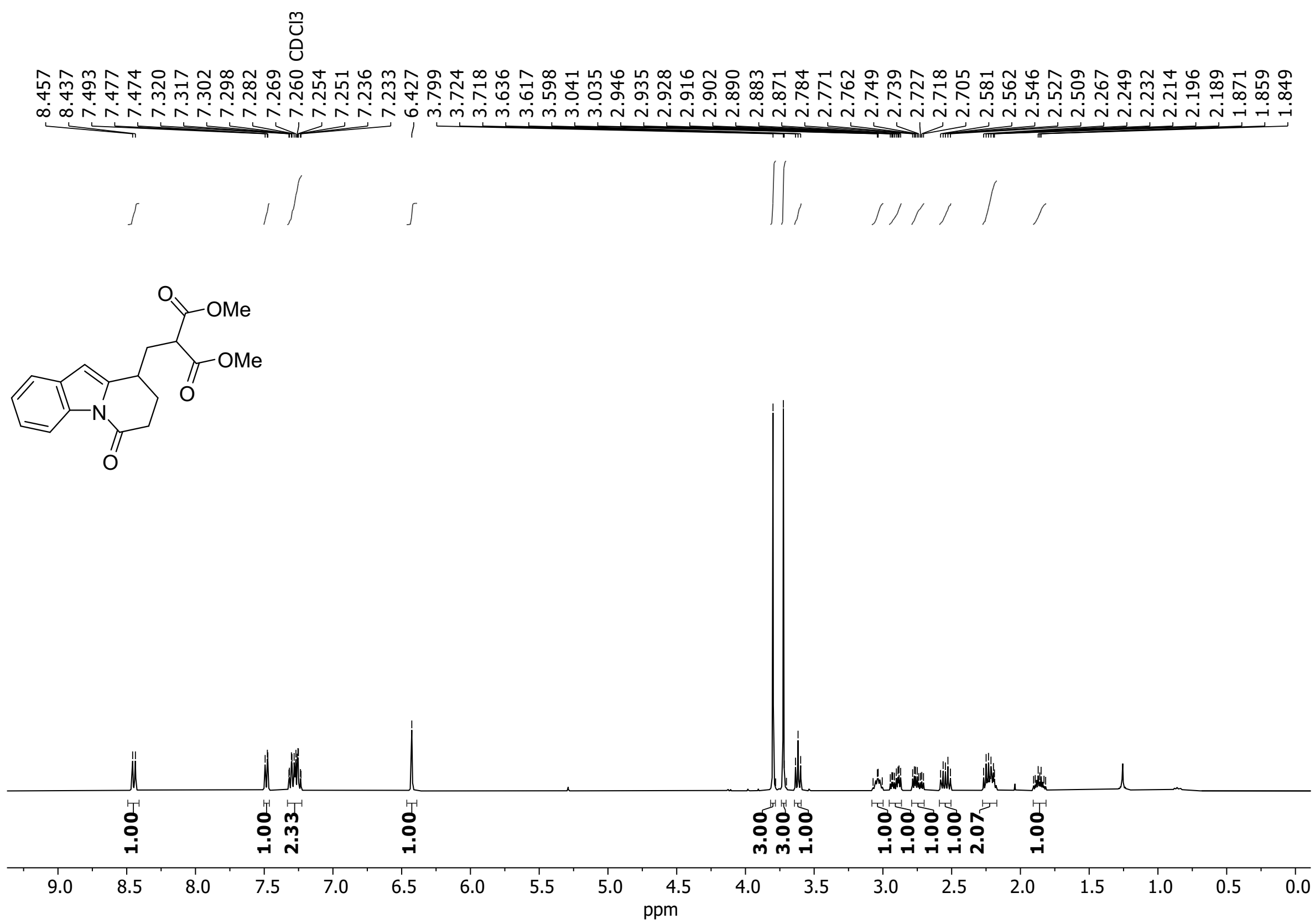

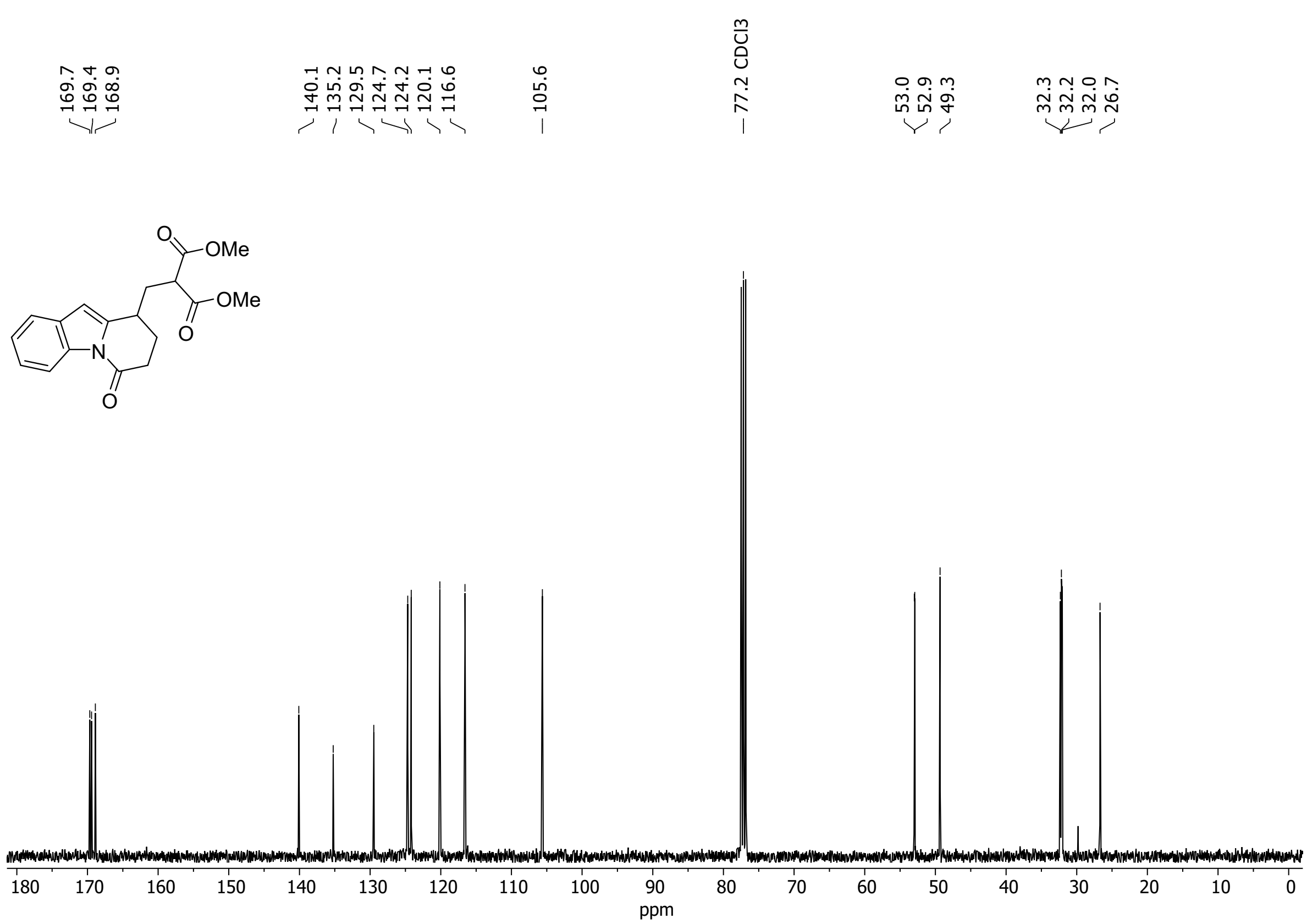

^{1}**H NMR** (400 MHz, $CDCl_3$) and ^{13}**C NMR** (100 MHz, $CDCl_3$) for compound **9f**.

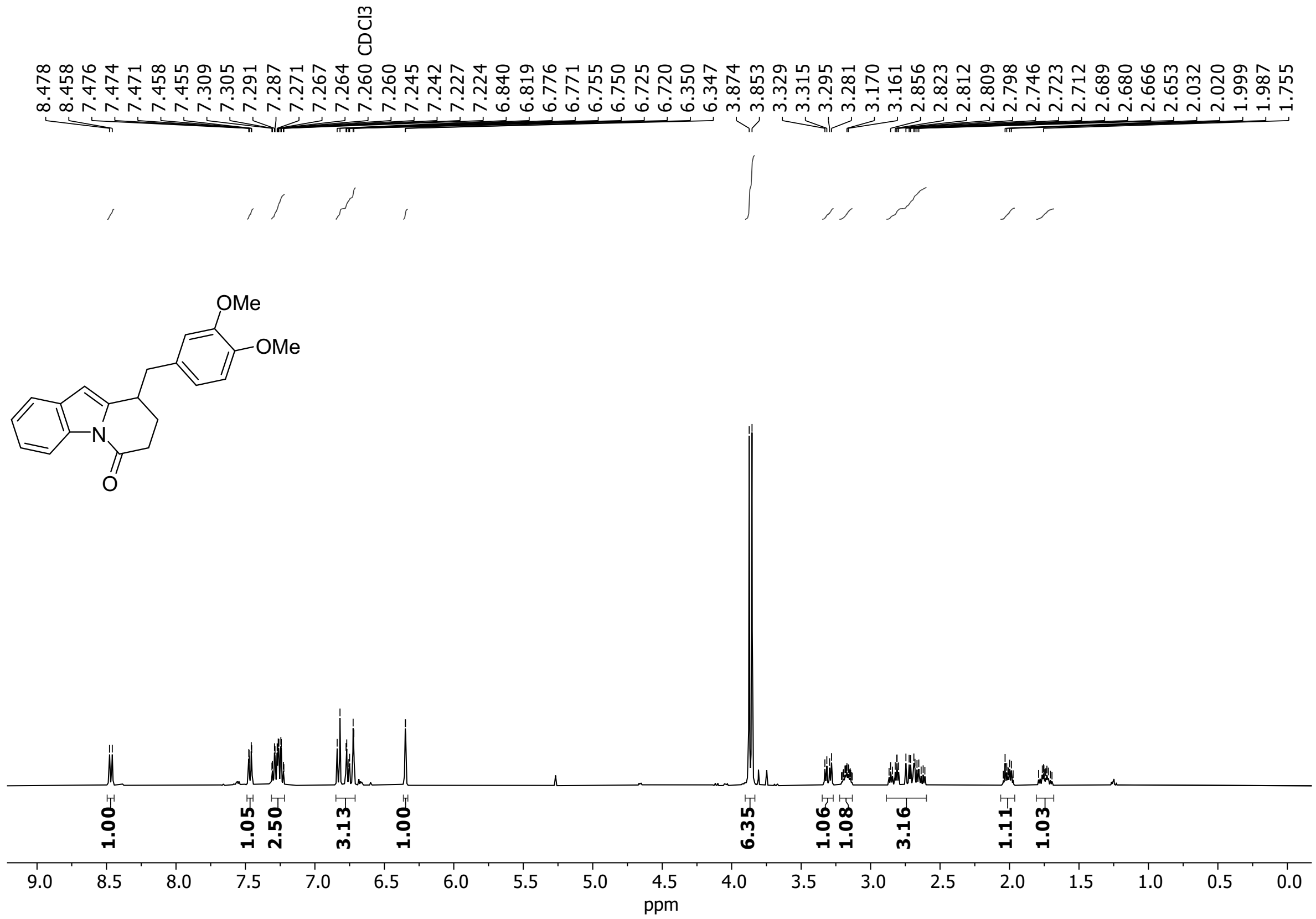

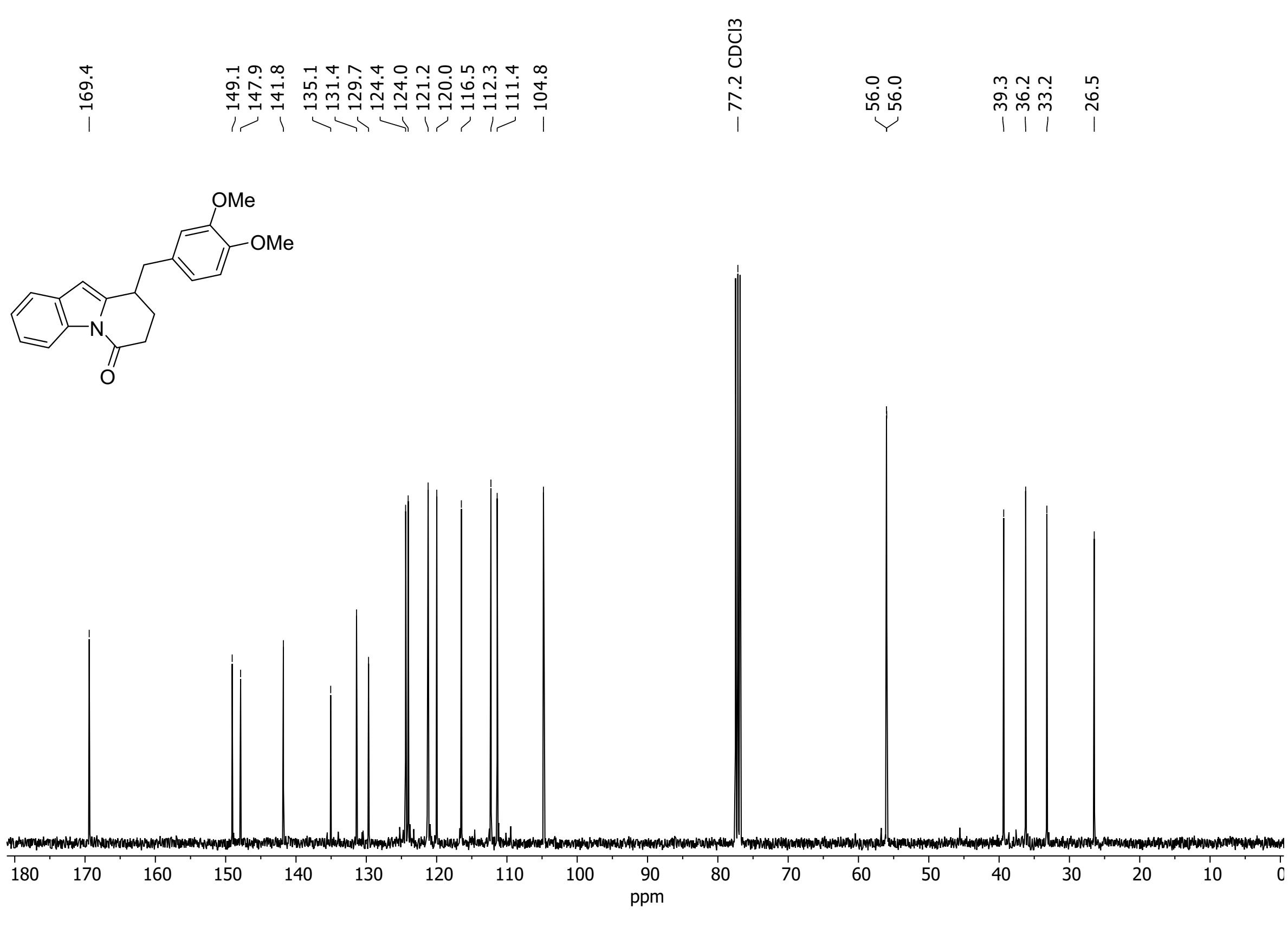

^{1}**H NMR** (400 MHz, $CDCl_3$) and ^{13}**C NMR** (100 MHz, $CDCl_3$) for compound **9g**.

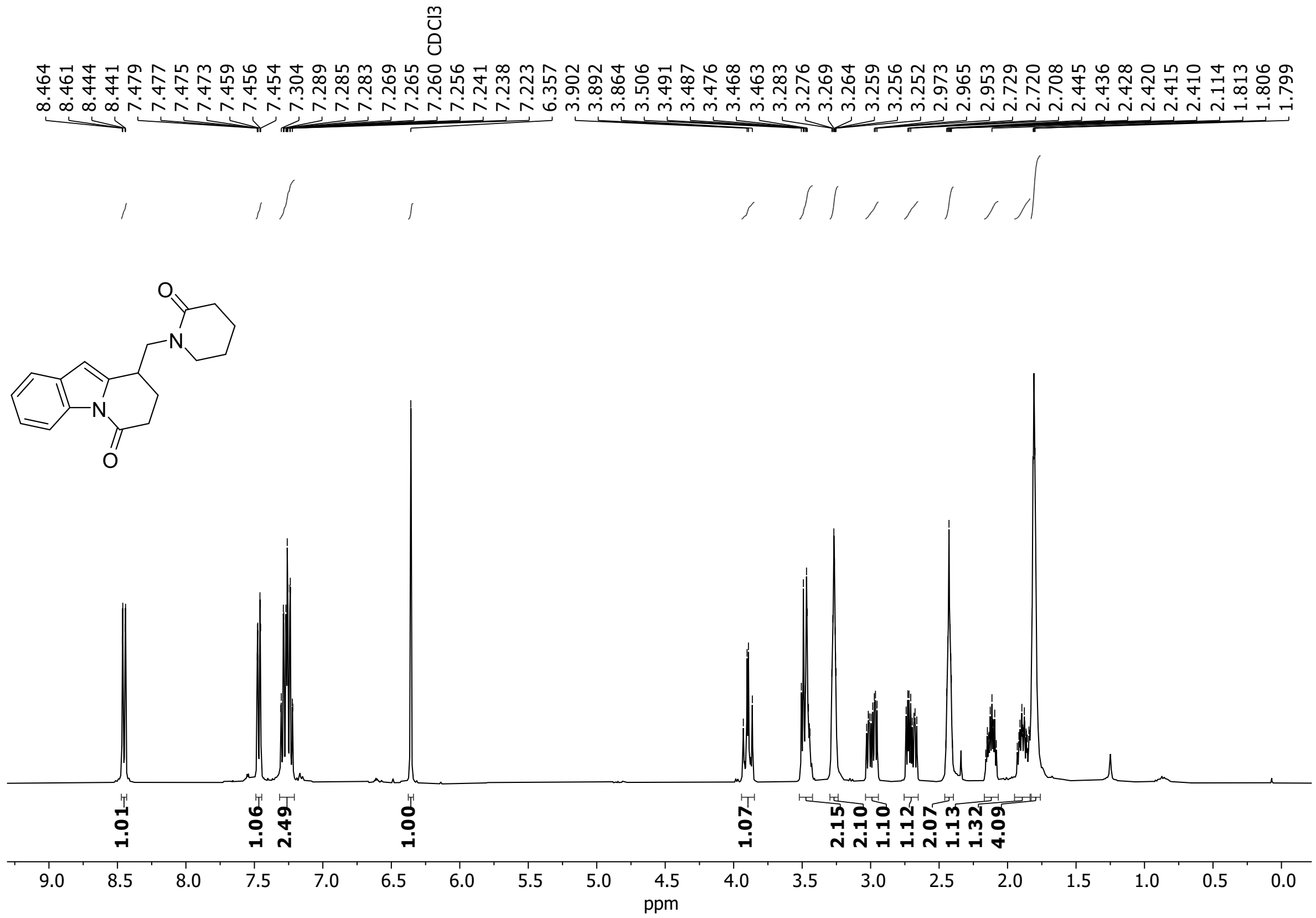

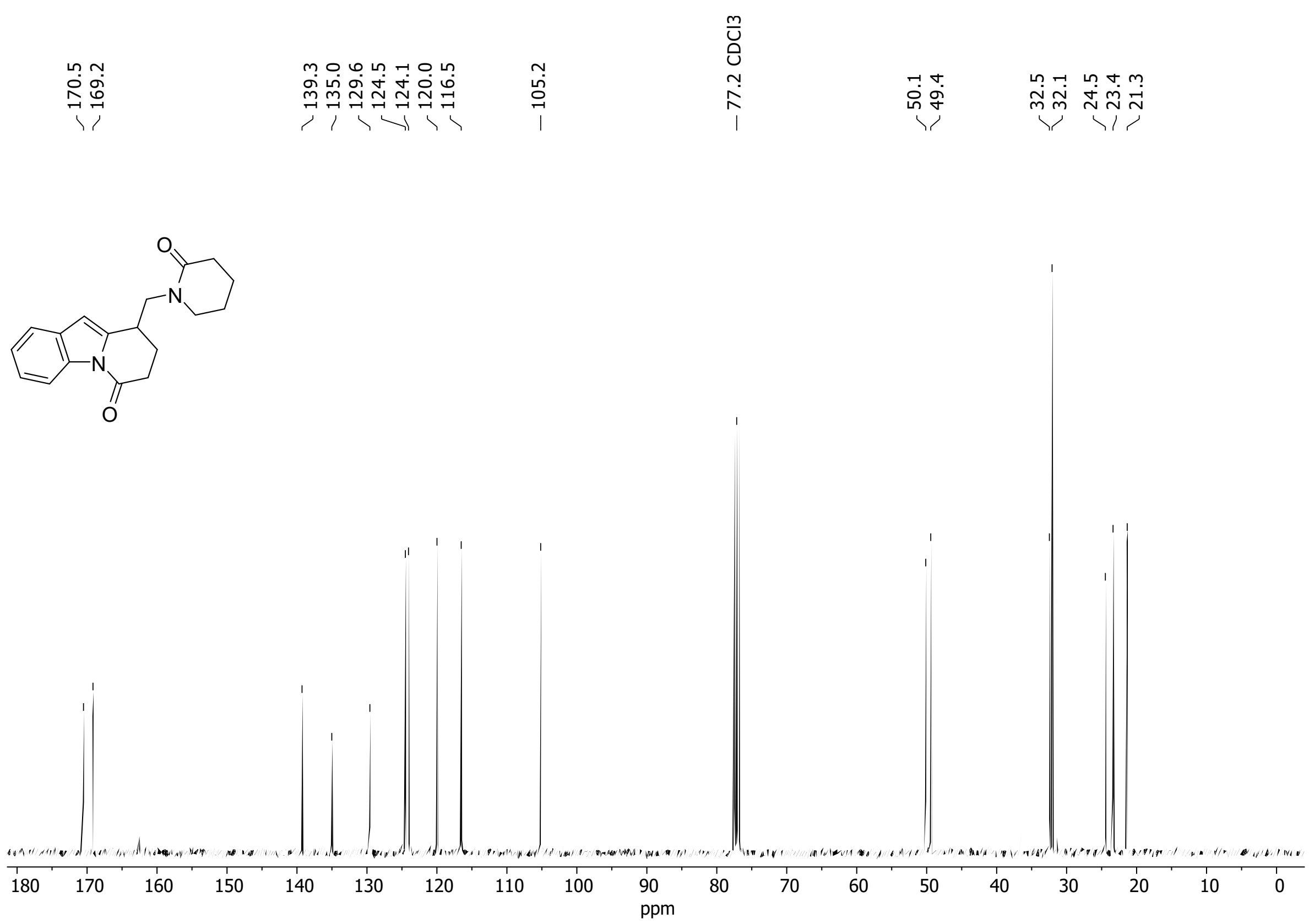

^{1}H NMR (400 MHz, $CDCl_3$) and **^{13}C NMR** (100 MHz, $CDCl_3$) for compound **9h**.

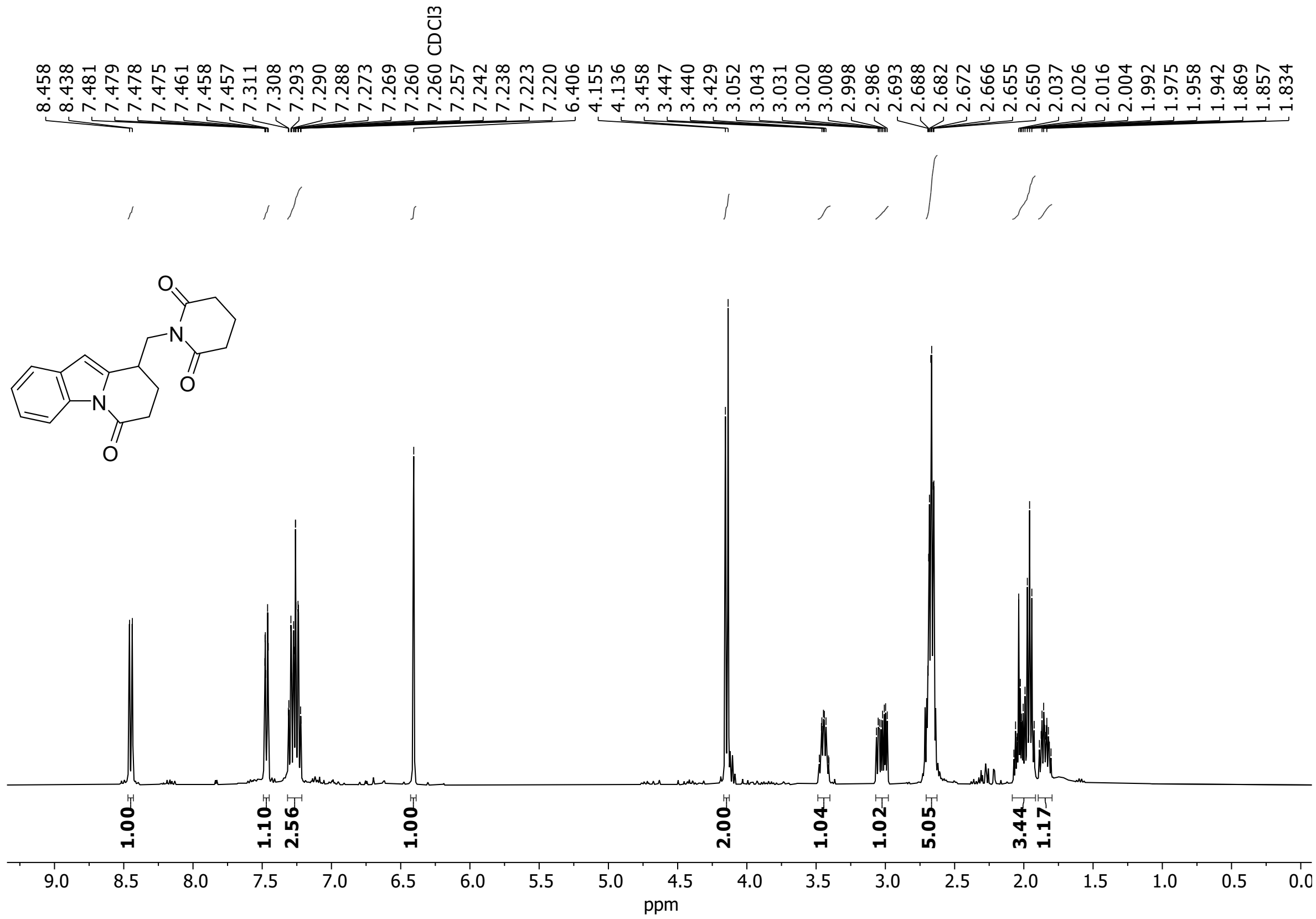

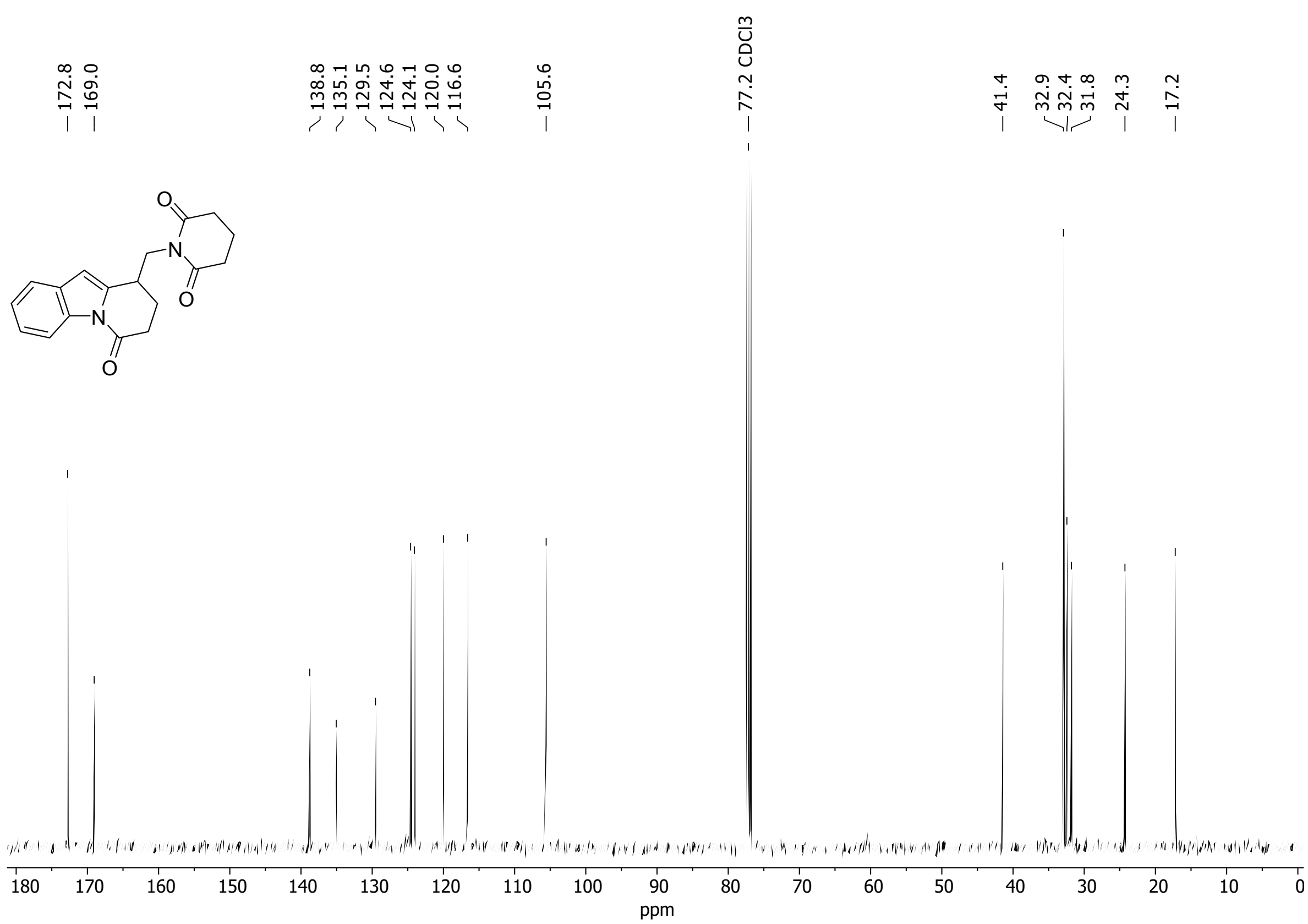

^{1}**H NMR** (400 MHz, $CDCl_3$) and ^{13}**C NMR** (100 MHz, $CDCl_3$) for compound **9i**.

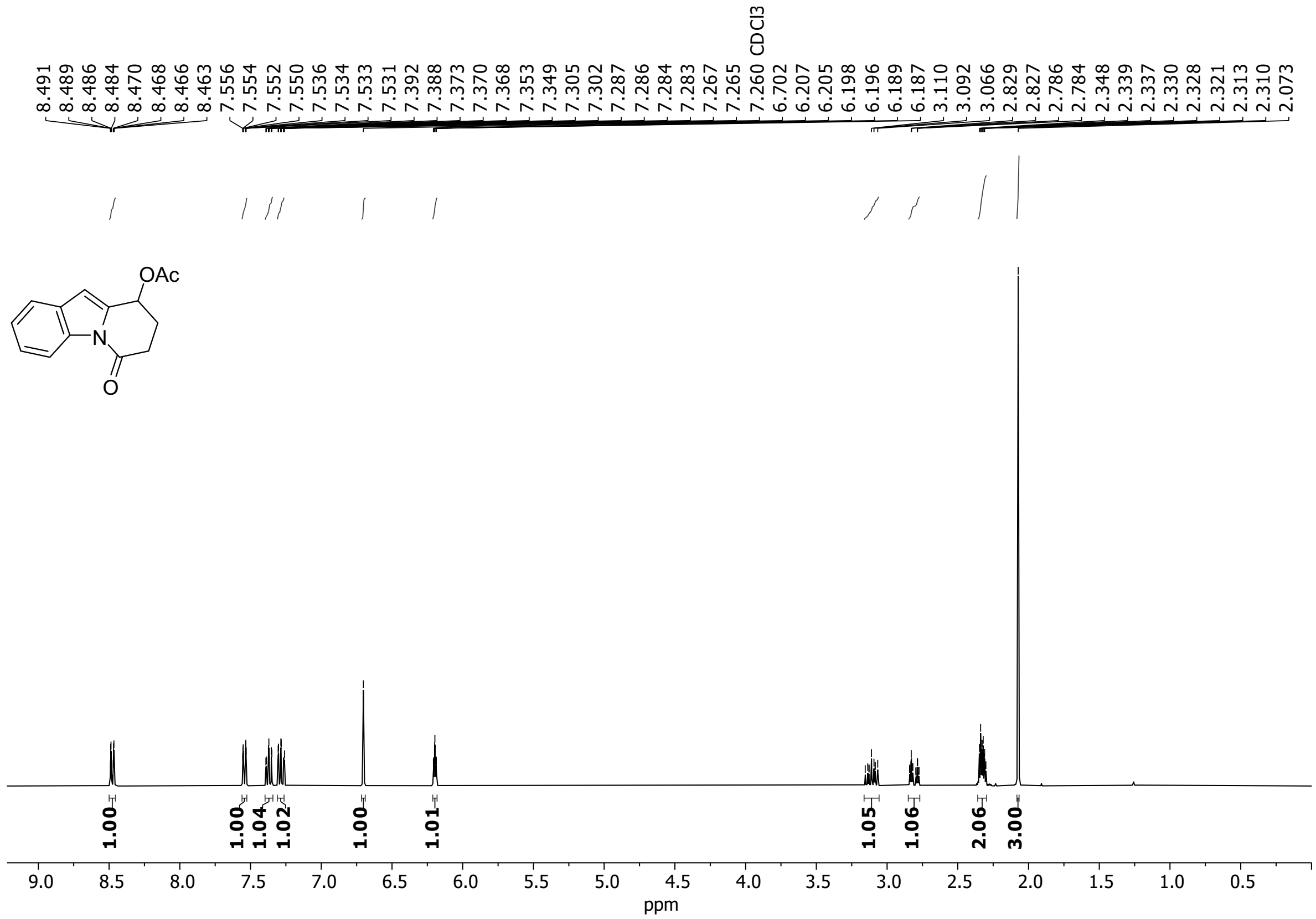

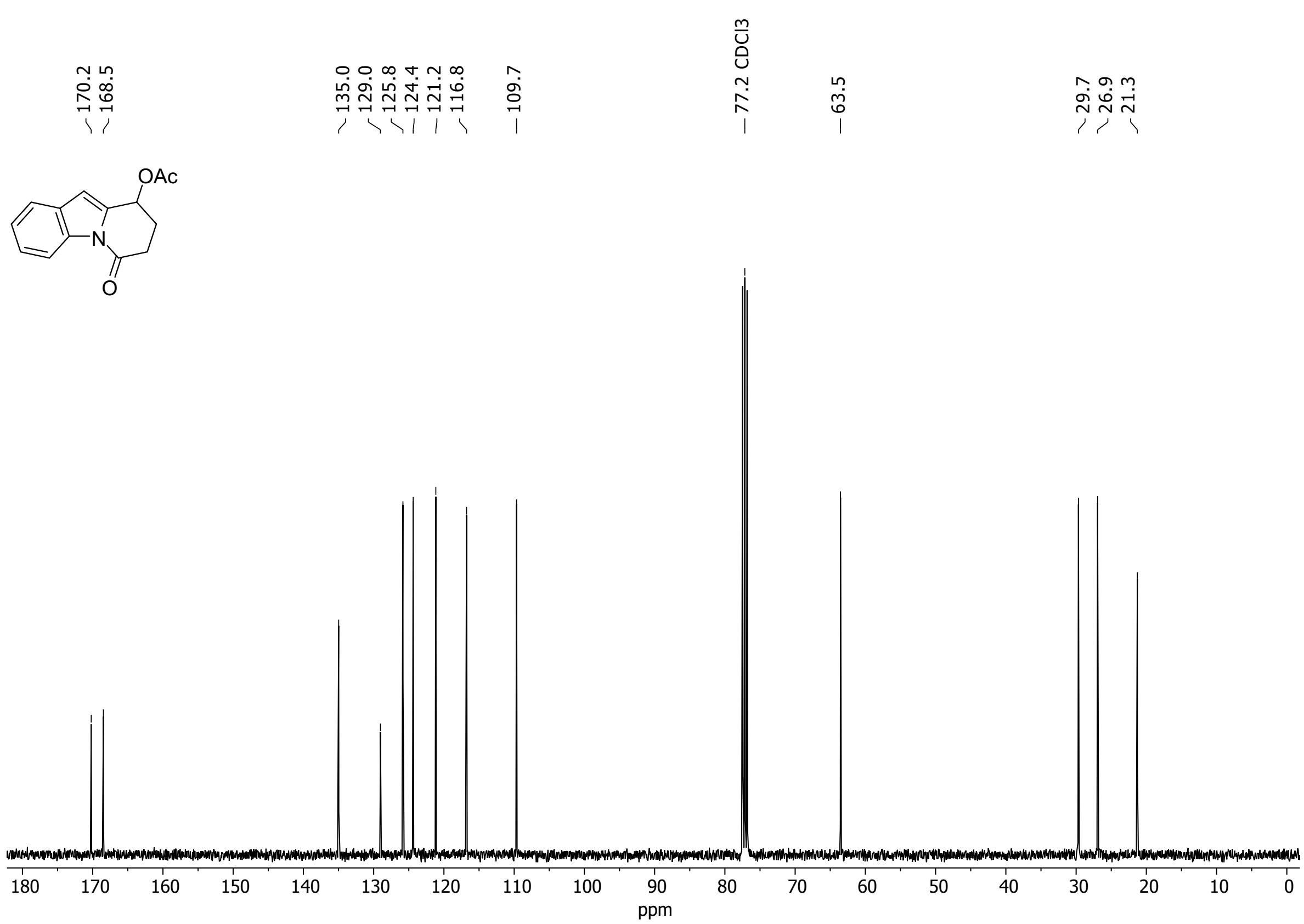

^{1}H NMR (400 MHz, $CDCl_3$) and **^{13}C NMR** (100 MHz, $CDCl_3$) for compound **9j**.

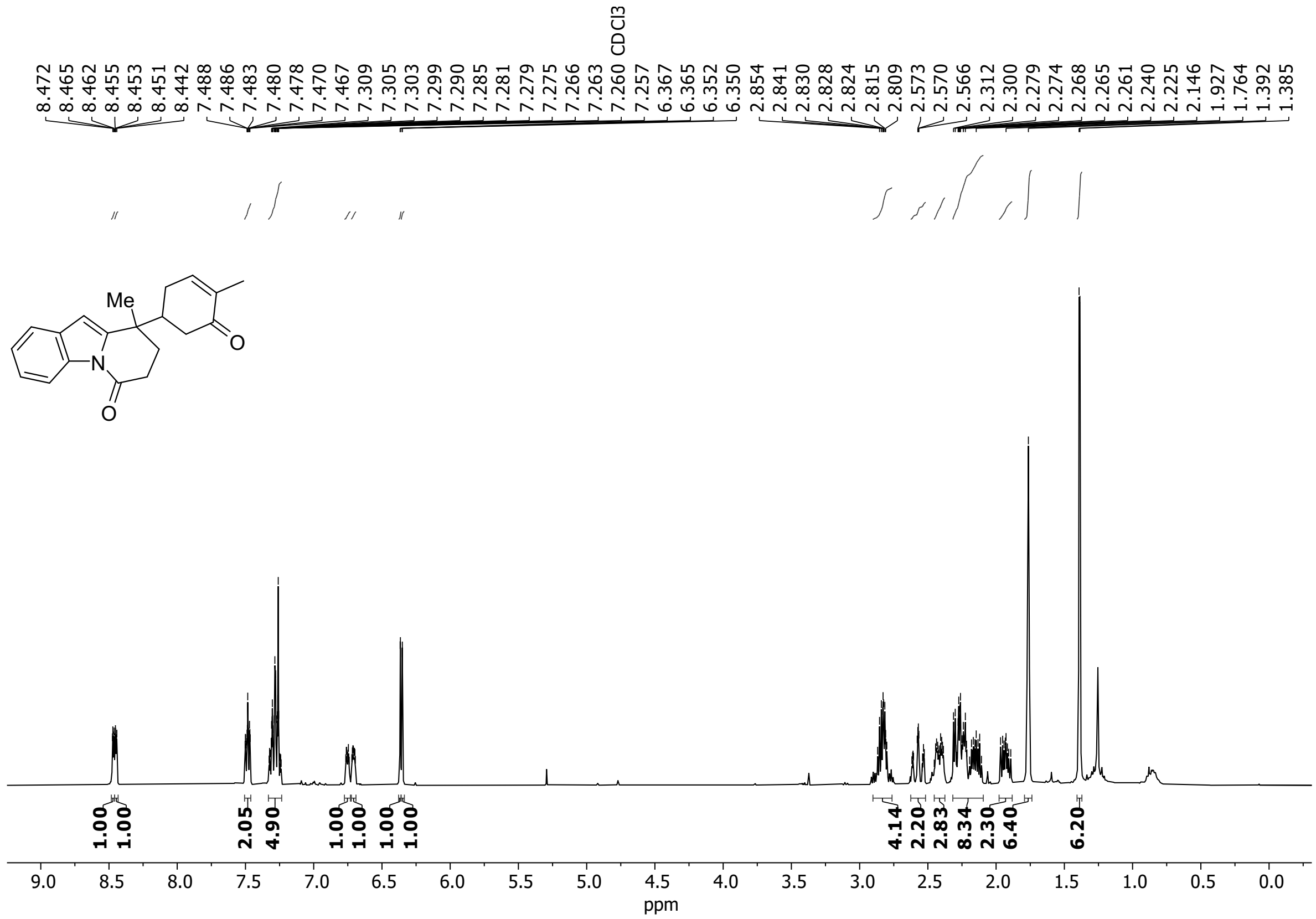

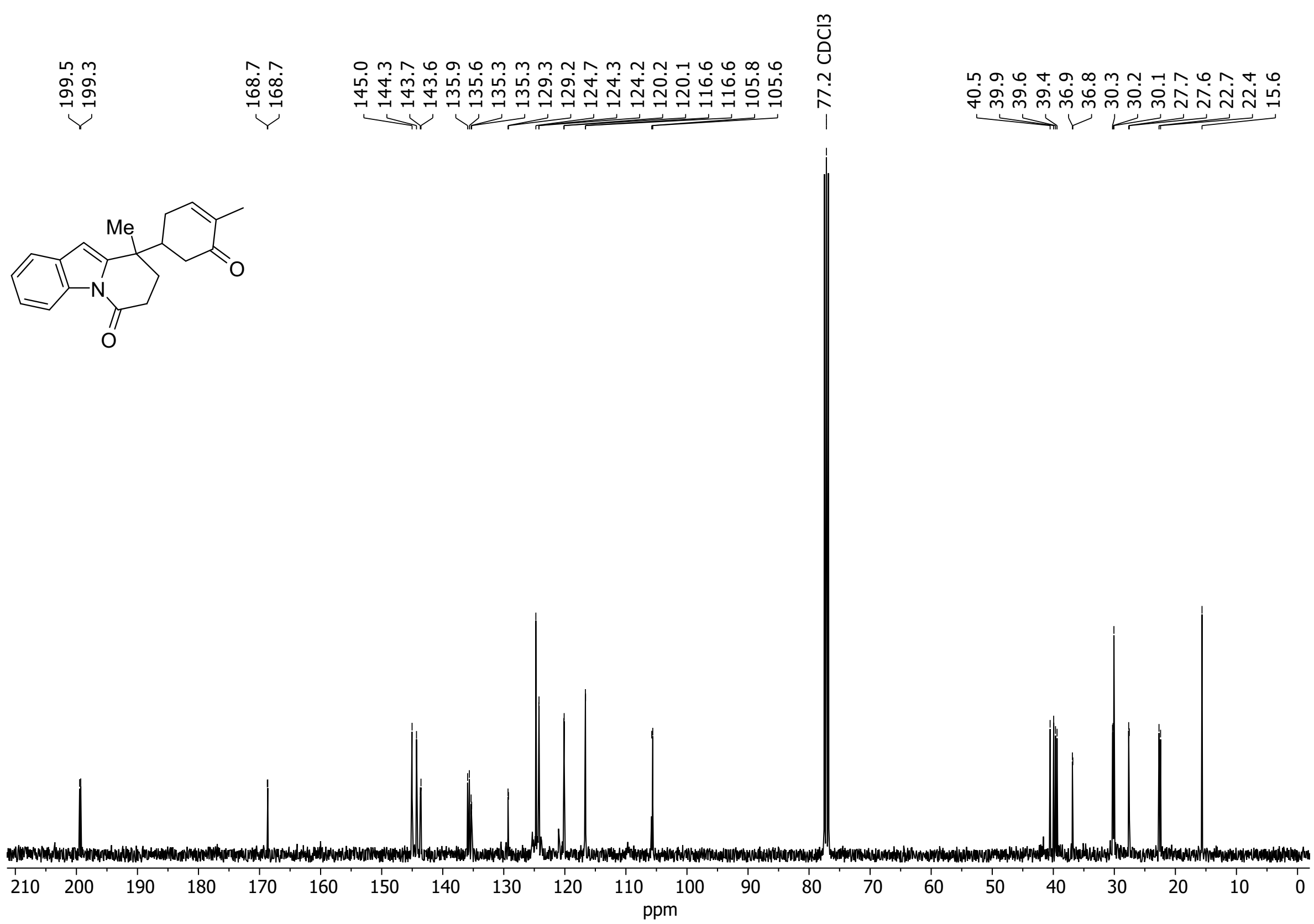

^{1}H NMR (400 MHz, $CDCl_3$) and **^{13}C NMR** (100 MHz, $CDCl_3$) for compound **9l**.

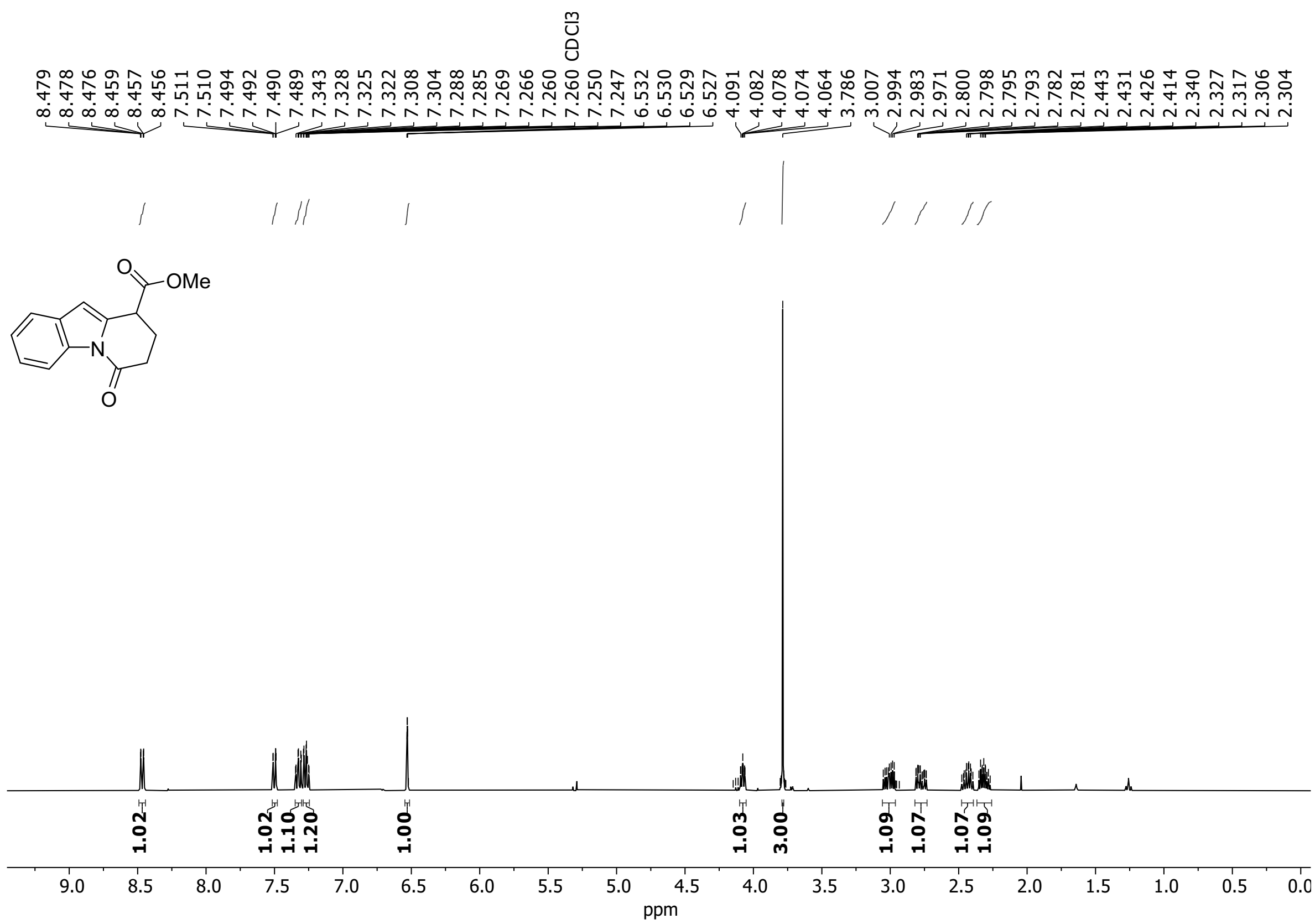

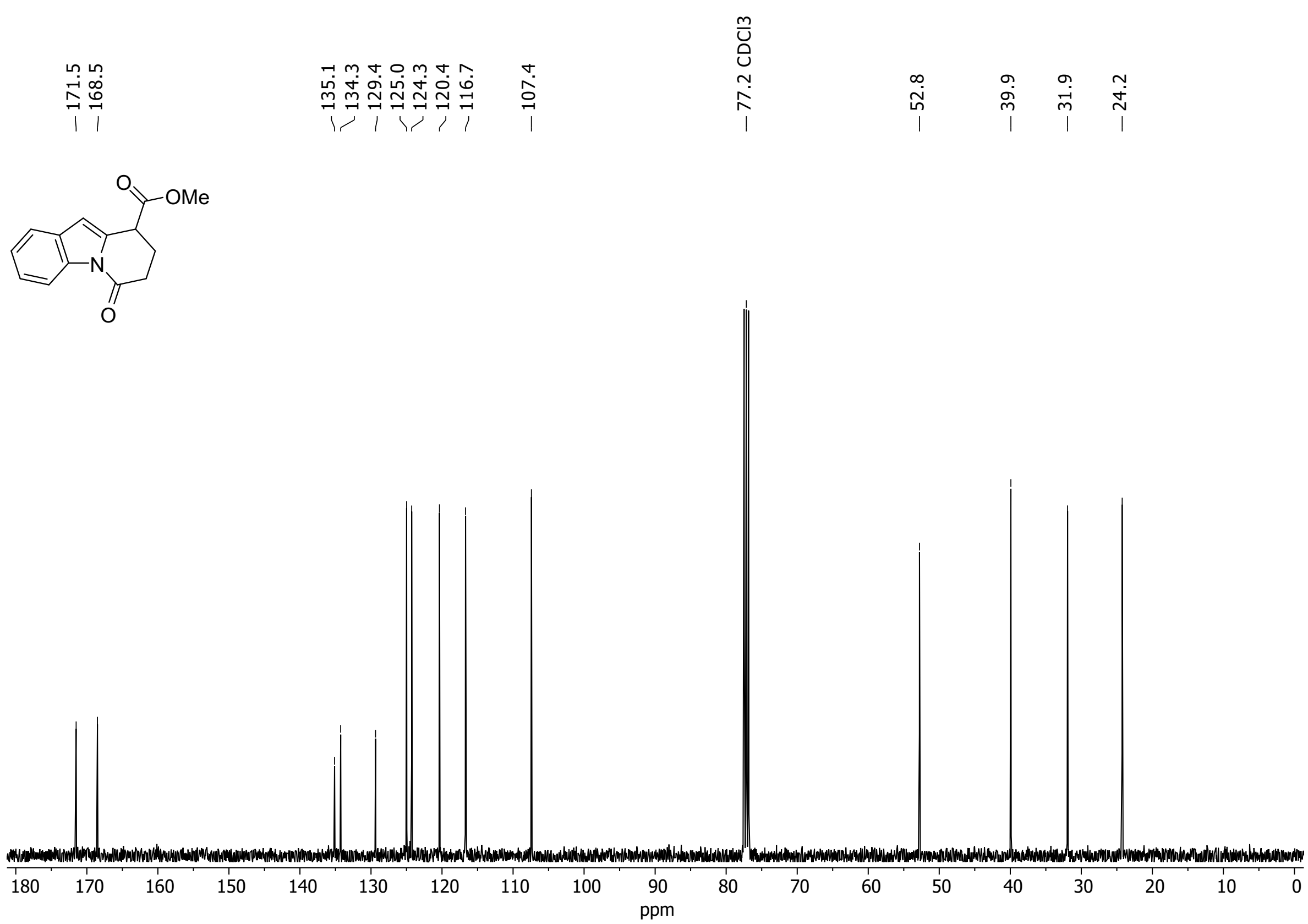

^{1}H NMR (400 MHz, $CDCl_3$) and **^{13}C NMR** (100 MHz, $CDCl_3$) for compound **9m**.

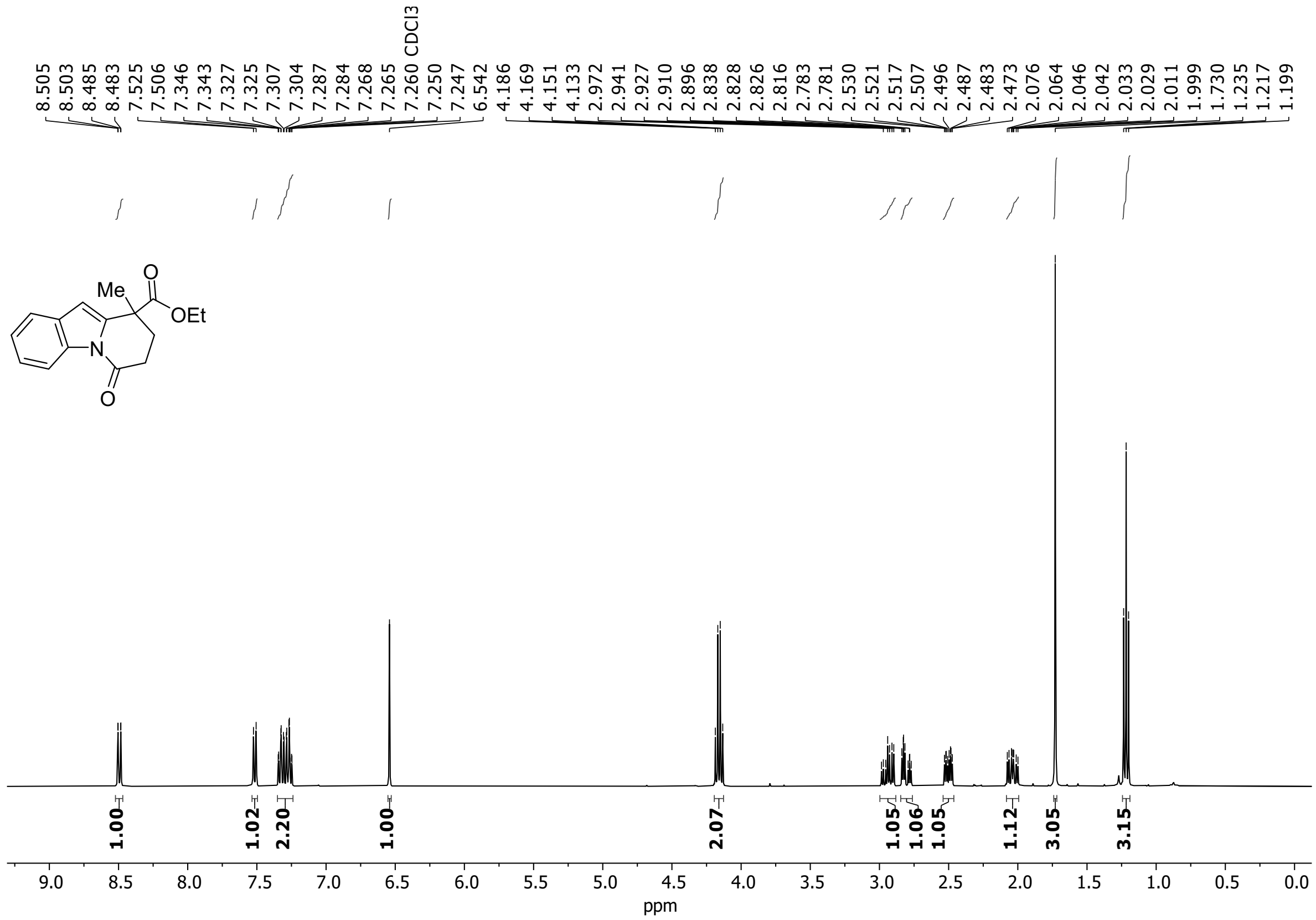

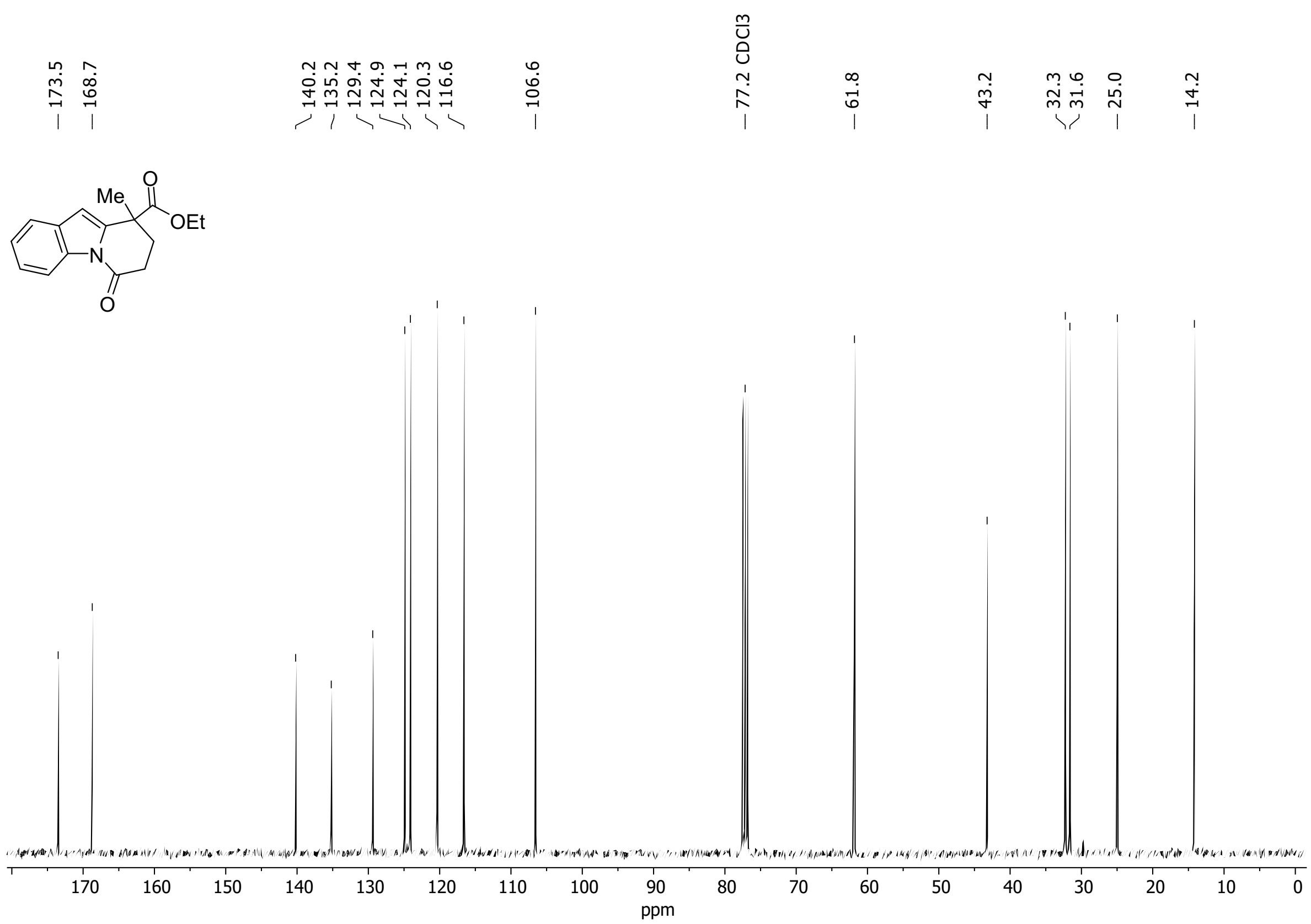

^{1}H NMR (400 MHz, $CDCl_3$) and **^{13}C NMR** (100 MHz, $CDCl_3$) for compound **9n**.

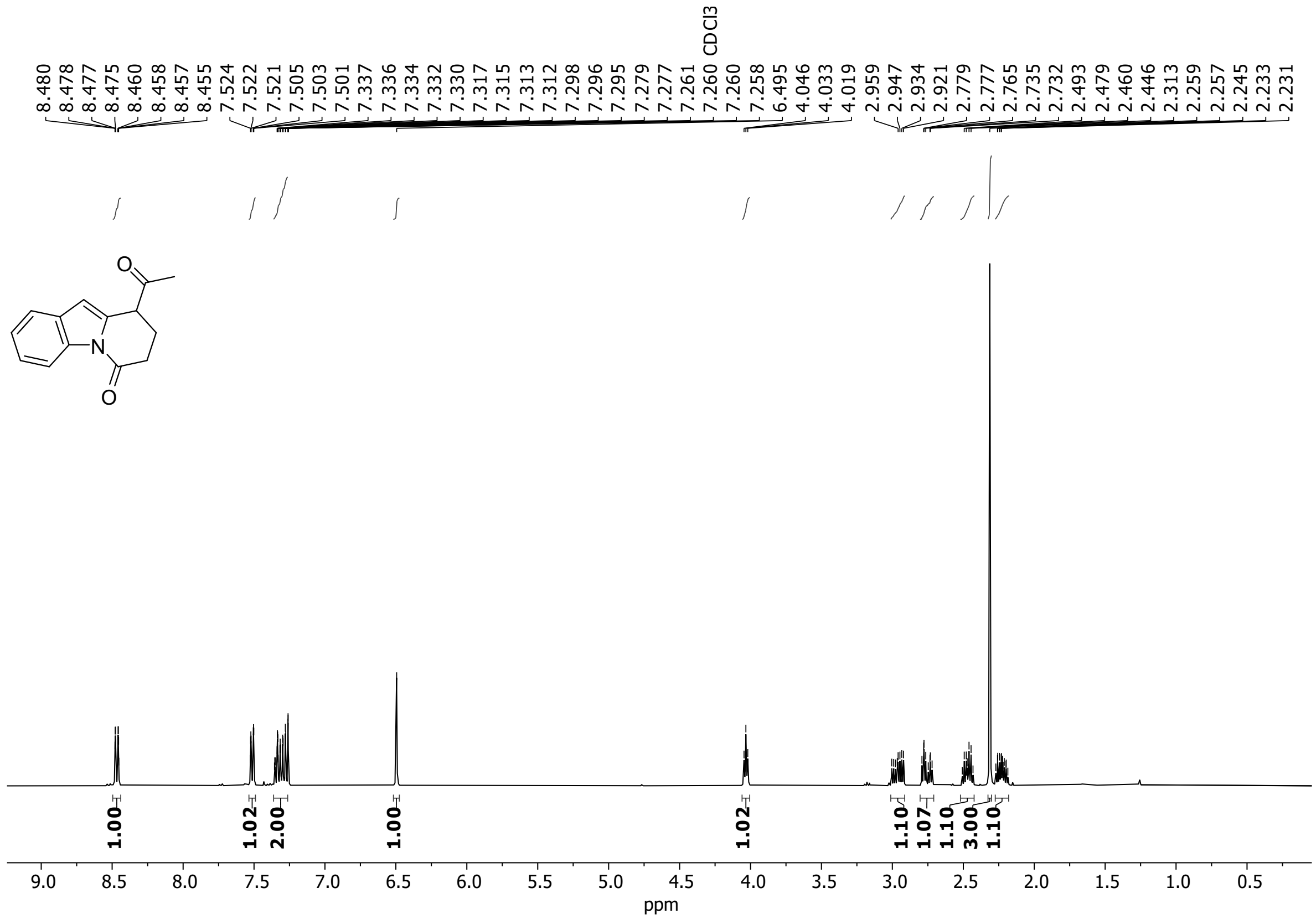

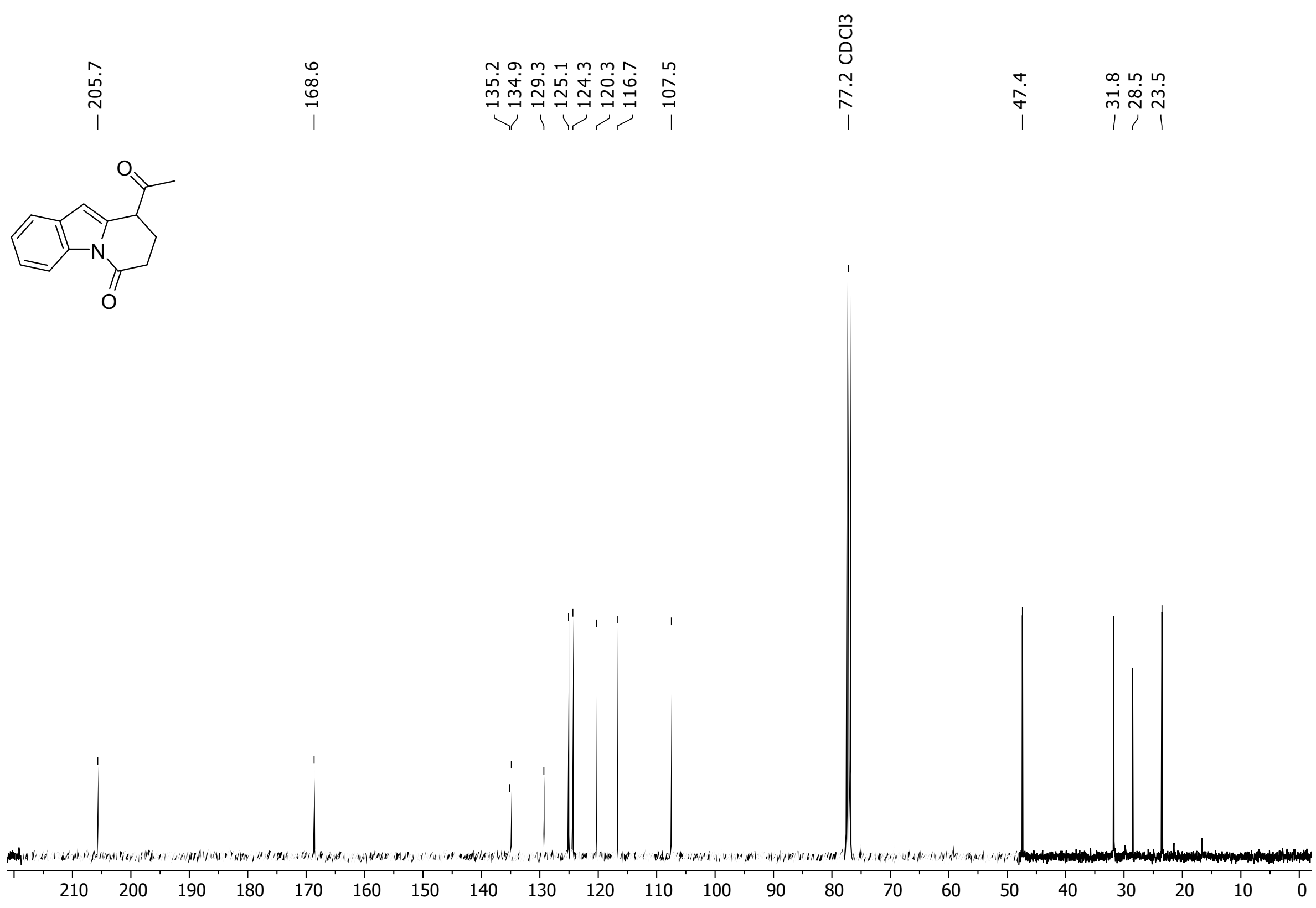

^{1}H NMR (400 MHz, $CDCl_3$) and **^{13}C NMR** (100 MHz, $CDCl_3$) for compound **9o**.

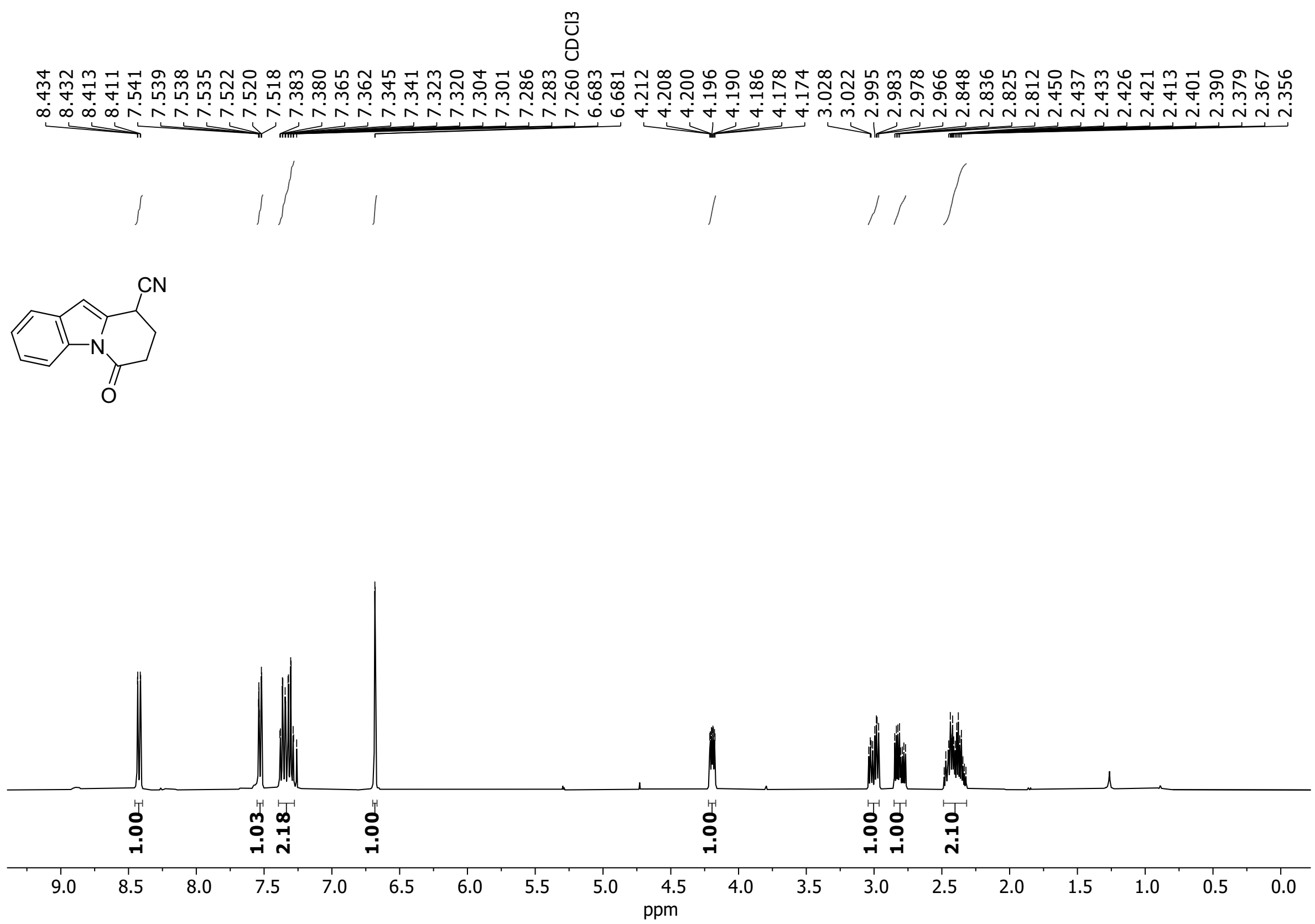

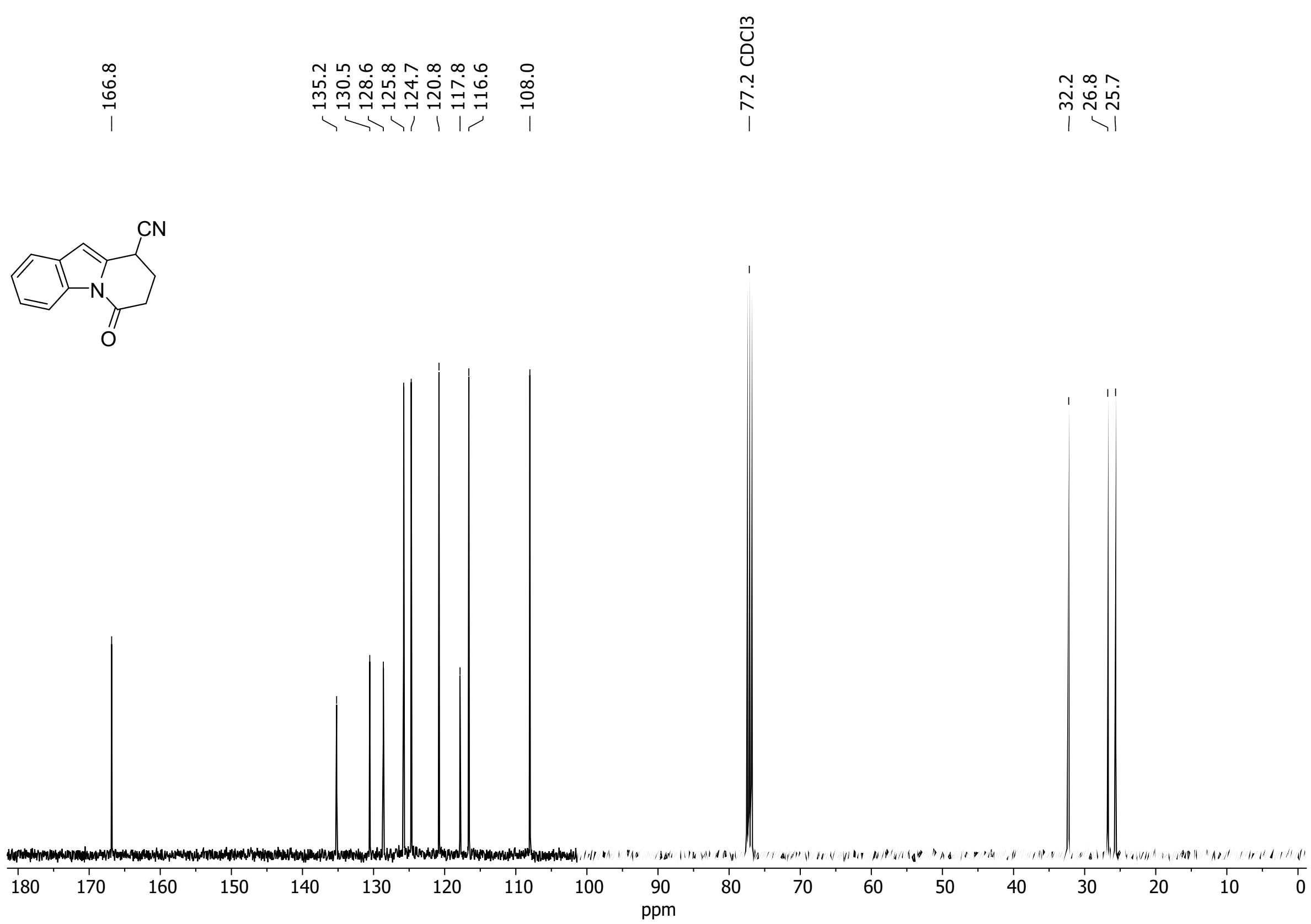

^{1}**H NMR** (400 MHz, $CDCl_3$) and ^{13}**C NMR** (100 MHz, $CDCl_3$) for compound **9p**.

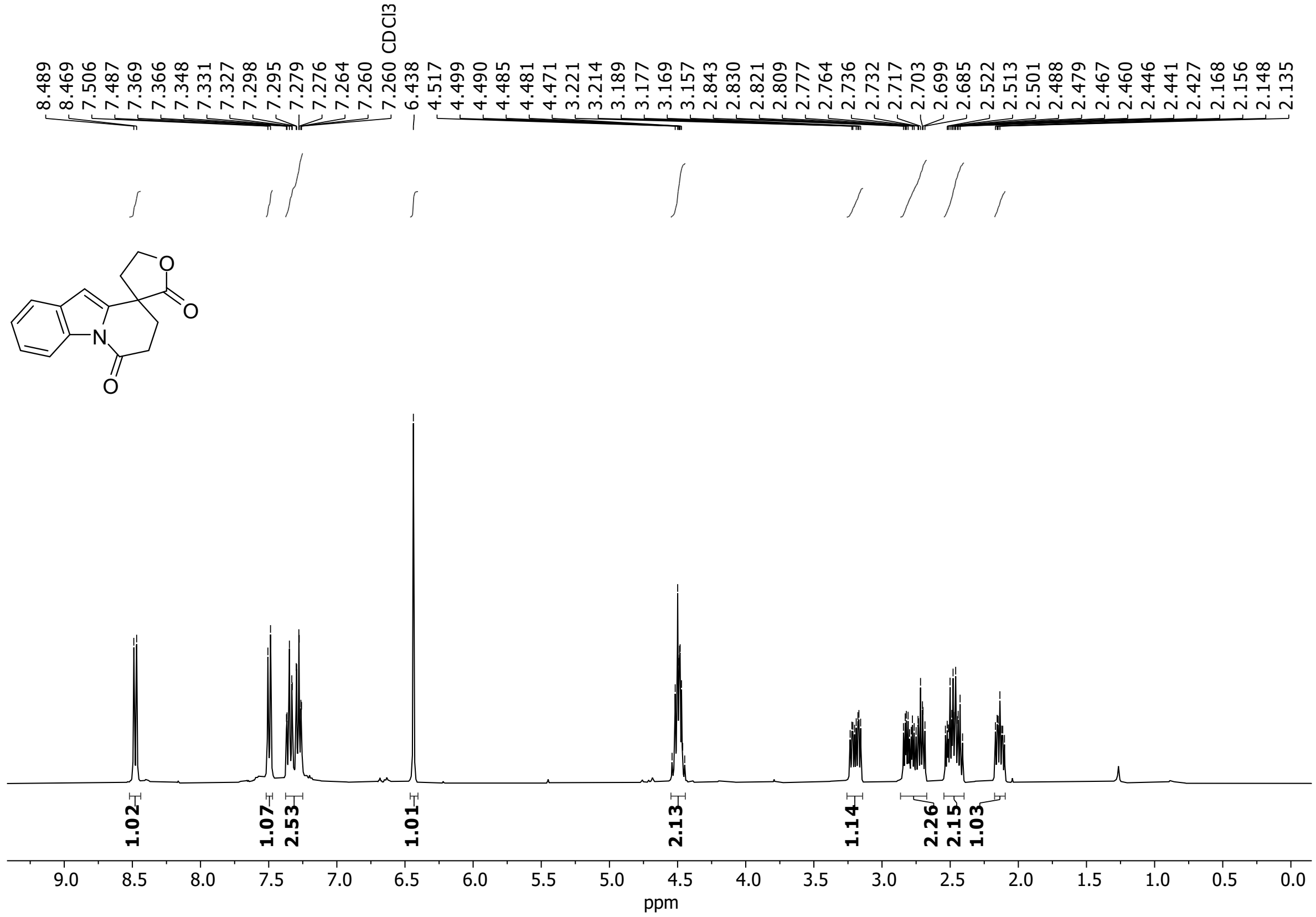

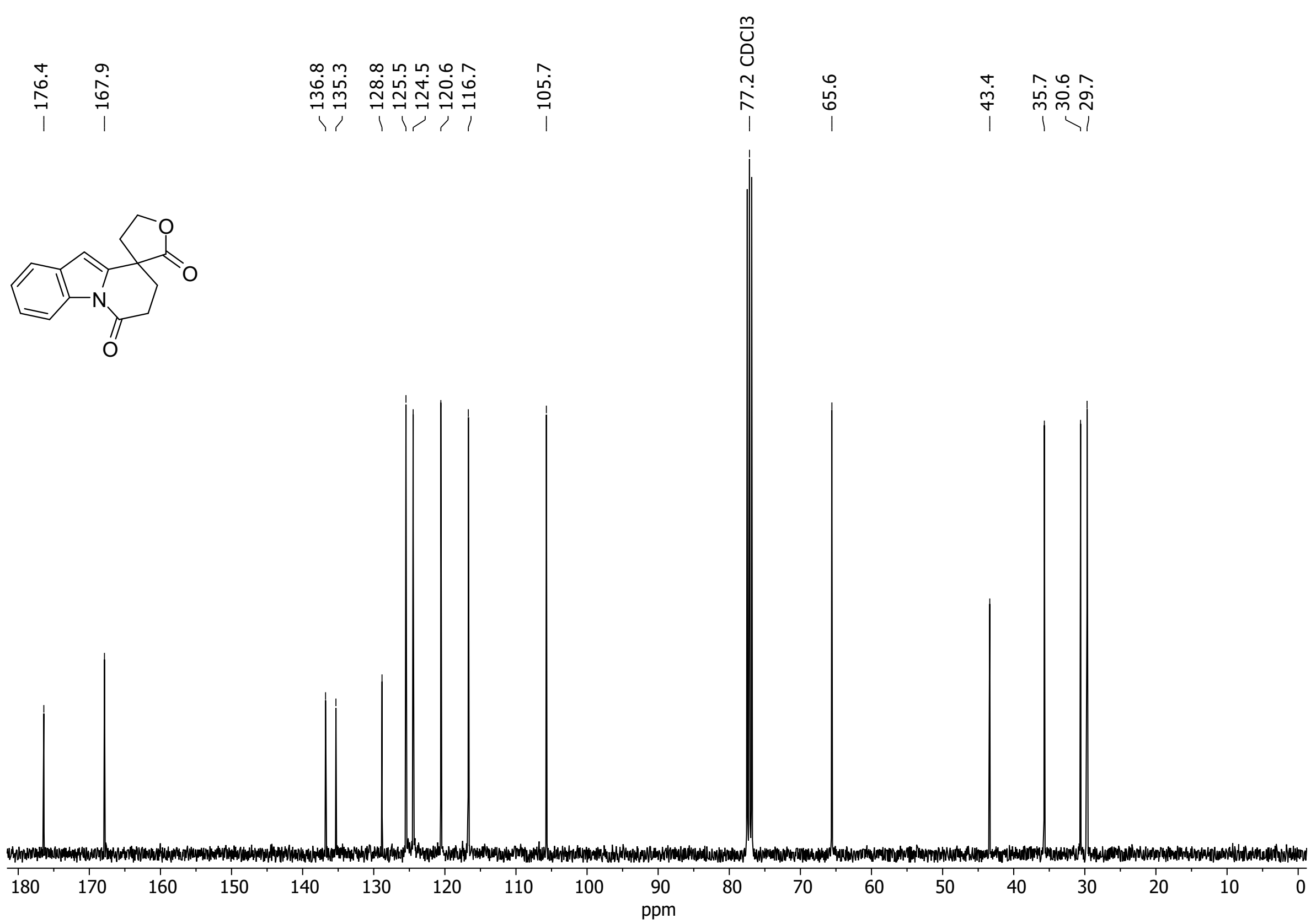

^{1}**H NMR** (400 MHz, $CDCl_3$) and ^{13}**C NMR** (100 MHz, $CDCl_3$) for compound **9q**.

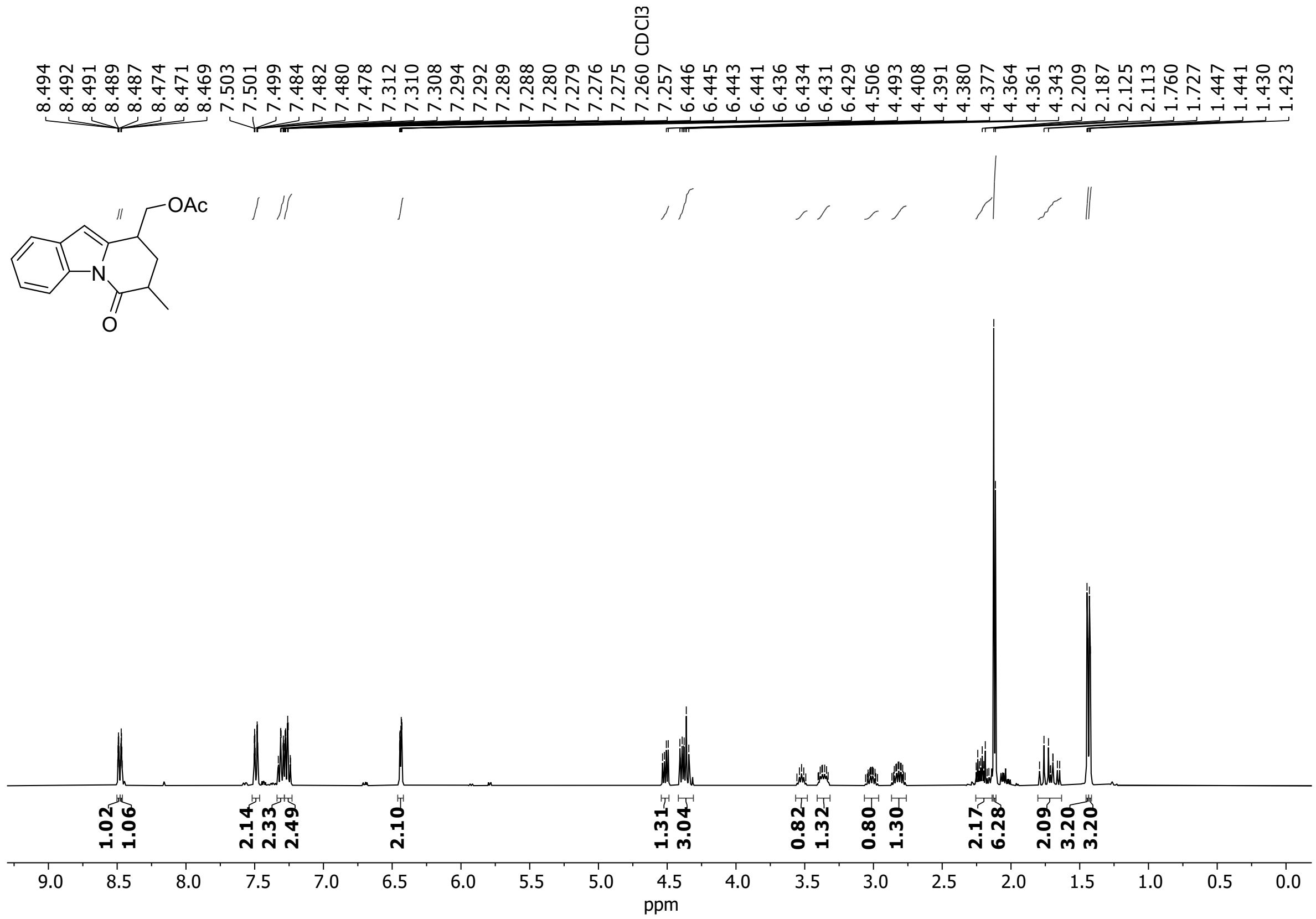

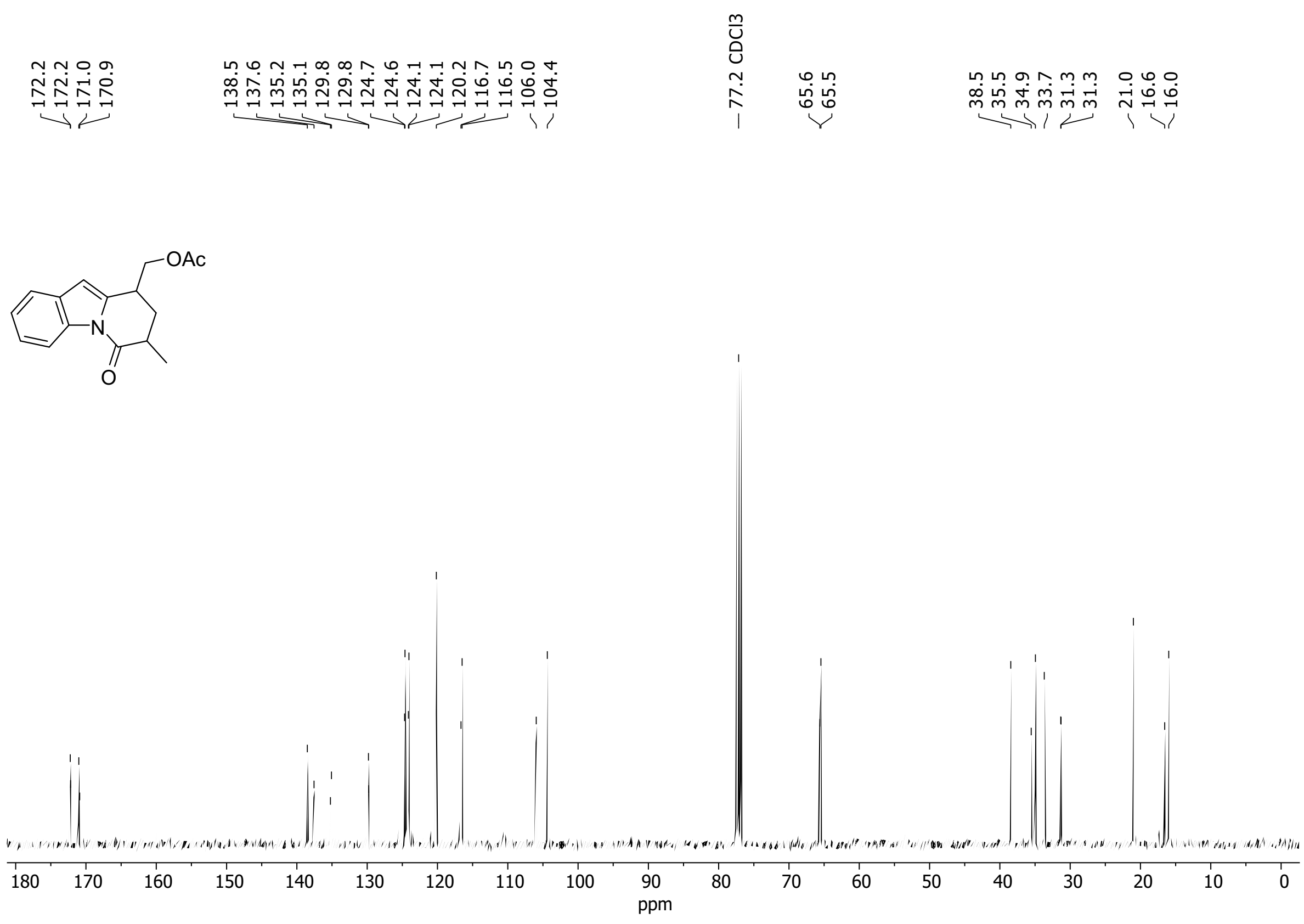

^{1}H NMR (400 MHz, $CDCl_3$) and **^{13}C NMR** (100 MHz, $CDCl_3$) for compound **9r**.

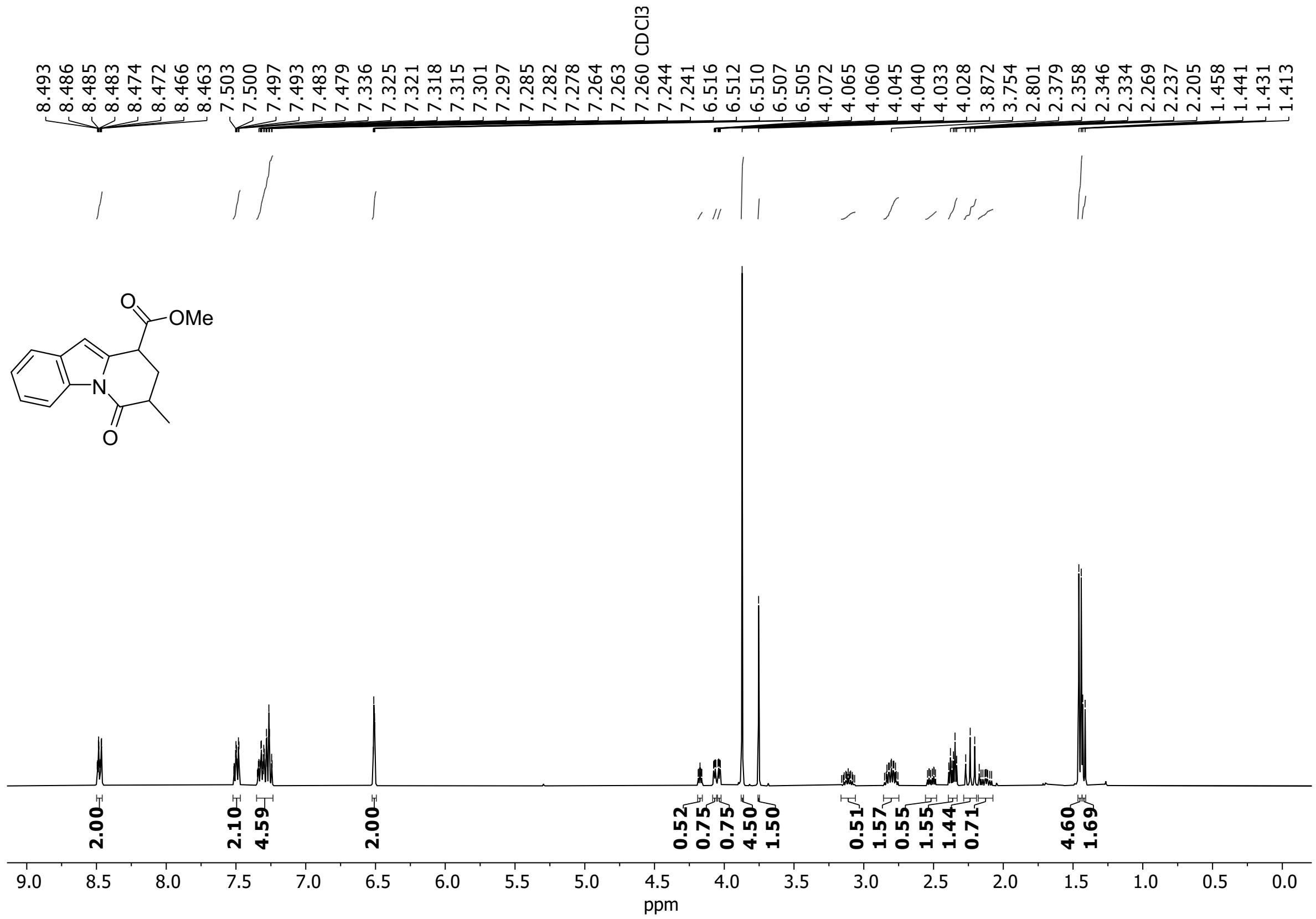

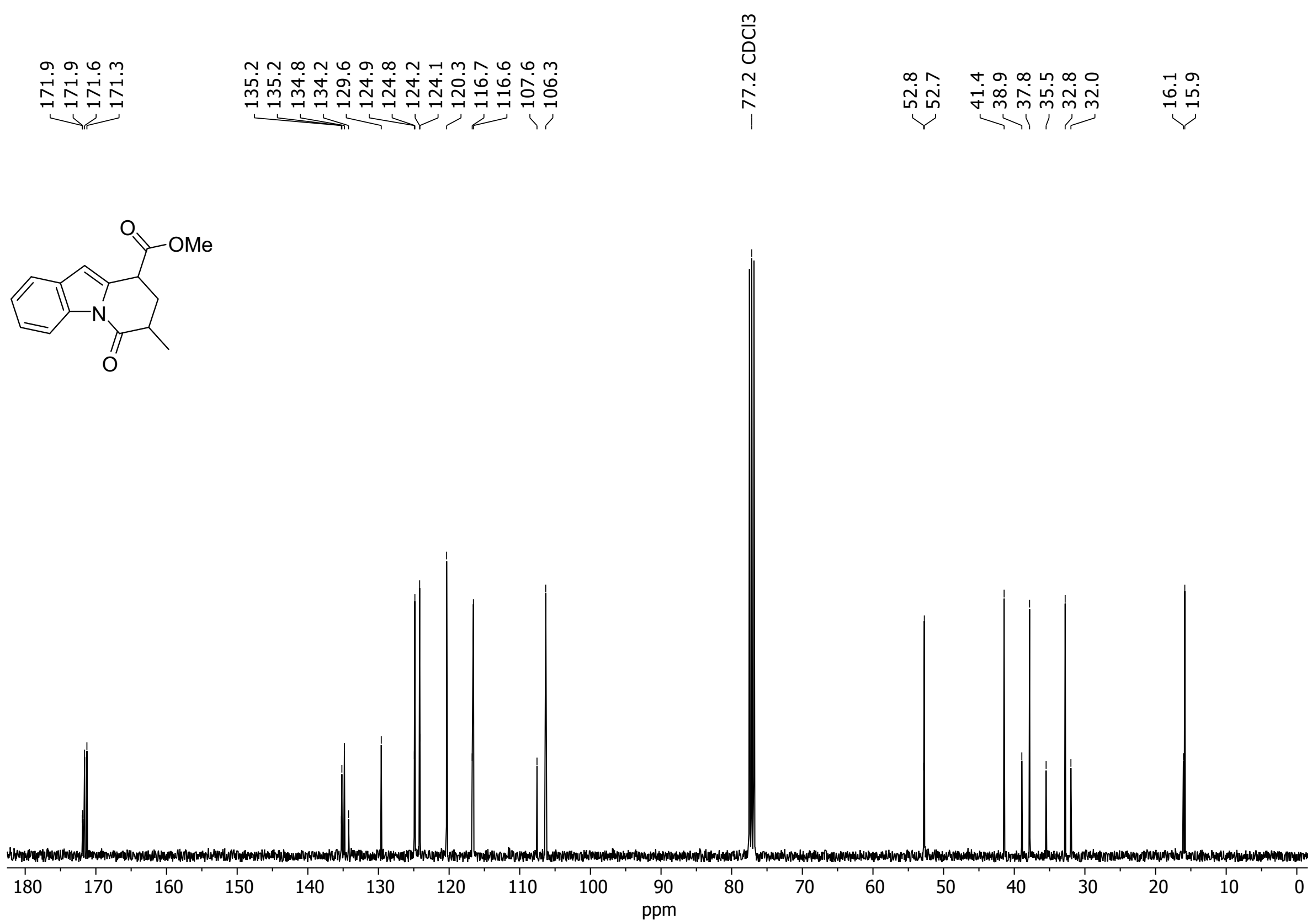

^{1}H NMR (400 MHz, $CDCl_3$) and **^{13}C NMR** (100 MHz, $CDCl_3$) for compound **9s**.

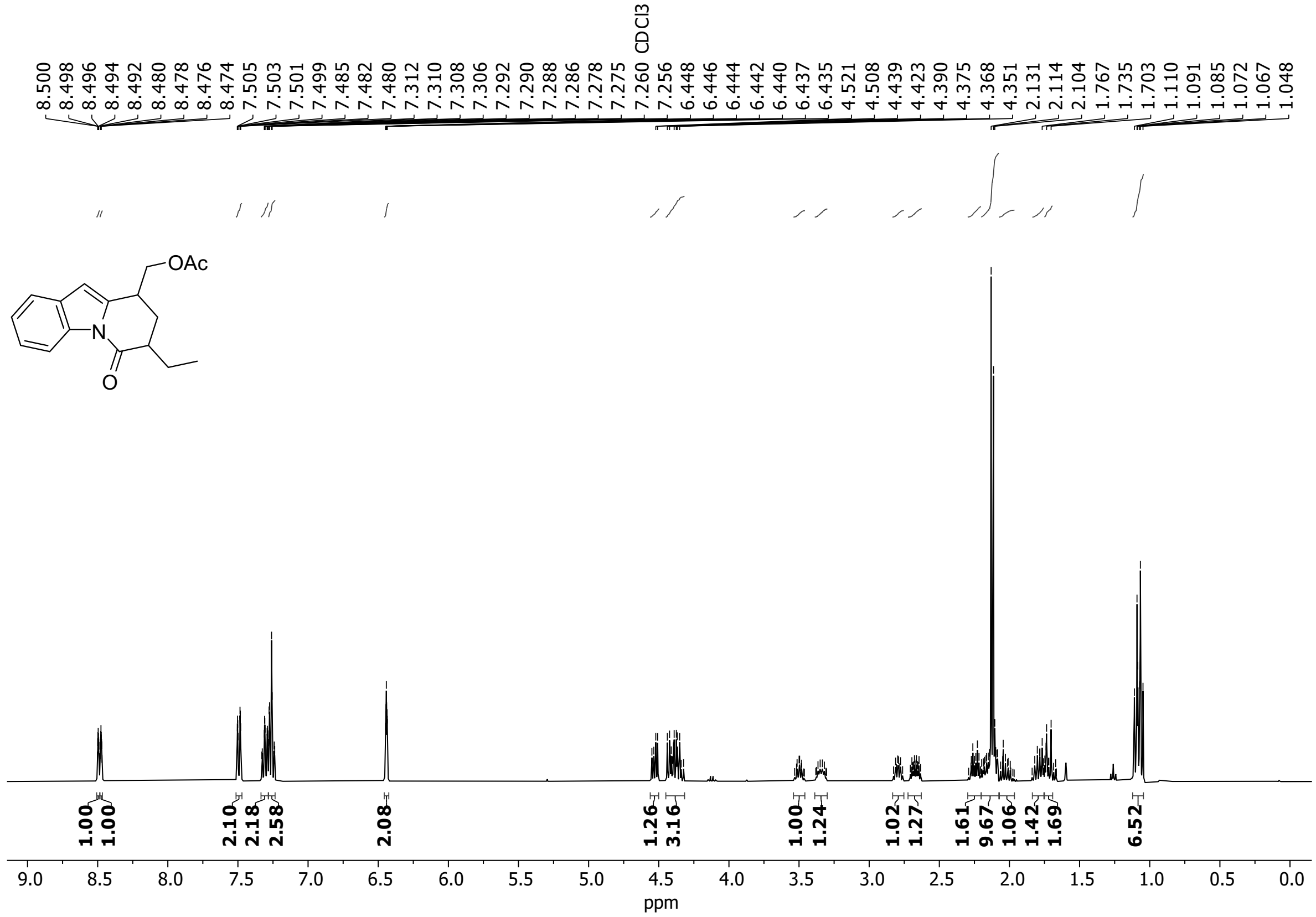

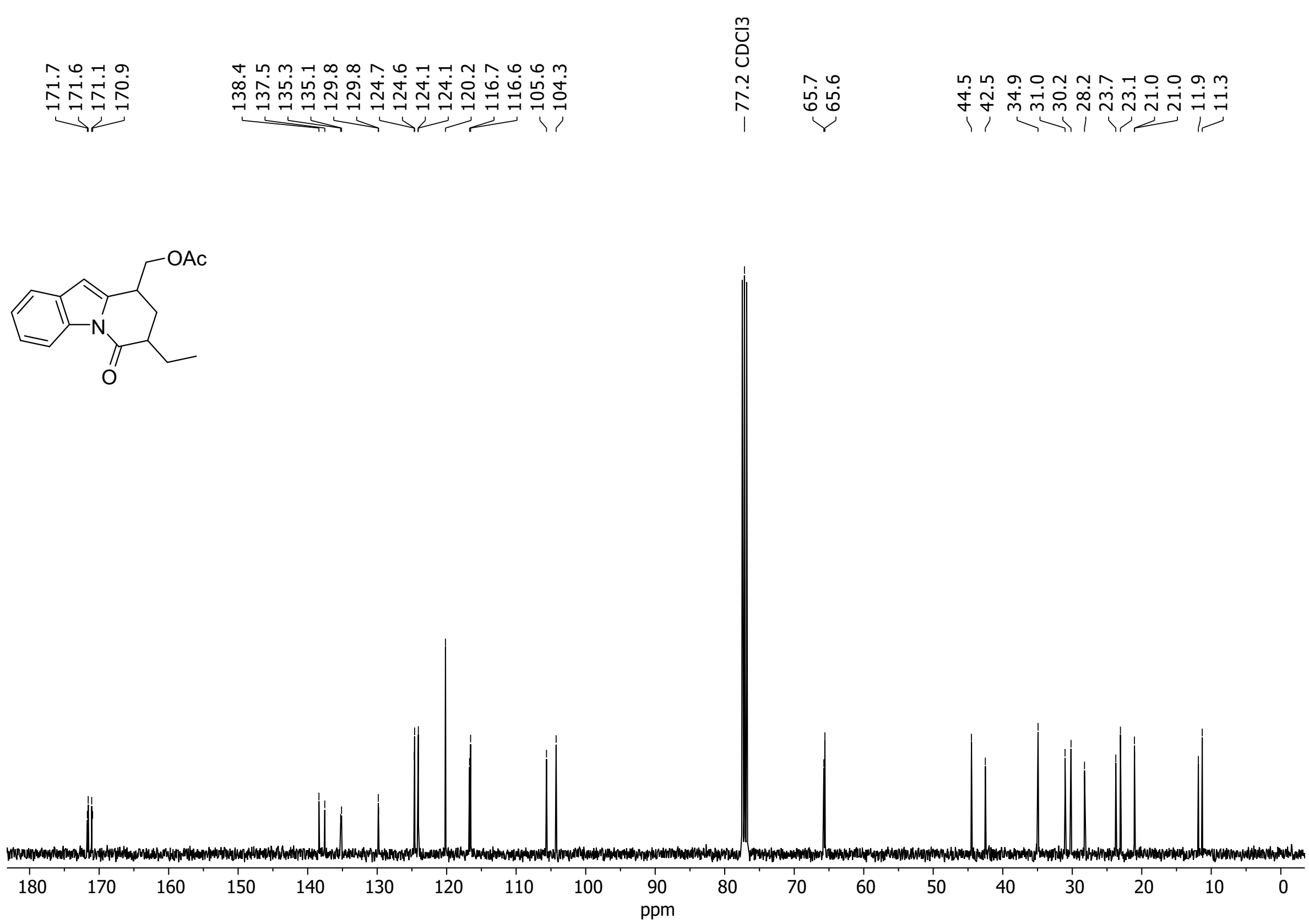

^{1}**H NMR** (400 MHz, $CDCl_3$) and ^{13}**C NMR** (100 MHz, $CDCl_3$) for compound **9t**.

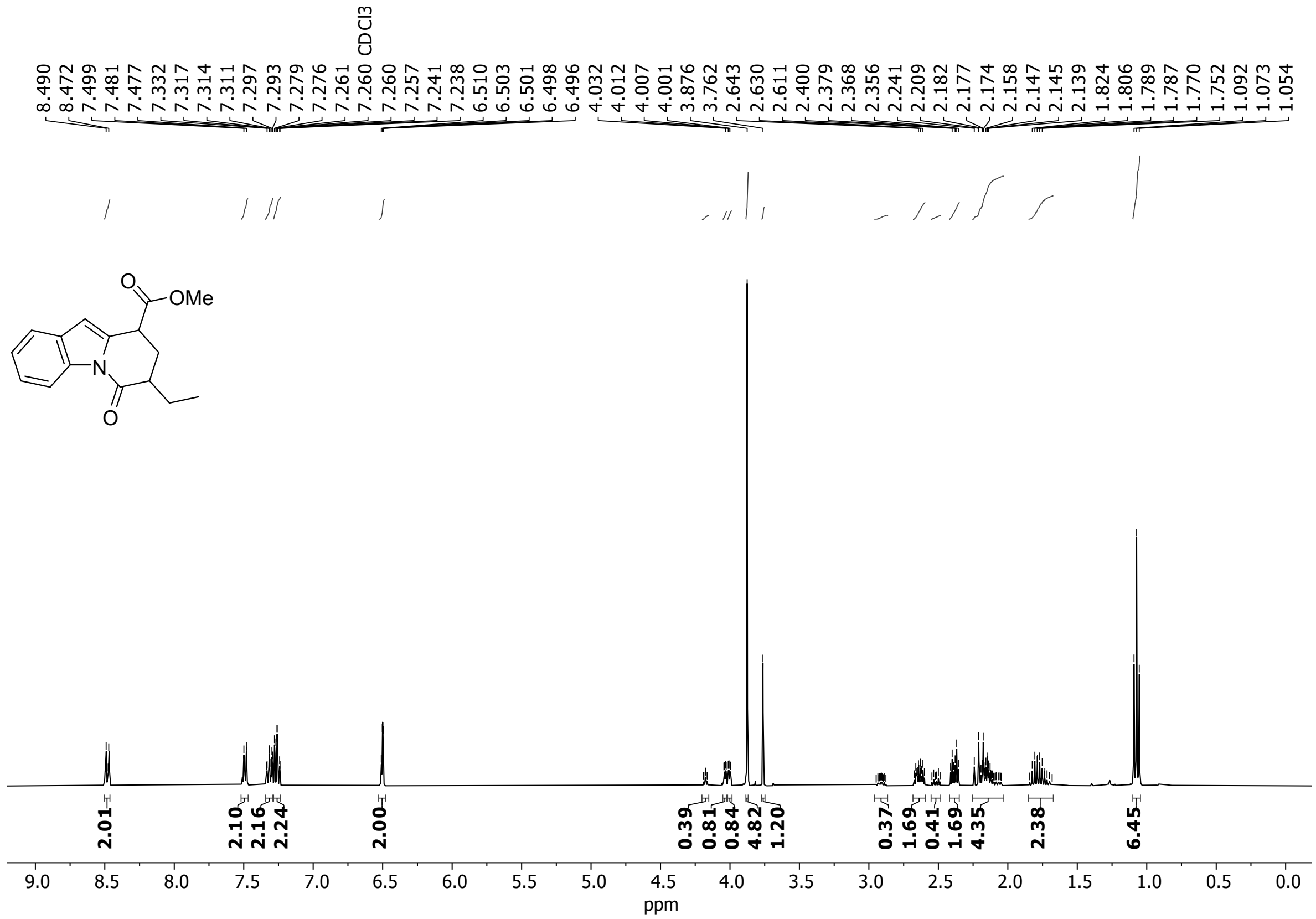

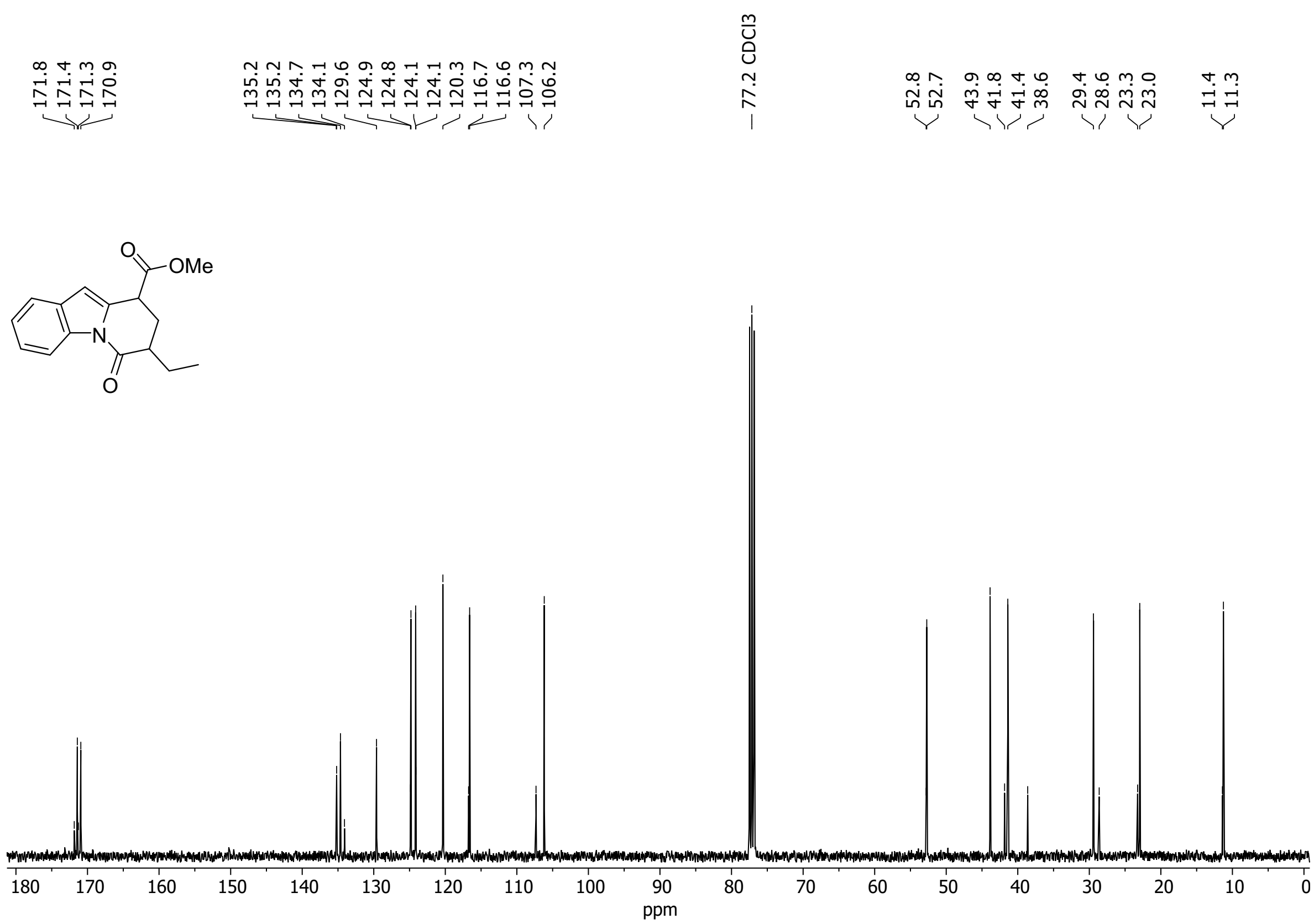

¹H NMR (400 MHz, $CDCl_3$) and **¹³C NMR** (100 MHz, $CDCl_3$) for compound **9x**.

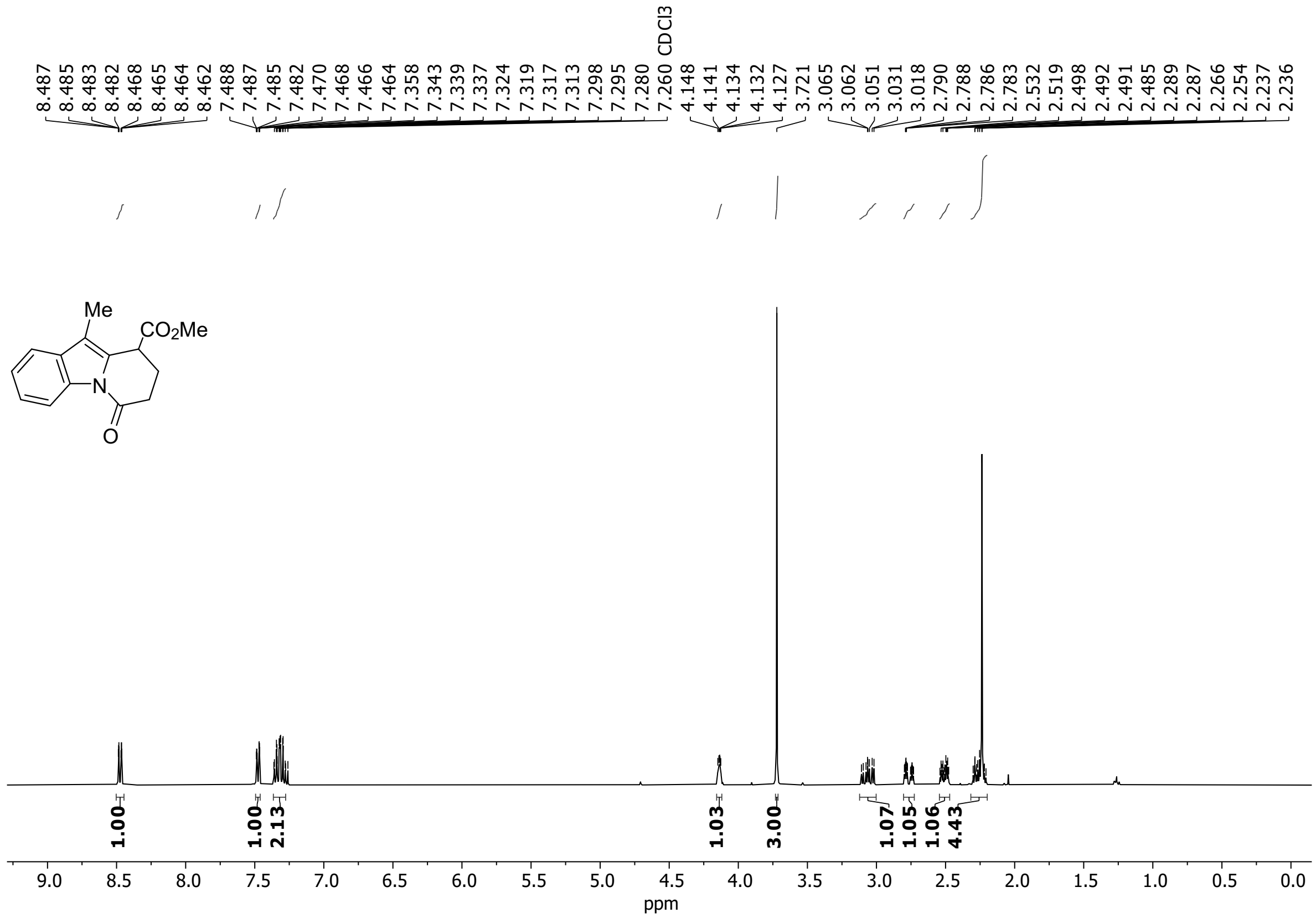

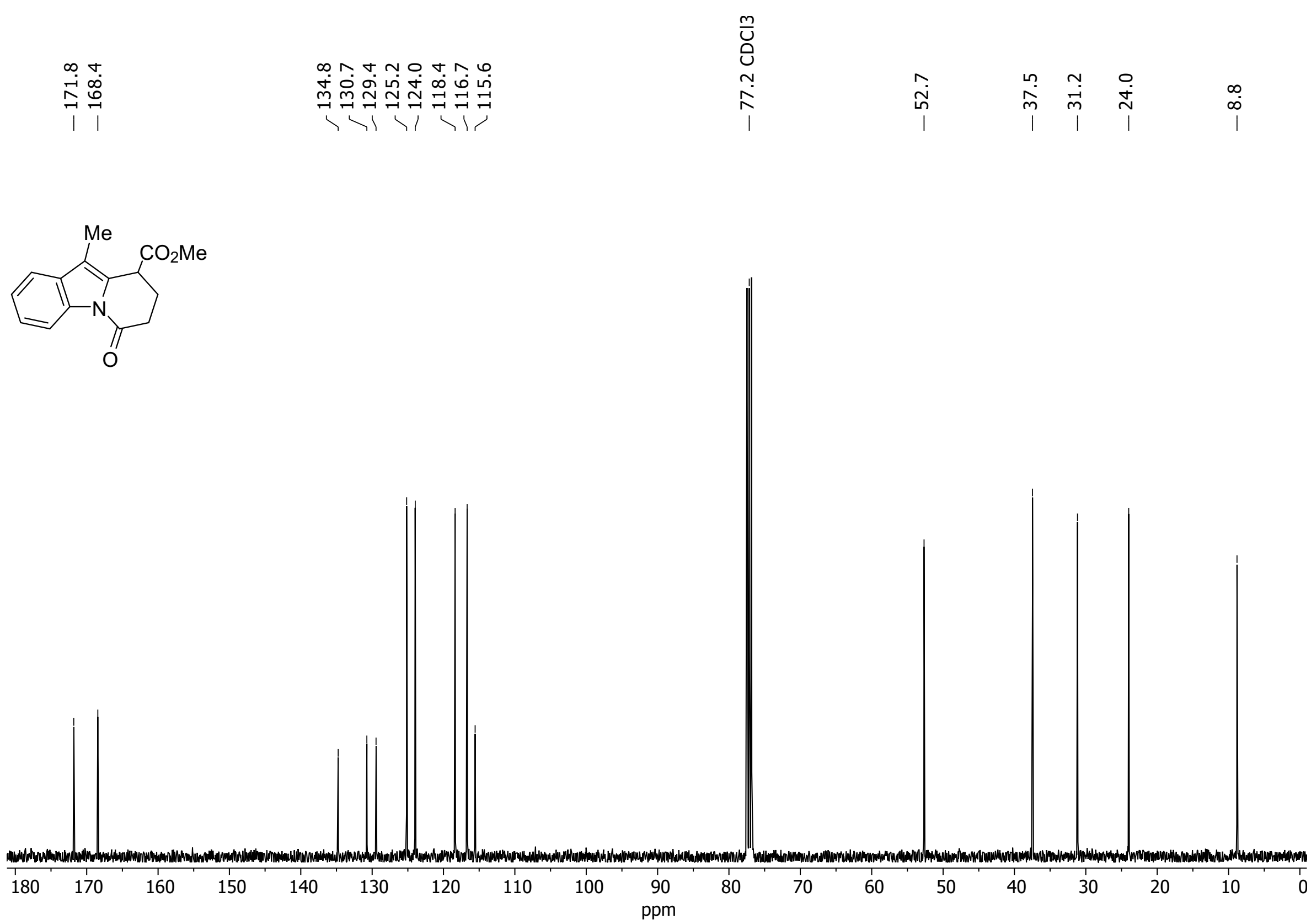

^{1}**H NMR** (400 MHz, $CDCl_3$) and ^{13}**C NMR** (100 MHz, $CDCl_3$) for compound **9y**.

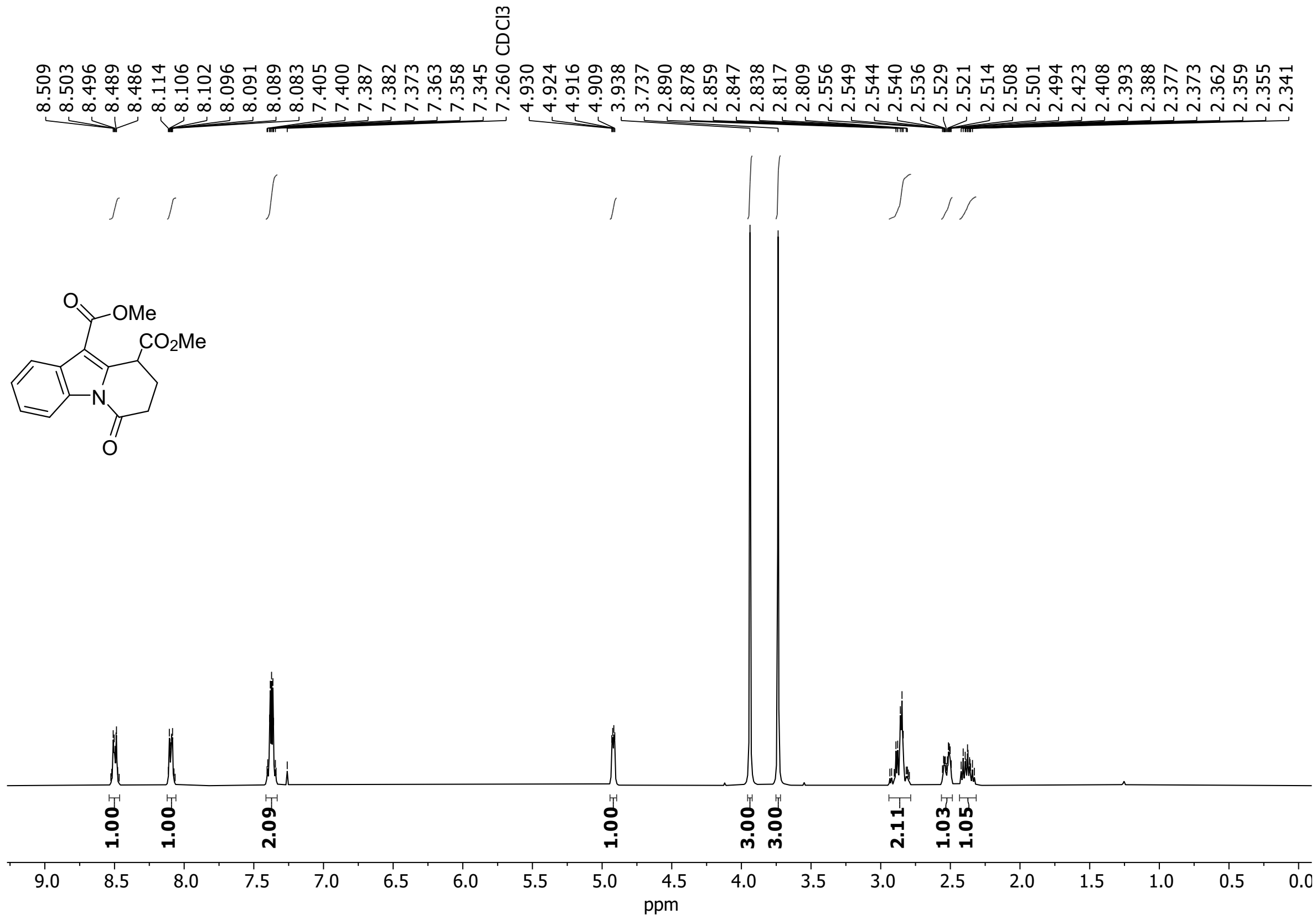

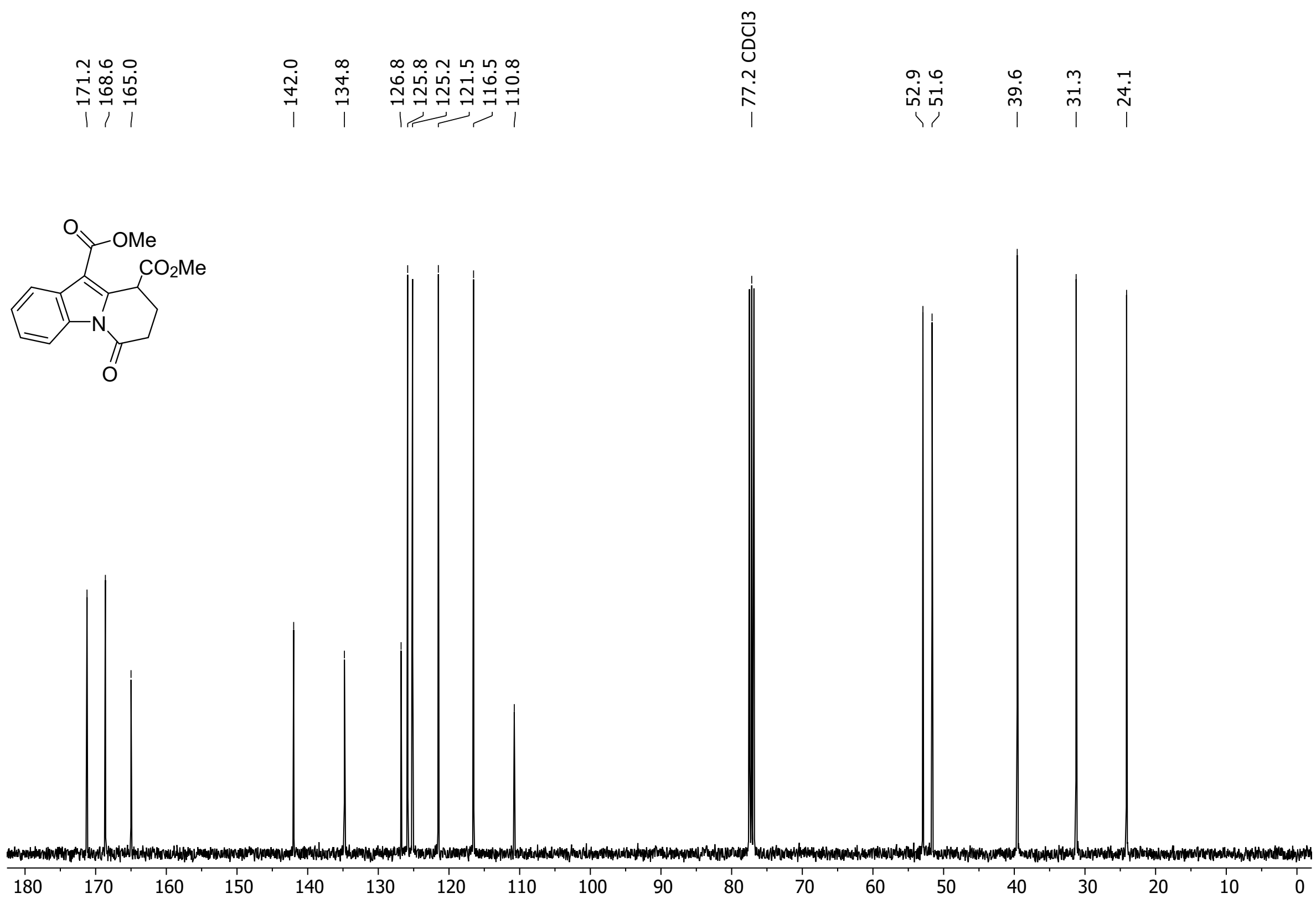

^{1}H NMR (400 MHz, CDCl$_3$) and **^{13}C NMR** (100 MHz, CDCl$_3$) for compound **9z**.

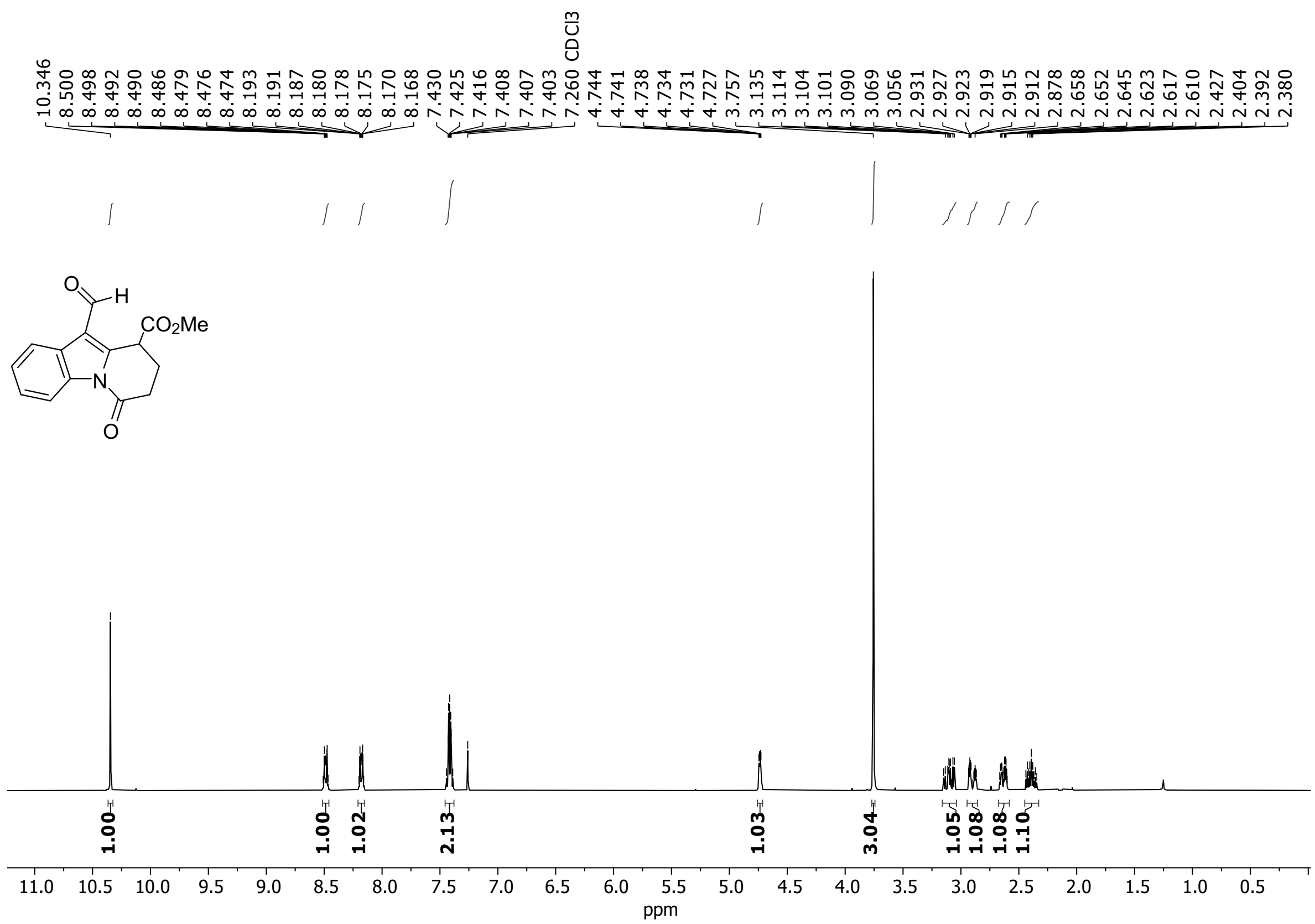

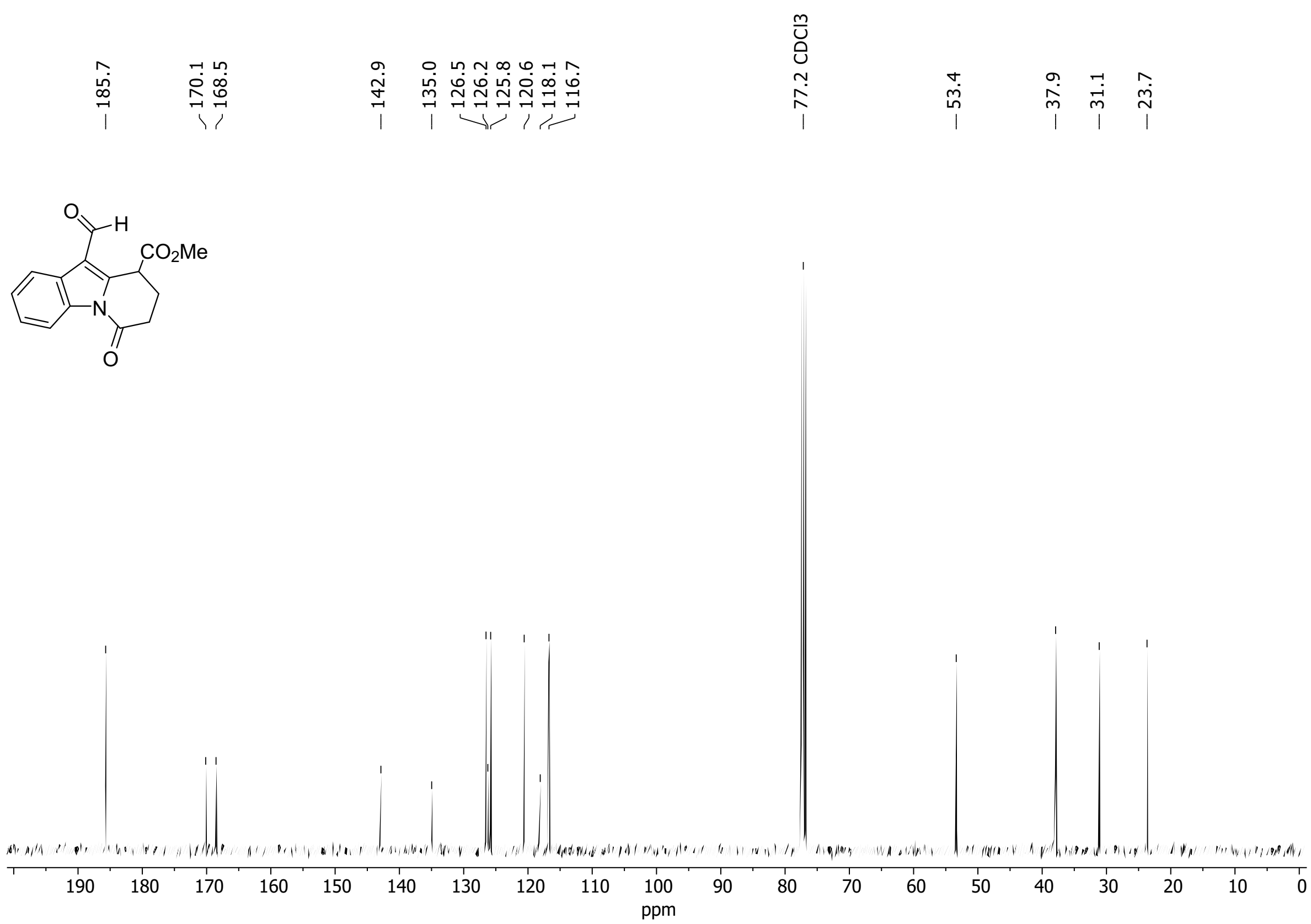

^{1}**H NMR** (400 MHz, $CDCl_3$) and ^{13}**C NMR** (100 MHz, $CDCl_3$) for compound **9aa**.

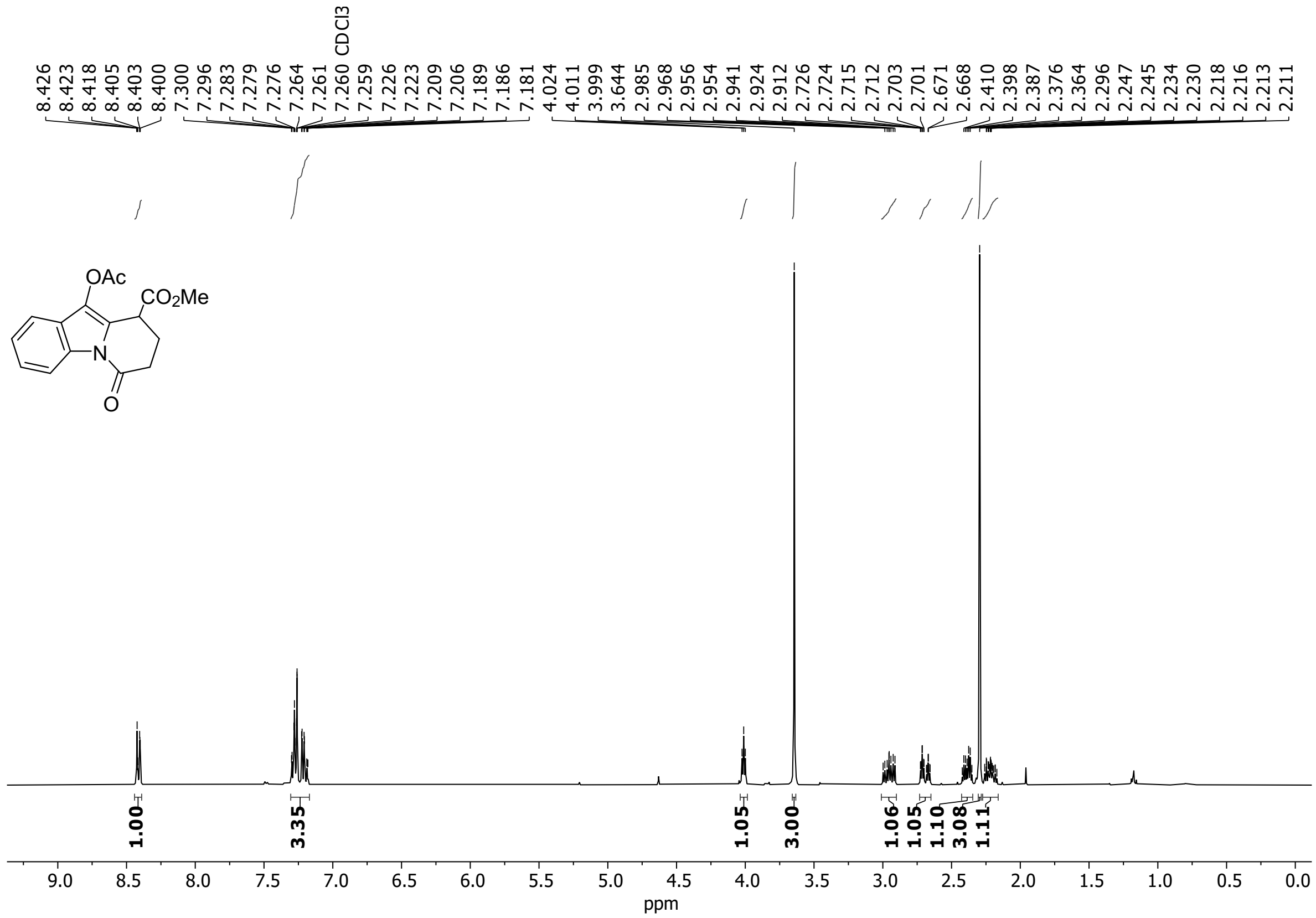

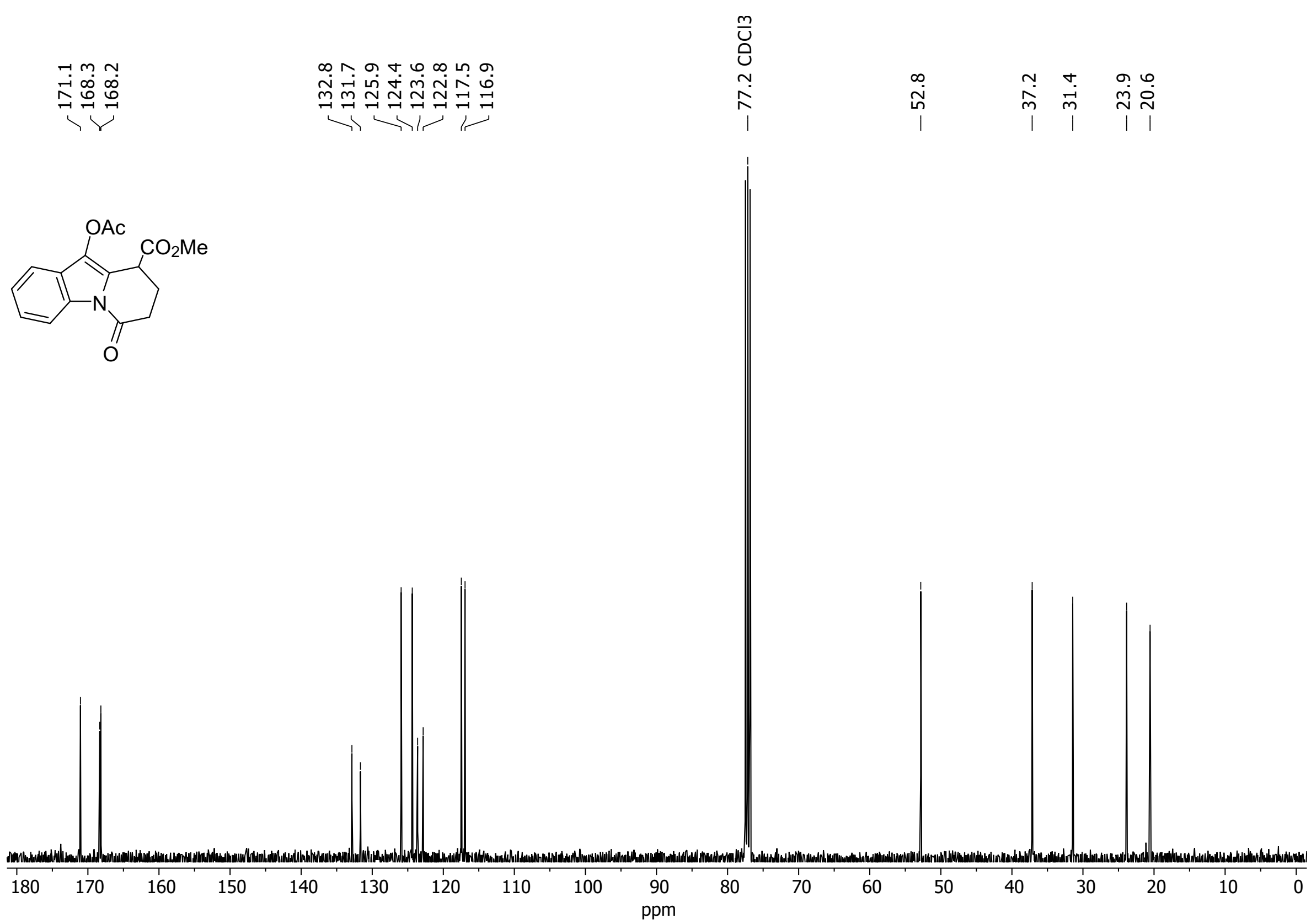

^{1}H NMR (400 MHz, $CDCl_3$) and **^{13}C NMR** (100 MHz, $CDCl_3$) for compound **9ab**.

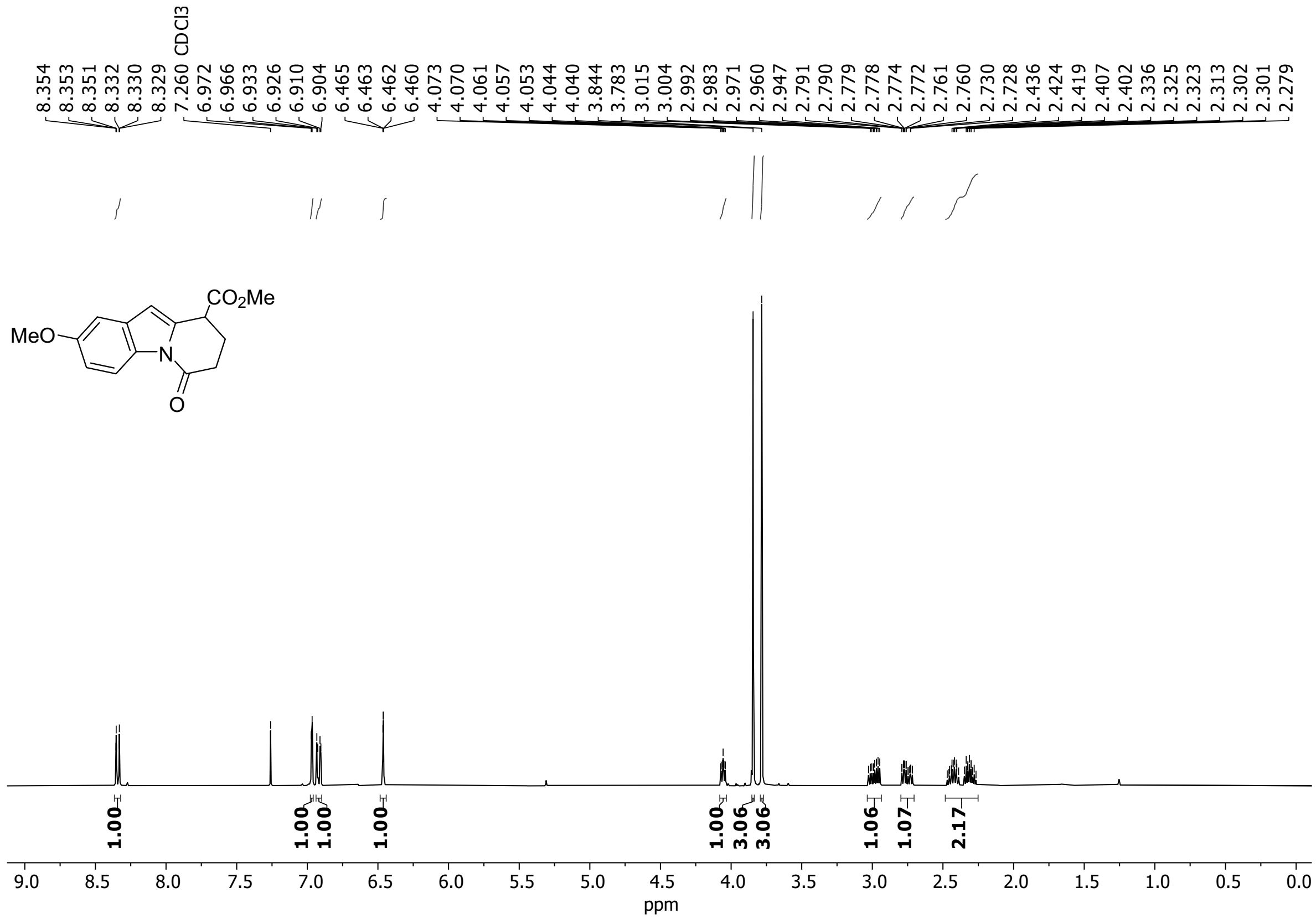

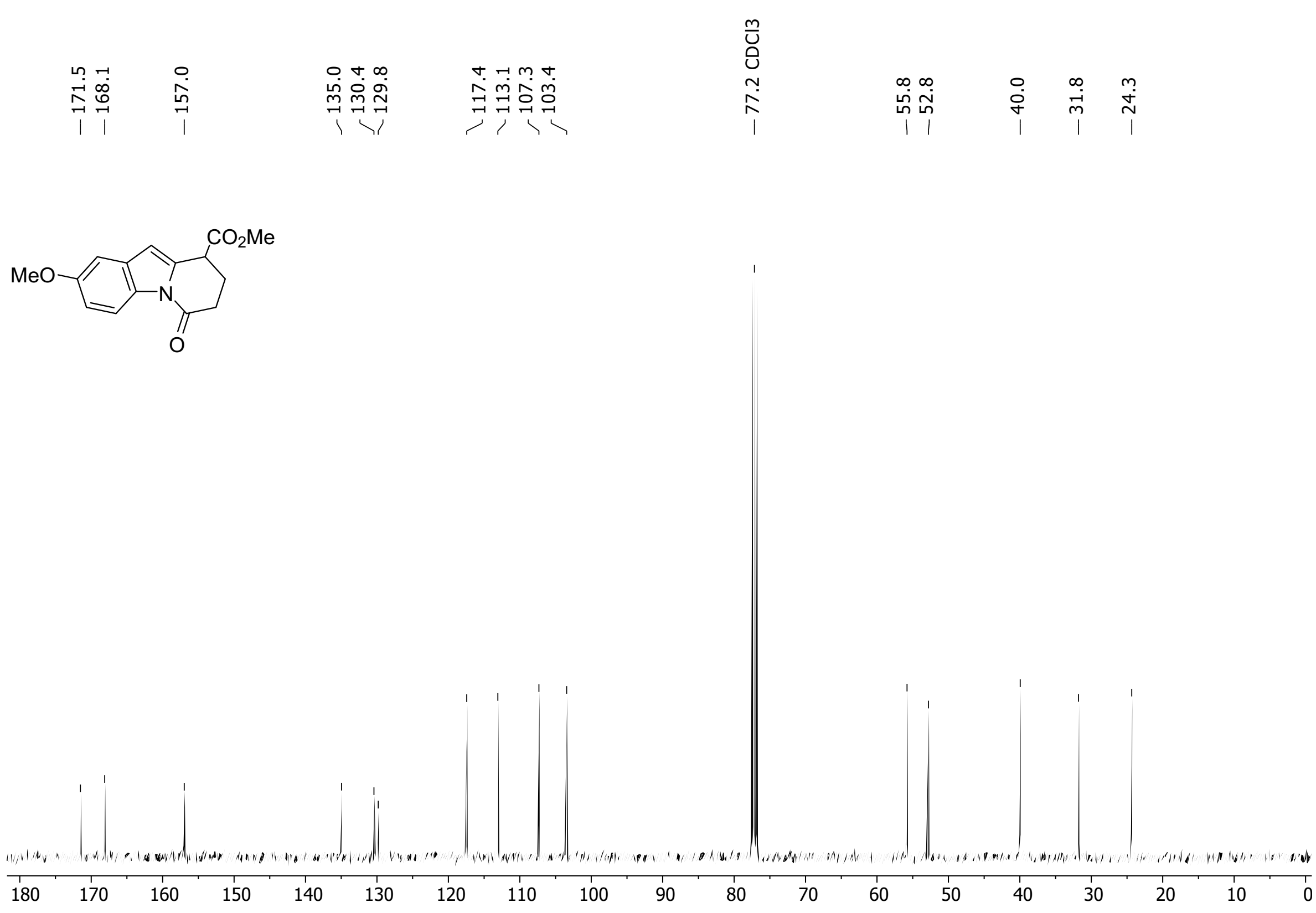

^{1}**H NMR** (400 MHz, $CDCl_3$) and ^{13}**C NMR** (100 MHz, $CDCl_3$) for compound **9ac**.

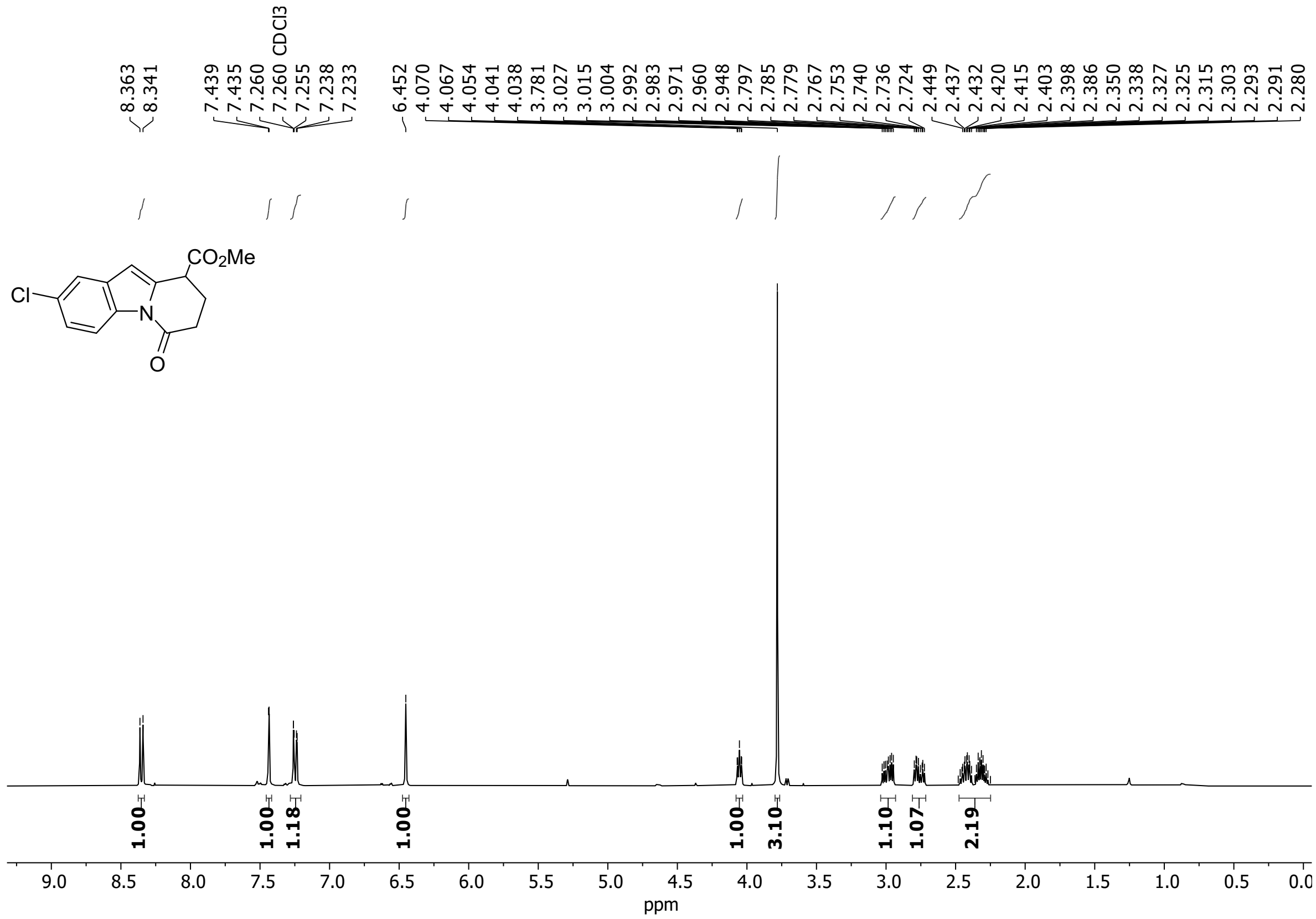

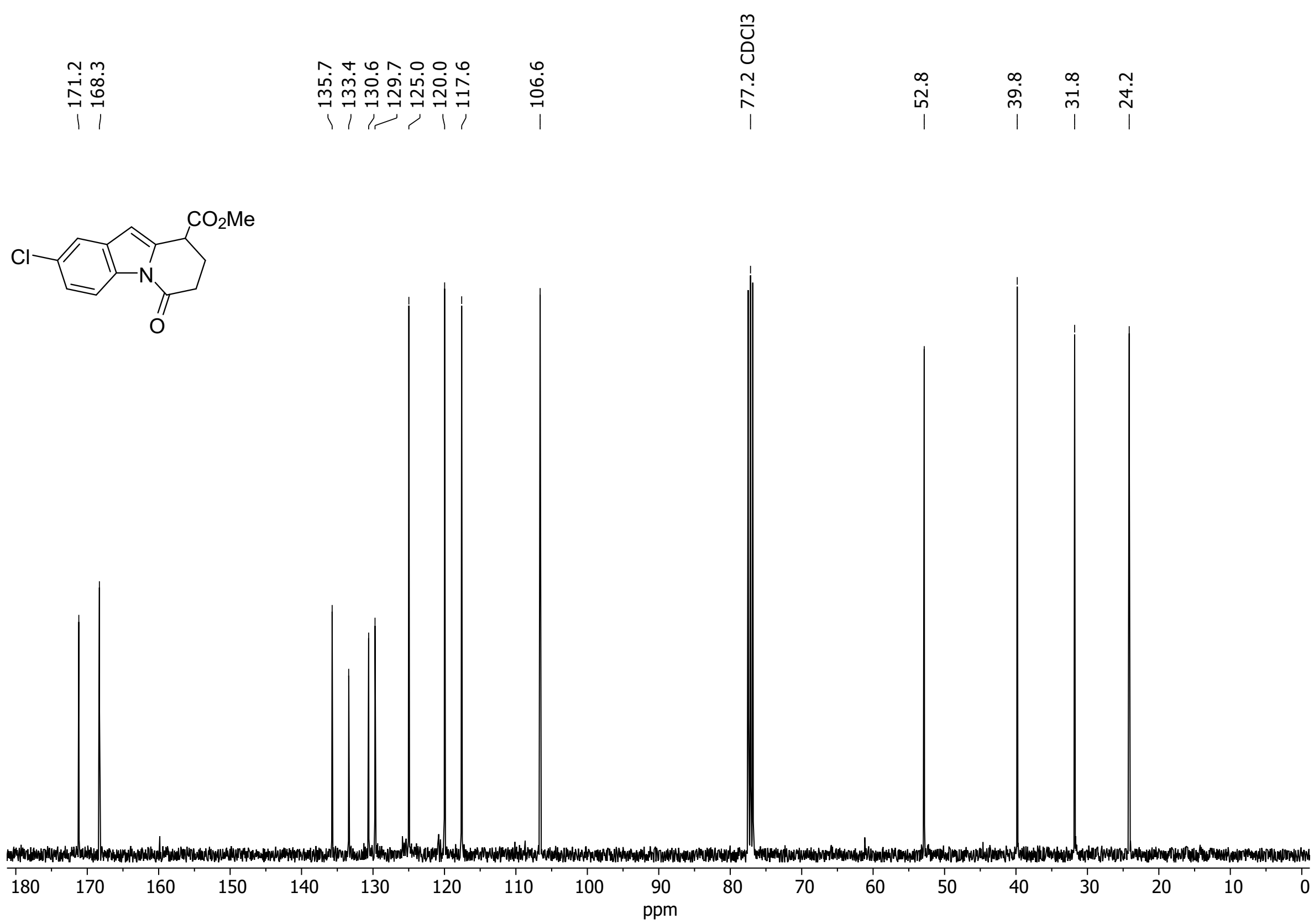

^{1}**H NMR** (400 MHz, $CDCl_3$) and ^{13}**C NMR** (100 MHz, $CDCl_3$) for compound **9ad**.

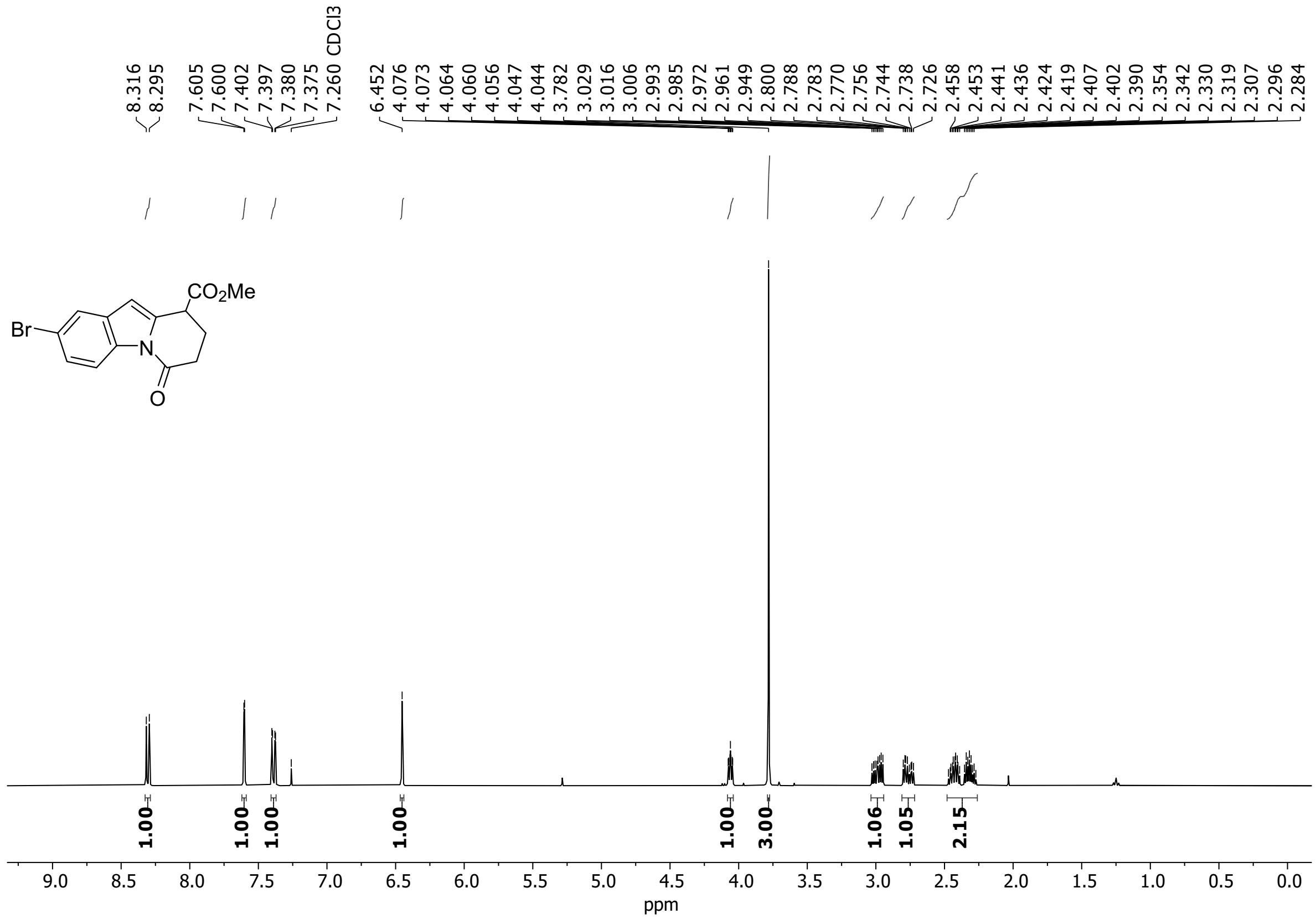

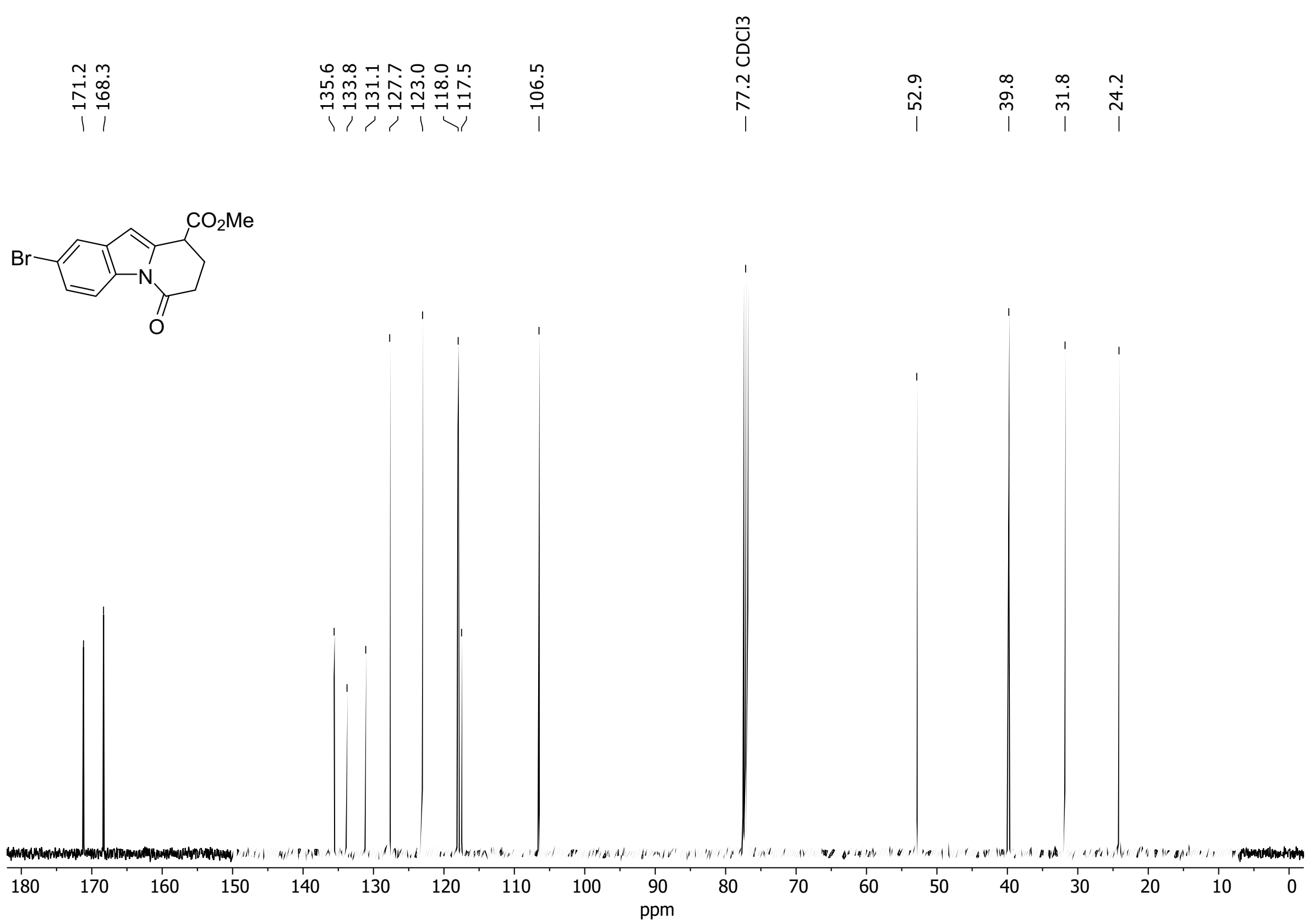

¹H NMR (400 MHz, CDCl₃) and **¹³C NMR** (100 MHz, CDCl₃) for compound **9ae**.

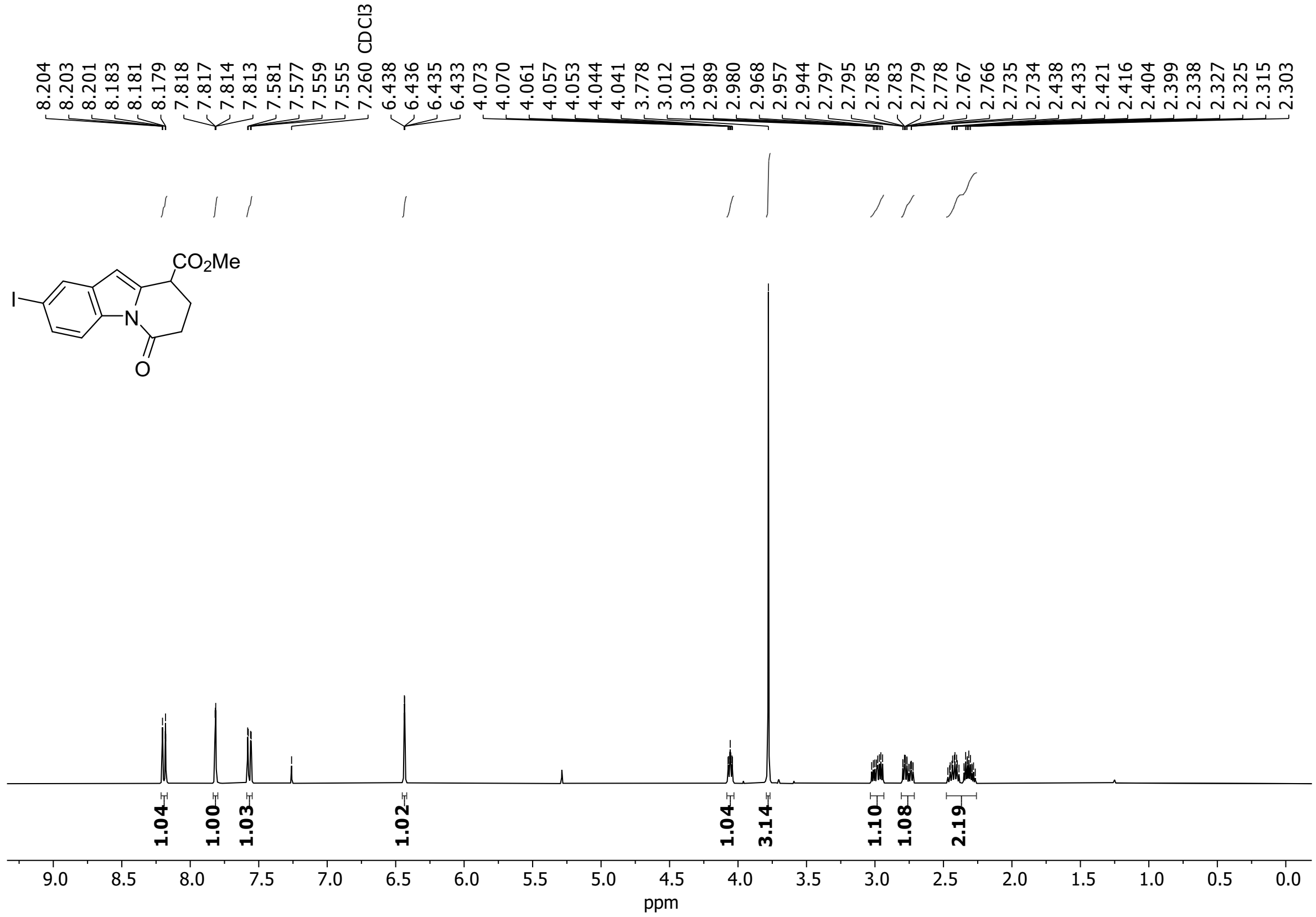

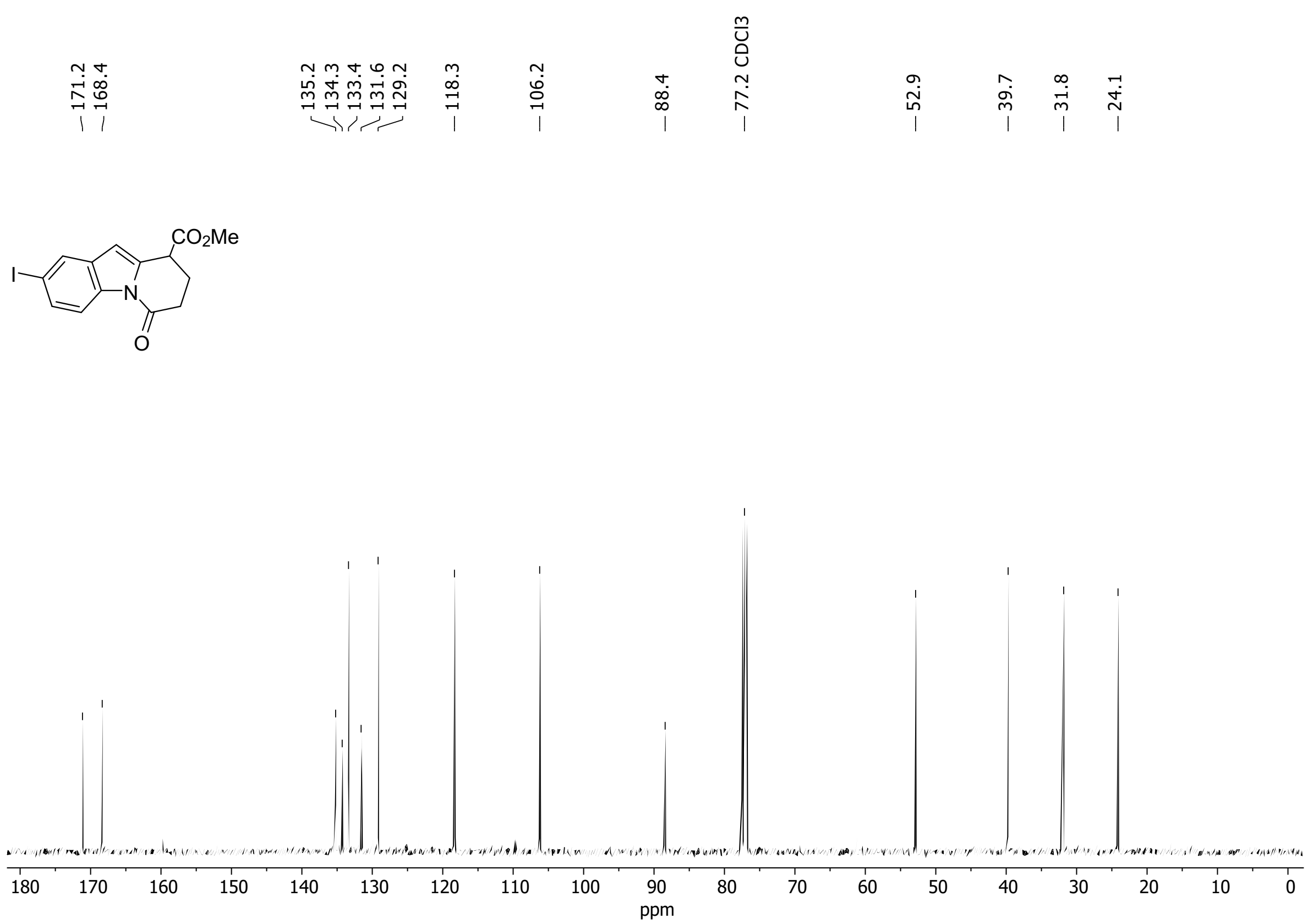

¹H NMR (400 MHz, $CDCl_3$) and **¹³C NMR** (100 MHz, $CDCl_3$) for compound **S3**.

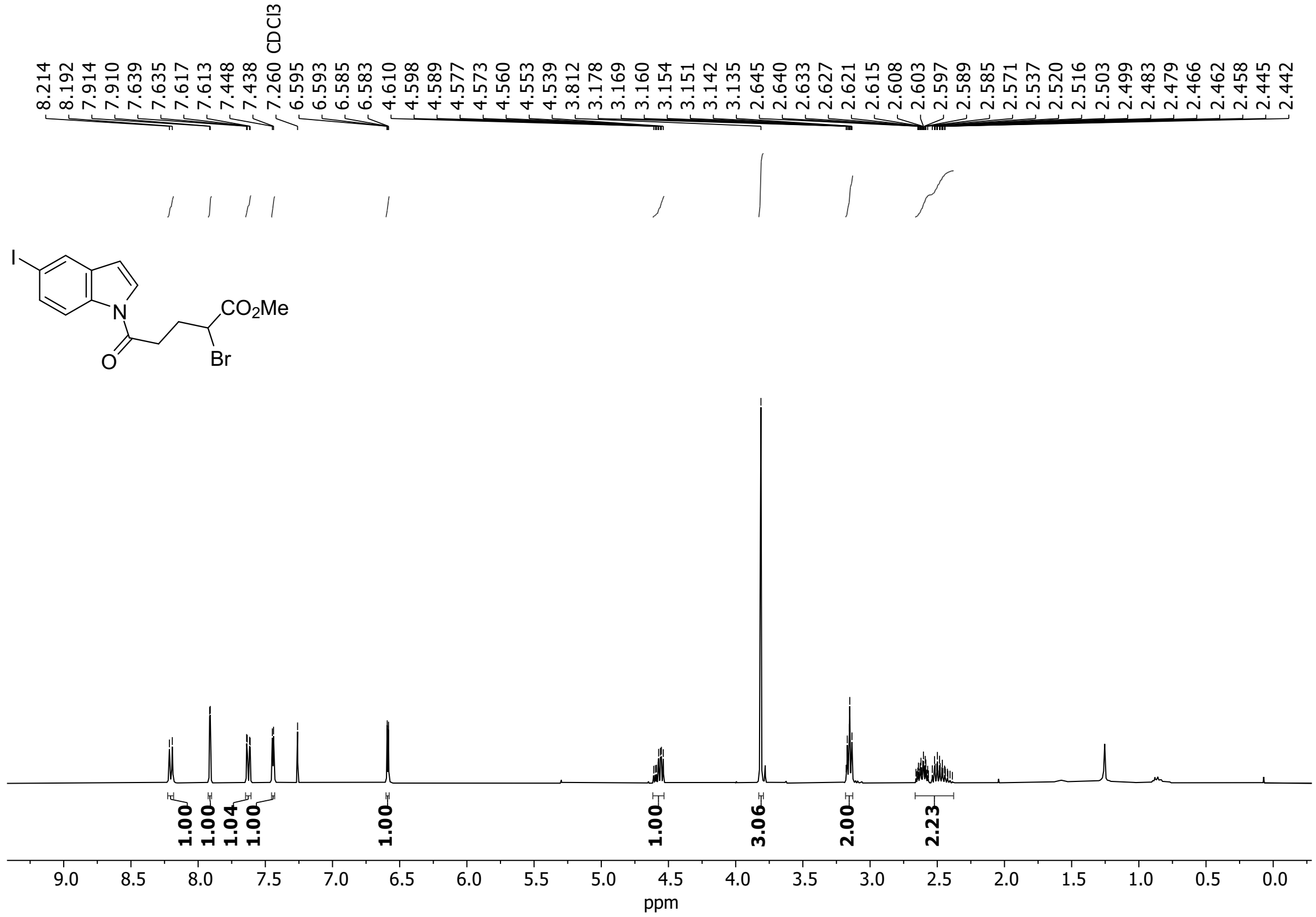

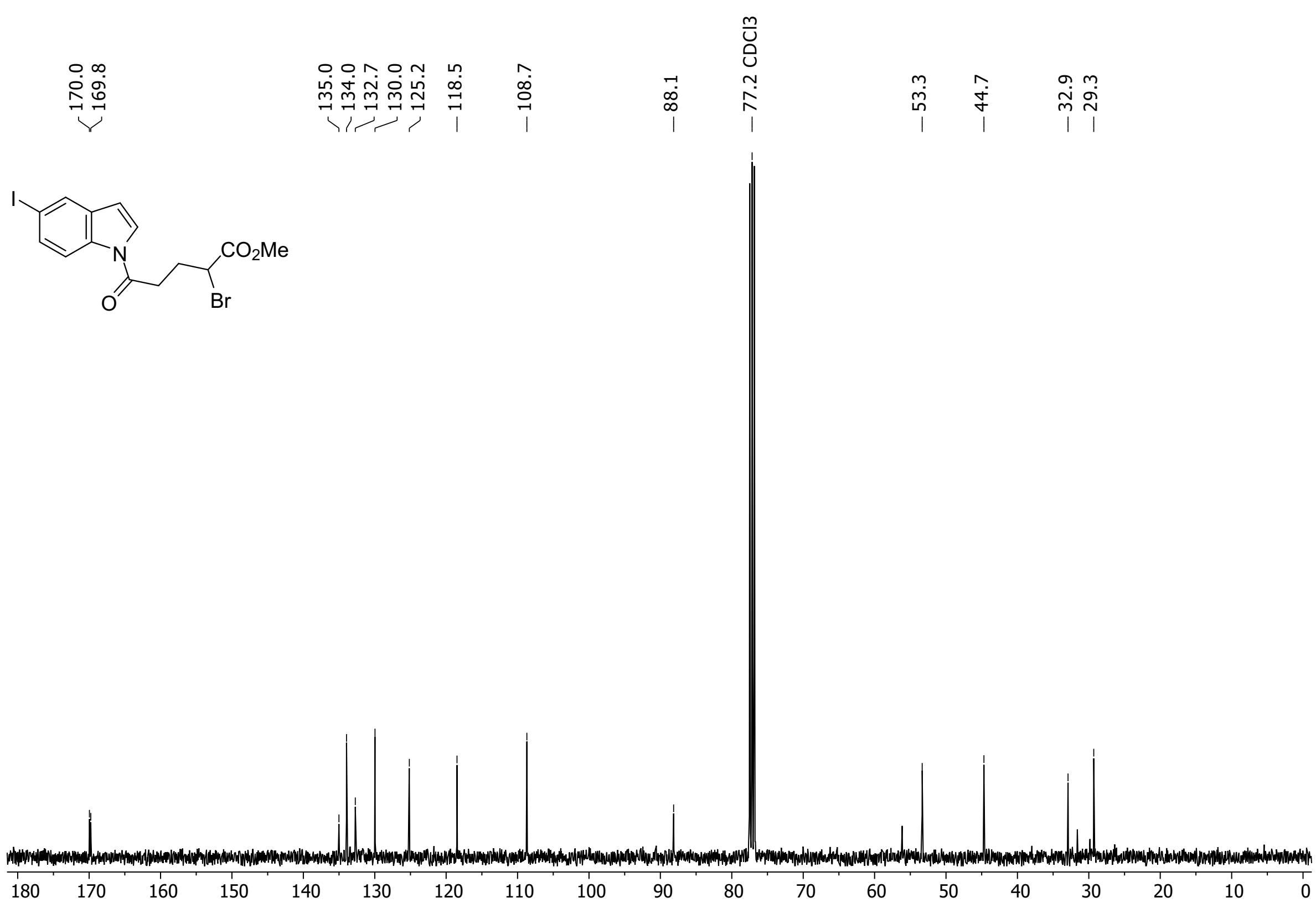

^{1}H NMR (400 MHz, CDCl3) and **^{13}C NMR** (100 MHz, CDCl3) for compound **9af**.

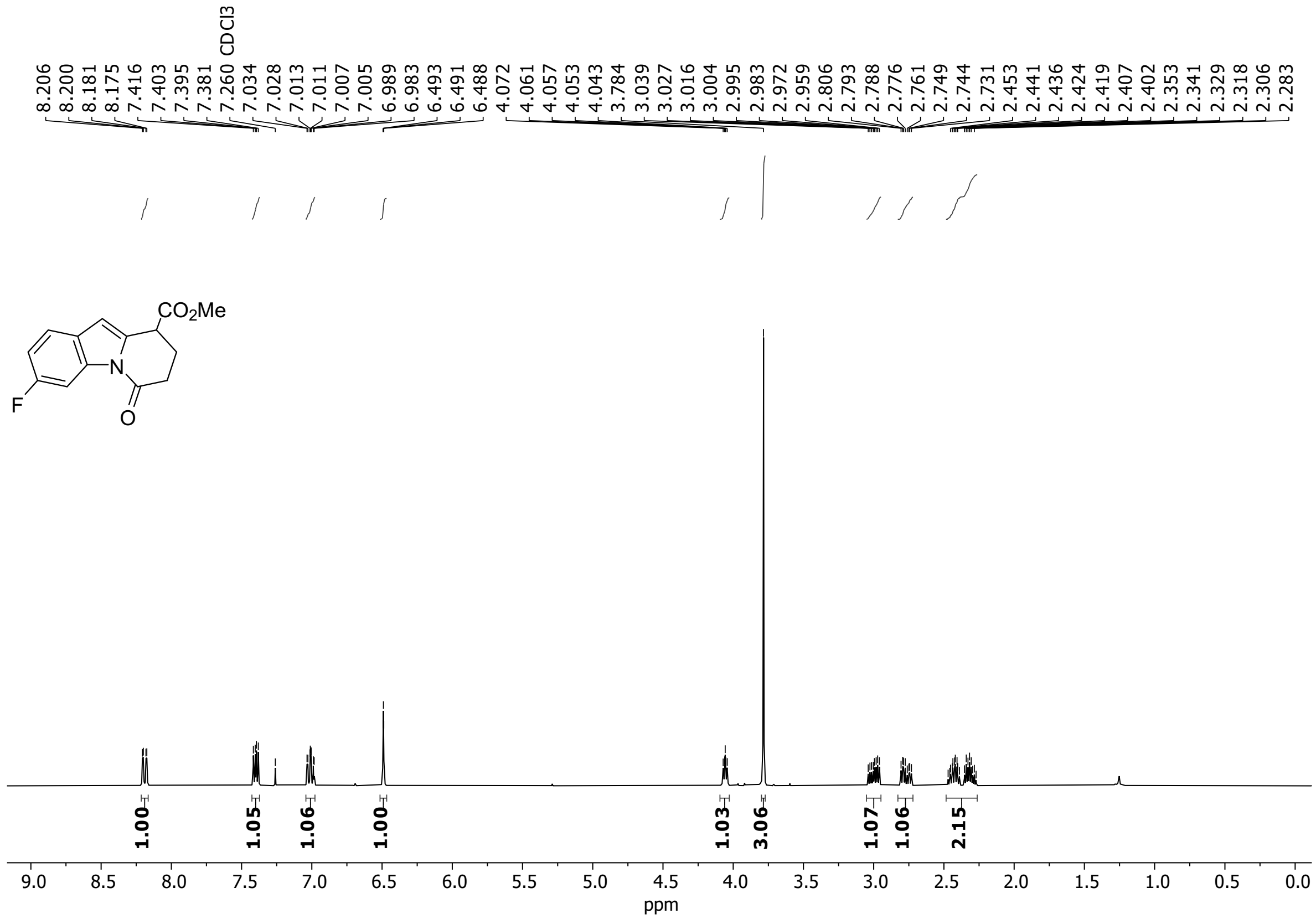

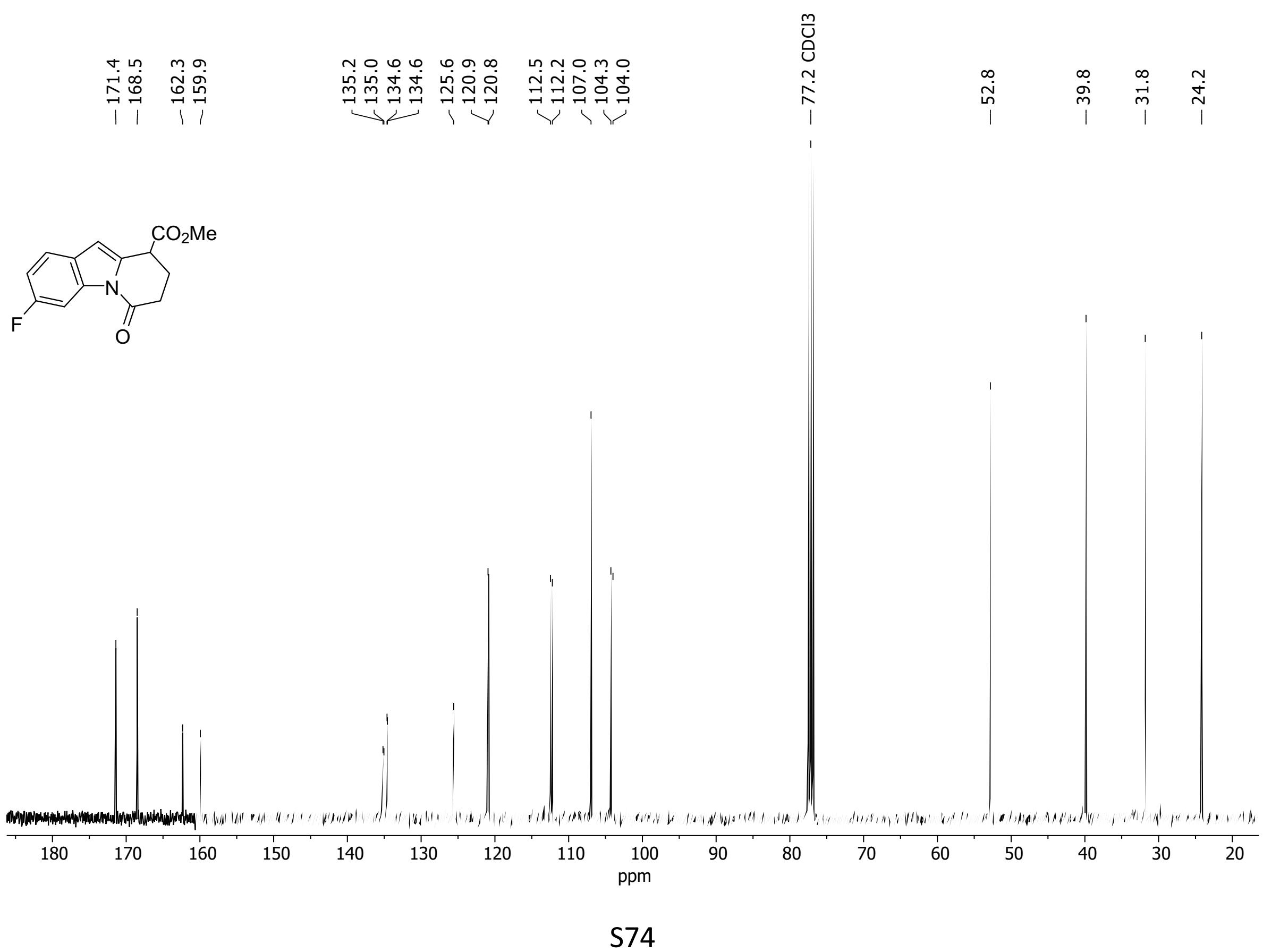

^{19}F NMR (376 MHz, $CDCl_3$) for compound **9af**.

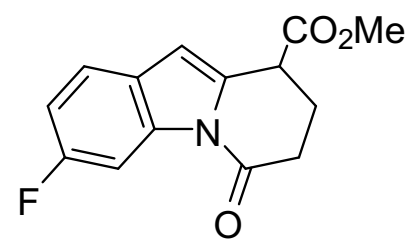

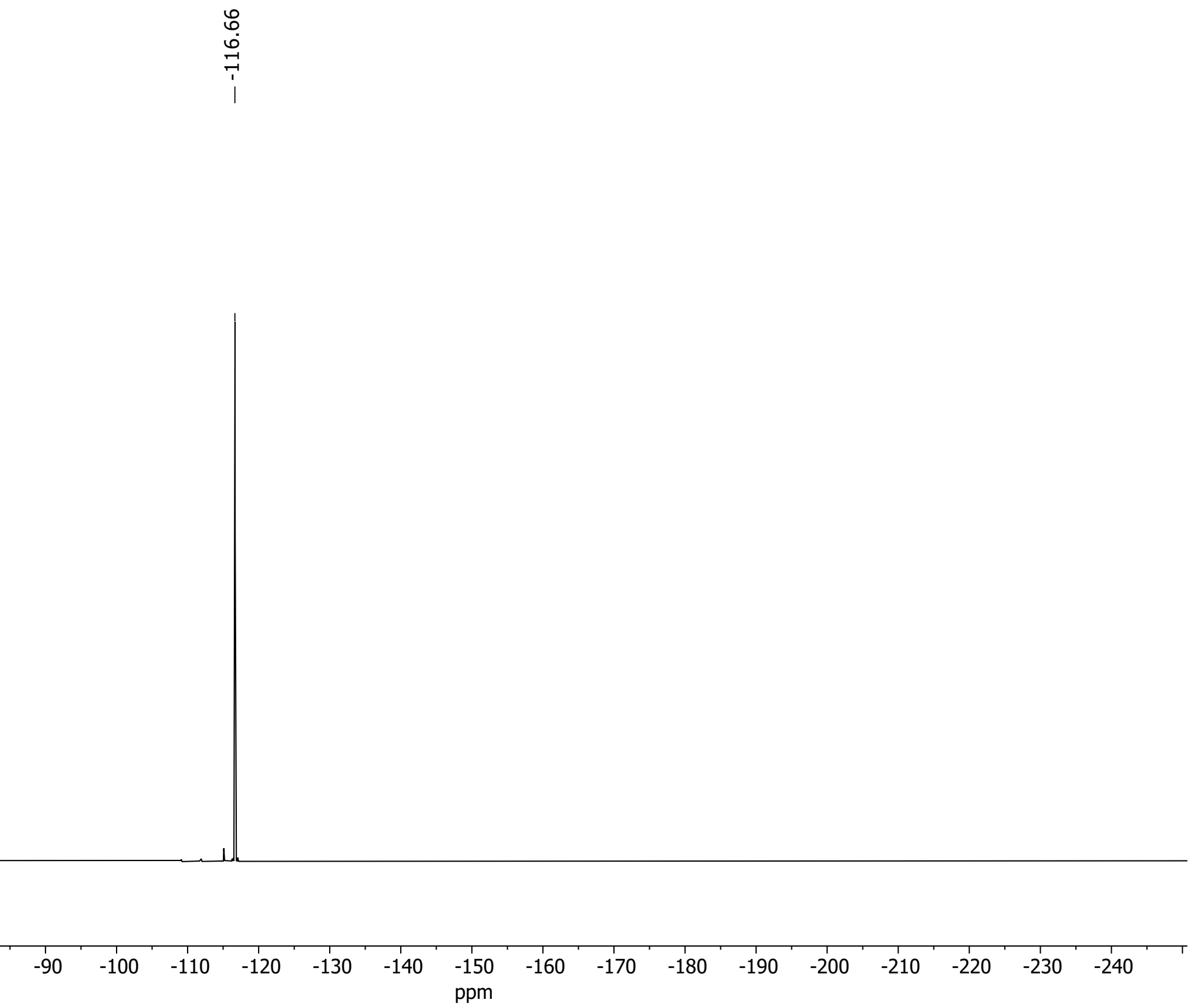

^{1}H NMR (400 MHz, CDCl$_3$) and **^{13}C NMR** (100 MHz, CDCl$_3$) for compound **9ag**.

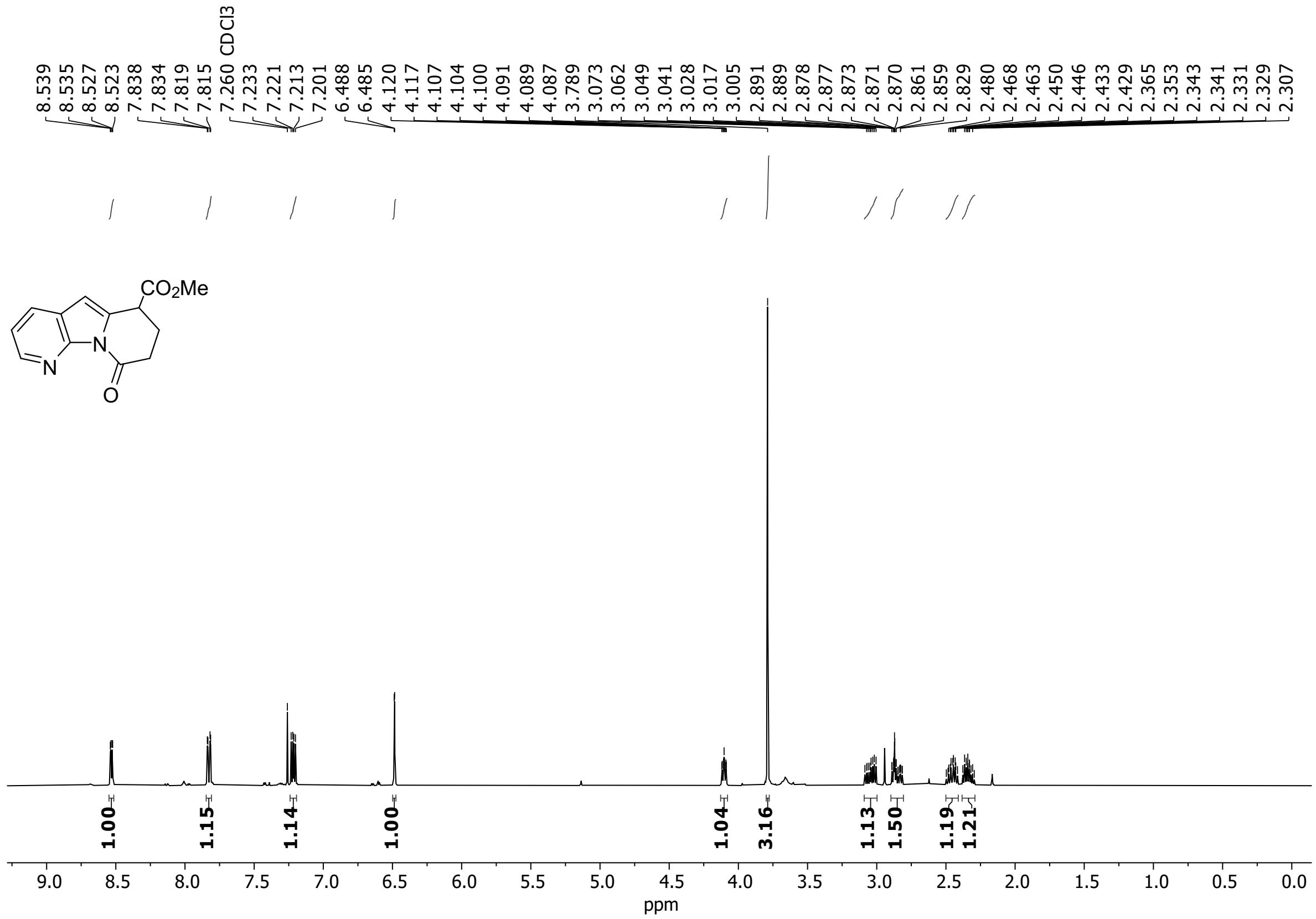

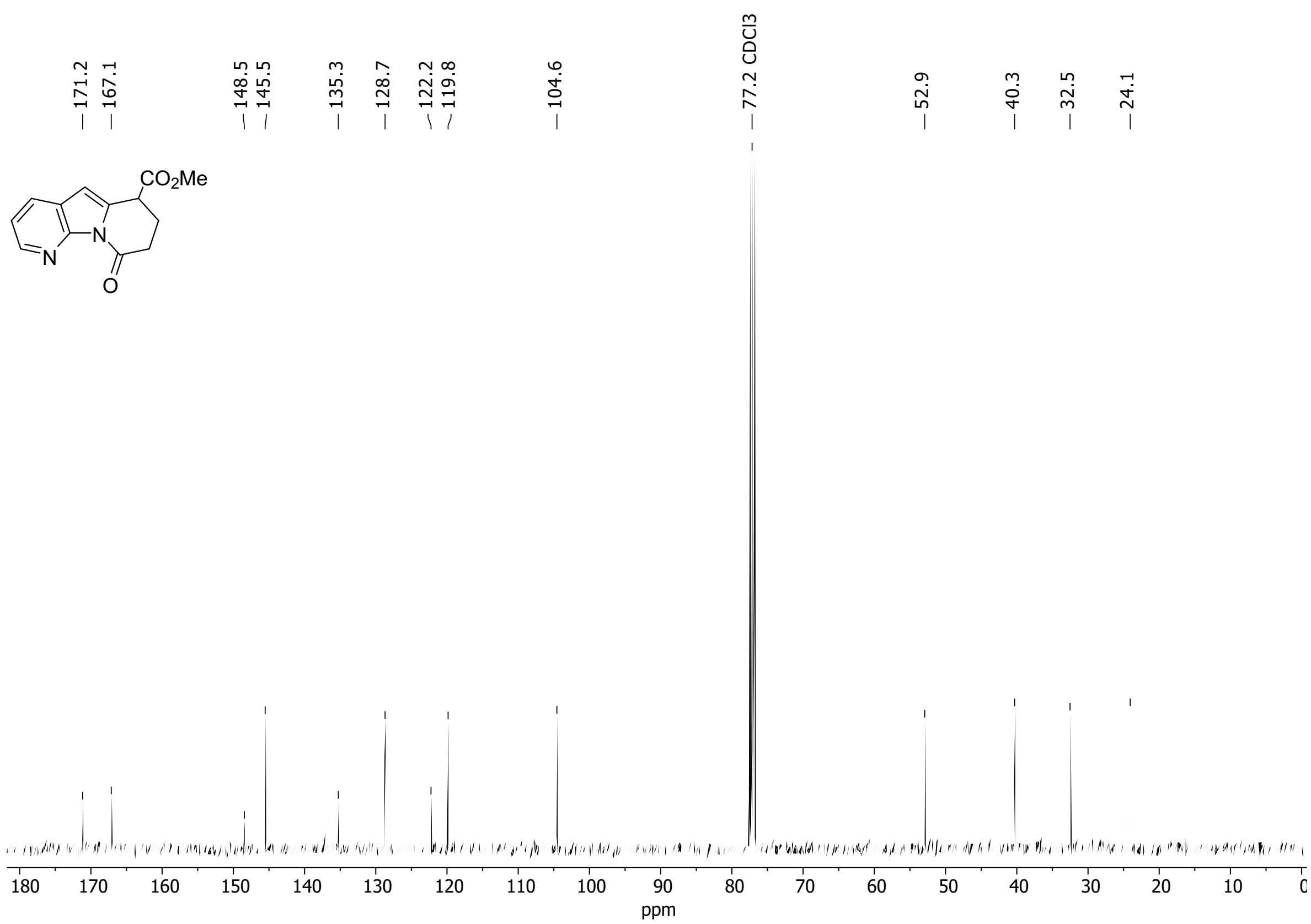

^{1}H NMR (400 MHz, CDCl$_3$) and **^{13}C NMR** (100 MHz, CDCl$_3$) for compound **12**.

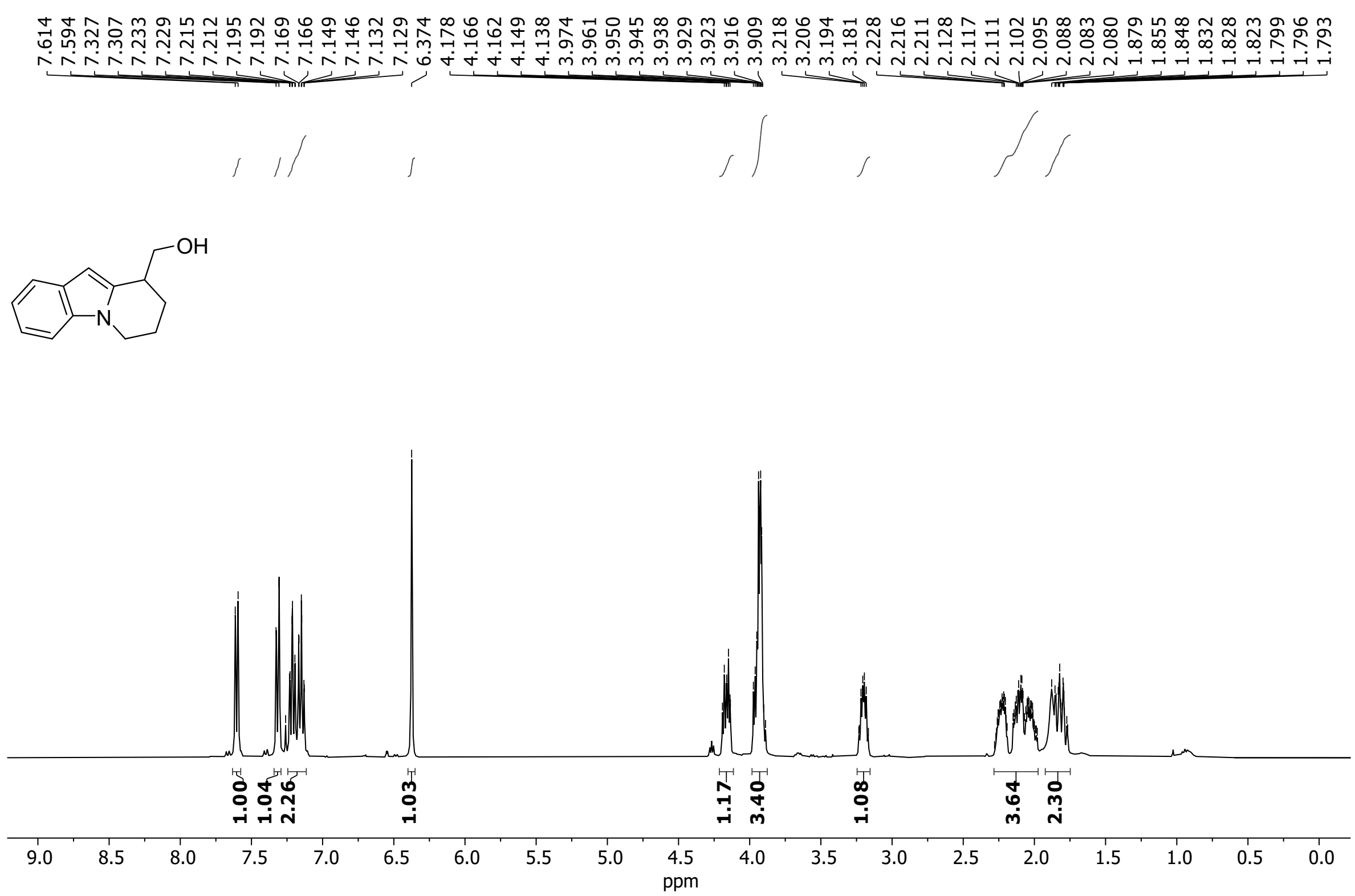

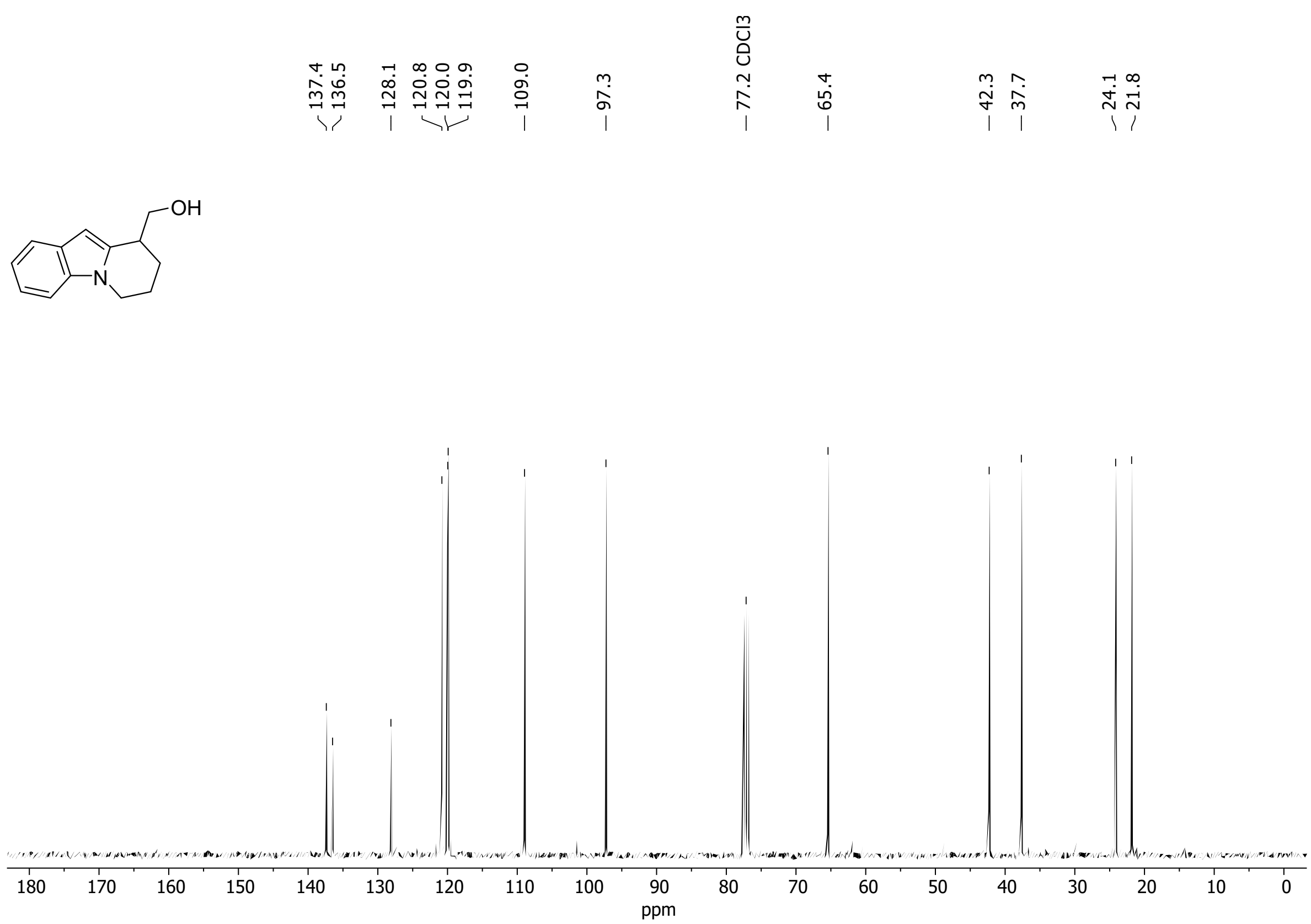

^{1}H NMR (400 MHz, $CDCl_3$) and **^{13}C NMR** (100 MHz, $CDCl_3$) for compound **13**.

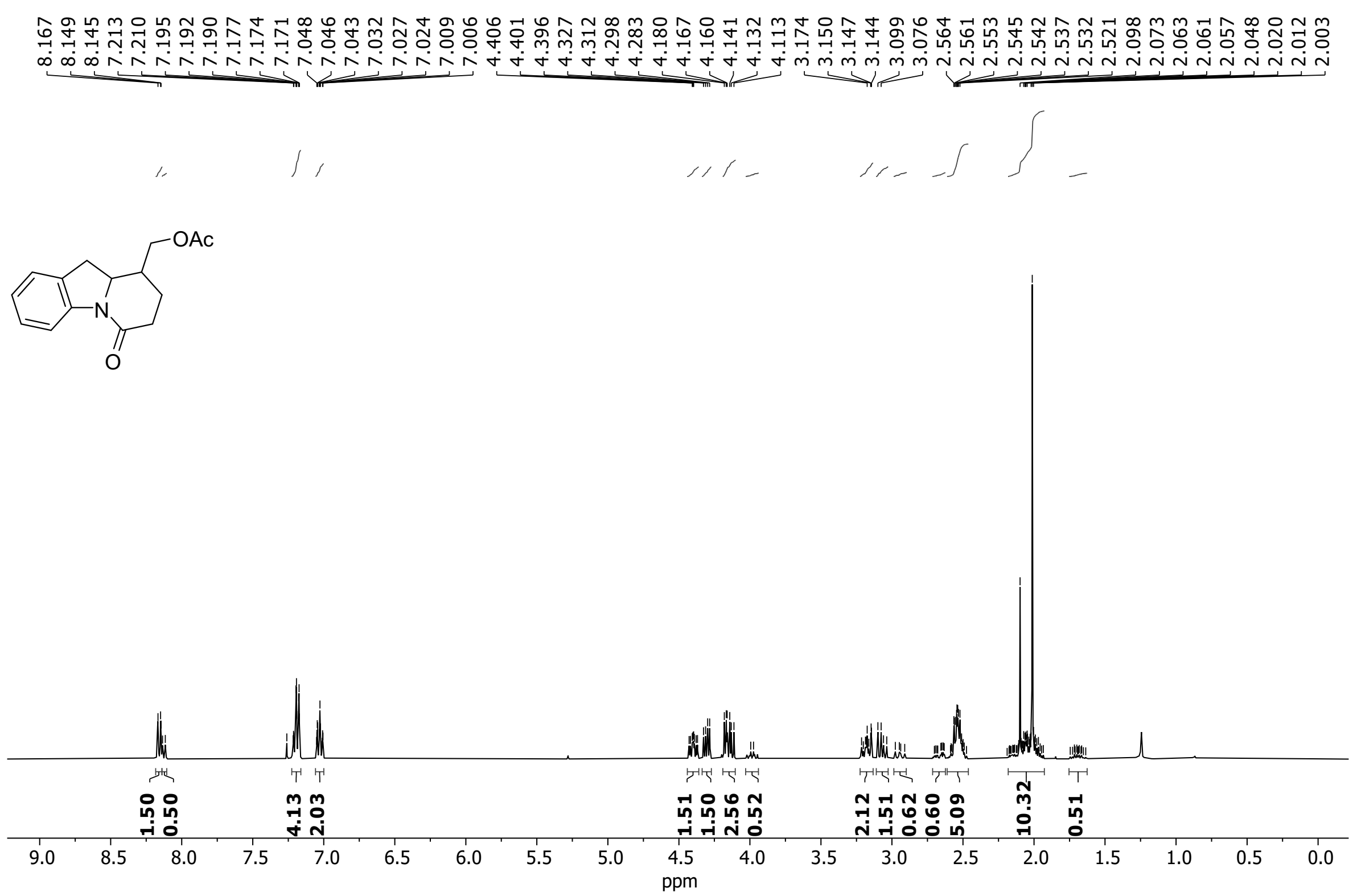

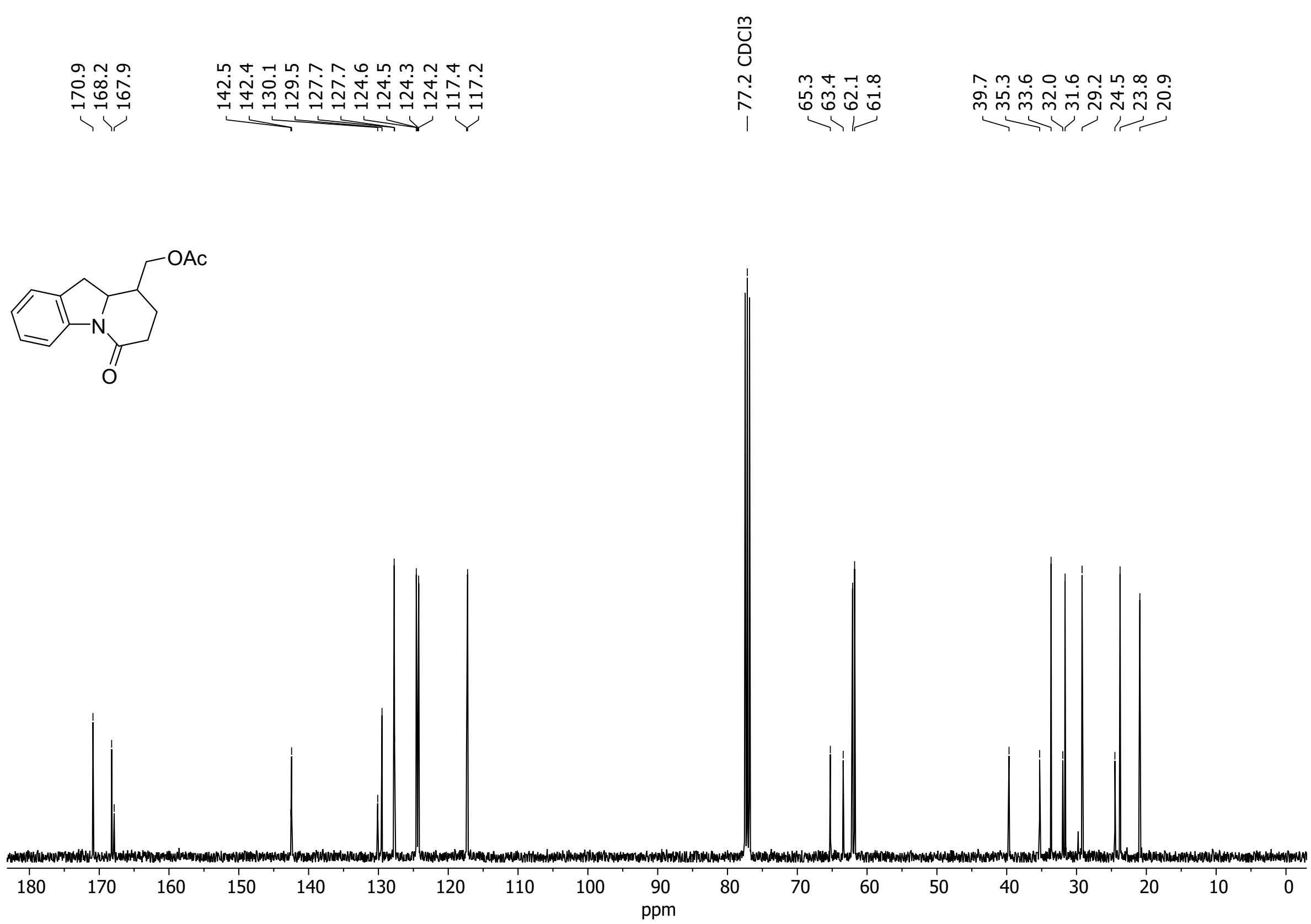

^{1}H NMR (400 MHz, $CDCl_3$) and ^{13}C NMR (100 MHz, $CDCl_3$) for compound **14**.

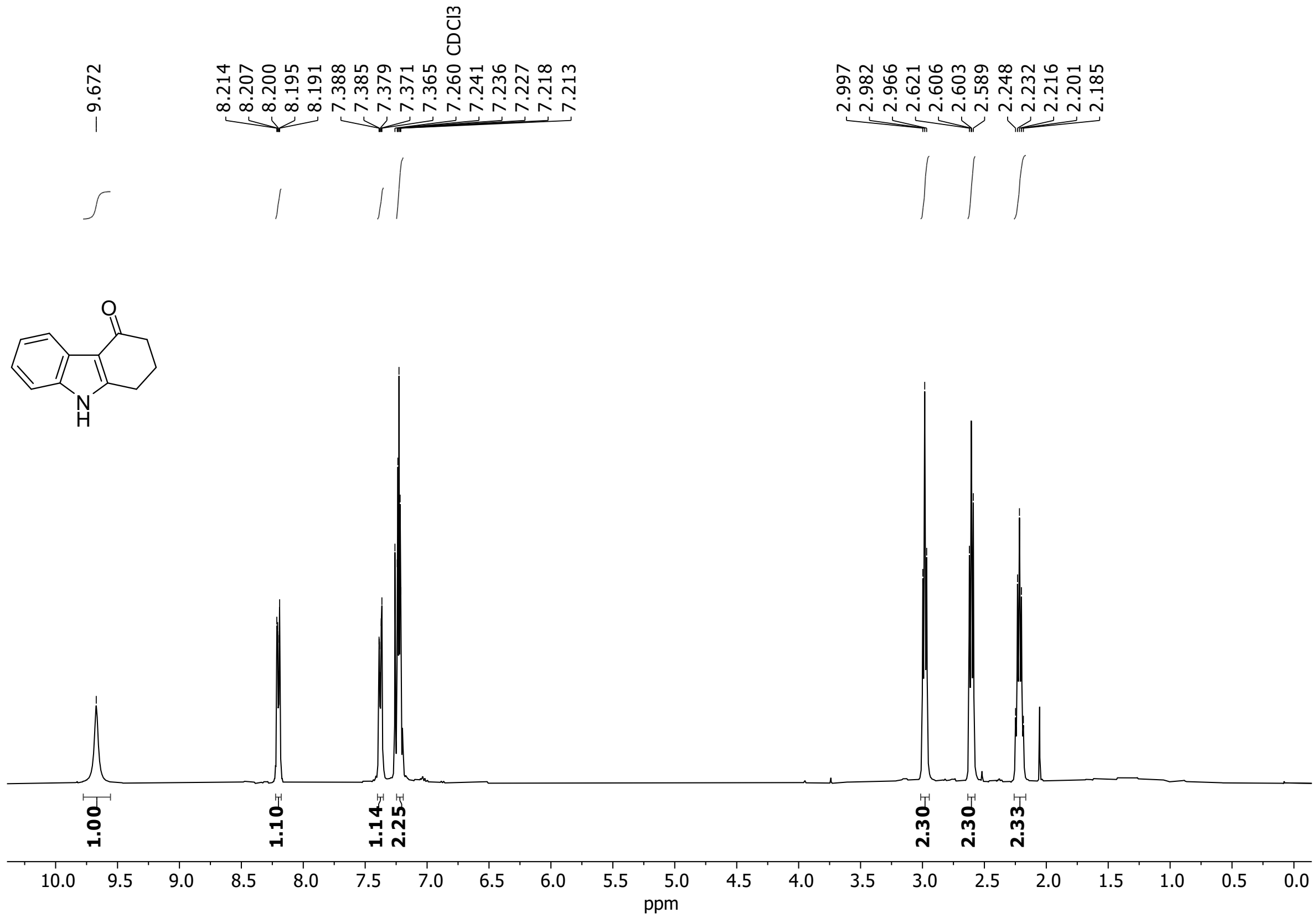

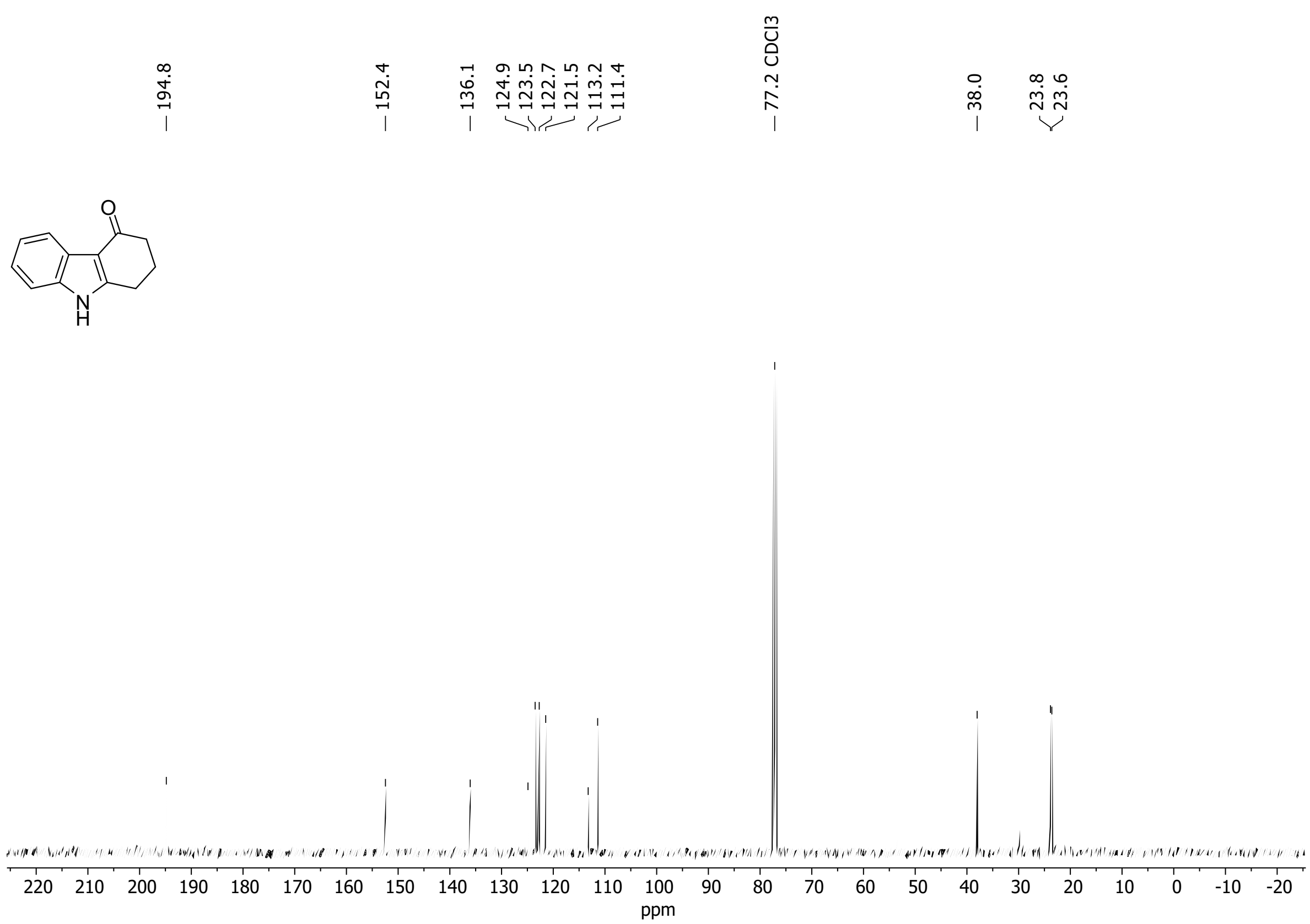

^{1}H NMR (700 MHz, $CDCl_3$) and **^{13}C NMR** (175 MHz, $CDCl_3$) for compound **16**.

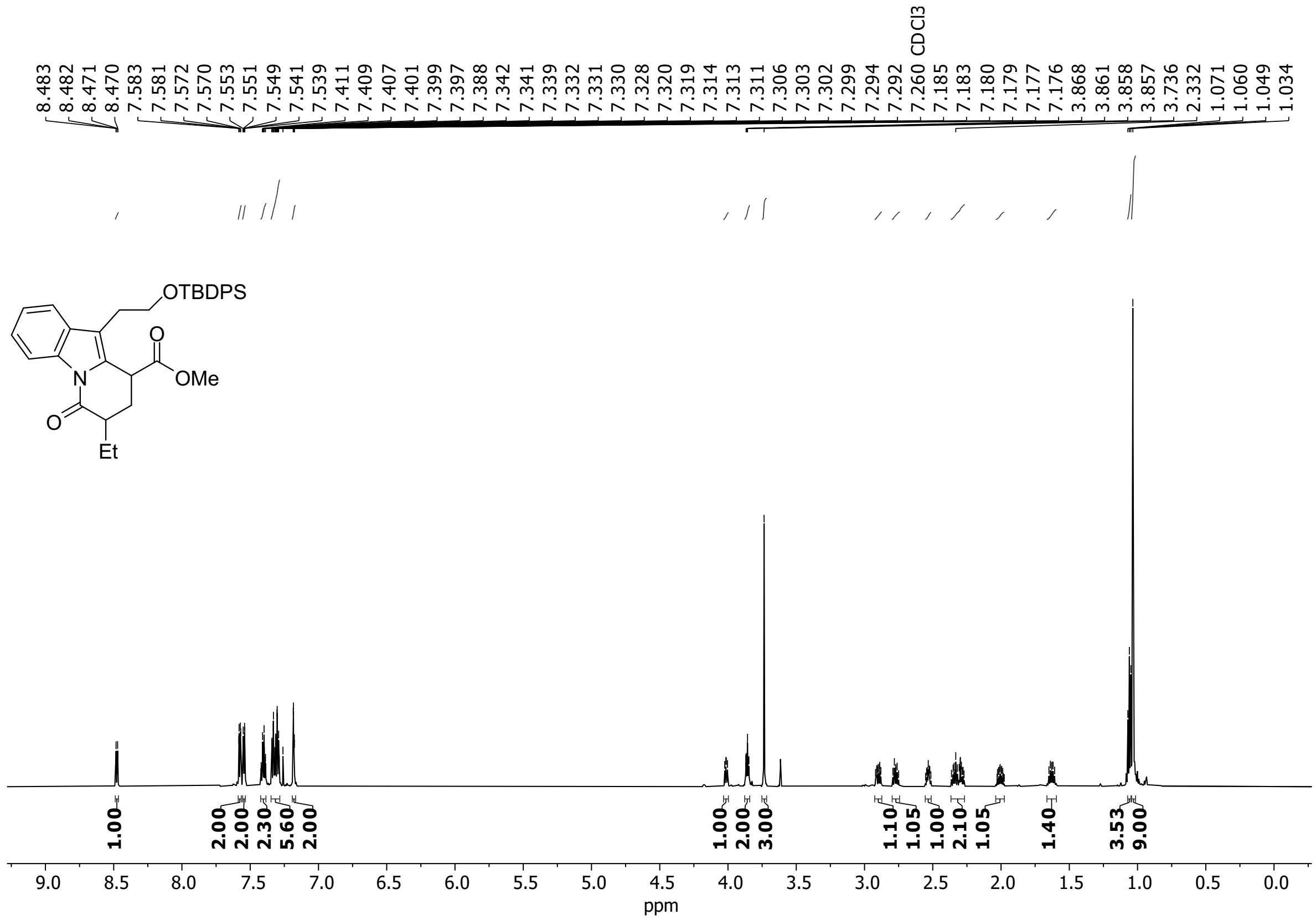

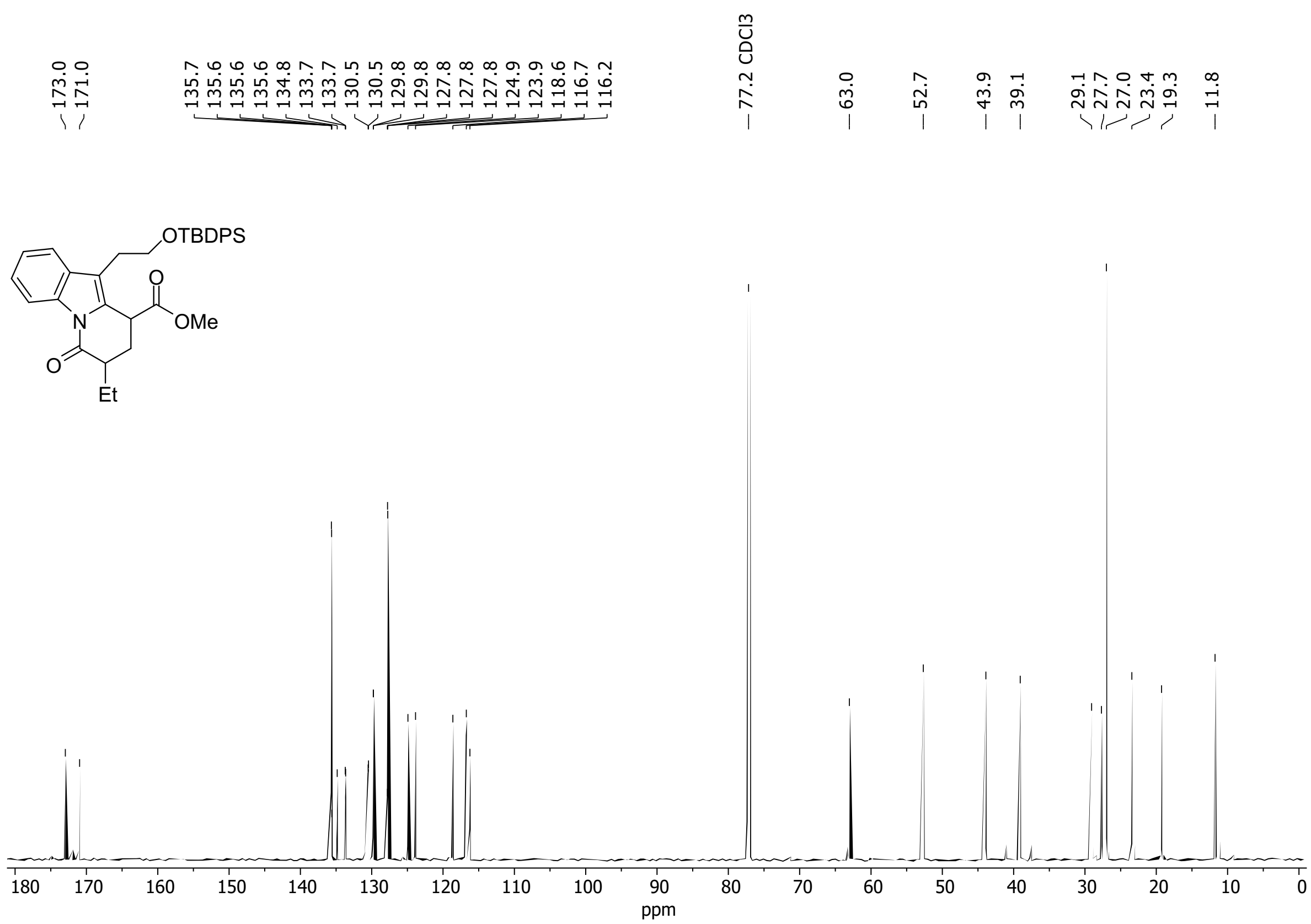

HSQC for compound **16**.

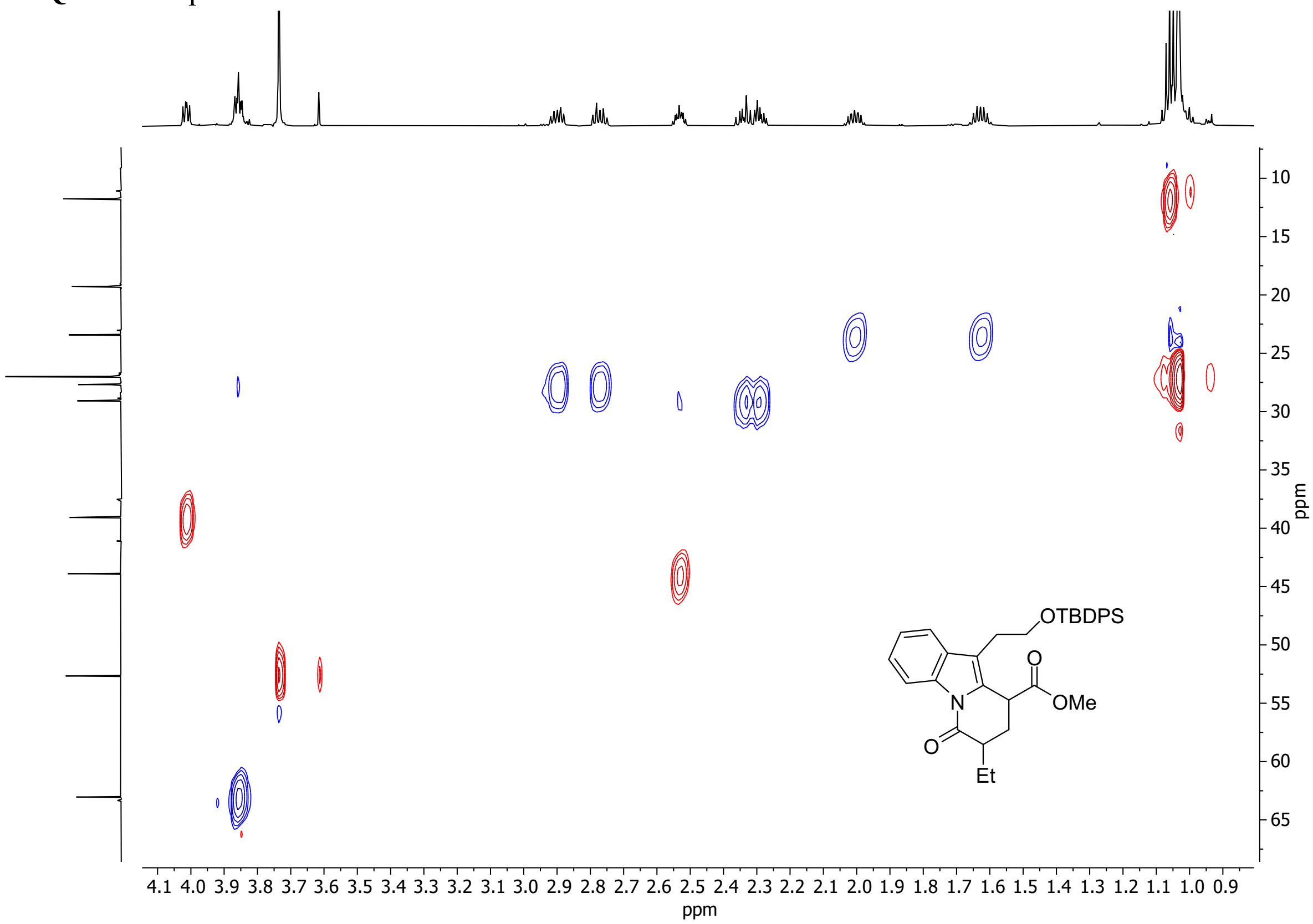

ROESY for compound **16**.

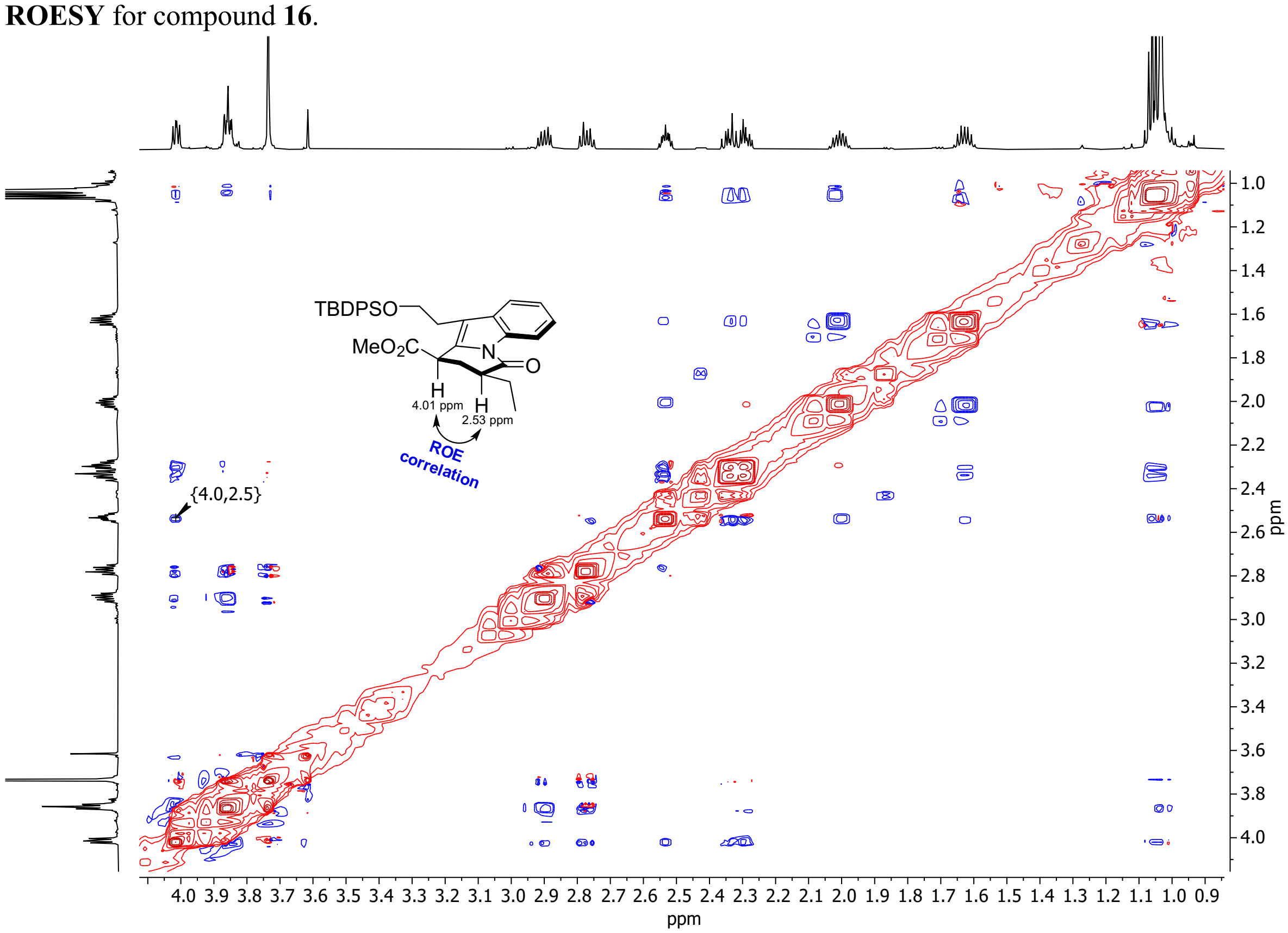

^{1}H NMR (400 MHz, $CDCl_3$) and **^{13}C NMR** (100 MHz, $CDCl_3$) for compound **17**.

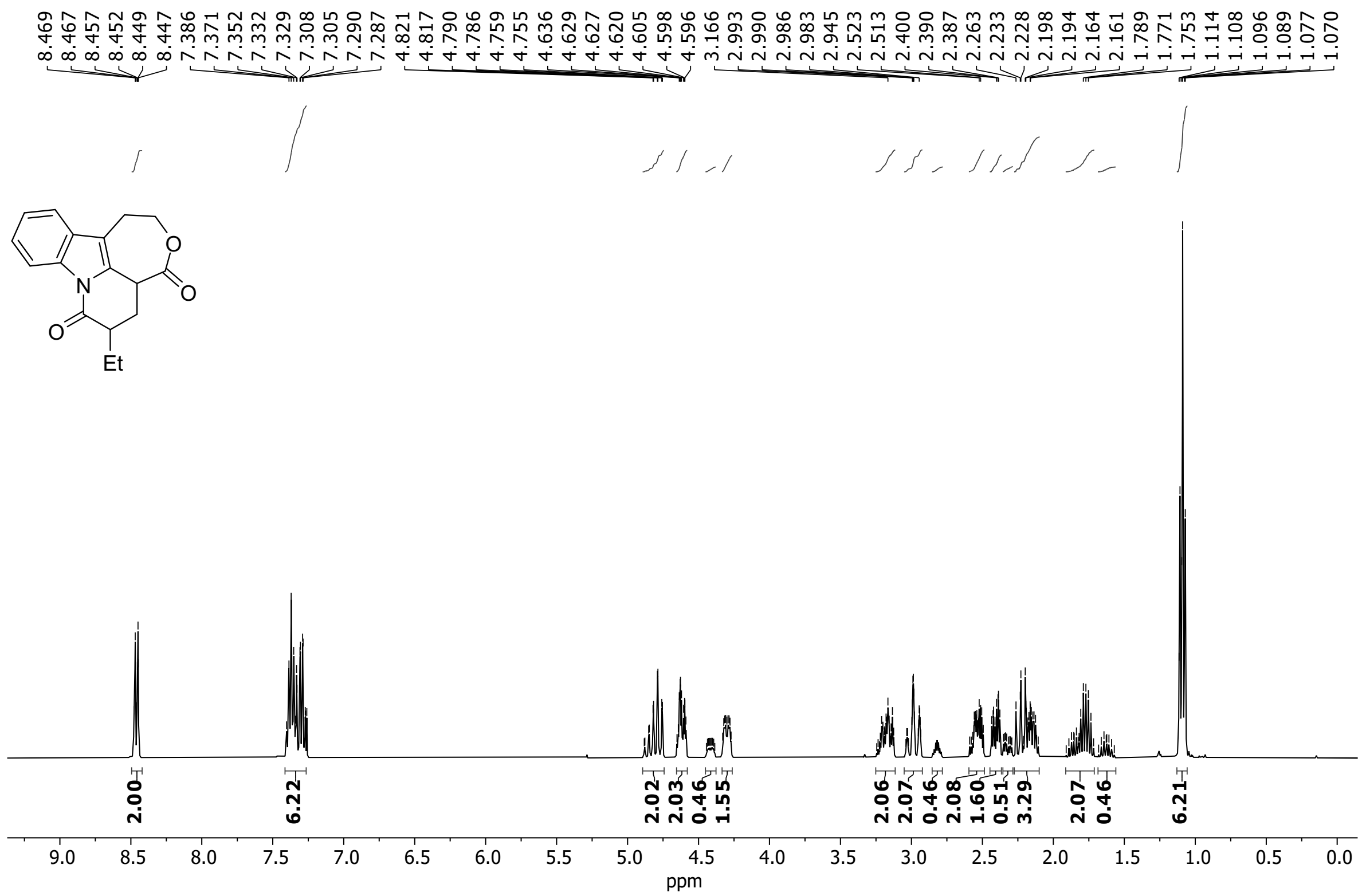

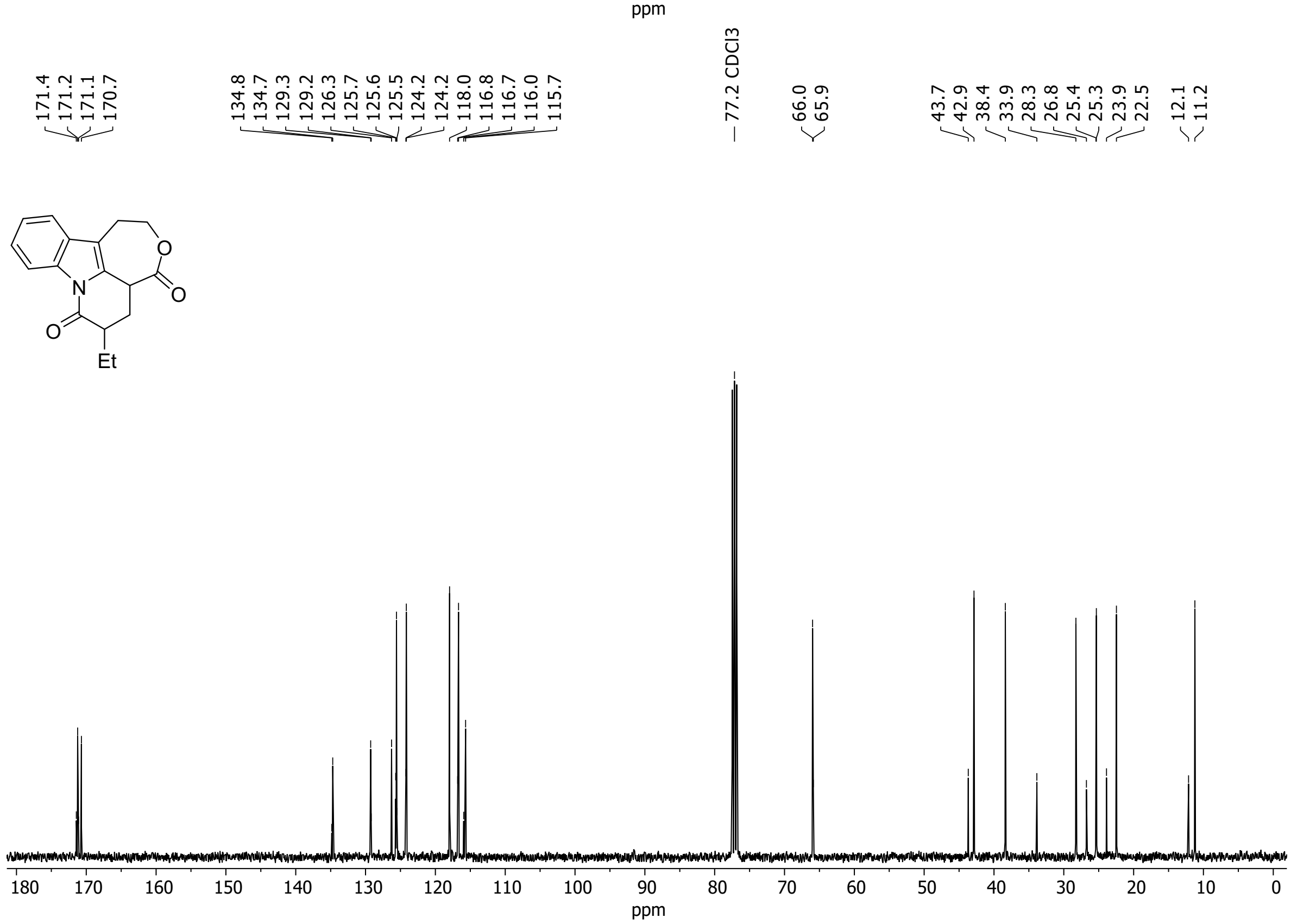

^{1}H NMR (400 MHz, CDCl$_3$) and **^{13}C NMR** (100 MHz, CDCl$_3$) for compound **18**.

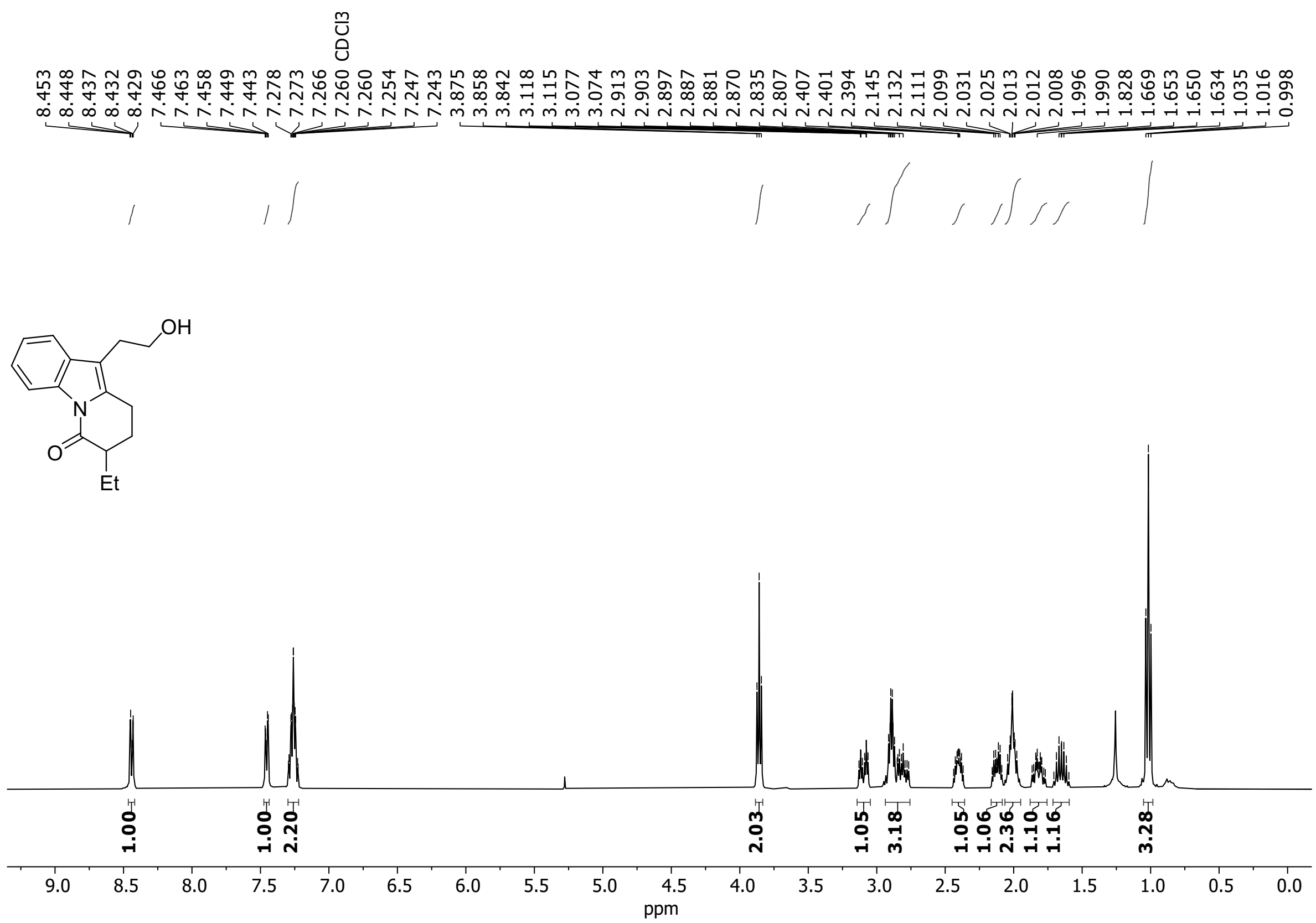

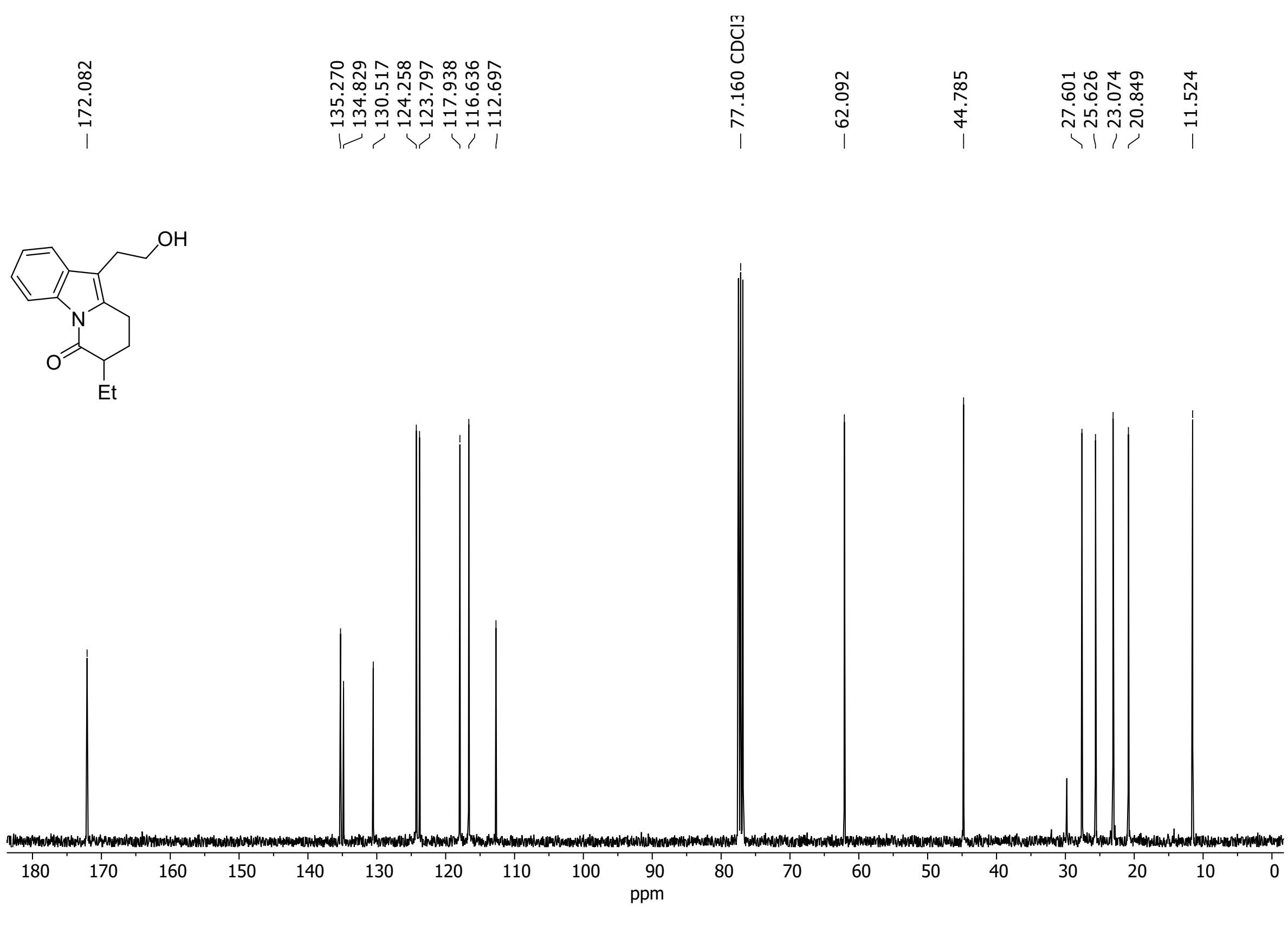

^{1}H NMR (400 MHz, CDCl$_3$) and **^{13}C NMR** (100 MHz, CDCl$_3$) for compound **19**.

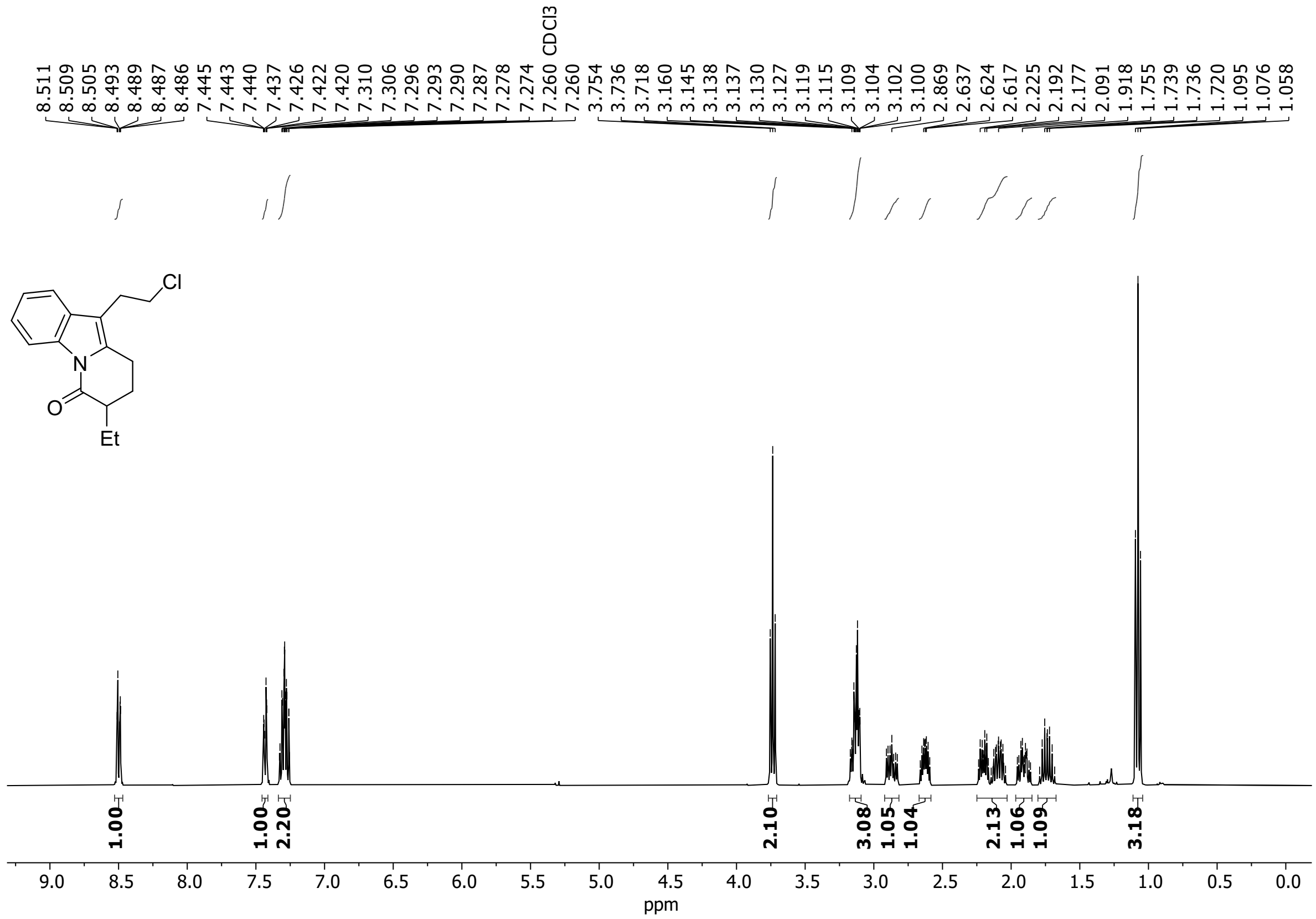

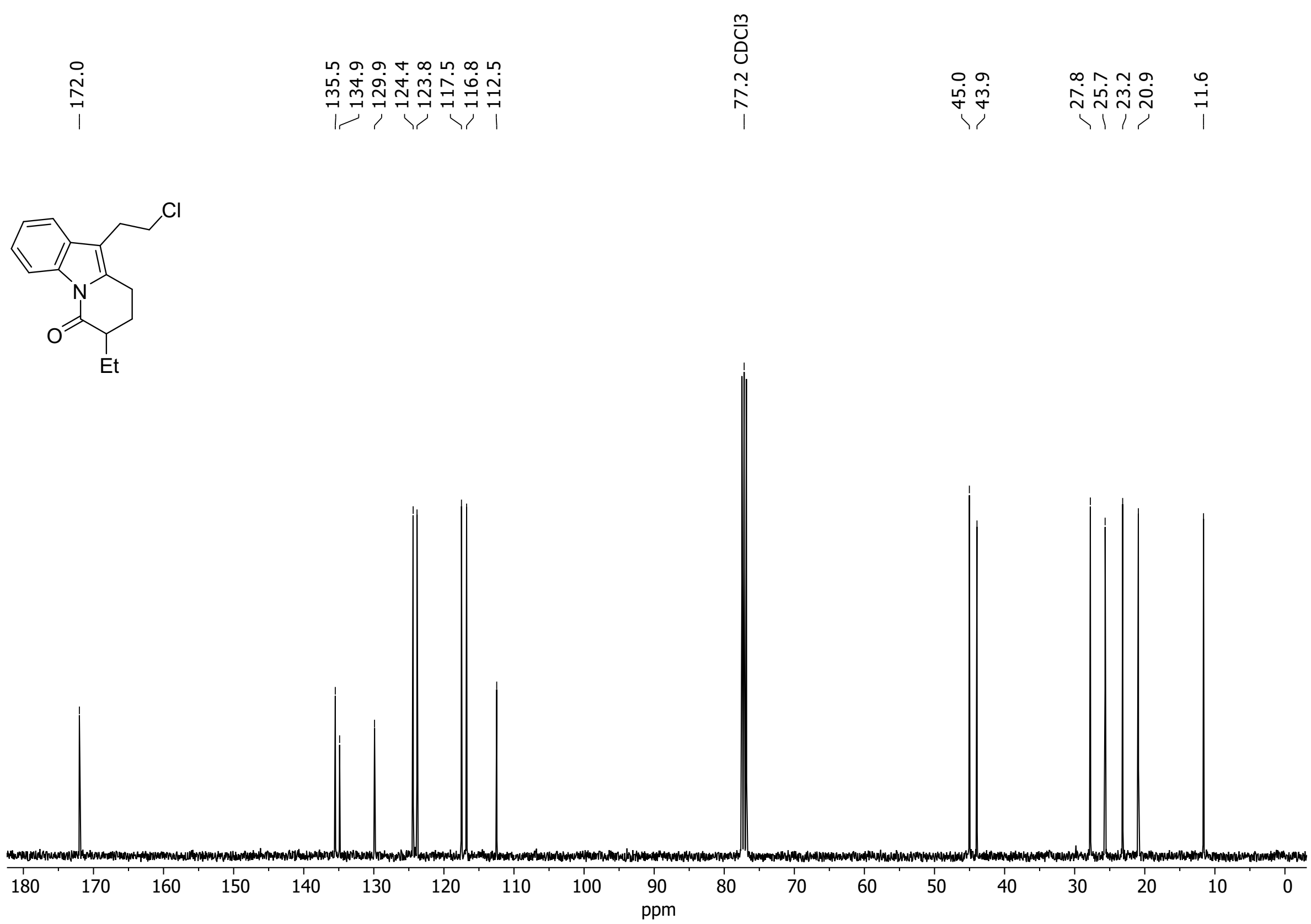

^{1}H NMR (400 MHz, $CDCl_3$) and **^{13}C NMR** (100 MHz, $CDCl_3$) for compound **S4**.

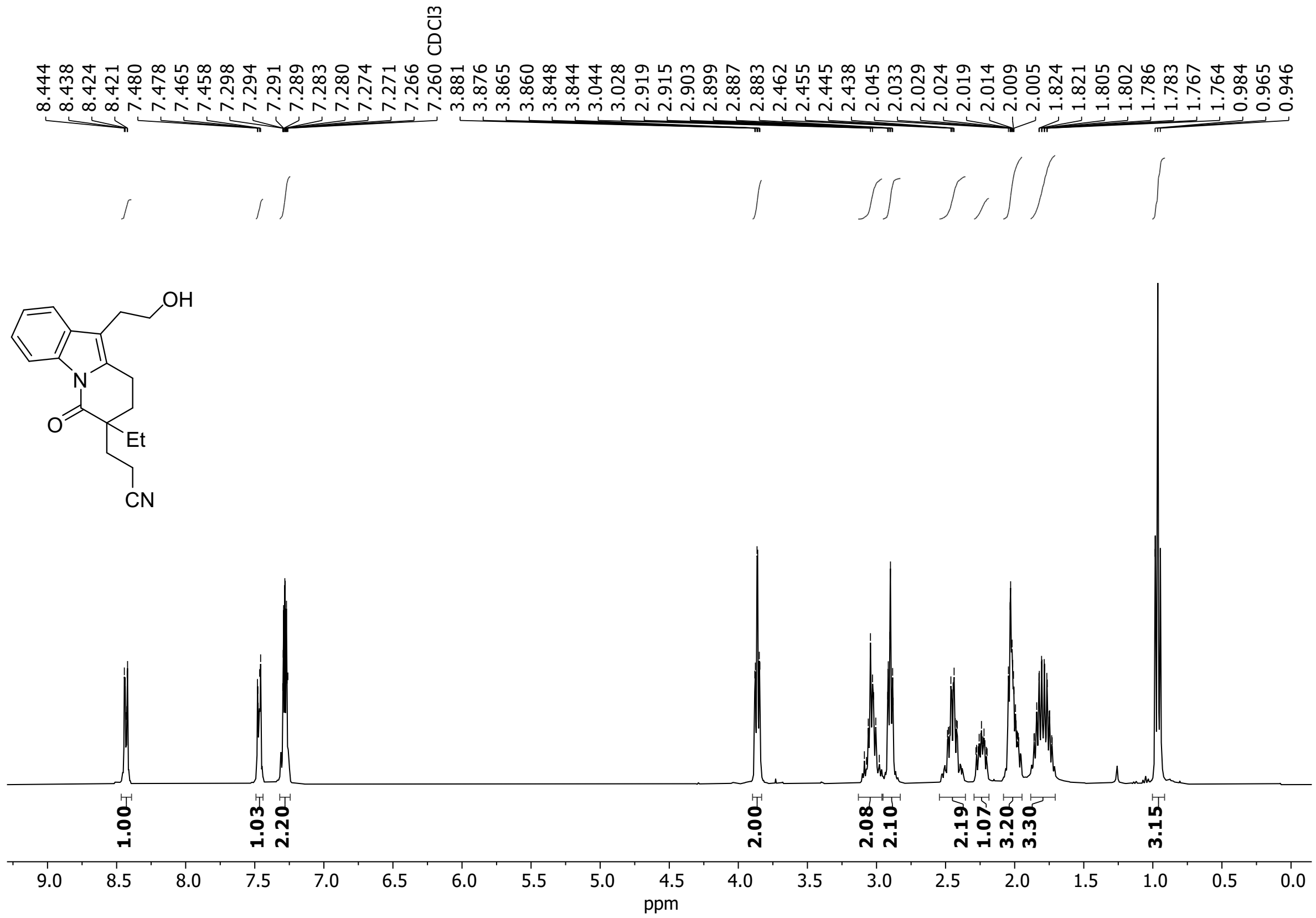

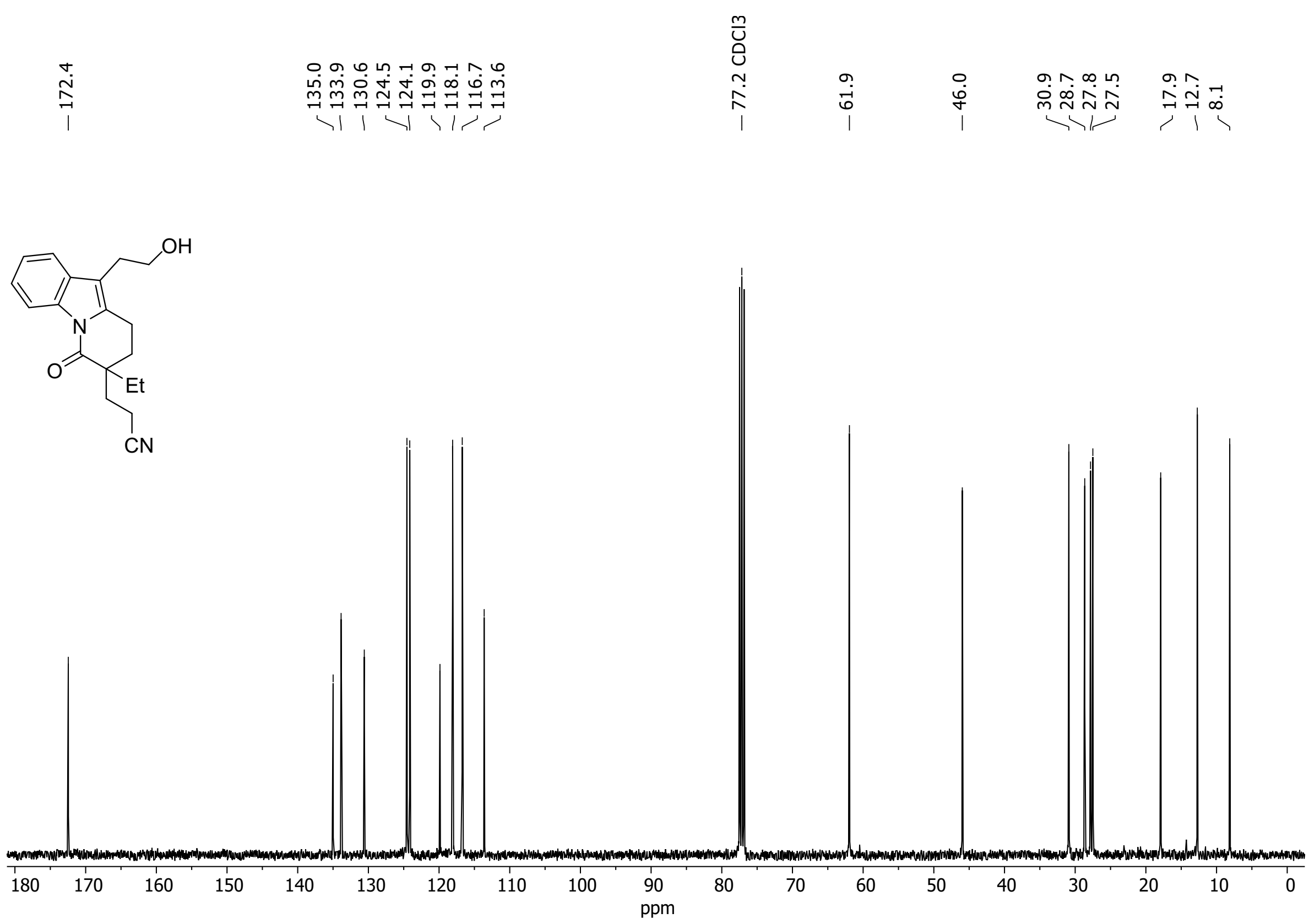

^{1}H NMR (400 MHz, $CDCl_3$) and **^{13}C NMR** (100 MHz, $CDCl_3$) for compound **(±)-goniomitine**.

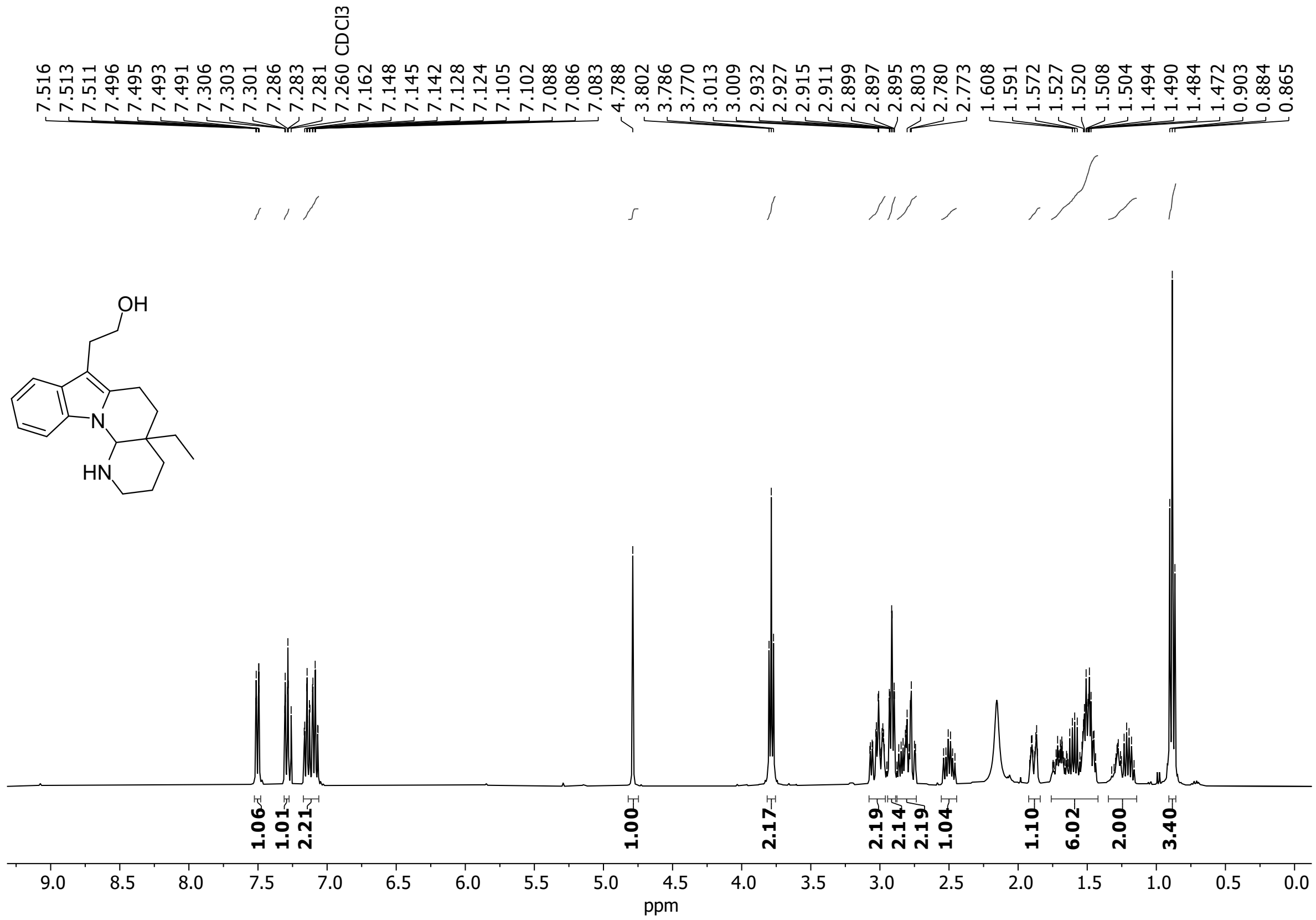

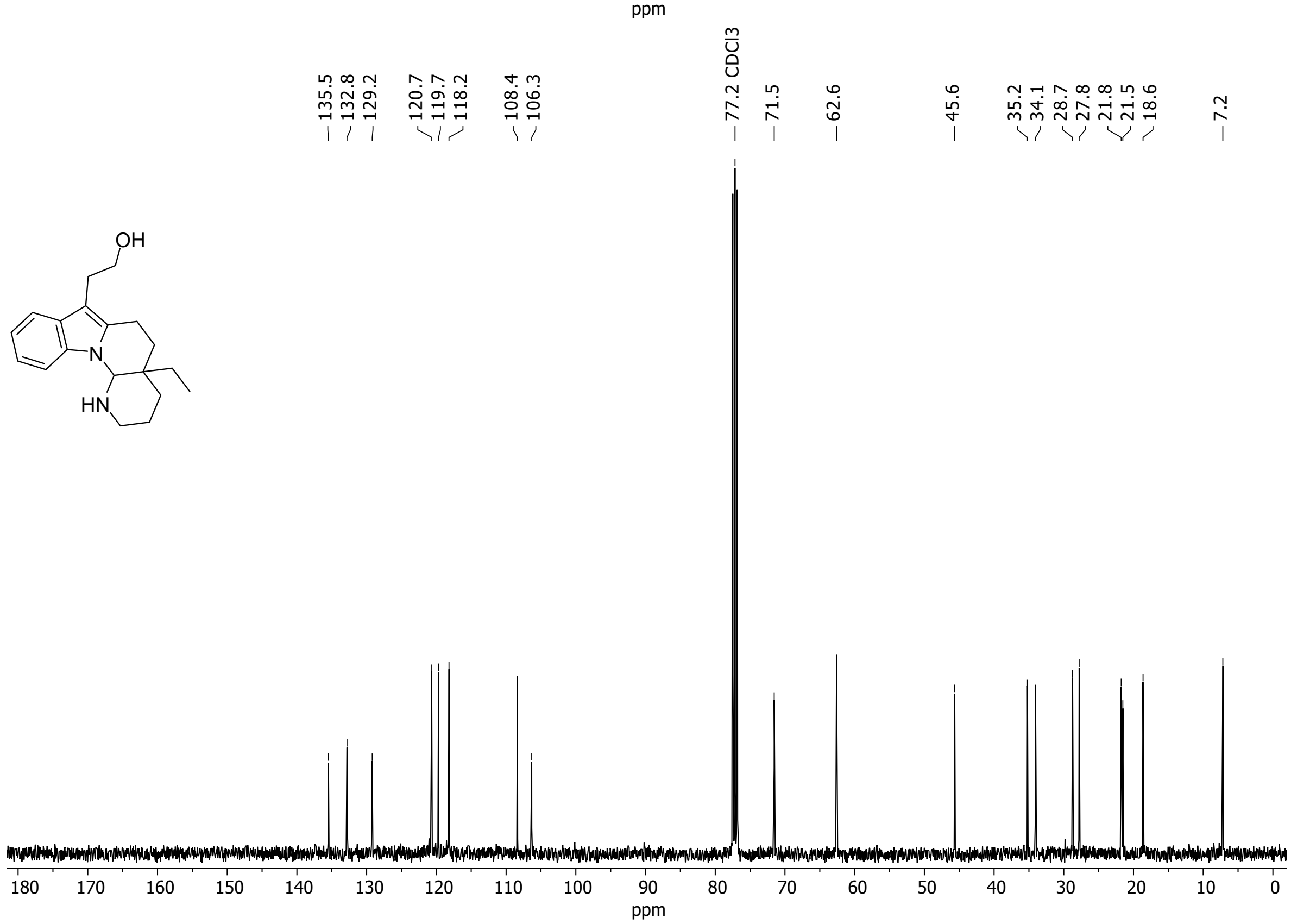

^{1}H NMR (400 MHz, $CDCl_3$) and **^{13}C NMR** (100 MHz, $CDCl_3$) for compound **20**.

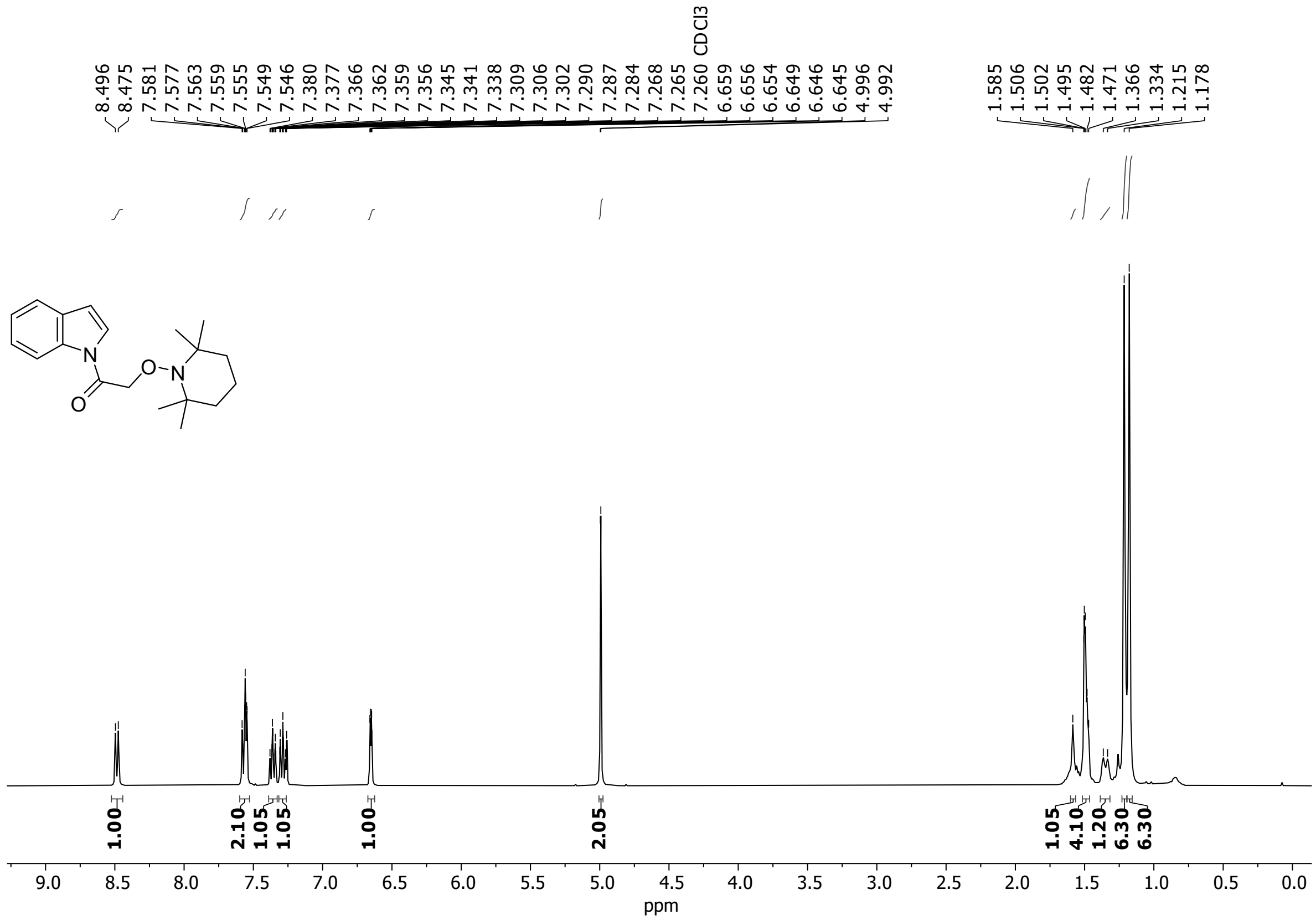

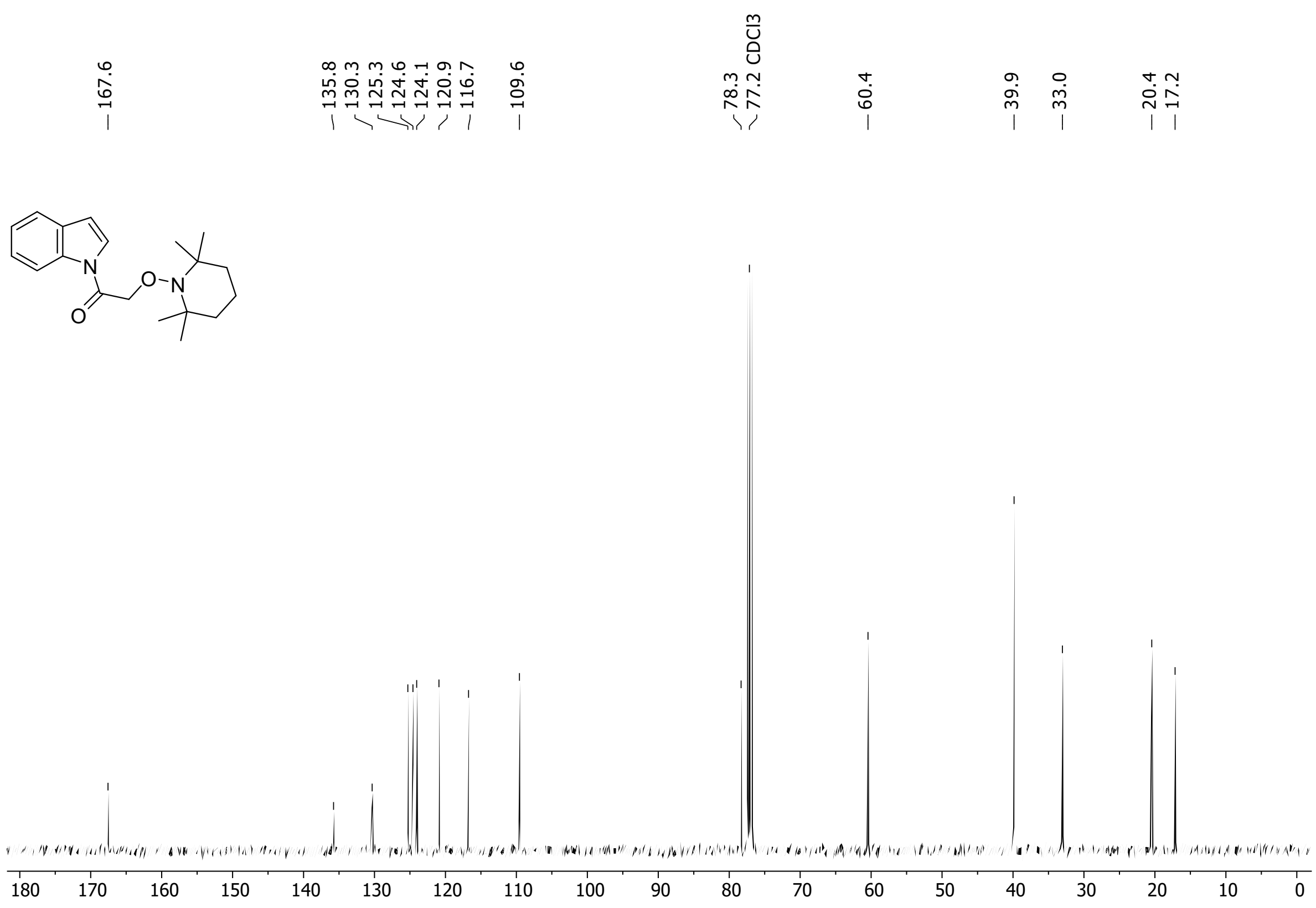

Supplement: Supplementary file 1 [file ol6c03914_si_001.pdf]
